# Aqueous Three-Component Self-Assembly of a Pseudo[1]rotaxane Using Hydrazone Bonds

**DOI:** 10.1021/acs.joc.3c00108

**Published:** 2023-04-28

**Authors:** Pablo Cortón, Natalia Fernández-Labandeira, Mauro Díaz-Abellás, Carlos Peinador, Elena Pazos, Arturo Blanco-Gómez, Marcos D. García

**Affiliations:** CICA − Centro Interdisciplinar de Química e Bioloxía and Departamento de Química, Facultad de Ciencias, Universidade da Coruña, 15071 A Coruña, Spain

## Abstract

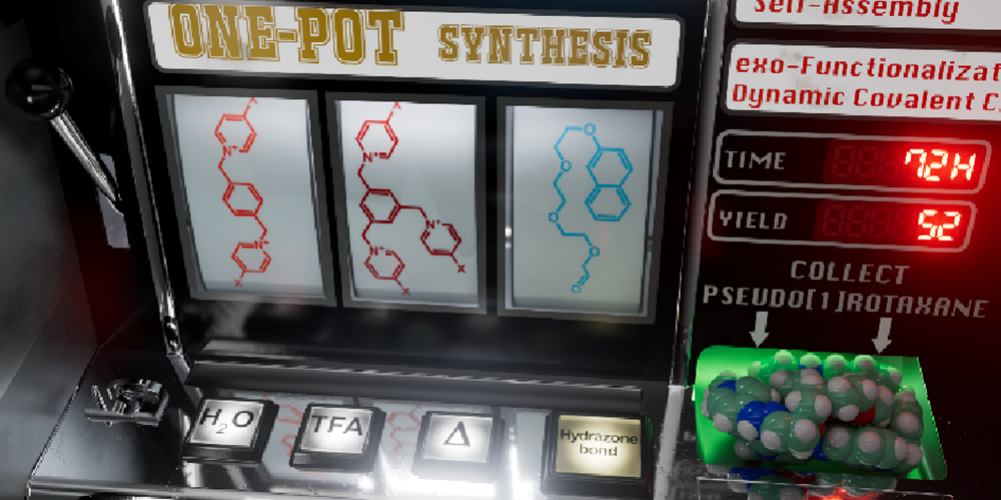

We present herein the synthesis of a new polycationic pseudo[1]rotaxane, self-assembled in excellent yield through hydrazone bonds in aqueous media of three different aldehyde and hydrazine building blocks. A thermodynamically controlled process has been studied sequentially by analyzing the [1 + 1] reaction of a bisaldehyde and a trishydrazine leading to the macrocyclic part of the system, the ability of this species to act as a molecular receptor, the conversion of a hydrazine-pending cyclophane into the pseudo[1]rotaxane and, lastly, the one-pot [1 + 1 + 1] condensation process. The latter was found to smoothly produce the target molecule through an integrative social self-sorting process, a species that was found to behave in water as a discrete self-inclusion complex below 2.5 mM concentration and to form supramolecular aggregates in the 2.5–70 mM range. Furthermore, we demonstrate how the abnormal kinetic stability of the hydrazone bonds on the macrocycle annulus can be advantageously used for the conversion of the obtained pseudo[1]rotaxane into other *exo*-functionalized macrocyclic species.

## Introduction

Contemporary host–guest macrocyclic chemistry is no longer focused exclusively on the development of new receptors with improved affinities and selectivities.^1^ Current challenges also include the introduction within the macrocycle of stimuli-responsiveness,^2^ constitutional dynamism,^3^ or *exo*-functionalization,^4^ as well as the implementation of the host’s recognition capabilities in aqueous media or complex biological milieu.^5^ In this context of increasing complexity of the targeted macrocyclic hosts, it should be noted that although multistep kinetically controlled syntheses are quite reliable in terms of structure feasibility, they are also tedious and result in low yields, mainly because of the poor performance of the key macrocyclization step.^6^ Conversely, self-assembly methodologies substantially improve the reaction yields of this cyclization process^7,8^ but significantly limit the range of pre-/postmodification of the macrocycle due to the potential functional group interference. Consequently, there is still plenty of room for the development of general orthogonal self-assembly strategies capable of producing complex macrocyclic-based (supra)molecules,^9^ for instance, by using integrative social self-sorting processes that yield complex asymmetric macrocycle-based species from simple building blocks.^10^

**Scheme 1:**
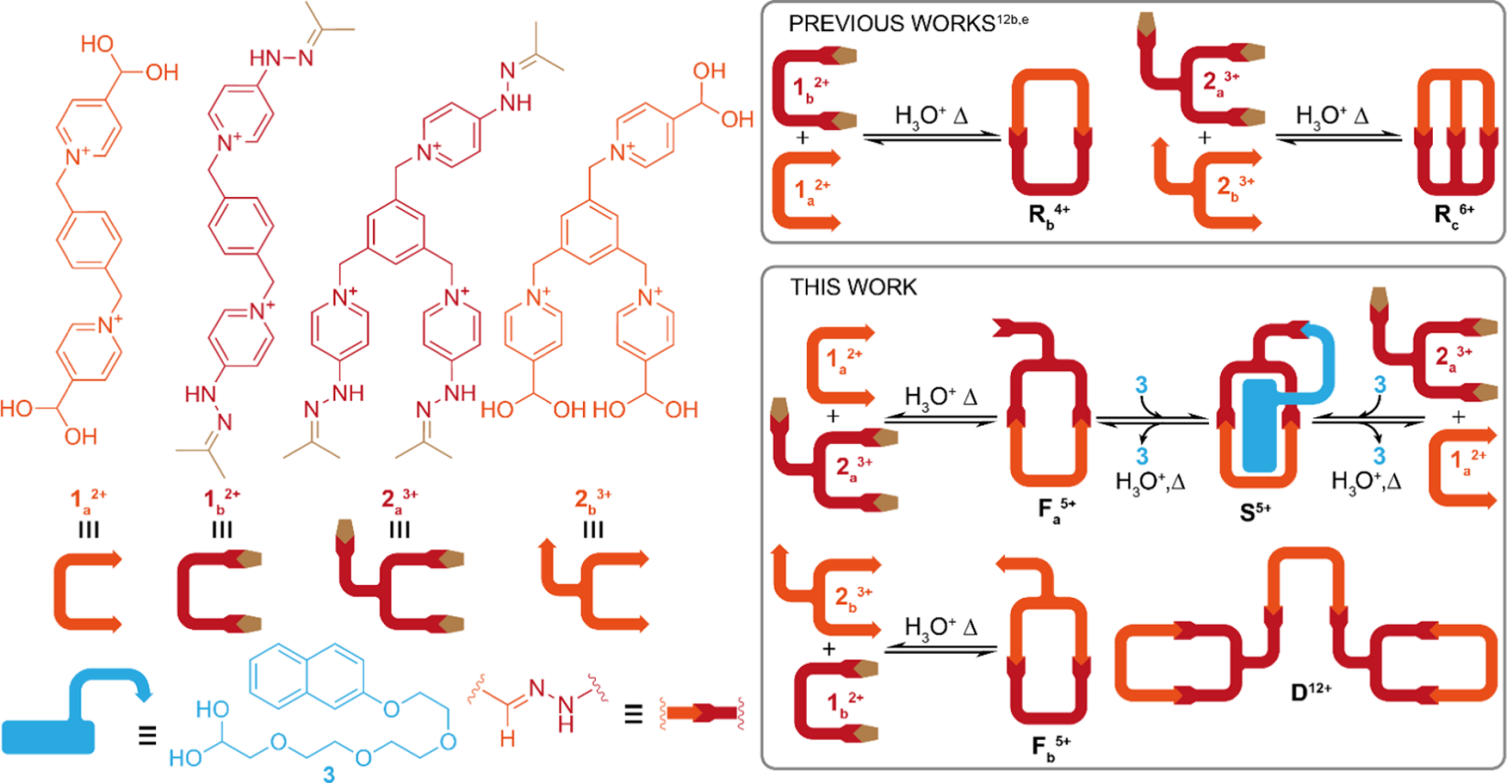
Structures of the Building Blocks and Main Self-Assembly Processes Discussed in This Work

Following our interest in the use of aqueous imine-based self-assembly in supramolecular chemistry,^11,12^ we have shown that the TFA-catalyzed hydrazone exchange reaction between complementary ditopic [**1**_**a**_^2+^ + **1**_**b**_^2+^] and tritopic [**2**_**a**_^3+^ + **2**_**b**_^3+^] building blocks smoothly produced [1 + 1] condensations in water, leading to the rectangular cyclophane “red box”^12b^ or its macrobicyclic analogue “red cage”^12e^ as the sole detectable species (**R**_**b**_^4+^ and **R**_**c**_^6+^, Scheme 1). The highly delocalized nature of the bispyridinium hydrazone bonds formed translates into two key features of **R**_**b**_^4+^/**R**_**c**_^6+^: high hydrolytic stability of the hydrazone bonds and abnormal p*K*_a_ of the amino groups (∼9).

Encouraged by these previous results, we turned our attention to the use of the above-mentioned building blocks for the self-assembly of more complex architectures, in particular by using the mismatched reaction partners **1**_**a**_^2+^/**2**_**a**_^3+^, in combination with the aldehyde **3**, for the self-assembly of the pseudo[1]rotaxane **S**^5+^ (Scheme 1). Apart from our interest in these structurally appealing targets,^13^ they have also shown utility as precursors of mechanically interlocked^14^ or supramolecular daisy chains.^15^ As depicted, the self-assembly of the bisaldehyde **1**_**a**_^2+^ and the trishydrazine **2**_**a**_^3+^ can potentially produce the [1 + 1] cyclophane **F**_**a**_^5+^, among other species. This, in turn, could be conveniently trapped by the aliphatic aldehyde **3**, which contains an ethylene glycol-based linker that would react with the appended hydrazine in **F**_**a**_^5+^ and a naphthalene moiety that could potentially be inserted into the cavity of the macrocycle. Consequently, the addition of **3** would conveniently push the equilibrium to the smallest cyclic species since the global thermodynamic minimum, the self-inclusion complex **S**^5+^, would maximize the number of hydrazone bonds and intramolecular interactions per self-assembled unit.^13−15^

## Results and Discussion

### Two-Component Self-Assembly of Functionalized Macrocycles

**Figure 1:**
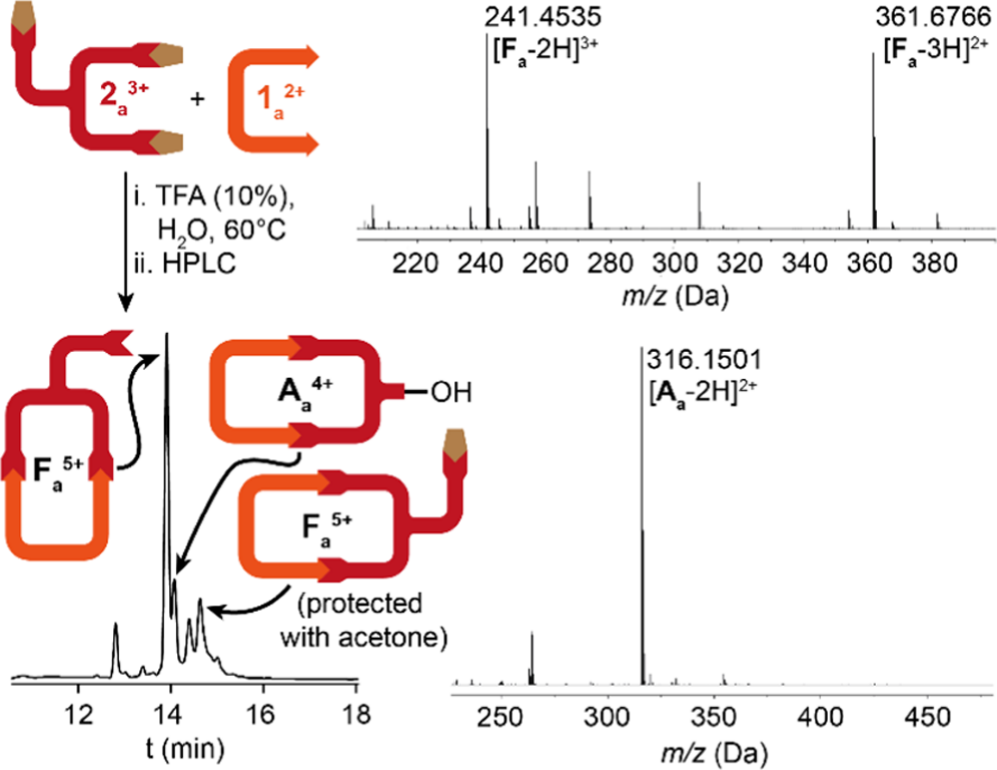
Schematic representation of the typical outcome of the reaction [**1**_**a**_·2Br + **2**_**a**_·3Br], showing the HPLC chromatogram at 220 nm and HR-ESI-MS of the main species detected.

As the starting point of our study, and in order to simplify the analysis of the results leading to the complex pseudo[1]rotaxane structure **S**^5+^, we first tested the self-assembly of the mismatched building blocks [**1**_**a**_·2Br + **2**_**a**_·3Br] using our standard synthetic protocol: heating an equimolar mixture of the species in H_2_O (2.5 mM) using 10% TFA as the catalyst at 60 °C in a round-bottom flask with a condenser.^12^ Monitoring of the reaction mixture by ^1^H NMR showed the results after 24 h, in good agreement with the complete consumption of the starting materials. After workup, the crude reaction mixture was purified by reverse-phase semipreparative HPLC and the main species obtained were analyzed by HR-ESI-MS (Figure 1). Pleasingly, we found that the [1 + 1] macrocyclic product **F**_**a**_·5TFA was obtained as the main species in an acceptable yield of 34%,^16^ which was fully characterized by 1D/2D NMR and HR-ESI-MS.^17^

**Figure 2:**
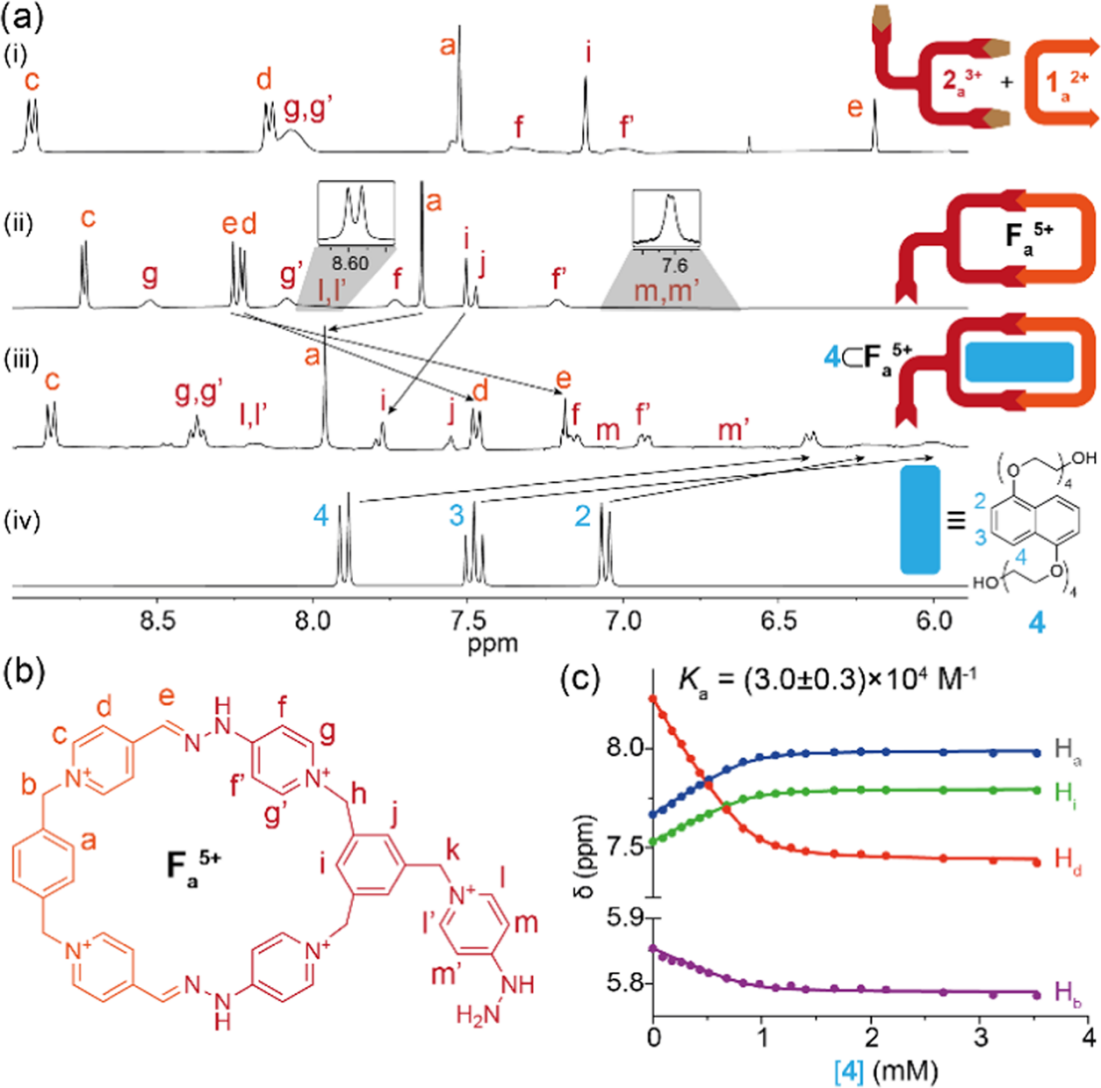
(a) Partial stacked ^1^H NMR (D_2_O, r.t., 500 MHz) of (i) a 2.5 mM 1:1 mixture of **1**_**a**_^2+^ and **2**_**a**_^3+^, (ii) isolated **F**_**a**_^5+^, inset: partial ^1^H NMR spectra at 328.15 K, showing H_l,l′_ and H_m,m′_ signals, (iii) 2.5 mM **4**⊂**F**_**a**_^5+^ at pD 5, and (iv) diol **4**. (b) Structure depiction of **F**_**a**_^5+^. (c) Fitting of the observed variation in the chemical shifts of the protons H_a_, H_i_, H_d_, and H_b_ against the total guest concentration at pD 5.

As shown in Figure 2a, ^1^H NMR in D_2_O of the species shows not only the characteristic resonance for the iminic hydrogen H_e_ (δ = 8.30 ppm) but also the restricted rotation around the (Py^+^)C–NHN bonds, which is characteristic for this type of compound.^12^ The nonequivalence of the nuclei, on the upper and lower sides of the corresponding pyridinium rings, was corroborated by EXSY exchange cross-peaks on a NOESY spectrum and by VT ^1^H NMR experiments (Figures S11 and S15, respectively). The latter showed the transition of the signals from slow to fast exchange regime on the timescale of the technique, allowing in turn to estimate a Δ*G*_rot_^‡^ = 15.2 kcal/mol for the restricted rotations using the coalescence method.^17^ Further evidence of the identity of **F**_**a**_^5+^ was obtained by HR-ESI-MS, which showed typical peaks associated with the deprotonation of the macrocycles in the gas phase (Figure 1, HR-ESI-MS for **F**_**a**_^5+^: *m*/*z* [M – 3H]^2+^ calculated: 361.6768; found: 361.6766 and [M – 2H]^3+^ calculated: 241.4536; found: 241.4535).

Despite the acceptable yield of the pure cyclophane, the reaction conditions also produced the benzylic alcohol **A**_a_^4+^ as a decomposition product (Figure 1), which was isolated in 8% yield from the HPLC purification and fully characterized (Figures S28–S36). Reductions in reaction time or heating temperature did not significantly alter the outcome of the process.^16,17^ Interestingly, none of the conditions tested for the condensation of **1**_**a**_·2Br and **2**_**a**_·3Br produced the otherwise typical [2 + 3] capsule-type species,^18^ even when the stoichiometry of the reaction partners was appropriately modified to favor those [3(**1**_**a**_·2Br) + 2(**2**_**a**_·3Br)]. In this case, the HPLC purification allowed the isolation of a reduced amount of the macrocycle **F**_**a**_·5TFA (19%), a similar amount of the hydrolysis product **A**_**a**_·4TFA (8%), and a new reaction byproduct with spectroscopic data in good agreement with the dimerization product **D**·12TFA (19%), in which two **F**_**a**_^5+^ are connected through the unreacted hydrazine with a **1**_**a**_^2+^ moiety as the bridge.^17^

Encouraged by the acceptable results on synthesizing the asymmetric cyclophane **F**_**a**_^5+^, we also tested the [1 + 1] condensation between the bishydrazine **1**_**b**_·2Br and the trisaldehyde **2**_**b**_·3Br, using the same synthetic conditions discussed above. After 24 h of reaction and subsequent purification by HPLC, we could isolate the corresponding aldehyde-attached cyclophane **F**_**b**_·5TFA in an acceptable 32% yield, accompanied by a significant amount of the benzylic alcohol **A**_**b**_·4TFA (15%).^17^ 1D/2D NMR data in D_2_O allowed us to characterize **F**_**b**_^5+^ cyclophane, showing the new characteristic signal of the hydrazone at 8.22 ppm as well as the same restricted rotation around the (Py^+^)C–NHN bonds displayed for the **F**_**a**_^5+^ macrocycle (Figures S17–S23). Moreover, a ΔG_rot_^‡^ = 14.4 kcal/mol was obtained by a VT-NMR experiment for this restricted rotation (Figure S27), comparable to that of its counterpart. Finally, the characterization of **F**_**a**_^5+^ was completed by HR-ESI-MS, which also showed the peaks corresponding to the deprotonated macrocycle (Figure S25).

**Figure 3:**
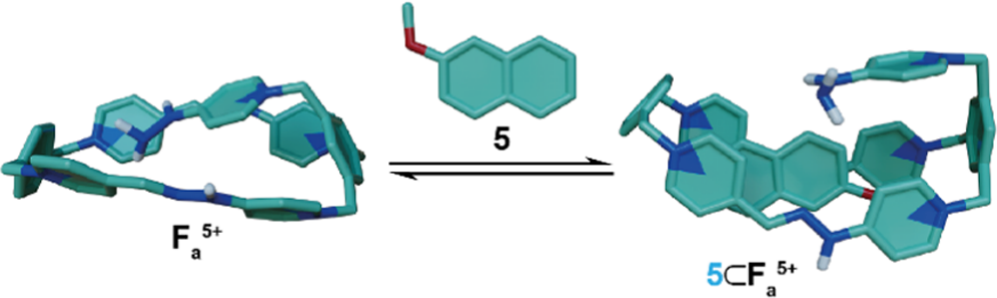
Representative structures minimized at the r^2^scan3c^21^/SMD^22^ (water) level for the model complexation process **F**_**a**_^5+^ + **5** ⇋ **5**⊂**F**_**a**_^5+^. Color code: carbon, green; nitrogen, blue; oxygen, red; hydrogen, white (some hydrogens were removed for clarity).

Following our planned synthesis of **S**^5+^, we proceeded to test the ability of **F**_**a**_^**5+**^ as a molecular receptor in water. First, the complexation process was studied *in silico* using as the potential guest 2-methoxynaphthalene (**5**, Figure 3), a truncated and less computationally demanding version of the aldehyde **3**. Using the multilevel modeling workflow CREST/CENSO developed by Grimme et al., which considers structural ensembles of conformers/complexes rather than individual structures,^17,19,20^ a value of Δ*G*_rot_^‡^ = −7.4 kcal/mol (*K*_a_ = 2.2 × 10^5^ M^–1^) was determined for the process in water, corroborating **F**_**a**_^5+^ as an appropriate receptor for electron-rich aromatics. Representative structures for each of the species at the r^2^scan-3c^21^/SMD^22^ (water) level of theory are represented in Figure 3, which, for **5**⊂**F**_**a**_^5+^, shows a longitudinal insertion mode for the guest within the complex and also establishes a stronger interaction with the pyridinium rings on the wider side of the isosceles trapezoidal cavity of **F**_**a**_^5+^.

The complexation ability of **F**_**a**_^5+^ was also tested experimentally, using in this occasion the naphthalene derivative **4** as an appropriate water-soluble and pH-insensitive model substrate. As shown in Figure 2a, the ^1^H NMR spectrum of a 2.5 mM 1:1 mixture of the host–guest pair in D_2_O displayed complexation-induced shifts in good agreement with the formation of the supramolecule. Hence, substrate signals are shielded due to the inclusion of the guest inside the hydrophobic π-deficient cavity of the receptor, with strong C–H···π interactions observed between the protons H_3,4_ in **4** and the phenyl rings of the macrocycle, which, in turn, appear strongly shielded. In addition, the shielding of the H_d,e_ signals of the macrocycle is due to the π–π interaction with the aromatic substrate, as observed with analogous macrocycles.^12b,12e^ DOSY experiments of the mixture showed a unique diffusion coefficient for the host–guest pair (Figure S72), corroborating the formation of the complex. On the other hand, as expected, the signals of the pyridinium pendant are only slightly altered as a result of the complexation. Association constants for the complexation of **4**, both with **F**_**a**_^5+^ and its conjugate base **F**_**a**_^3+^, could be calculated by ^1^H NMR titrations in buffered aqueous solution at pD = 5 and 11, respectively.^23^ Essentially, receptors **F**_**a**_^5+^/**F**_**a**_^3+^ demonstrated similar complexation abilities for the naphthalene substrate, with *K*_a_ values in the 10^4^ M^–1^ range (Figure 2c at pD = 5 and Figure S75 at pD = 11), in good agreement with those previously computed and other analogous systems.^12^ Similar features, as those described above for **F**_**a**_^5+^/**F**_**a**_^3+^, were also found for the aldehyde-appended cyclophane receptors **F**_**b**_^5+^/**F**_**b**_^3+^.^17^ As clear evidence of the importance of the hydrophobic effect in this type of complex, neither **F**_**a/b**_^5+^ nor the conjugate base **F**_**a/b**_^3+^ was found able to complex the model substrate **4** in CD_3_CN.

### Self-Assembly of the Pseudo[1]Rotaxane **S^5+^**

**Figure 4:**
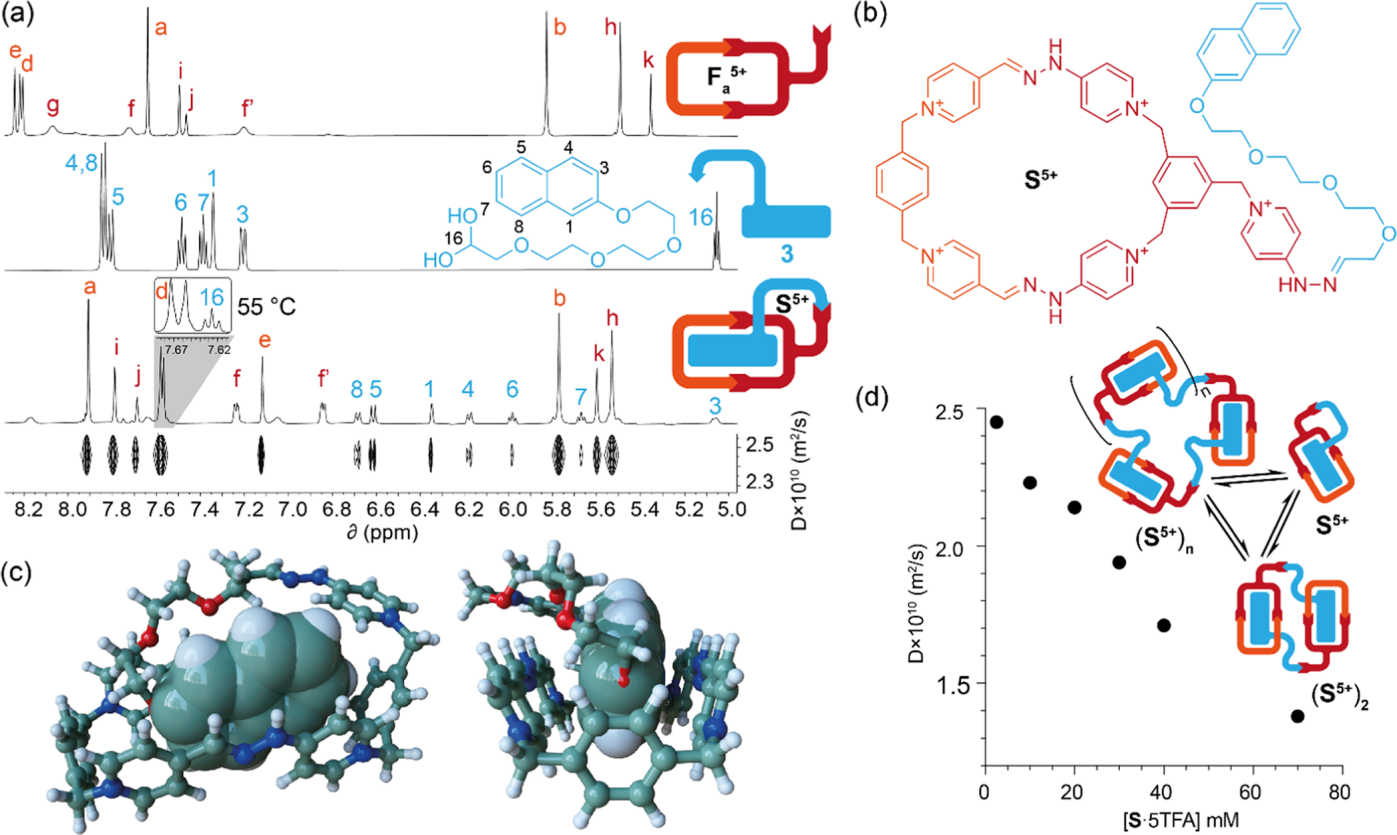
(a) Partially stacked ^1^H NMR (D_2_O, 500 MHz) spectra of (i) **F**_**a**_·5TFA (proton labeling as in Figure 2b), (ii) **3**, (iii) isolated **S**·5TFA at 2.5 mM, inset: partial ^1^H NMR spectrum at 328.15 K showing the H_16_ signal, (iv) DOSY (D_2_O, 500 MHz) experiment of 2.5 mM **S**·5TFA. (b) Structure depiction of **S**^5+^. (c) Stick and ball (macrocyclic part and poly(ethylene glycol) chain) and van der Waals (naphthalene moiety) representation of the minimized structure of **S**^5+^. Color code as in Figure 3. (d) Graphical representation of diffusion coefficient versus **S**·5TFA concentration.

Once the outcome of the self-assembly of **1**_**a**_^2+^ and **2**_**a**_^3+^ to the cyclophane **F**_**a**_^5+^ was firmly established, as well as its ability to complex aromatic molecules in water, we proceeded to explore the self-assembly of our target molecule **S**^5+^. First, we analyzed the outcome of the reaction of the isolated **F**_**a**_^5+^ and the aldehyde **3** under acidic aqueous conditions. To this end, **F**_**a**_·5TFA (2.5 mM) and an excess of **3** (1.5 equiv) were mixed in water with 10% molar TFA as the catalyst, and the mixture was heated at 60 °C in a round-bottom flask with a condenser. After 24 h, the consumption of **F**_**a**_^5+^ and the formation of a new major species were observed by HPLC-MS (Figure S92). The reaction crude was worked up and purified by semipreparative HPLC, resulting in the isolation of a compound (54% yield) with spectroscopic data in good agreement with the target pseudo[1]rotaxane **S**·5TFA. Crucially, the NMR and UV–vis data obtained for the compound were found to be concentration-independent below 2.5 mM (Figures S103 and S104). However, upon increasing the concentration, a broadening effect of all ^1^H NMR signals is observed in addition to a significant change in the chemical shifts of the tetraethylene glycol signals (Figure S104). As shown in Figure 4d, a clear decrease from 2.45 × 10^–10^ to 1.38 × 10^–10^ m^2^/s of the diffusion coefficient is observed from the DOSY experiments in the 2.5–70 mM range (Figures S105–S109), confirming that the compound behaves, as expected, as a typical supramolecular aggregate.^24^

Once the aggregation of the compound was qualitatively assessed, we proceeded to characterize the monomeric self-inclusion complex at 2.5 mM by 1D/2D NMR experiments. In this regard, the signals for the pseudo[1]rotaxane species compared to **F**_**a**_^5+^ show similar key features to those discussed for the inclusion complex **4**⊂**F**_**a**_^5+^. In essence, the new hydrazone bond formed between the aldehyde **3** and the unreacted hydrazine pendant is seen as a clear signal in the spectrum for the imine proton H_16_, which overlaps with H_d_ at r.t. but is clearly observed as a triplet at 7.63 ppm in the spectrum recorded at 328.15 K (Figure 4a inset). The NMR data also allowed us to establish a tentative longitudinal insertion mode similar to that of **4**⊂**F**_**a**_^5+^ for the naphthalene moiety within the cavity of **S**^5+^, as derived from the strong C–H···π interactions observed for H_3,6,7_ with the short walls of the receptor, which is also reflected in the pronounced deshielding of hydrogens H_a,i,j_ of the annulus. Nevertheless, due to the fast exchange regime observed on the NMR timescale for all of the nuclei, no further structural information about the potential conformation of the pseudo[1]rotaxane could be obtained from the NMR data (i.e., the relative disposition of the asymmetric guest part with respect to the plane of the macrocycle).^13^ Further evidence of the formation of the target species could be obtained by the corresponding DOSY experiment of **S**^5+^ at 2.5 mM concentration, where the experimental diffusion coefficient of **S**^5+^ (2.45 × 10^–10^ m^2^/s) is comparable to that obtained theoretically from the dimensions of a local minimum found for the pseudo[1]rotaxane (Figure 4c, 2.32 × 10^–10^ m^2^/s).^17^ Likewise, HR-ESI-MS of the obtained compound also corroborated the formation of **S**^5+^, with *m*/*z* calculated for C_62_H_62_N_11_O_4_^3+^ [M – 2H]^3+^ 341.4990, found: 341.4989.

**Figure 5:**
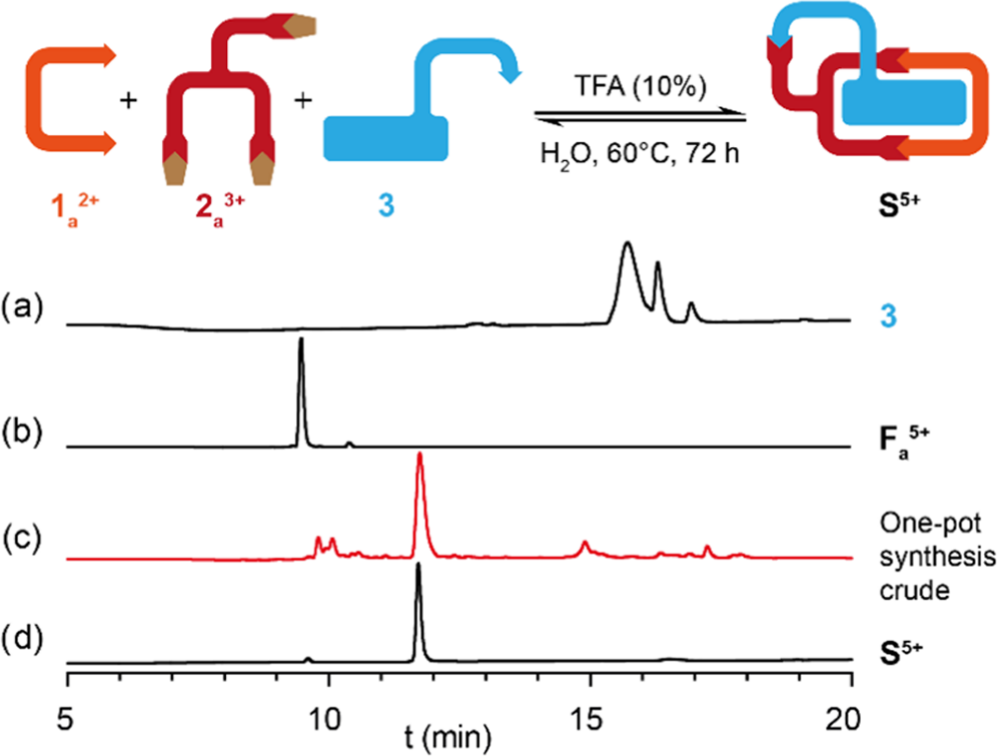
Stacked analytical HPLC chromatograms at 220 nm of (a) the aldehyde **3**,^25^ (b) isolated **F**_**a**_^5+^, (c) the reaction crude from the one-pot synthesis of **S**^5+^, and (d) isolated **S**^5+^.^26^

As a final step of our synthetic journey, we proceeded to test the one-pot three-component synthesis of **S**^5+^, monitoring by ^1^H NMR and analytical HPLC the reaction of a 2.5 mM equimolar mixture of **1**_**a**_·2Br and **2**_**a**_·3Br with an excess of **3** (1.5 equiv), in our standard condensation conditions.^12,17^ Pleasingly, after 72 h, the reaction was found to proceed as expected, producing the pseudo[1]rotaxane **S**^5+^ as the major species in the reaction crude through an integrative social self-sorting process (Figure 5),^10^ which could be isolated pure in 42% yield by semipreparative HPLC.

**Figure 6:**
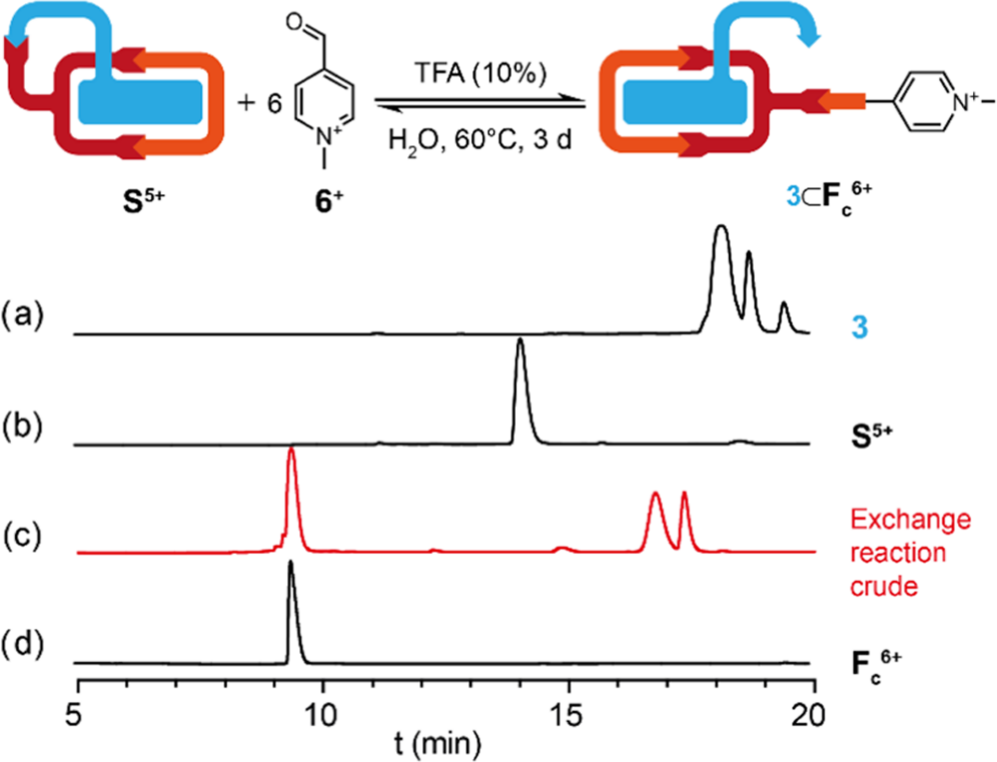
Schematic representation of the exchange reaction between **S**^5+^ and **6**^**+**^. Stacked analytical HPLC chromatograms at 220 nm of (a) the aldehyde **3**,^25^ (b) isolated **S**^5+^, (c) exchange reaction crude showing traces of **F**_**c**_^6+^ and **3**, and (d) isolated **F**_**c**_^6+^.^26^

To shed some light on the different kinetic labilities and thermodynamic stabilities observed for the two hydrazone bonds involved in the synthesis of **S**^5+^, we decided to carry out a competition study by reacting the less electrophilic aldehyde **6**^+^ with **S**^5+^ and monitoring the reaction by ^1^H NMR and analytical HPLC. For this purpose, we added an excess of **6**·I (6 equiv) to a 40 mM aqueous solution of **S**·5TFA under acidic conditions (10% TFA) and heated the mixture at 60 °C. After 3 days, a complete exchange between **6**^+^ and the linker **3** was observed, keeping the macrocyclic part intact and giving rise to the new *exo*-functionalized macrocycle **F**_**c**_^6+^.^27^ As shown in Figure 6, the HPLC chromatogram of the reaction crude shows only the trace of **F**_**c**_^6+^ and the free linker **3**, confirming the ability to transform from one species to another by exploiting the different hydrazone bond labilities of the *exo*-functionalized macrocycle under the synthetic conditions tested. Likewise, the ^1^H NMR spectrum of the crude recorded in D_2_O shows identical results but with the linker **3** forming the **3**⊂**F**_**c**_^6+^ inclusion complex (Figure S114), with the typical complexation-induced shifts of the aromatic signals from the substrate **3** as from the macrocycle. Moreover, the DOSY experiment of the crude not only showed that the inclusion complex is indeed formed (Figure S115) but also that the potential oligomers formed at 40 mM from **S**^5+^ are broken after the exchange, going from a diffusion of 1.7 10^–10^ m^2^/s for **S**^5+^ to 2.0 10^–10^ m^2^/s for **3**⊂**F**_**c**_^6+^.

## Conclusions

In summary, in this work, we have reported the self-assembly in water of a new polycationic pseudo[1]rotaxane **S**^5+^ through hydrazone bonds with different labilities and using three complementary components. The process was first tested sequentially, allowing us to obtain the *exo*-functionalized macrocyclic receptors **F**_**a,b**_^5+^ in good yields. In turn, **F**_**a**_^5+^ was smoothly converted to the target **S**^5+^ by reaction with the aliphatic aldehyde **3**. On the other hand, the three-component one-pot synthesis of **S**^5+^ was also achieved through an integrative social self-sorting process that ends with the pseudo[1]rotaxane as the species that maximizes the number of host–guest interactions per self-assembled unit. As expected, **S**^5+^ showed the typical dynamic behavior of a donor–acceptor moiety, with NMR experiments confirming how increasing the concentration of **S**^5+^ induces the discrete self-inclusion complex to reorganize into daisy chain oligomers. Finally, we have demonstrated the ability of **S**^5+^ to morph into other functionalized **F**_**a**_^5+^ derivatives by exchanging the aliphatic aldehyde pendant for an aromatic aldehyde, which produces a more stable imine bond. These results not only prove the utility of the imine bond for the aqueous self-assembly of macrocyclic derivatives of low symmetry but also open the door for the use of the obtained *exo*-functionalized cyclophanes for the nontrivial implementation of macrocyclic hosts into more complex materials.^28^

## Data Availability

The data underlying this study are available in the published article and its Supporting Information.

## Supporting Information Available

Experimental details; synthetic procedures and characterization data for new compounds; titration data for the determination of the supramolecular association constants *K*_a_; aggregation studies of **S**^**5+**^ in solution; computational details; and Cartesian coordinates for the different energy minima discussed in the manuscript and additional figures (PDF)
