# Supplementary material for: Aqueous Three-Component Self-Assembly of a Pseudo[1]rotaxane Using Hydrazone Bonds
Source: J Org Chem. 2023 Apr 28;88(11):6784–90. doi: 10.1021/acs.joc.3c00108 (PMC10731646; doi:10.1021/acs.joc.3c00108)
Supplement: Supplementary file 1 — jo3c00108_si_001.pdf [file jo3c00108_si_001.pdf]

Supporting Information

Aqueous Three-component Self-assembly of a Pseudo[1]rotaxane using Hydrazone Bonds

Pablo Cortón, Natalia Fernández-Labandeira, Mauro Díaz-Abellás, Carlos Peinador, Elena Pazos, Arturo Blanco-Gómez,* Marcos D. García*

CICA – Centro Interdisciplinar de Química e Bioloxía and Departamento de Química, Facultad de Ciencias. Universidade da Coruña, 15071, A Coruña, Spain.

Corresponding Author's Email: arturo.blanco.gomez@udc.es; marcos.garcia1@udc.es

Index

1. Materials and general procedures. S2
2. Experimental Section. S3
2.1. Synthesis and characterization of F_a^{5+}. S3
2.2. Determination of the energy of the rotational barrier ($\Delta G^{\neq}$) for F_a^{5+}. S12
2.3. Synthesis and characterization of F_b^{5+}. S13
2.4. Determination of the energy of the rotational barrier ($\Delta G^{\neq}$) for F_b^{5+}. S20
2.5. Macrocyclization side products. S21
2.5.1. Characterization of A_a^{4+}. S21
2.5.2. Characterization of A_b^{4+}. S26
2.5.3. Characterization of D^{12+}. S31
2.6. General synthesis and NMR characterization of the inclusion complexes. S37
2.6.1. NMR characterization of $4\subset F_a^{5+}$. S37
2.6.2. NMR characterization of $4\subset F_b^{5+}$. S42
2.7. ^{1}H NMR titration experiments: determination of K_a values. S47
2.7.1. $4\subset F_a^{5+}$ at pD = 5. ....S47
2.7.2. $4\subset F_a^{3+}$ at pD = 11. ....S48
2.7.3. $4\subset F_b^{5+}$ at pD = 5. ....S49
2.7.4. $4\subset F_b^{3+}$ at pD = 11. ....S50
2.8. Synthesis of 2-(2-(2-(2-(naphthalen-2-yloxy)ethoxy)ethoxy)ethoxy) acetaldehyde (Compound 3). S52
2.9. Synthesis of the pseudo[1]rotaxane S^{5+}. S61
2.10. Aggregation studies of S^{5+}. S68
2.10.1. UV-Vis at low concentrations. S68
2.10.2. ^{1}H-NMR and DOSY experiments at high concentration. S68
2.11. Determination of diffusion coefficient from theoretical model. S73
2.12. One-pot synthesis of S^{5+}. S74
2.13. Hydrazone metathesis of S^{5+}. S76
2.14. Characterization of F_c^{6+}. S80
2.15. Computational details. S86
2.15.1. Computation of Gibb's free energy for $F_a^{5+} + 5 \leftrightharpoons 5\subset F_a^{5+}$ S86
2.15.2. Local minimum for pseudo[1]rotaxane S^{5+}. S87
2.15.3. Cartesian coordinates for representative structures S87

1. Materials and general procedures.

All reagents and solvents were purchased from commercial sources and used without further purification. Compounds $\mathbf{1}_{\mathbf{a}}^{2+}$,[1] $\mathbf{1}_{\mathbf{b}}^{2+}$,[1] $\mathbf{2}_{\mathbf{a}}^{3+}$ [2] and $\mathbf{2}_{\mathbf{b}}^{3+}$ [2] were synthesized according to the literature. Manipulations were performed under normal atmosphere unless special noted. Nuclear magnetic resonance (NMR) spectra were recorded at ambient temperature using Bruker AVANCE III 300/400/500 spectrometers, with working frequencies of 300/400/500 and 75/100/125 MHz for ^{1}H and ^{13}C, respectively. Chemical shifts are reported in ppm relative to the residual internal non deuterated solvent signals (D_2O: δ = 4.79 ppm, CD_3CN: δ = 1.94 ppm). Structural assignments were made with additional information from gCOSY, gHSQC, and gHMBC experiments. High-resolution mass spectra (HRMS) were recorded on a LTC-Orbitrap Discovery mass spectrometer. HPLC purifications were performed on an Agilent 1260 Infinity II with a semipreparative column Luna® Omega 5 µm Polar C18 100 Å from Phenomenex. HPLC-MS analysis was performed on a Thermo Scientific UltiMate 3000 connected to a photo-diode array (PDA) detector and a single quadrupole mass spectrometer Thermo Scientific MSQ, and a *Bruker* Elute UHPLC connected to a mass spectrometer Bruker amaZon speed Toxtyper or a high-resolution TIMS-QTOF *Bruker Daltonics* timsTOF Pro with a *Bruker Daltonics* CaptiveSpray ion source using an Aeris analytical column from *Phenomenex* (peptide XB-C18 stationary phase, 3.6 µm, 100 Å pore size, 150 × 2.1 mm). The standard method used for analytical HPLC was 5 → 95% MeCN, 0.04% TFA / H_2O, 0.04% TFA over 23 min. UV/vis spectra were recorded on a Jasco V-650 spectrometer.

---

[1] A. Blanco-Gómez, I. Neira, J. L. Barriada, M. Melle-Franco, C. Peinador and M. D. García, *Chem. Sci.*, 2019, **10**, 10680-10686.
[2] P. Cortón, H. Wang, I. Neira, A. Blanco-Gómez, E. Pazos, C. Peinador, H. Li and M. D. García, *Org. Chem. Front.*, 2022, **9**, 81-87.

2. Experimental Section.

2.1. Synthesis and characterization of F_a^{5+}.

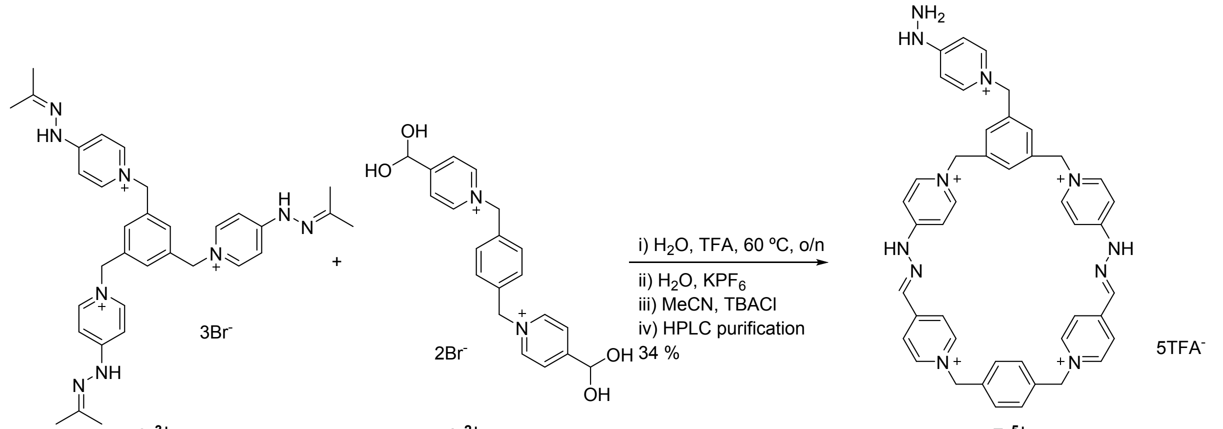

$\mathbf{2_a}^{3+}$ $\mathbf{1_a}^{2+}$ $\mathbf{F_a}^{5+}$

A solution of $\mathbf{1_a}^{2+}$ (64.0 mg, 0.125 mmol), $\mathbf{2_a}^{3+}$ (101.0 mg, 0.125 mmol) and TFA (10% molar) in 50 mL of water was heated at 60 ºC overnight using a heat-on block. After cooling, excess of KPF_6 was added until no further precipitation was observed. The obtained solid was filtered and washed with water (5 × 5 mL) and ether (5 x 5 mL), yielding a reddish powder. The solid was redissolved in MeCN and an excess of TBACl was added until no more precipitate was formed. The solid was washed with MeCN (5 x 5 mL) and Et_2O (5 x 5 mL). A fraction of the solid (51.1 mg) was dissolved in H_2O + TFA 10%mol, and heated at 60 ºC for 6 h using a heat-on block. The solution was lyophilized, redissolved in H_2O + TFA (100 μL) filtered and purified by reverse-phase semipreparative HPLC (A: H_2O + 0.1% TFA, B: MeCN + 0.1% TFA), giving a yellowish solid (21.8 mg, 34%, $\mathbf{F_a}^{5+}$).

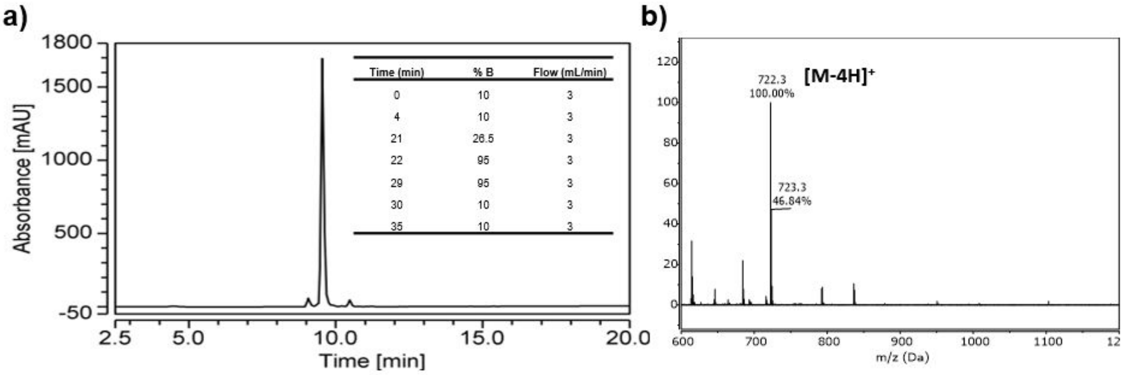

Figure S1: a) HPLC chromatogram (220 nm) of purified F_a^{5+} at t_R = 9.5 min (Inset: purification method). b) MS spectrum from the chromatographic peak at t_R = 9.5 min.

The reaction for the synthesis of the macrocycle $\mathbf{F_a}^{5+}$ was performed at an NMR scale under different conditions. The diminishing of the temperature yields to slightly dirtier raw products, which can be assessed by looking at the CH_2 signals around 5-6 ppm. 60 ºC at 2.5 mM with no template remains the best option.

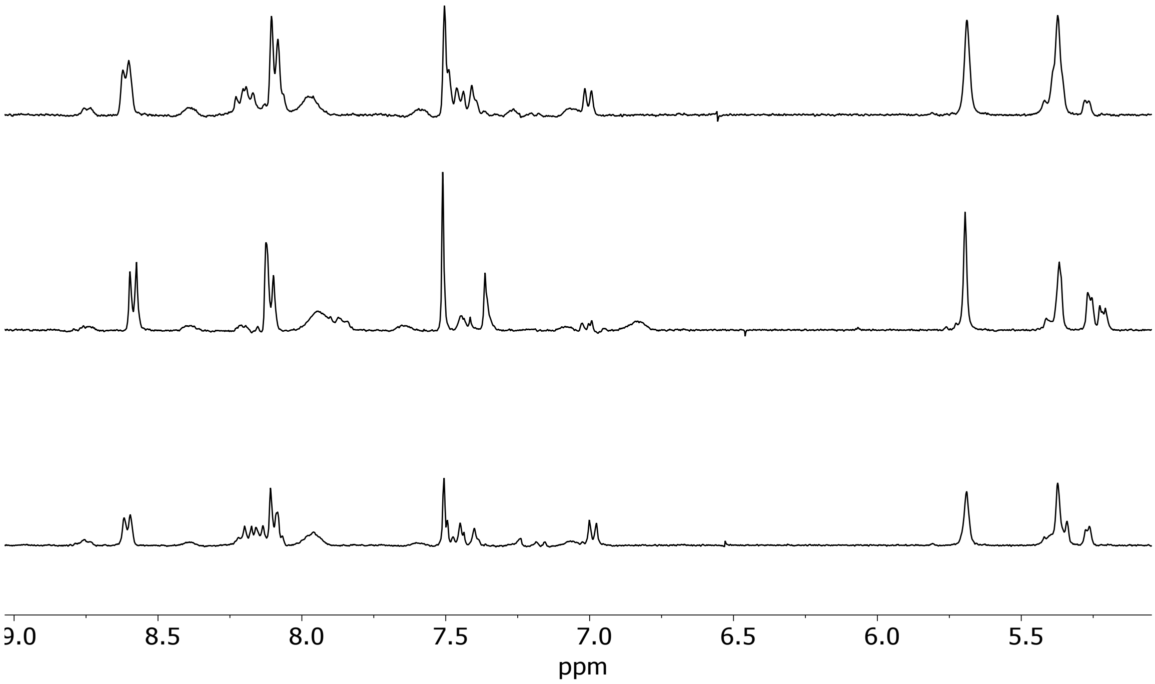

Figure S2: ^{1}H-NMR (300 MHz, D_2O) spectra of the crude products of the condensations of F_a^{5+} at different temperatures: 60 ºC (top), 40 ºC (middle) and rt (bottom).

While maintaining the temperature at 60 ºC, changing the concentration of the monomers from 2.5 mM to 1 mM does not improve the results either, and makes the resulting raw product less clear.

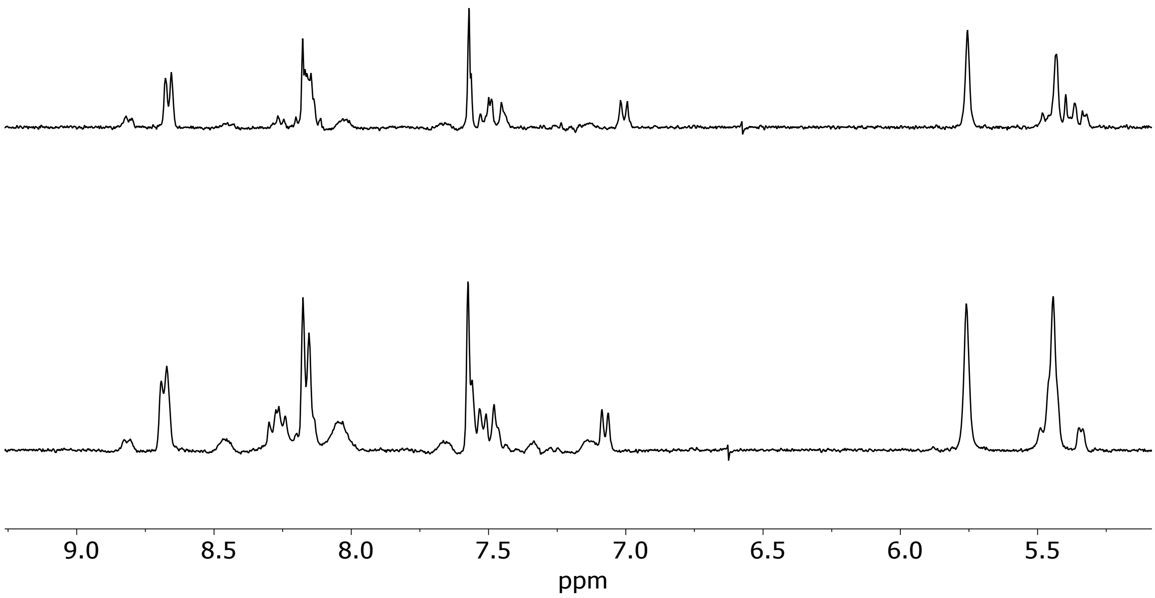

Figure S3: ^{1}H-NMR (300 MHz, D_2O) spectra of the crude products of the condensations of F_a^{5+} at different concentrations: 1 mM (top) and 2.5 mM (bottom).

The addition of a template (1,5-DHN) does not seem to improve the resulting crude product.

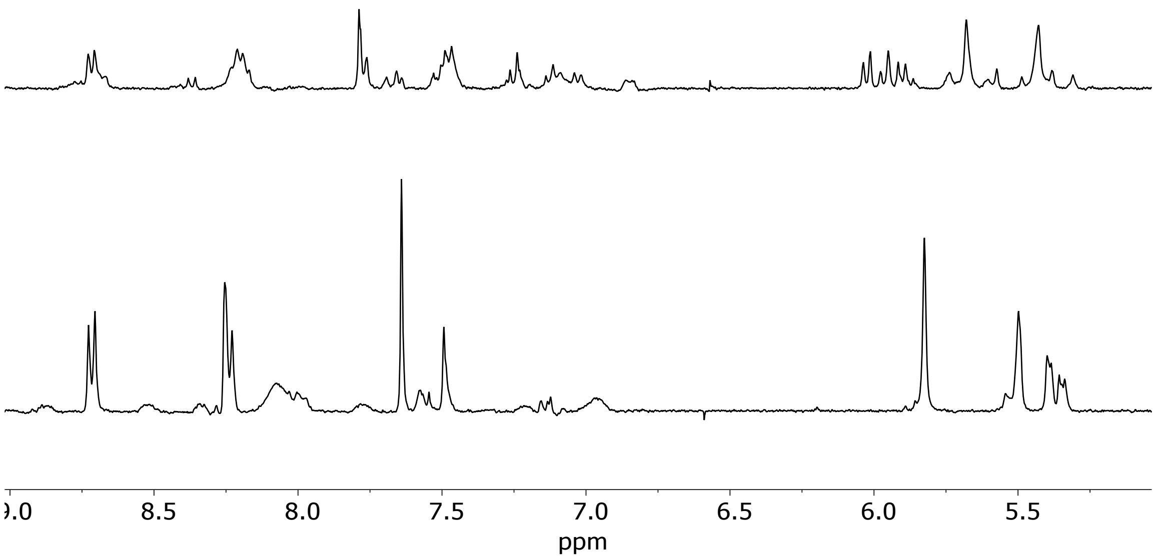

Figure S4: ^{1}H-NMR (300 MHz, D_2O) spectra of the crude products of the condensations of F_a^{5+} with template (1,5-DHN, top) and without template (bottom).

The results indicate that the best reaction conditions (as indicated above) are 60 ºC and a concentration of 2.5 mM, without template.

^{1}H-NMR (D_2O, 500 MHz): δ = 8.73 (d, J = 6.7 Hz, 4H), 8.52 (s, 2H), 8.26 (s, 2H), 8.22 (d, J = 6.5 Hz, 4H), 8.08 (s, 2H), 7.97 (d, J = 7.3 Hz, 2H), 7.73 (s, 2H), 7.65 (s, 4H), 7.50 (s, 2H), 7.47

(s, 1H), 7.21 (s, 2H), 6.83 (d, *J* = 7.3 Hz, 2H), 5.83 (s, 4H), 5.49 (s, 4H), 5.36 (s, 2H). $^{13}C\{^1H\}$-NMR (125 MHz, D_2O): δ = 158.4 (C), 154.6 (C), 149.9 (C), 144.1 (CH), 143.9 (CH), 143.0 (CH), 141.0 (CH), 137.4 (C), 136.1 (C), 134.6 (C), 131.1 (CH), 129.7 (CH), 129.6 (CH), 124.8 (CH), 117.4 (CH), 115.1 (CH), 110.4 (CH), 109.8 (CH), 63.6 (CH_2), 61.1 (CH_2), 59.8 (CH_2). HRMS (ESI): *m/z* calculated for $C_{44}H_{42}N_{11}^{2+}$ [*M*-2H]$^{3+}$ 241.4536, found 241.4535; *m/z* calculated for $C_{44}H_{41}N_{11}^{2+}$ [*M*-3H]$^{2+}$ 361.6768, found 361.6767.

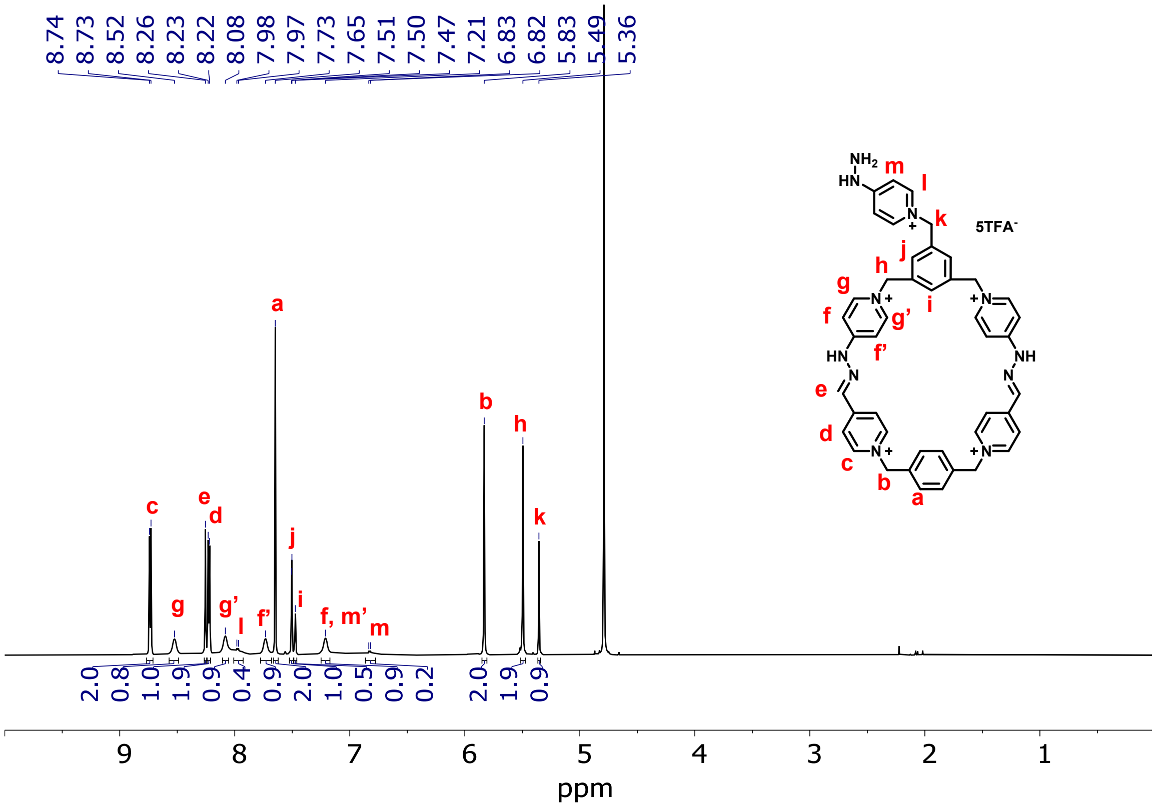

Figure S5: ¹H-NMR (500 MHz, D_2O) spectrum of F_a^{5+}.

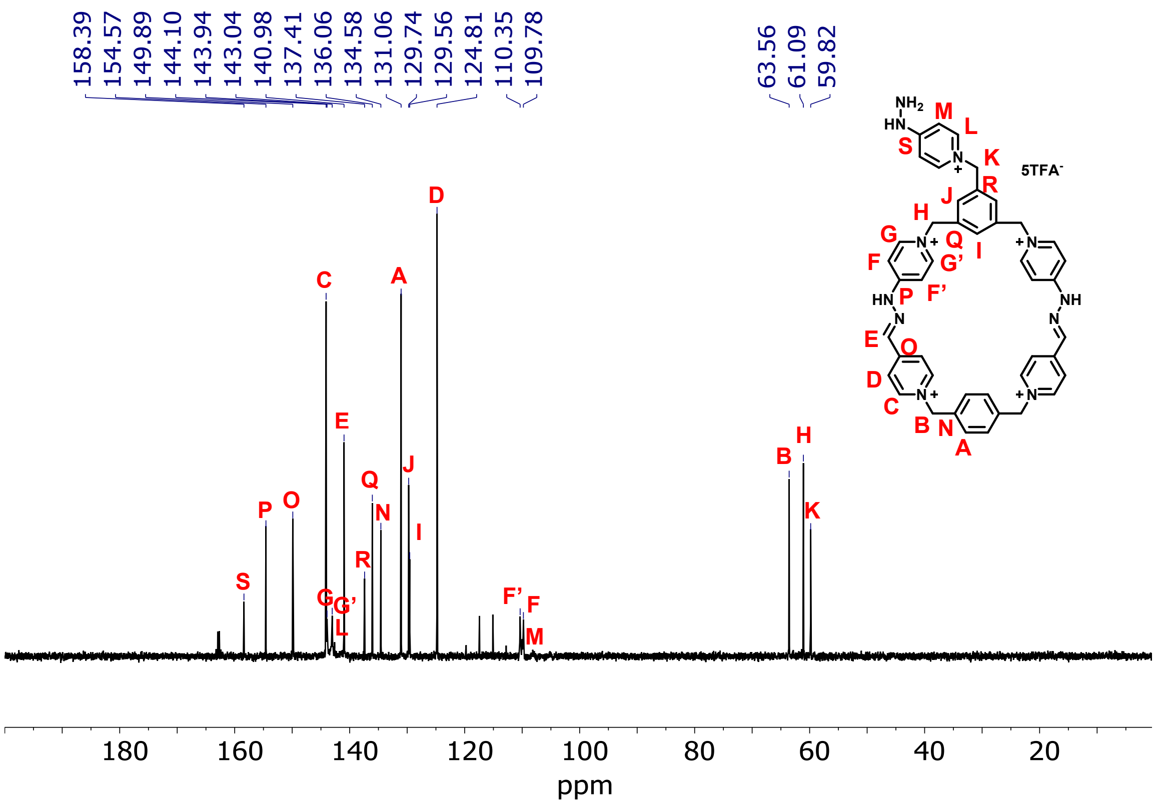

Figure S6: $^{13}C\{^1H\}$-NMR (126 MHz, D_2O) spectrum of F_a^{5+}.

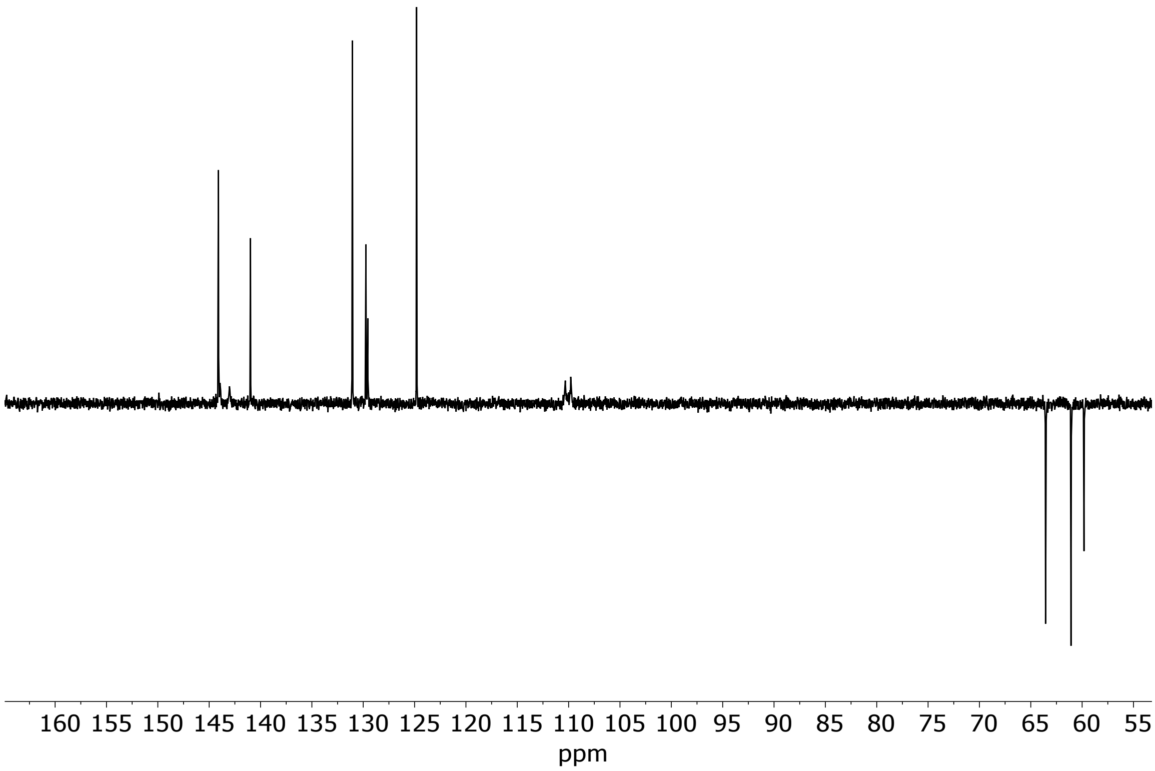

Figure S7: DEPT-135 (126 MHz, D_2O) spectrum of F_a^{5+}.

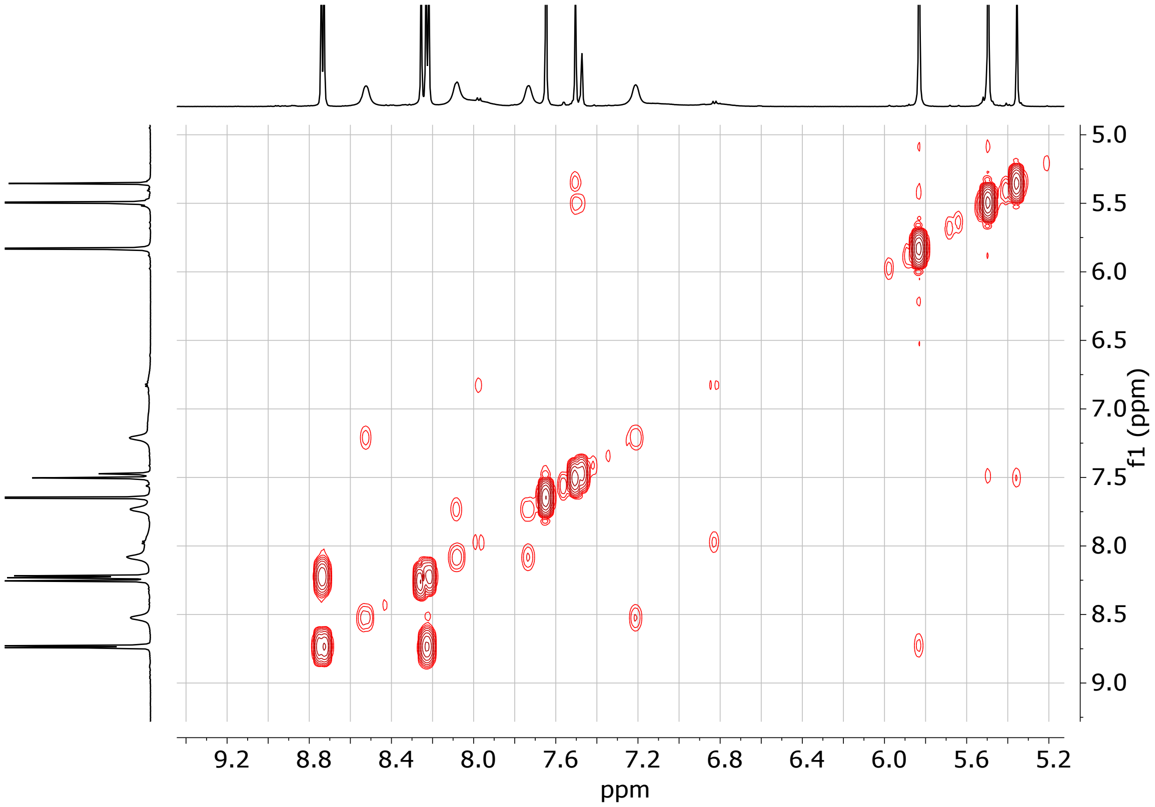

Figure S8: ^1H-^1H COSY (500 MHz, D_2O) spectrum of F_a^{5+}.

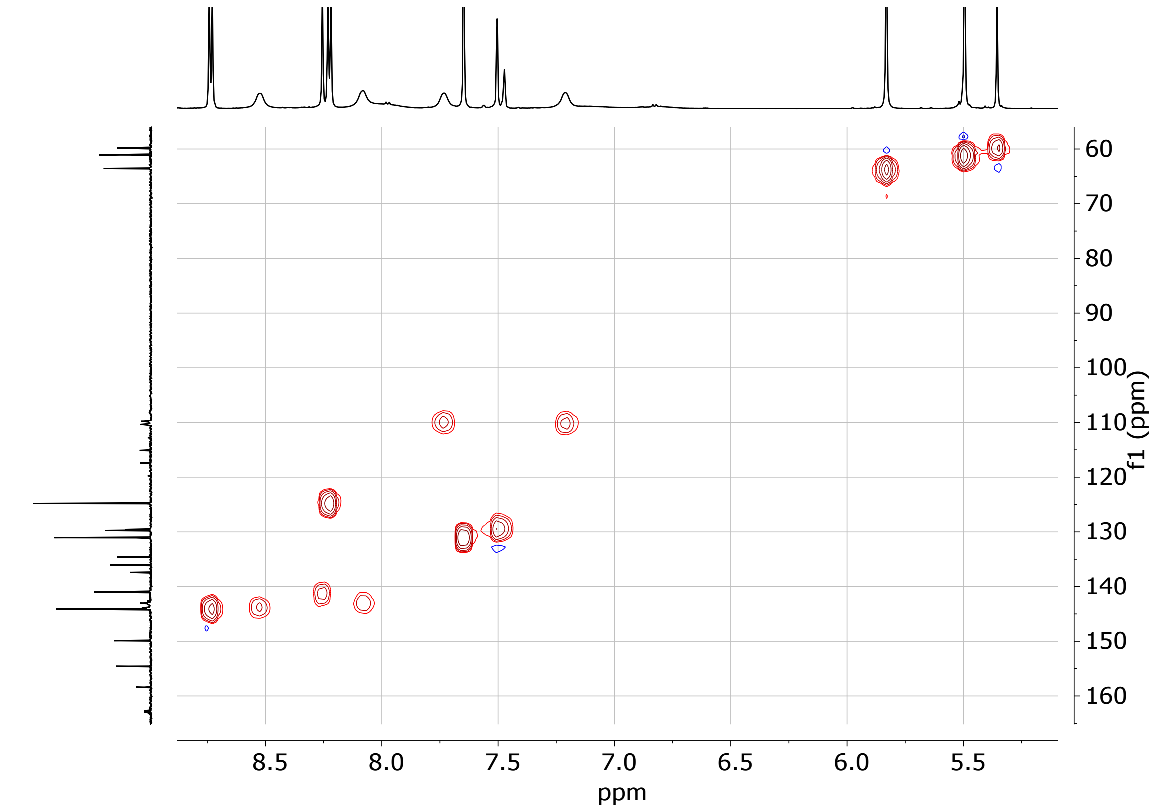

Figure S9: ^{1}H-$^{13}C\{^{1}H\}$ HSQC (500 and 126 MHz, D_2O) spectrum of F_a^{5+}.

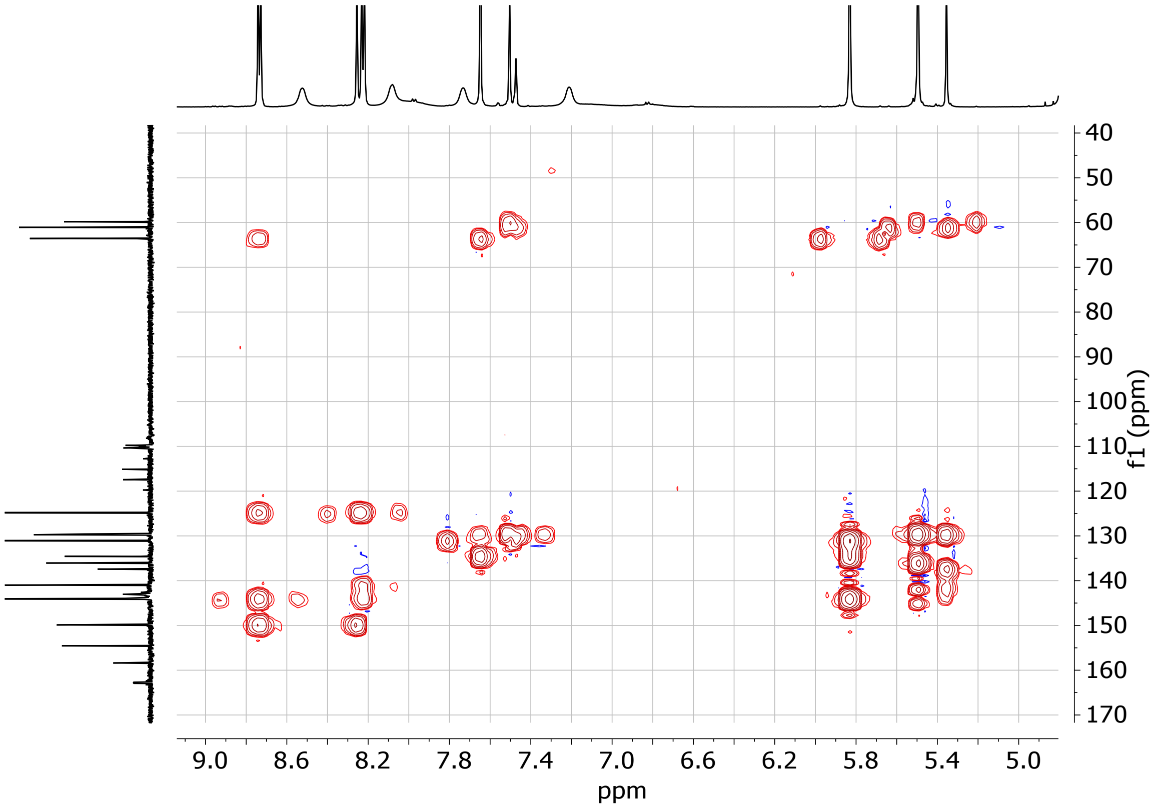

Figure S10: ^{1}H-$^{13}C\{^{1}H\}$ HMBC (500 and 126 MHz, D_2O) spectrum of F_a^{5+}.

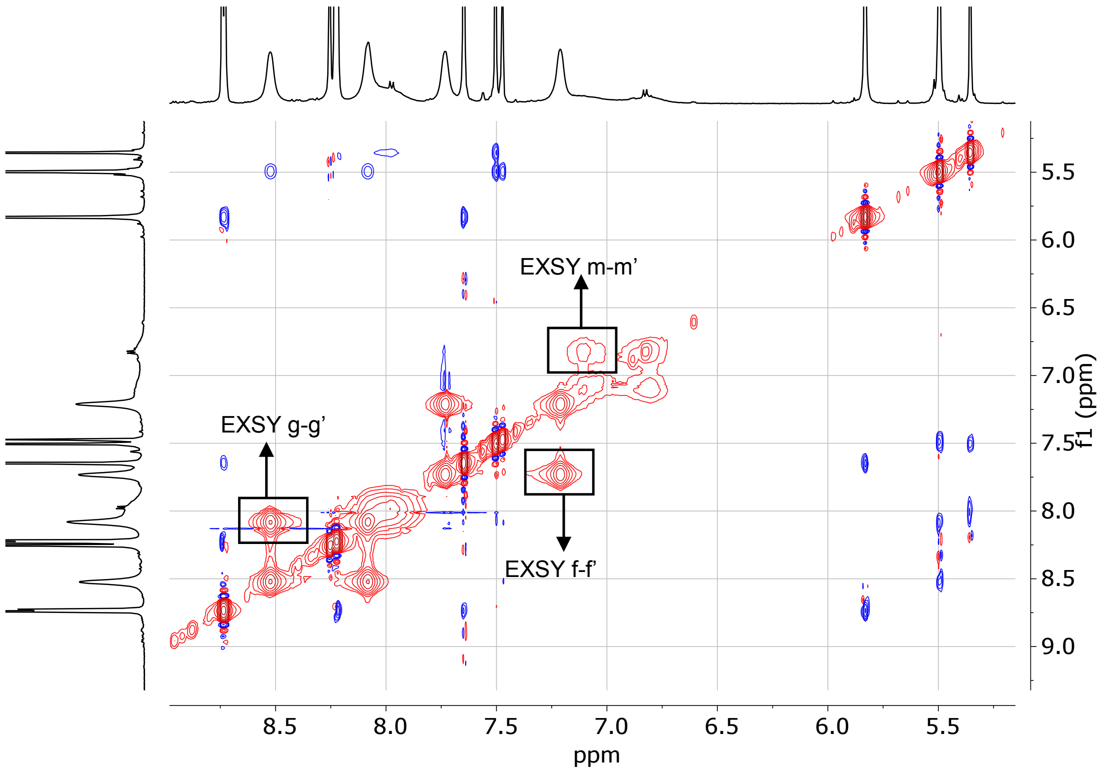

Figure S11: ^{1}H-^{1}H NOESY (500 MHz, D_2O) spectrum of F_a^{5+}.

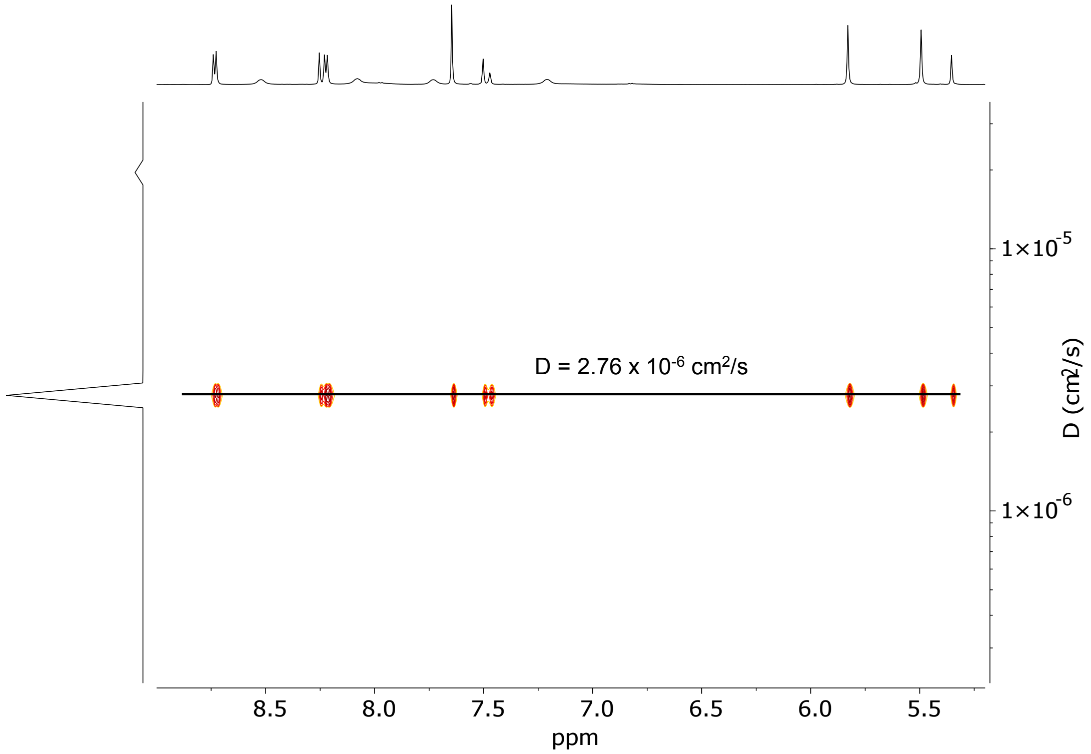

Figure S12: DOSY spectrum of F_a^{5+}.

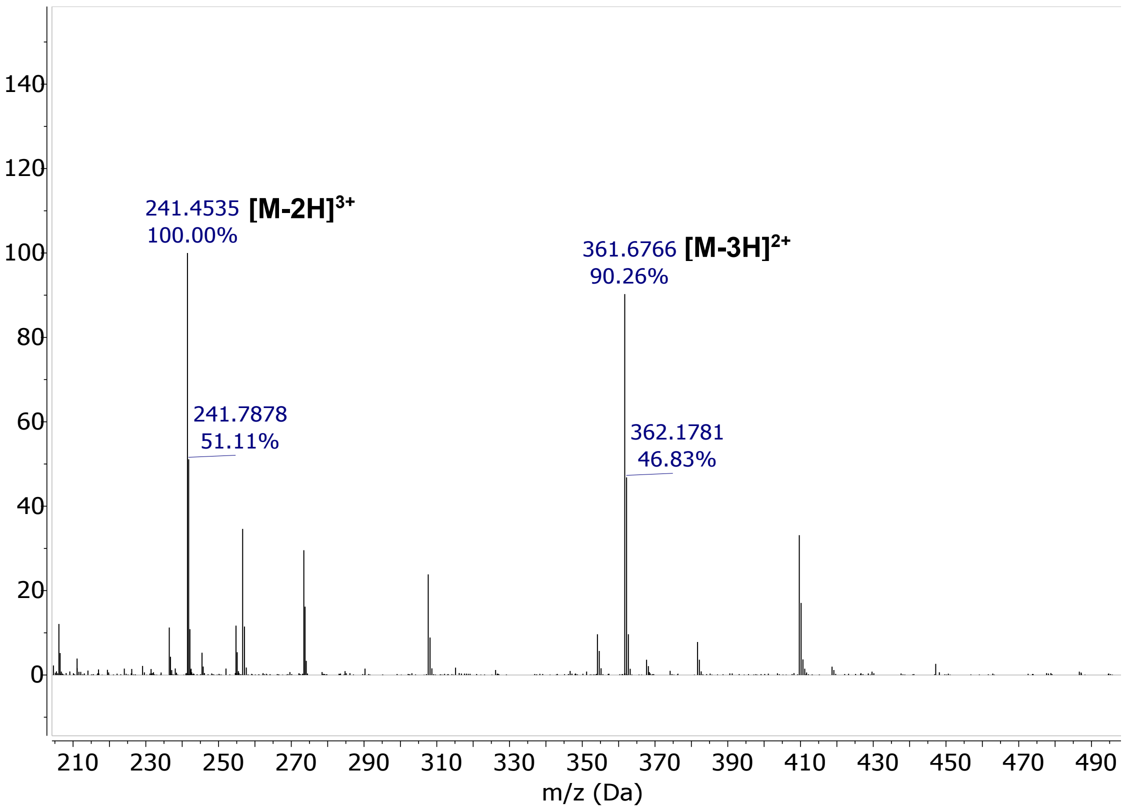

Figure S13: ESI-HRMS of F_a^{5+}.

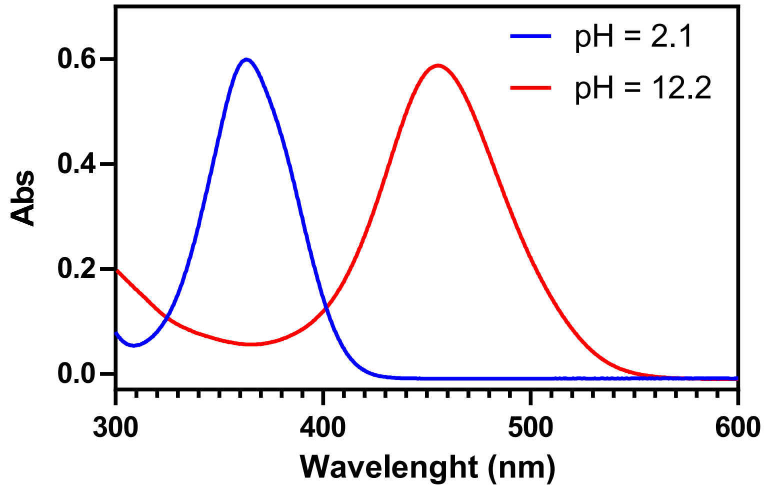

Figure S14: UV-Vis absorption spectra (phosphate buffer 10 mM) of F_a^{5+} at pH = 2 (blue) and 12 (red).

2.2. Determination of the energy of the rotational barrier ($\Delta G^{\neq}$) for F_a^{5+}.

The coalescence temperature (T_c) could be estimated for different protons on VT NMR experiments. This provides, in association with the maximum peak separation ($\Delta\nu$ in Hz) at slow exchange between **f** – **f'** and **g** – **g'** for $\mathbf{F_a}^{5+}$, the energy of the rotational barrier using Equation (1).[3]

$$\Delta G^{\neq} = 4.57 \cdot 10^{-3} T_c (9.972 + \log T_c/\Delta\nu) \quad (1)$$

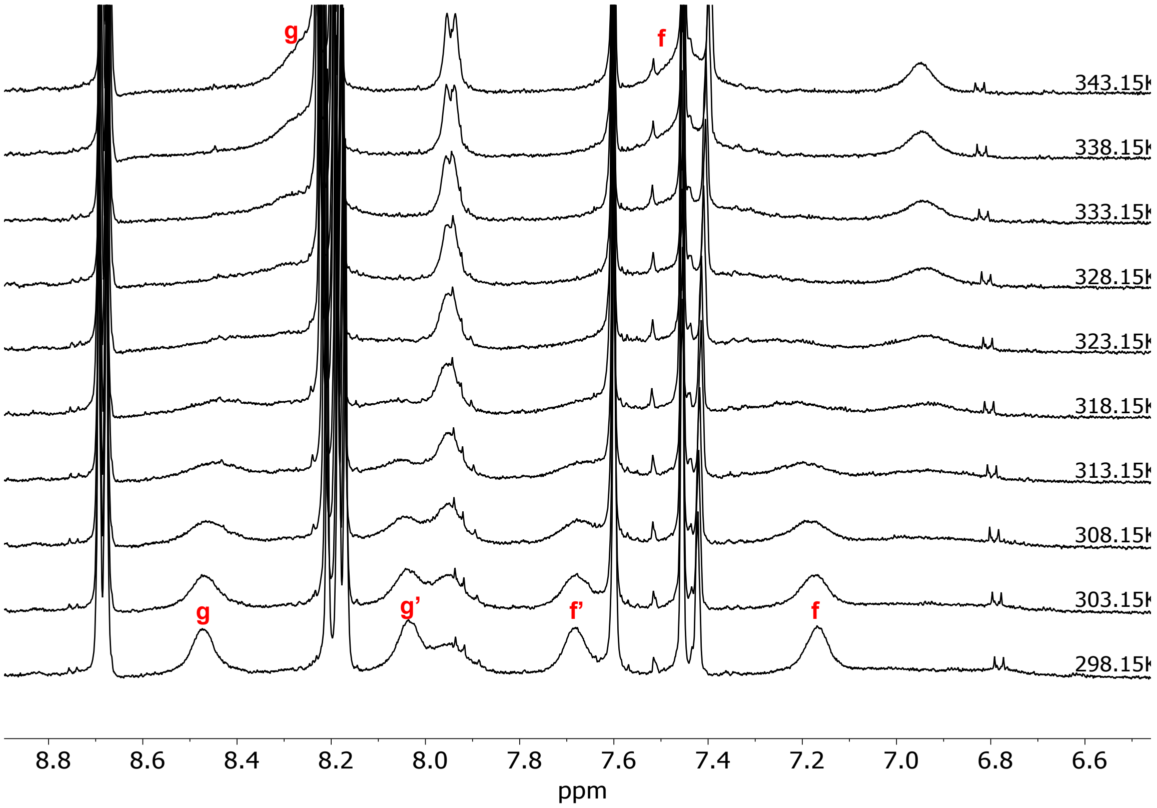

Figure S15: VT ^{1}H-NMR (400 MHz, D_2O) stacked spectra for F_a^{5+}.

Signal	$\Delta\nu$ (Hz)	T_c (K)	$\Delta G^{\neq}$ (kcal·mol^{-1})
f – f'	206	323.15	15.0
g – g'	175	328.15	15.4

Table S1: Experimental data obtained for the calculation of $\Delta G^{\neq}$ via coalescence temperatures of various signals on the VT ^{1}H NMR of F_a^{5+} in D_2O.

[3] a) J. Sandstrom, Dynamic NMR Spectroscopy; Academic Press: New York, NY, USA, **1983**. b) H. Kessler, Detection of Hindered Rotation and Inversion by NMR Spectroscopy, *Angew. Chem. Int. Ed.* 1970, **9**, 219-235.

2.3. Synthesis and characterization of F_b^{5+}.

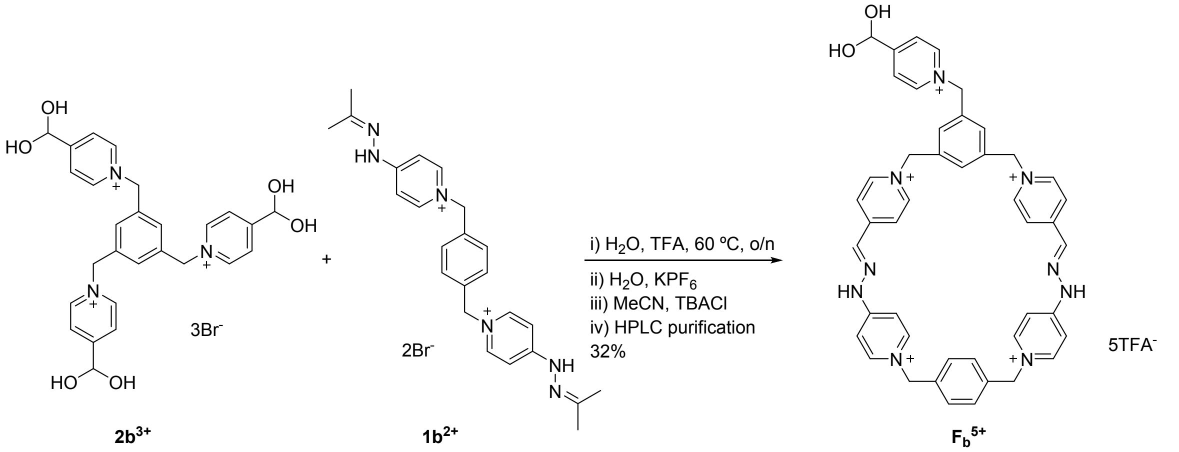

$2b^{3+}$ $1b^{2+}$ F_b^{5+}

A solution of 1_b^{2+} (70.0 mg, 0.125 mmol), 2_b^{3+} (92.0 mg, 0.125 mmol) and TFA (10% molar) in 50 mL of water was heated in a heat-on block at 60 ºC overnight. After cooling, excess of KPF_6 was added until no further precipitation was observed. The obtained solid was filtered and washed with water (3 × 5 mL) and ether (3 x 5 mL), yielding a reddish powder. The solid was redissolved in MeCN and an excess of TBACl was added until no more precipitate was formed. The solid was washed with MeCN (5 x 5 mL) and Et_2O (5 x 5 mL). A fraction of the solid (51.0 mg) was dissolved in H_2O + TFA (100 μL), filtered and purified by reverse-phase semipreparative HPLC (A: H_2O + 0.1% TFA, B: MeCN + 0.1% TFA), giving a yellowish solid (25.8 mg, 32%, F_b^{5+}).

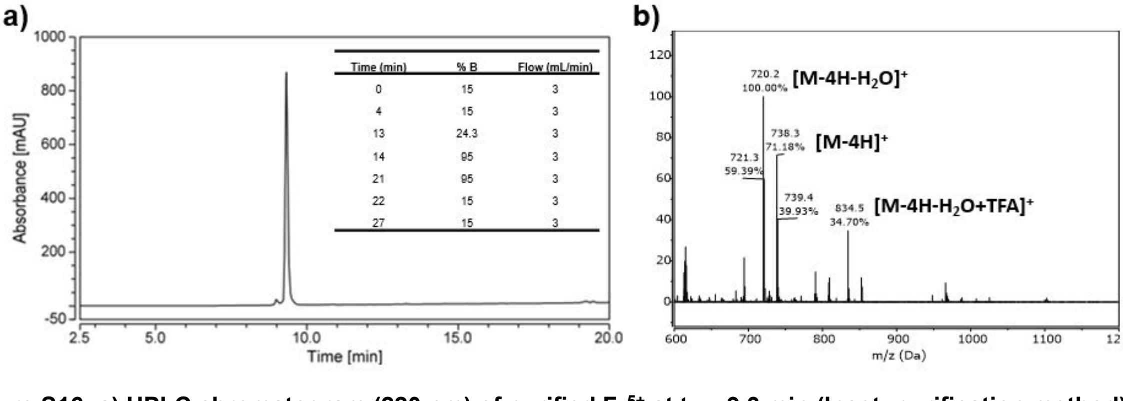

Figure S16: a) HPLC chromatogram (220 nm) of purified F_b^{5+} at t_R = 9.3 min (Inset: purification method). b) MS spectrum from the chromatographic peak at t_R = 9.3 min.

^1H NMR (500 MHz, D_2O) δ = 8.91 (d, J = 6.5 Hz, 2H), 8.75 (d, J = 6.5, 4H), 8.59 (s, 2 H), 8.24 (d, J = 6.4 Hz, 4H), 8.22 (s, 2H), 8.17 (d, J = 6.3 Hz, 2H), 7.74 (s, 2H), 7.71 (s, 1H), 7.64 (s, 1H), 7.56 (s, 4H), 7.22 (s, 2H), 6.20 (s, 1 H), 5.89 (s, 2 H), 5.79 (s, 4H), 5.54 (s, 4H). $^{13}C\{^1H\}$-NMR (125 MHz, D_2O): δ = 160.7 (C), 154.5 (C), 150.2 (C), 144.7 (CH), 144.1 (CH), 142.9

(CH), 140.5 (CH), 135.6 (C), 135.3 (C), 135.1 (C), 131.7 (CH), 131.5 (CH), 130.6 (CH), 125.8 (CH), 124.9 (CH), 110.0 (2 x CH), 87.4 (CH), 63.1 (CH_2), 63.0 (CH_2), 61.5 (CH_2). HRMS (ESI): *m/z* calculated for $C_{45}H_{41}N_9O_2^{2+}$ [*M*-3H]$^{2+}$ 369.6686, found 369.6688; *m/z* calculated for $C_{45}H_{39}N_9O^{2+}$ [*M*-3H-H_2O]$^{2+}$ 360.6634, found 360.6636; *m/z* calculated for $C_{49}H_{42}F_6N_9O_6^{2+}$ [*M*-2H+2TFA]$^{+}$ 966.3157, found 966.3175.

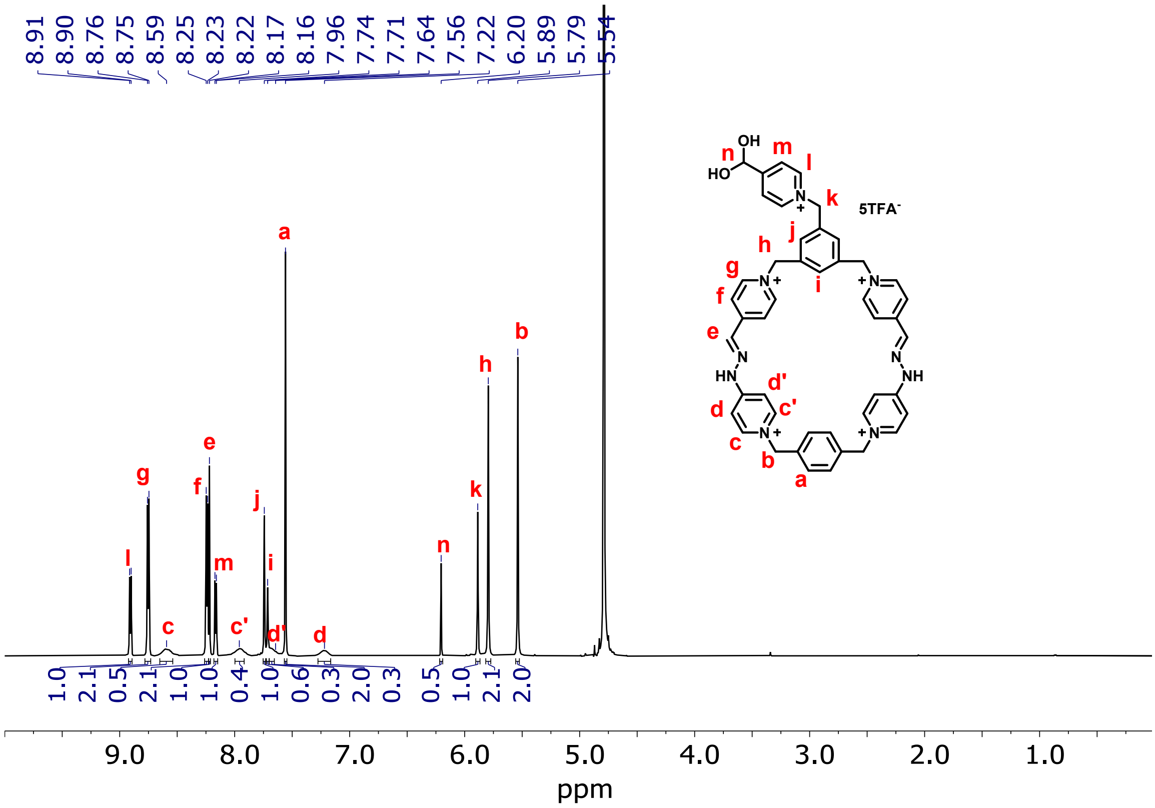

Figure S17: ^{1}H-NMR (500 MHz, D_2O) spectrum of F_b^{5+}.

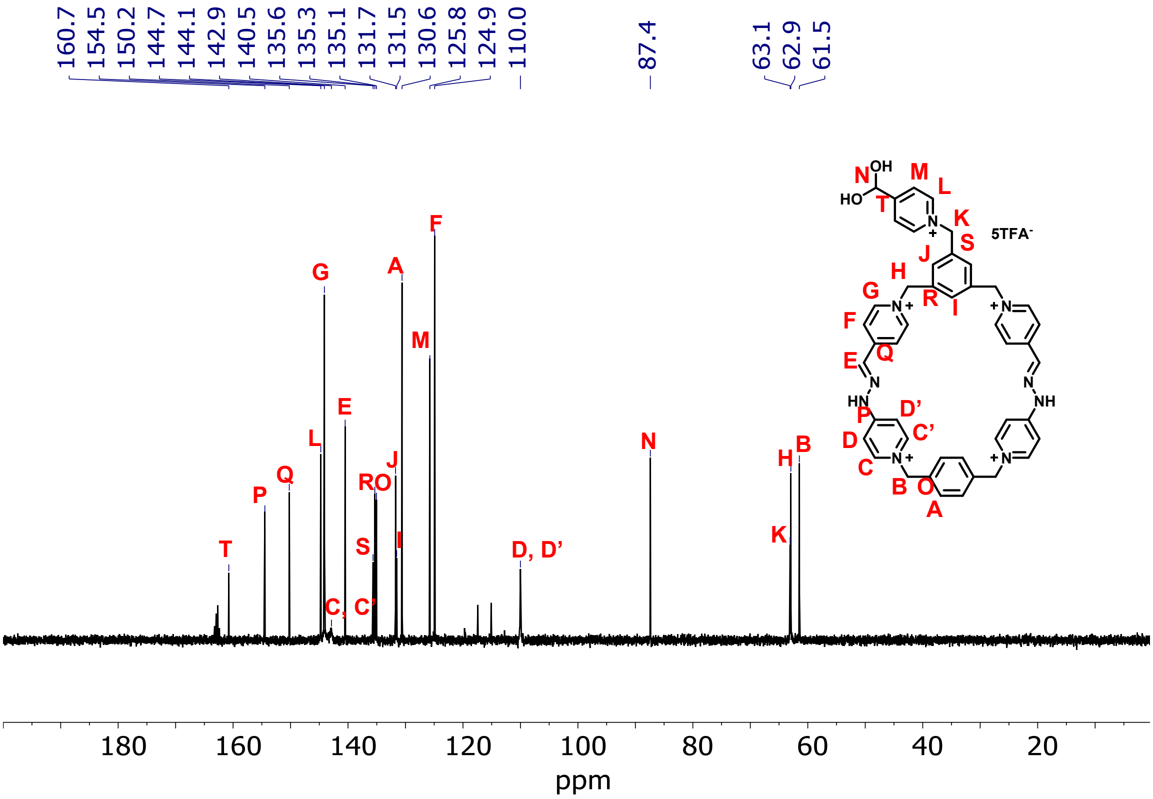

Figure S18: $^{13}C\{^{1}H\}$-NMR (126 MHz, D_2O) spectrum of F_b^{5+}.

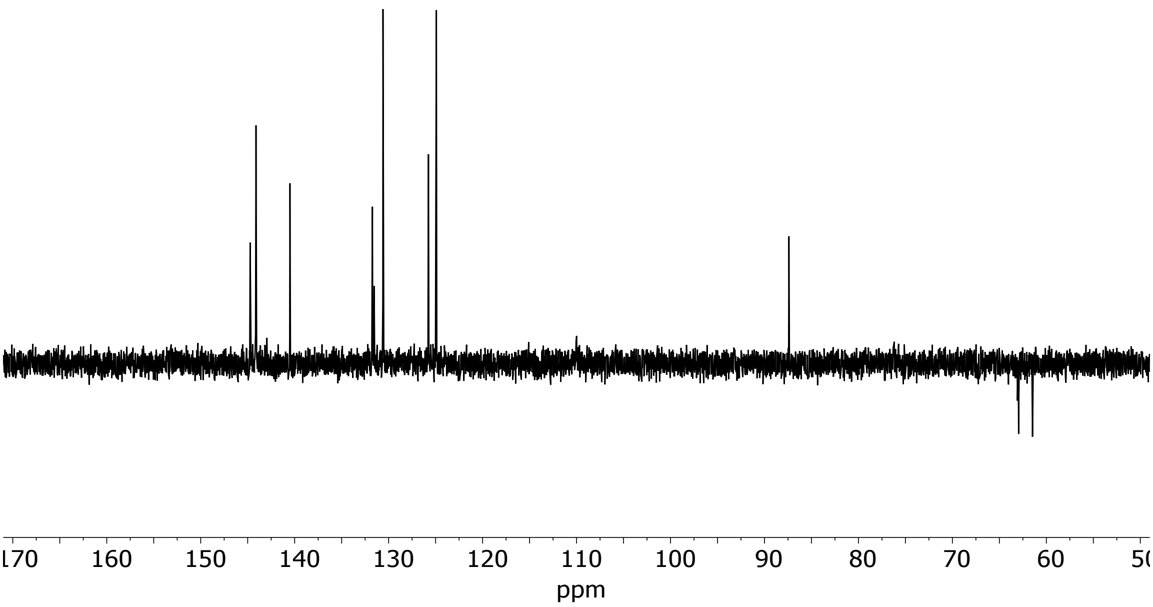

Figure S19: DEPT-135 (126 MHz, D_2O) spectrum of F_b^{5+}.

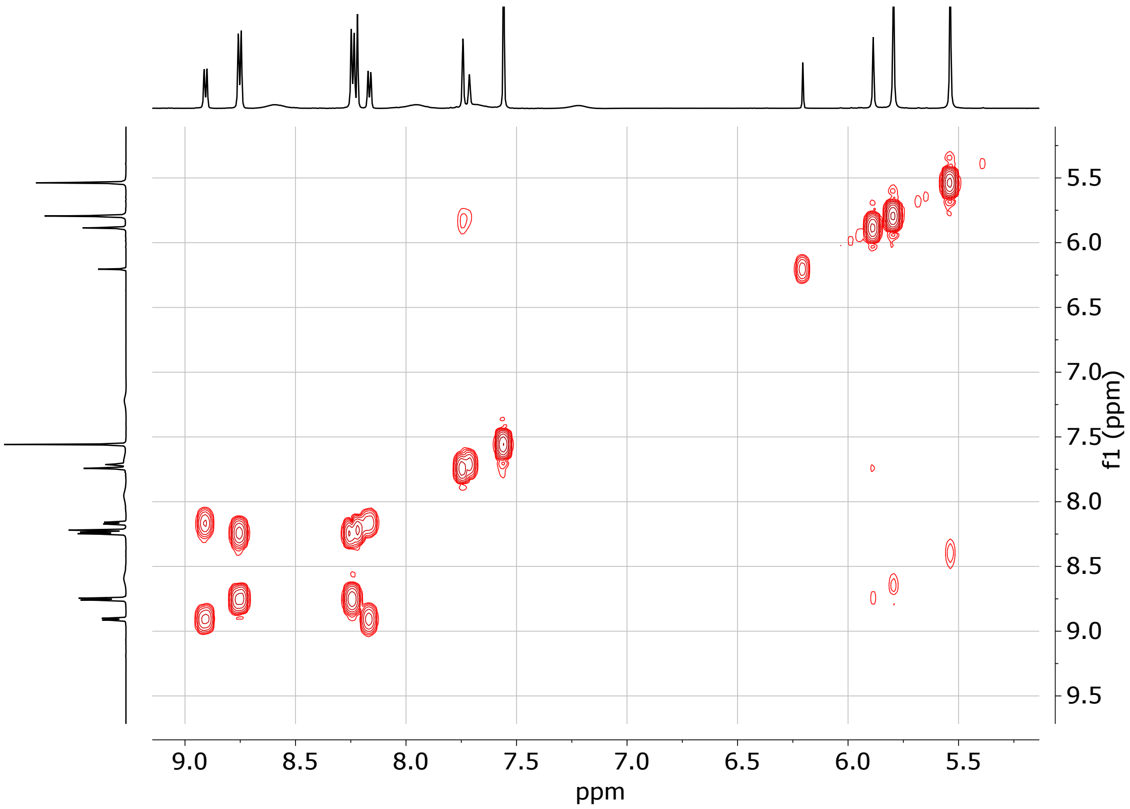

Figure S20: ^{1}H-^{1}H COSY (500 MHz, D_2O) spectrum of F_b^{5+}.

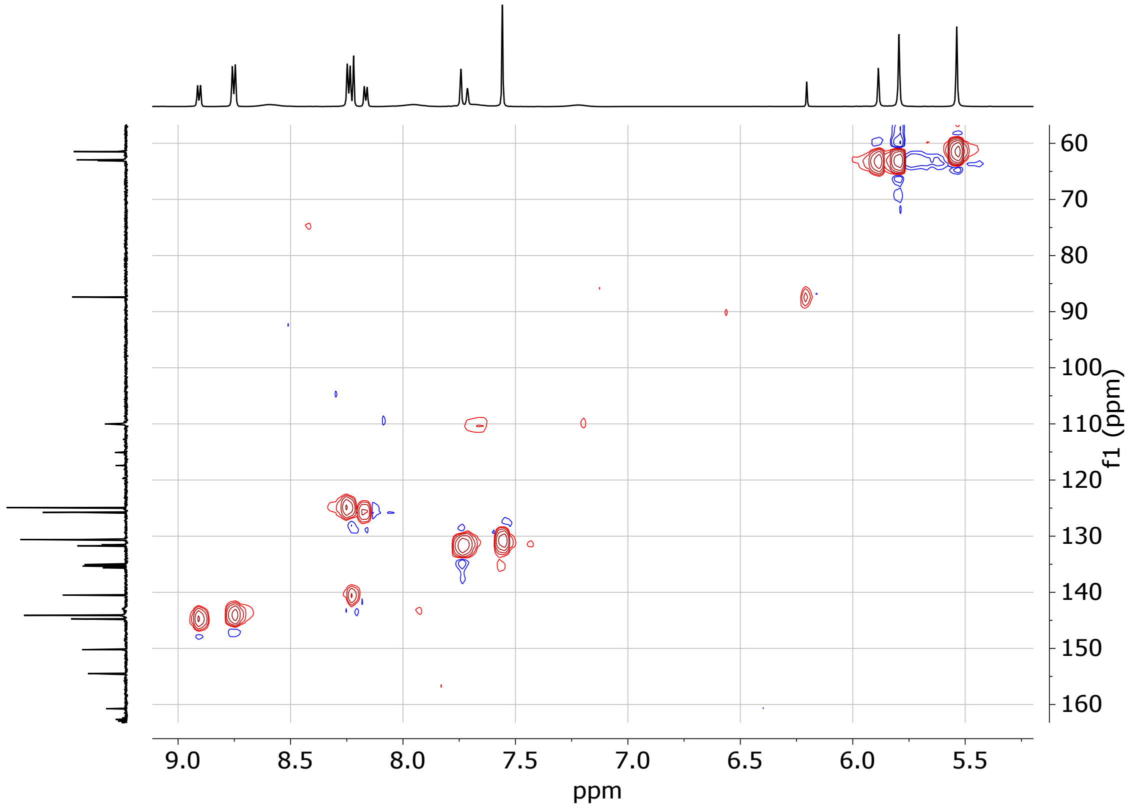

Figure S21: ^{1}H-$^{13}C\{^{1}H\}$ HSQC (500 and 126 MHz, D_2O) spectrum of F_b^{5+}.

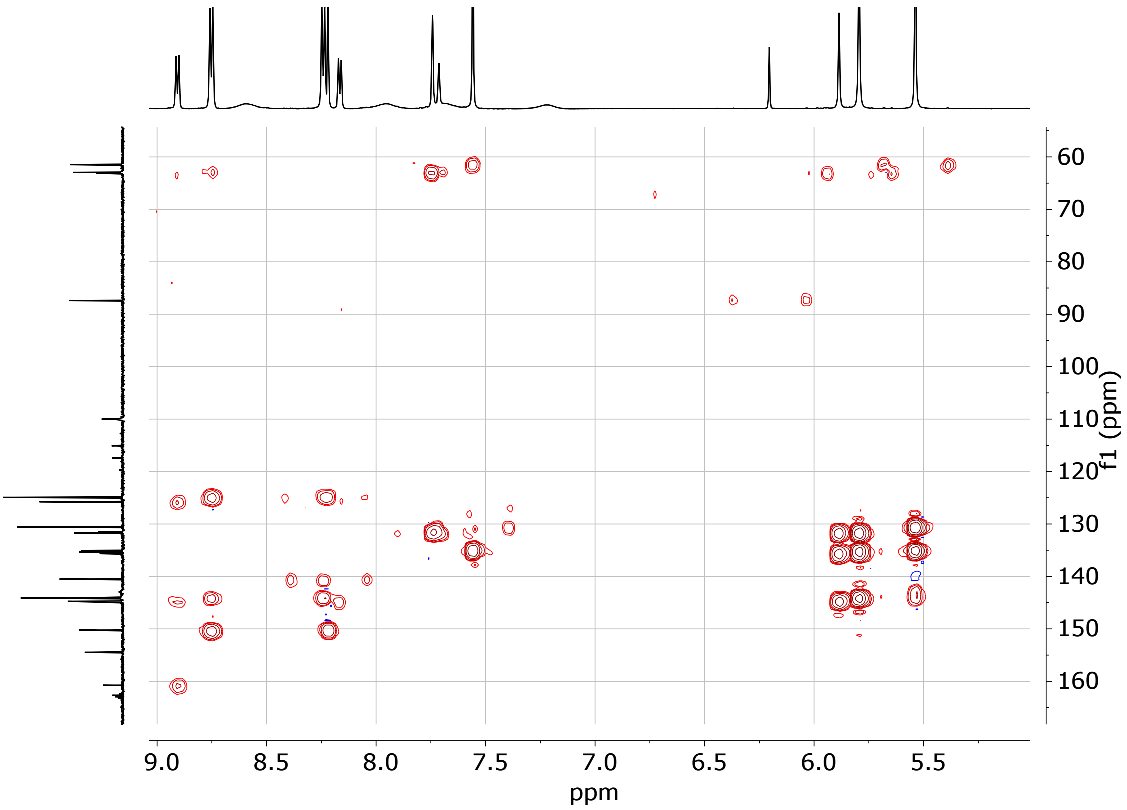

Figure S22: ^{1}H-$^{13}C\{^{1}H\}$ HMBC (500 and 126 MHz, D_2O) spectrum of F_b^{5+}.

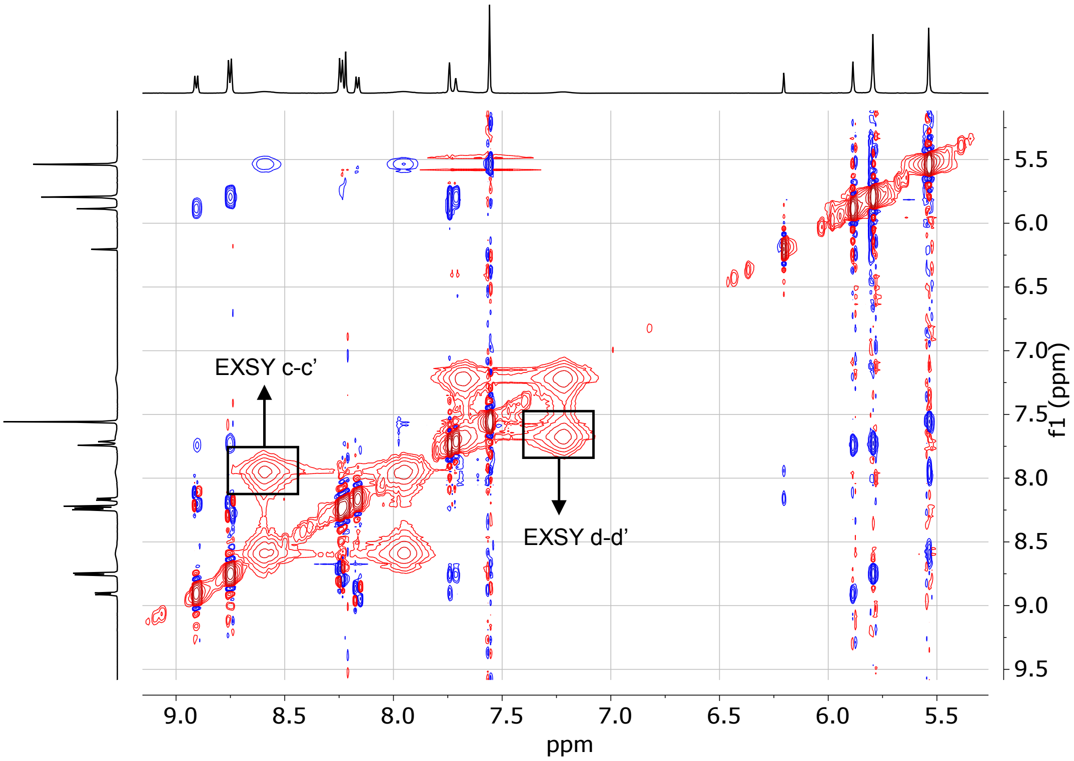

Figure S23: ^{1}H-^{1}H NOESY (500 MHz, D_2O) spectrum of F_b^{5+}.

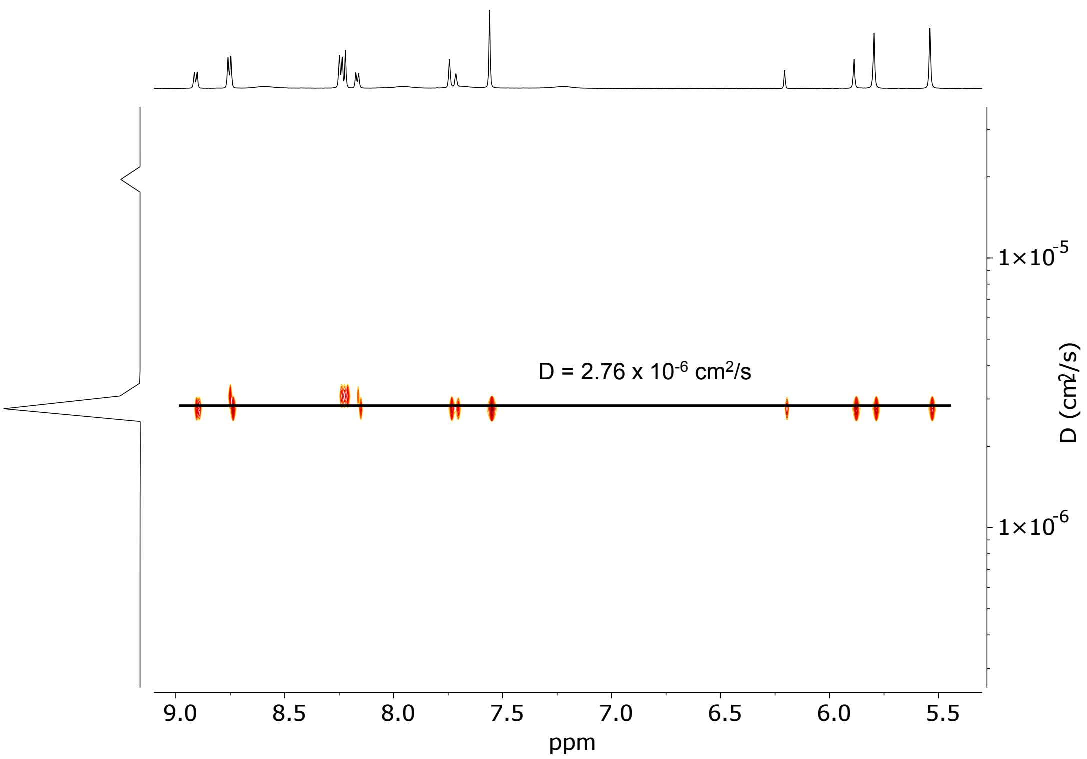

Figure S24: DOSY spectrum of F_b^{5+}.

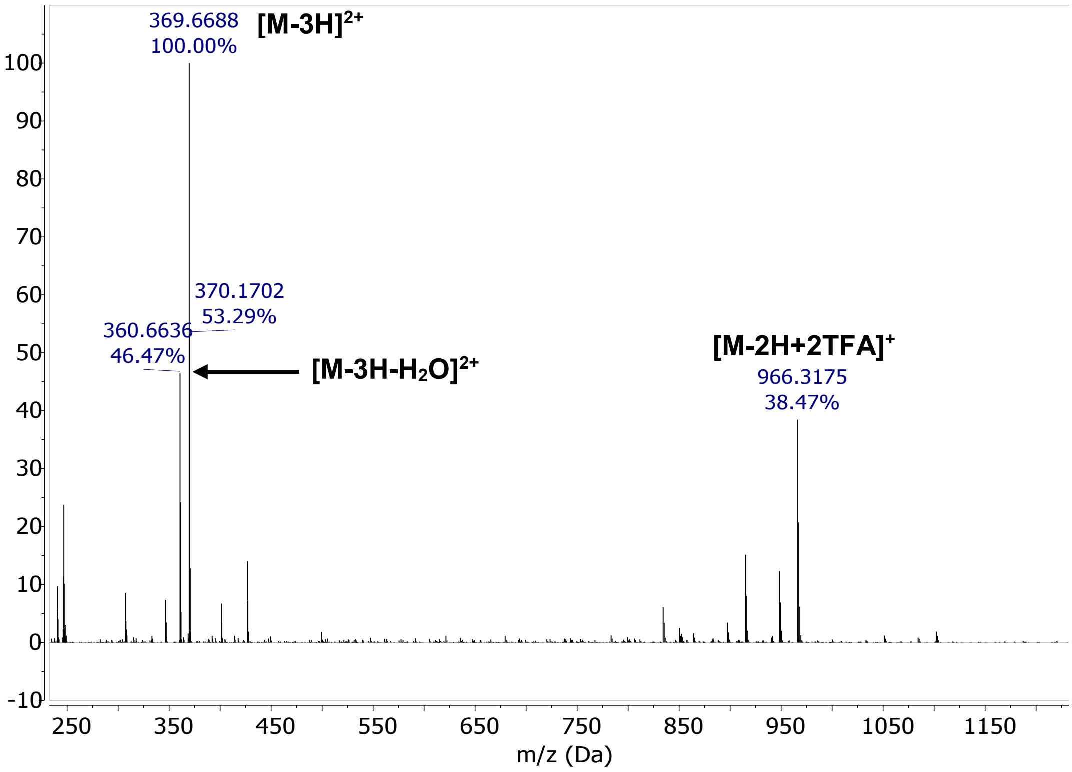

Figure S25: ESI-HRMS of F_b^{5+}.

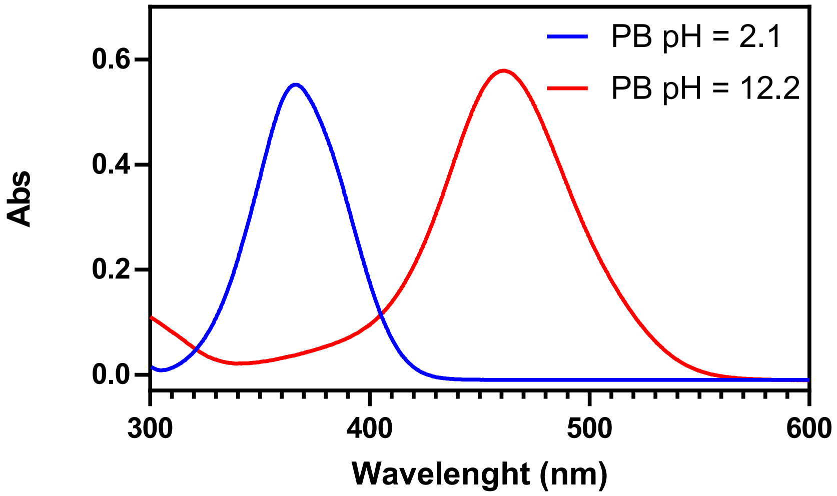

Figure S26: UV-Vis absorption spectra (phosphate buffer 10 mM) of F_b^{5+} at pH = 2 (blue) and 12 (red).

2.4. Determination of the energy of the rotational barrier ($\Delta G^{\neq}$) for F_b^{5+}.

The coalescence temperature (T_c) could be estimated for different protons on VT NMR experiments. This provides, in association with the maximum peak separation ($\Delta\nu$ in Hz) at slow exchange between **c** – **c'** and **d** – **d'** for $\mathbf{F_b}^{5+}$, the energy of the rotational barrier using Equation (1).

$$\Delta G^{\neq} = 4.57 \cdot 10^{-3} T_c(9.972 + \log T_c/\Delta\nu) \quad (1)$$

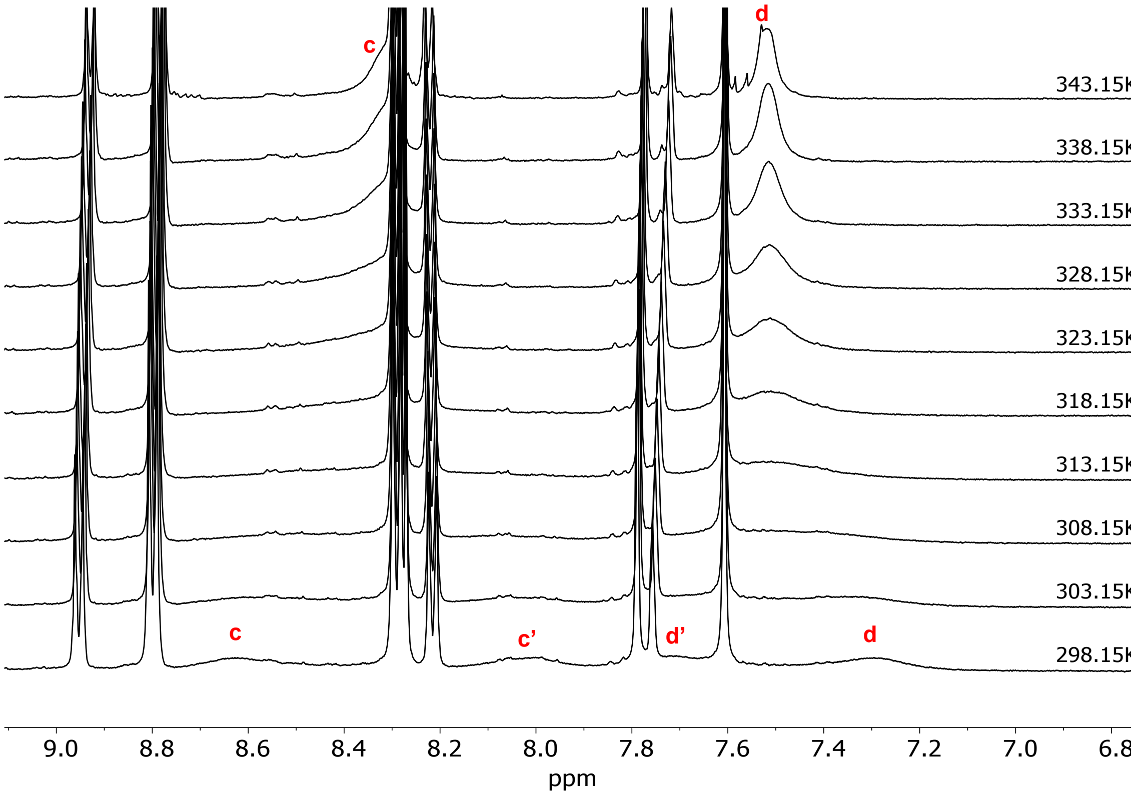

Figure S27: VT ^{1}H-NMR (400 MHz, D_2O) stacked spectra for F_b^{5+}.

Signal	Δv (Hz)	T_c (K)	$\Delta G^{\neq}$ (kcal·mol^{-1})
c – c'	246	313.15	14.4
d – d'	170	308.15	14.4

Table S2: Experimental data obtained for the calculation of $\Delta G^{\neq}$ via coalescence temperatures of various signals on the VT ^{1}H NMR of F_b^{5+} in D_2O.

2.5. Macrocyclization side products.

2.5.1. Characterization of A_a^{4+}.

$\mathbf{A_a}^{4+}$ was obtained as a side product from the condensation reaction for the synthesis of $\mathbf{F_a}^{5+}$, yielding a yellowish solid (4.0 mg, 8%).

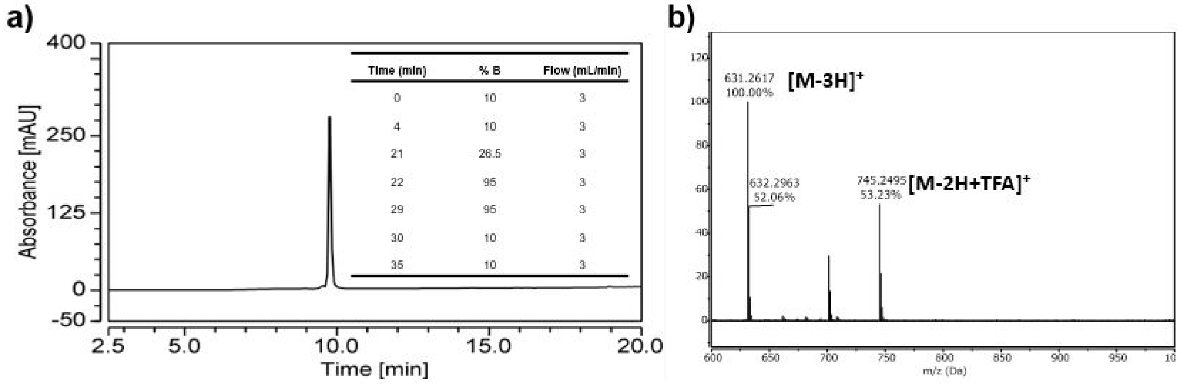

Figure S28: a) HPLC chromatogram (220 nm) of purified A_a^{4+} at t_R = 9.8 min (Inset: purification method). b) MS spectrum from the chromatographic peak at t_R = 9.8 min.

^{1}H-NMR (D_2O, 500 MHz): δ = 8.66 (d, J = 6.5 Hz, 4H), 8.52 (s, 2H), 8.20 (s, 2H), 8.17 (d, J = 6.4 Hz, 4H), 8.02 (s, 2H), 7.68 (s, 2H), 7.58 (s, 4H), 7.49 (s, 2H), 7.36 (s, 1H), 7.17 (s, 2H), 5.77 (s, 4H), 5.46 (s, 4H), 4.59 (s, 2H). ^{13}C{^{1}H}-NMR (D_2O, 125 MHz): δ = 154.4 (C), 149.9 (C), 144.0 (CH), 143.9 (CH), 143.1 (CH, C), 142.9 (CH), 140.7 (CH), 135.0 (C), 134.4 (C), 131.0 (CH), 129.0 (CH), 128.5 (CH), 124.7 (CH), 110.2 (CH), 109.7 (CH), 63.4 (CH_2), 62.7 (CH_2), 61.3 (CH_2). HRMS (ESI): m/z calculated for $C_{39}H_{36}N_8O^{2+}$ [M-2H]$^{2+}$ 316.1501, found 316.1500.

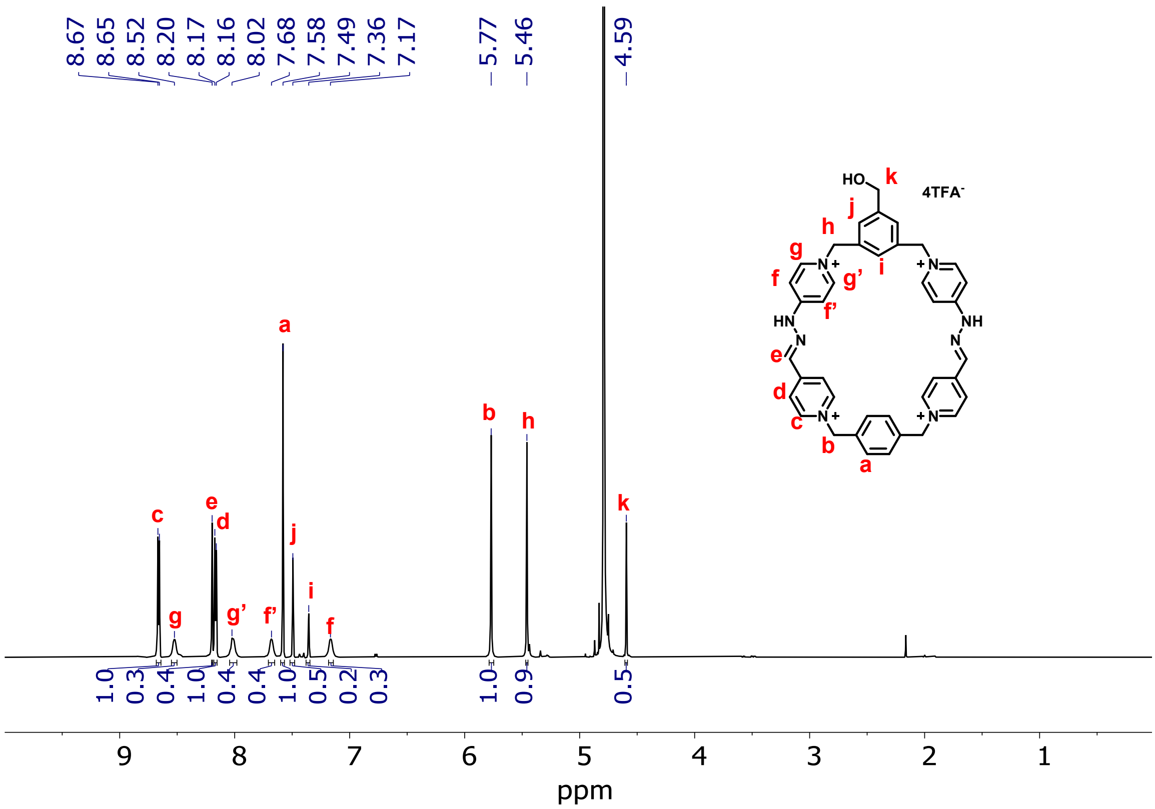

Figure S29: ^{1}H-NMR (500 MHz, D_2O) spectrum of A_a^{4+}.

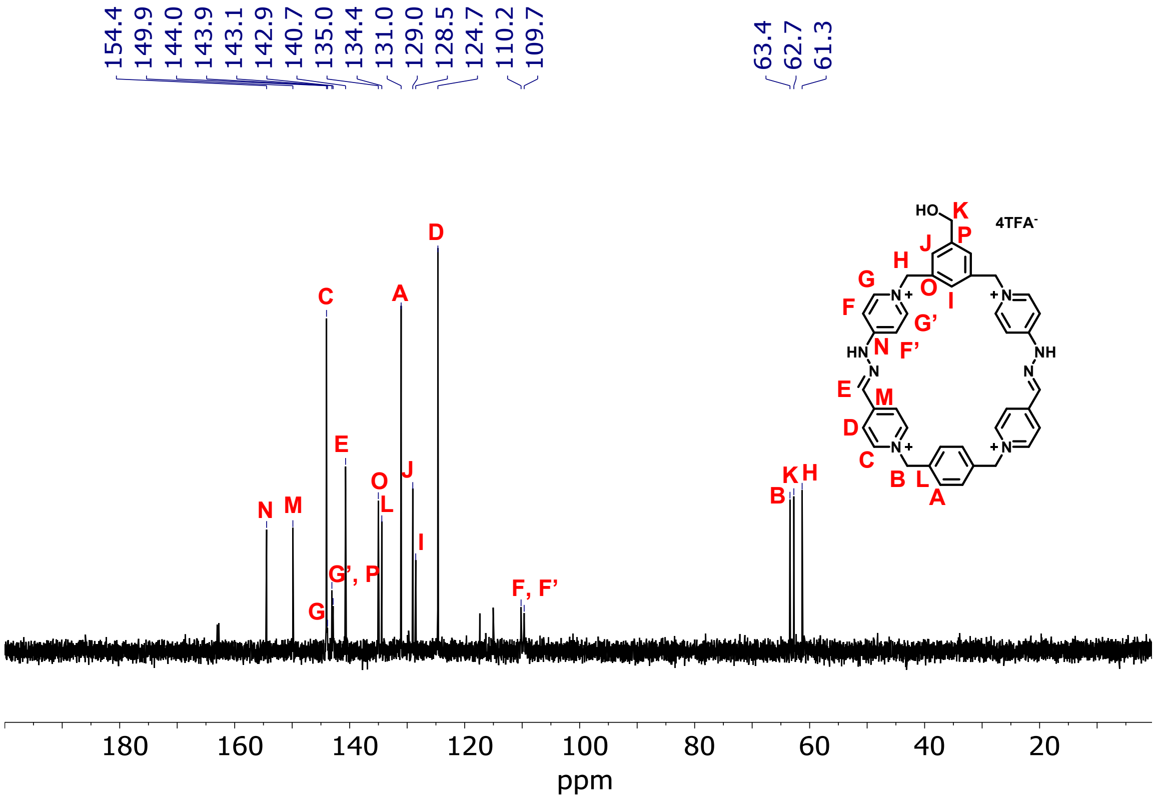

Figure S30: $^{13}C\{^{1}H\}$**-NMR (126 MHz,** D_2O**) spectrum of** A_a^{4+}**.**

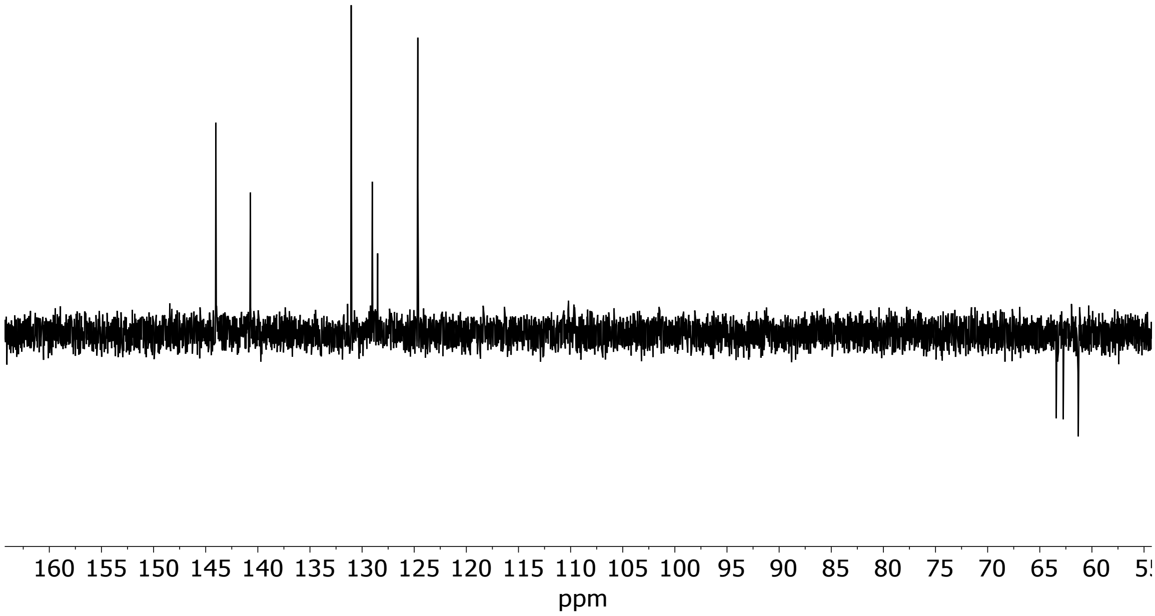

Figure S31: DEPT-135 (126 MHz, D_2O**) spectrum of** A_a^{4+}**.**

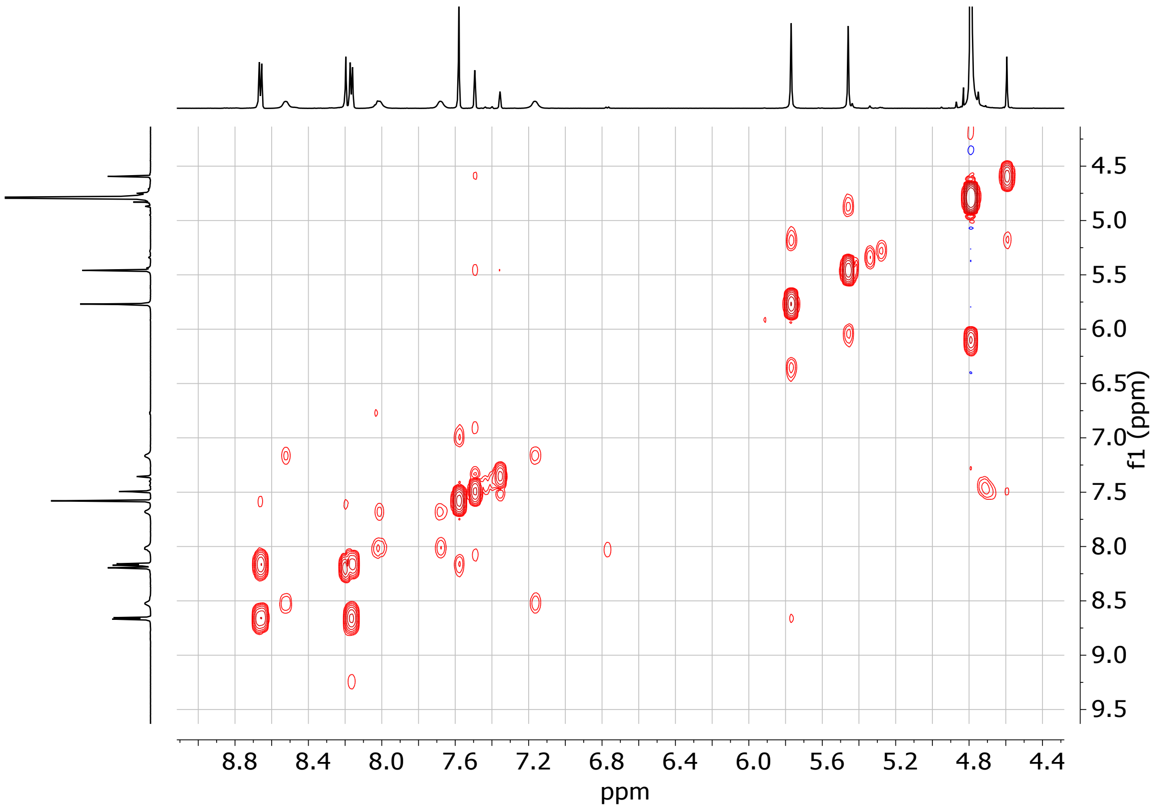

Figure S32: ^1H-^1H COSY (500 MHz, D_2O) spectrum of A_a^{4+}.

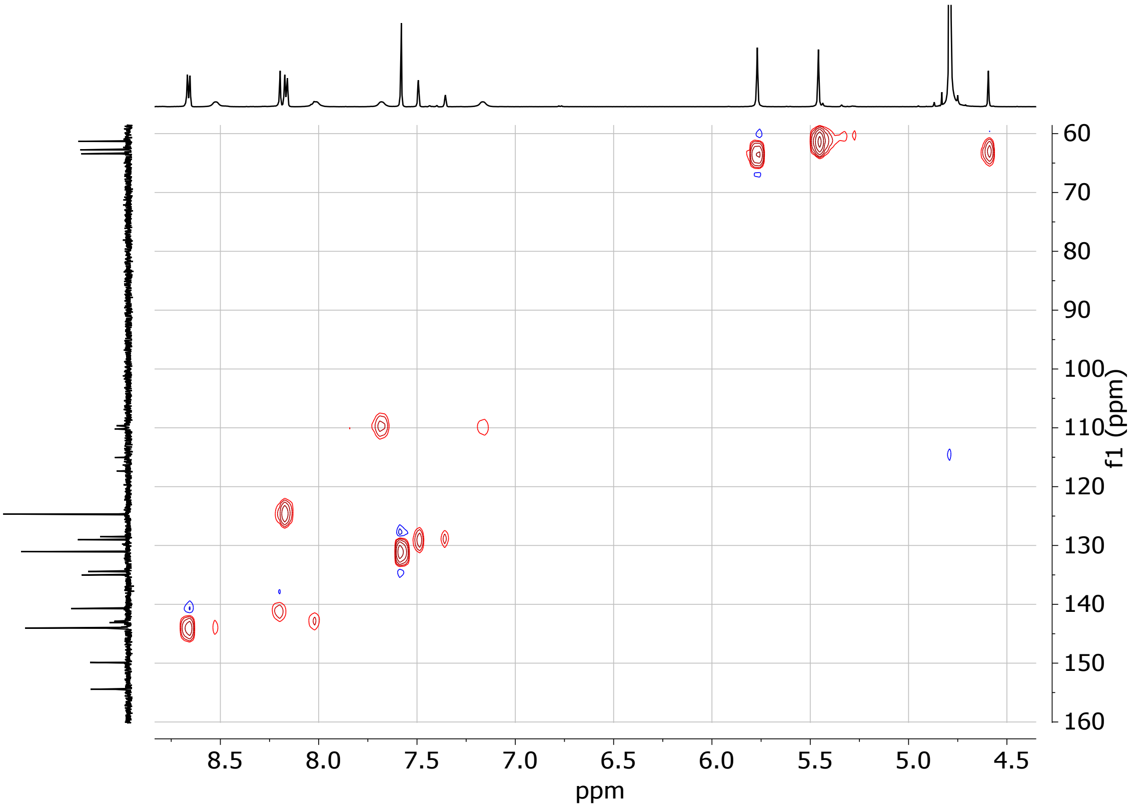

Figure S33: ^1H-$^{13}C\{^1H\}$ HSQC (500 and 126 MHz, D_2O) spectrum of A_a^{4+}.

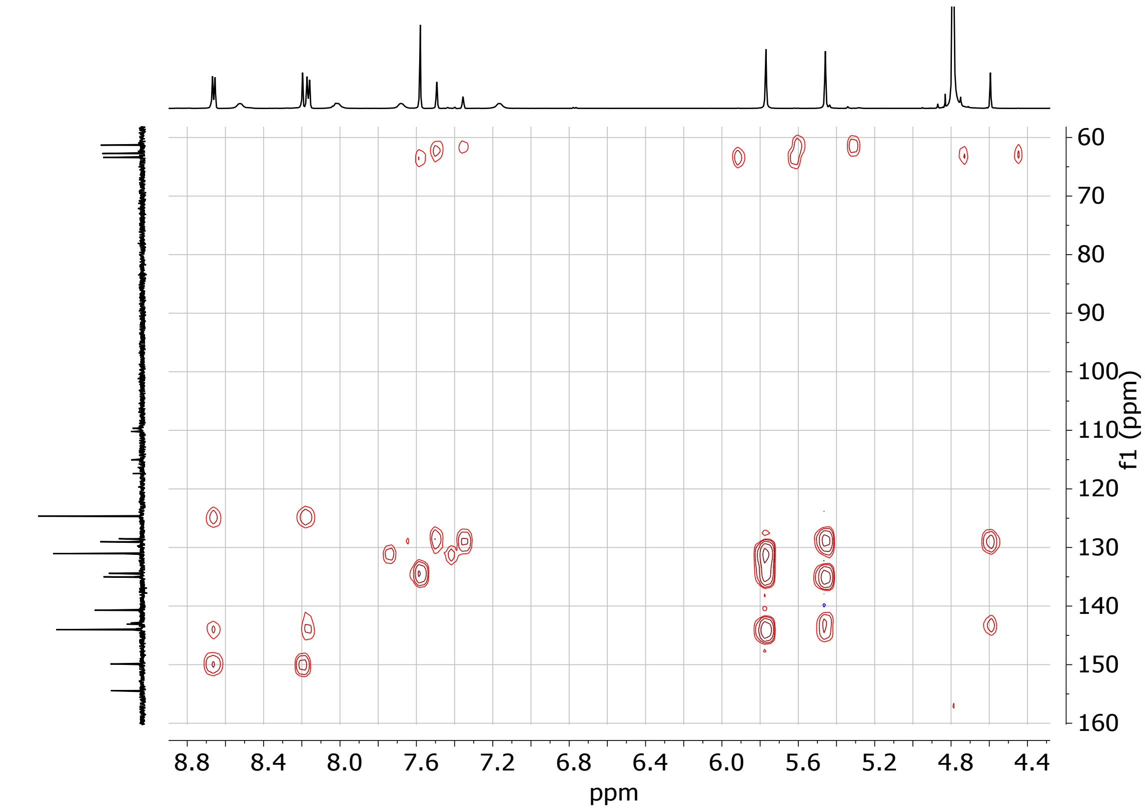

Figure S34: ^{1}H-$^{13}C\{^{1}H\}$ HMBC (500 and 126 MHz, D_2O) spectrum of A_a^{4+}.

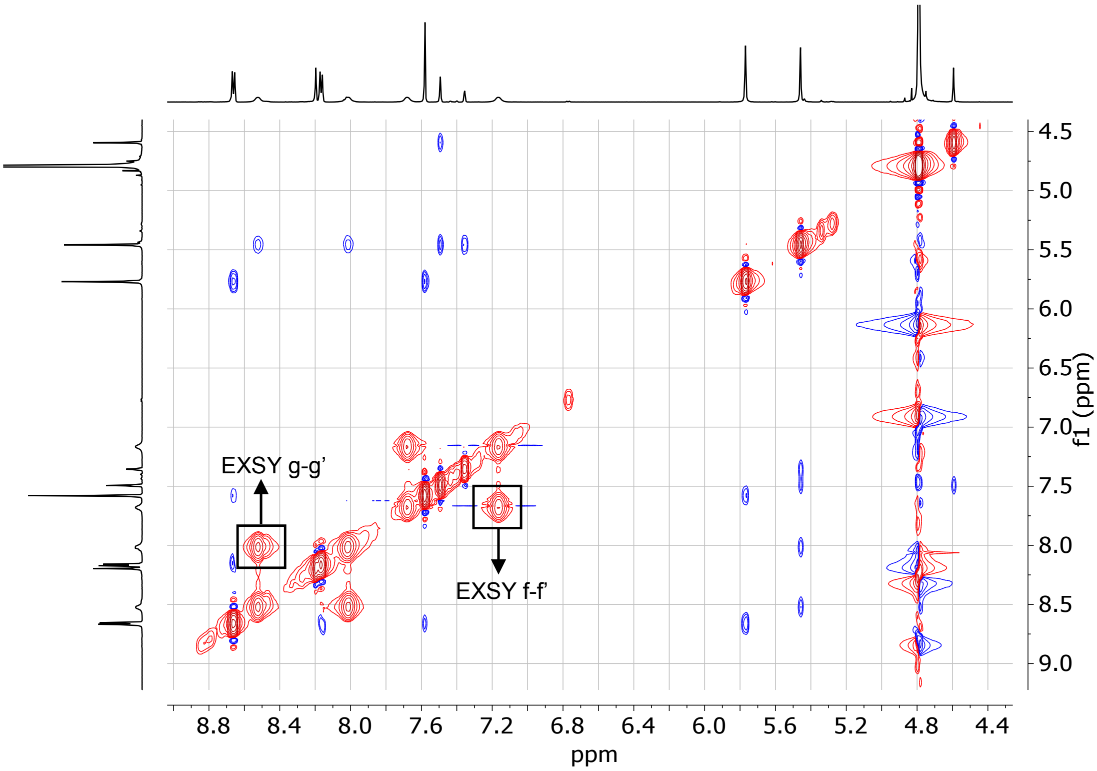

Figure S35: ^{1}H-^{1}H NOESY (500 MHz, D_2O) spectrum of A_a^{4+}.

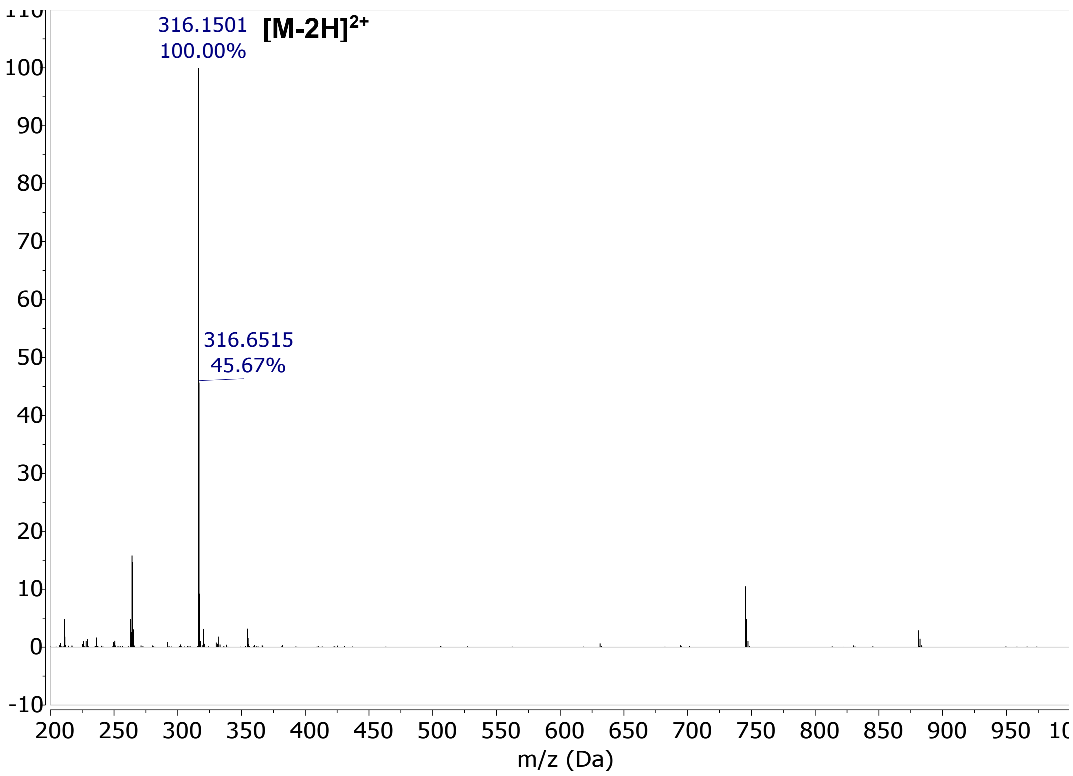

Figure S36: ESI-HRMS of A_a^{4+}.

2.5.2. Characterization of A_b^{4+}.

A_b^{4+} was obtained as a side product from the condensation reaction for the synthesis of **F_b^{5+}**, yielding a yellowish solid (10.4 mg, 15%).

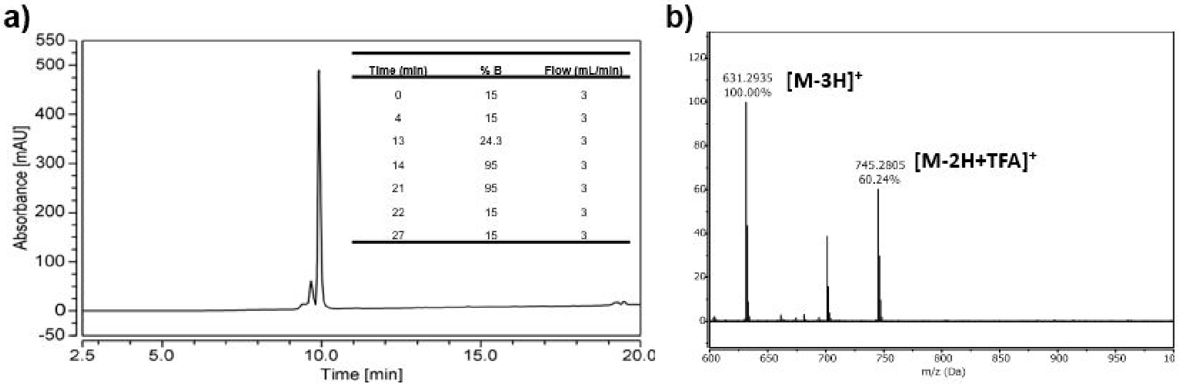

Time (min)	% B	Flow (mL/min)
0	15	3
4	15	3
13	24.3	3
14	95	3
21	95	3
22	15	3
27	15	3

Figure S37: a) HPLC chromatogram (220 nm) of purified A_b^{4+} at t_R = 9.9 min (Inset: purification method). b) MS spectrum from the chromatographic peak at t_R = 9.9 min.

^{1}H-NMR (D_2O, 500 MHz): δ = 8.72 (d, J = 6.5 Hz, 4H), 8.54 (s, 2H), 8.19 (d, J = 6.9 Hz, 6H), 7.87 (s, 2H), 7.64 (s, 2H), 7.59 (s, 2H), 7.54 (s, 1H), 7.50 (s, 4H), 7.16 (s, 2H), 5.75 (s, 4H), 5.48 (s, 4H), 4.61 (s, 2H). ^{13}C{^{1}H}-NMR (D_2O, 125 MHz): δ = 154.4 (C), 150.0 (C), 144.0 (CH),

143.5 (C), 142.7 (CH), 140.5 (CH), 134.9 (C), 134.0 (C), 130.6 (CH), 130.0 (CH), 129.6 (CH), 124.7 (CH), 109.8 (CH), 63.3 (CH_2), 62.6 (CH_2), 61.3 (CH_2). HRMS (ESI): *m/z* calculated for $C_{39}H_{36}N_8O^{2+}$ [*M*-2H]$^{2+}$ 316.1501, found 316.1500.

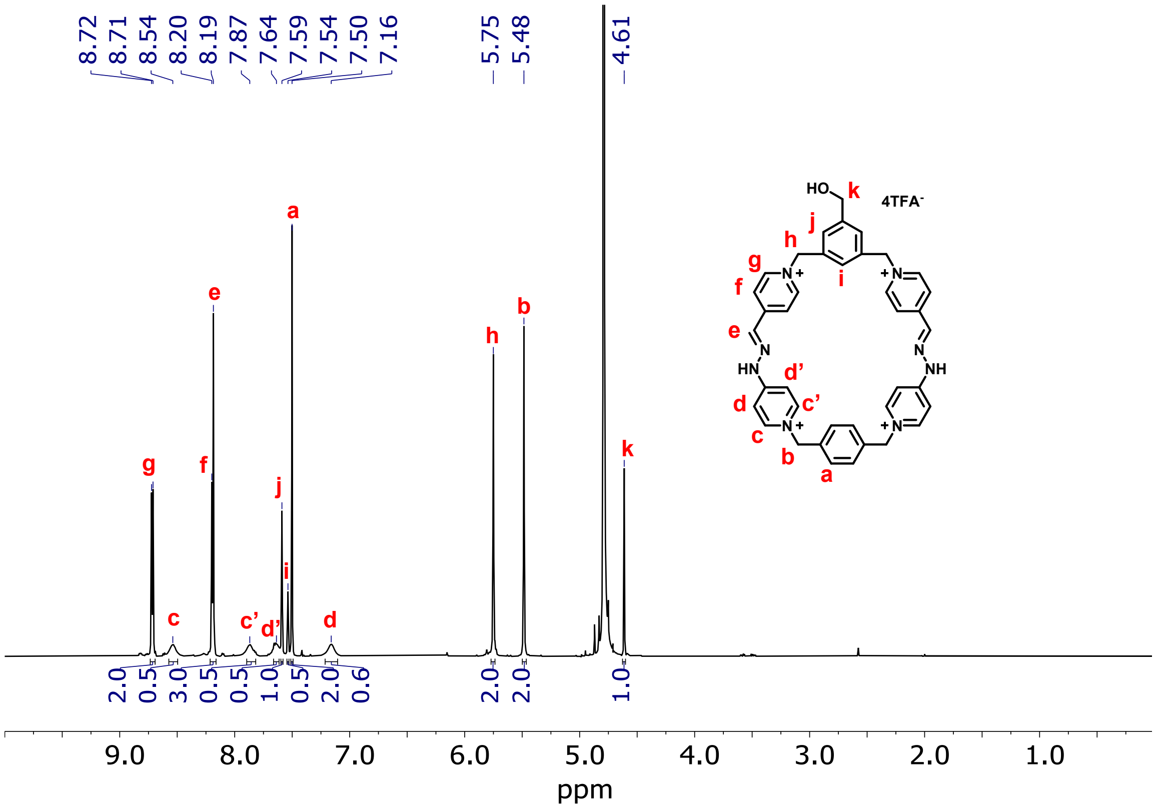

Figure S38: ^{1}H-NMR (500 MHz, D_2O) spectrum of A_b^{4+}.

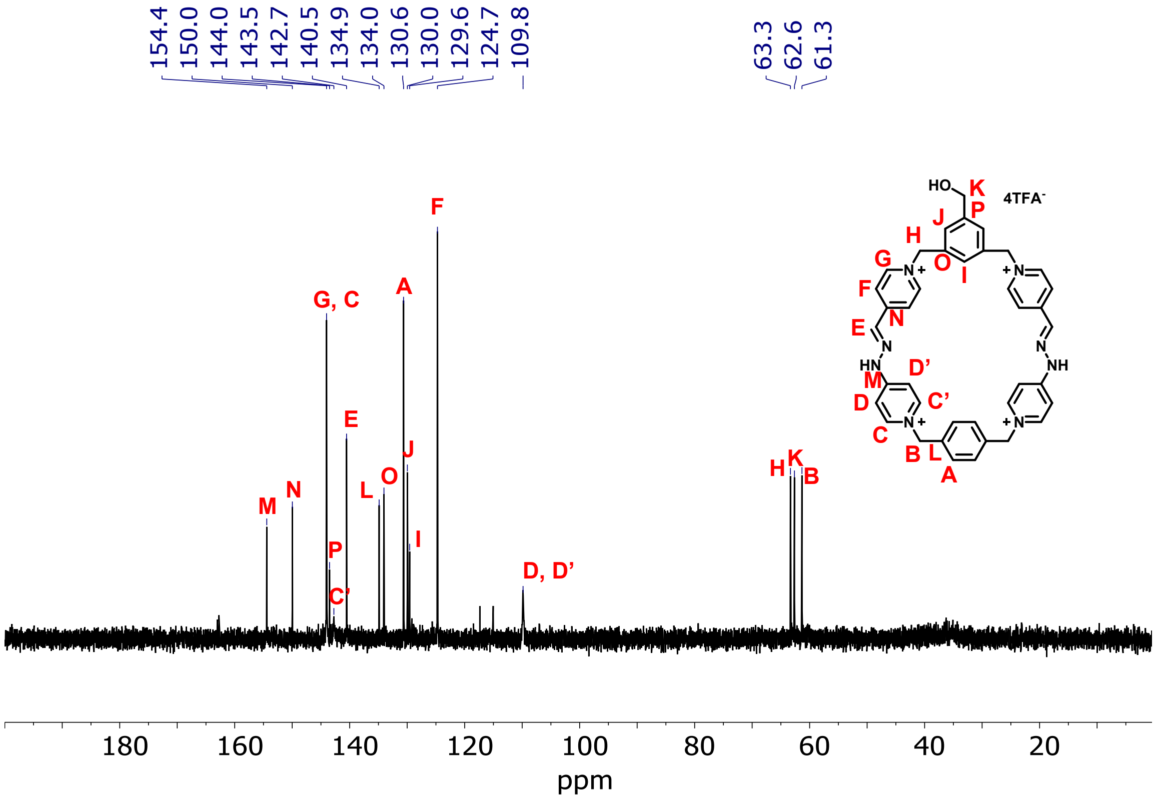

Figure S39: $^{13}C\{^{1}H\}$-NMR (126 MHz, D_2O) spectrum of A_b^{4+}.

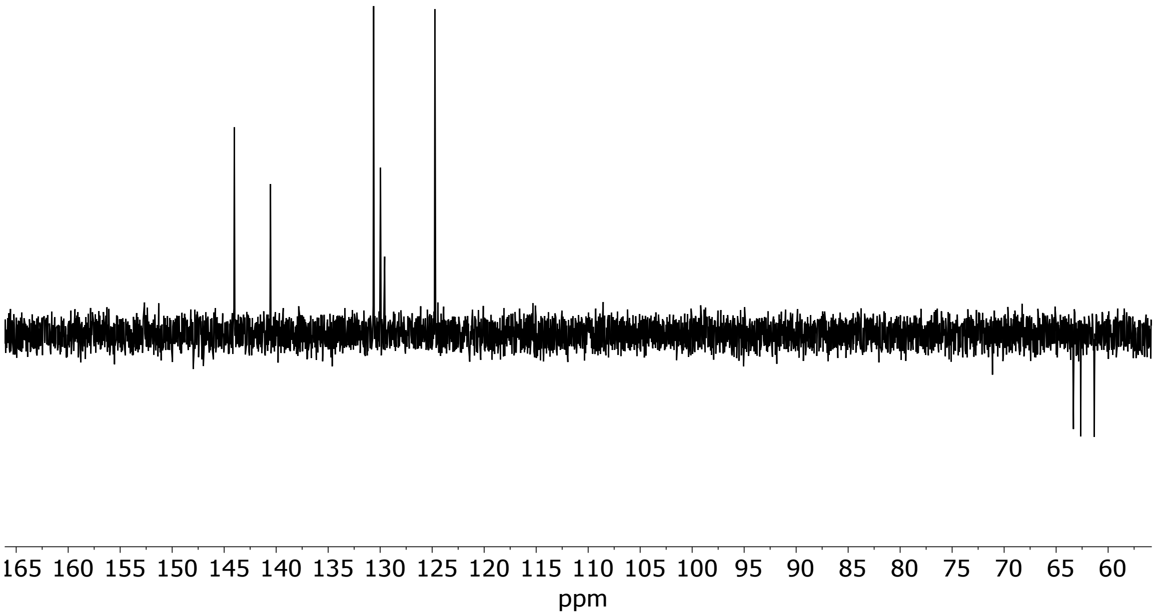

Figure S40: DEPT-135 (126 MHz, D_2O) spectrum of A_b^{4+}.

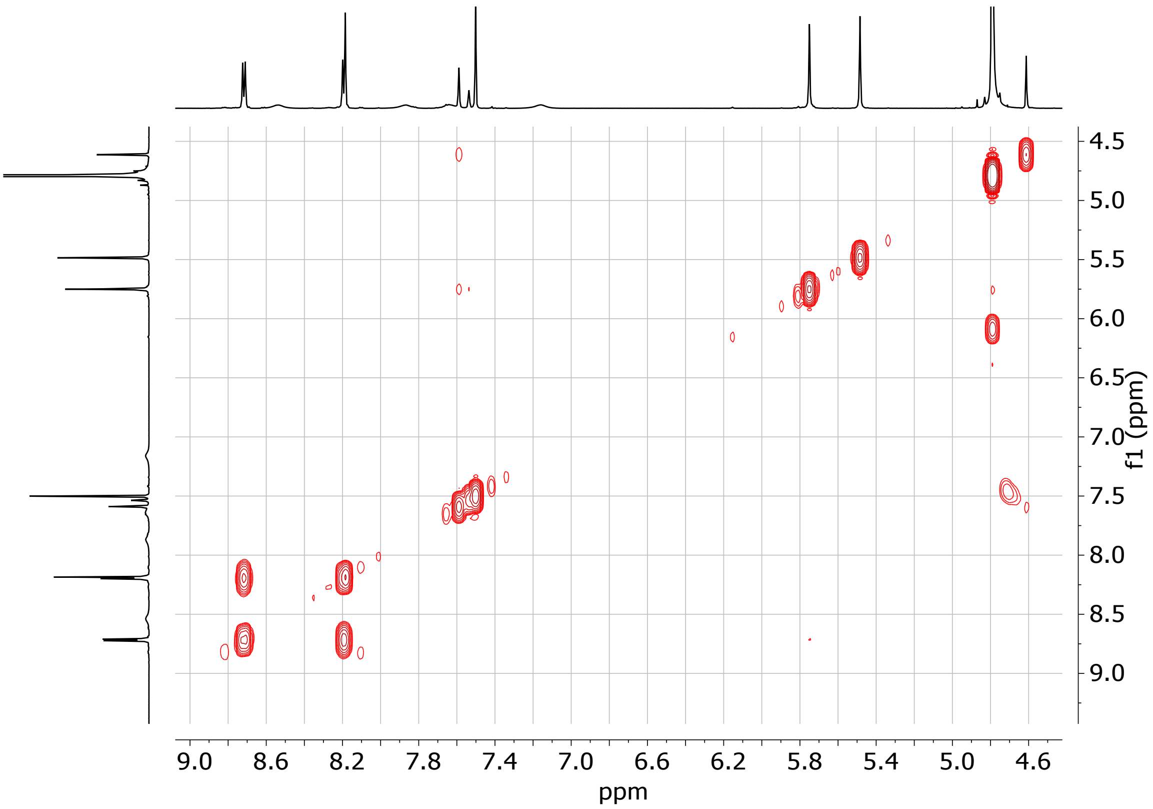

Figure S41: ^{1}H-^{1}H COSY (500 MHz, D_2O) spectrum of A_b^{4+}.

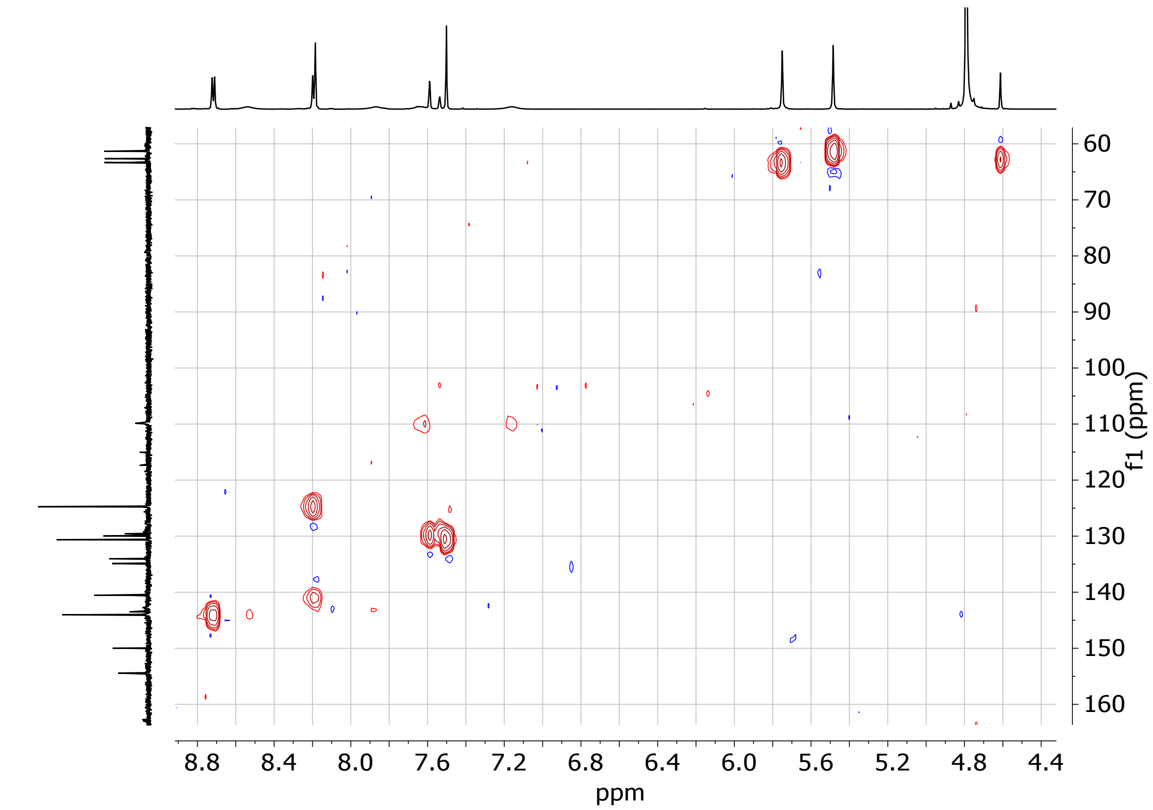

Figure S42: ^{1}H-$^{13}C\{^{1}H\}$ HSQC (500 and 126 MHz, D_2O) spectrum of A_b^{4+}.

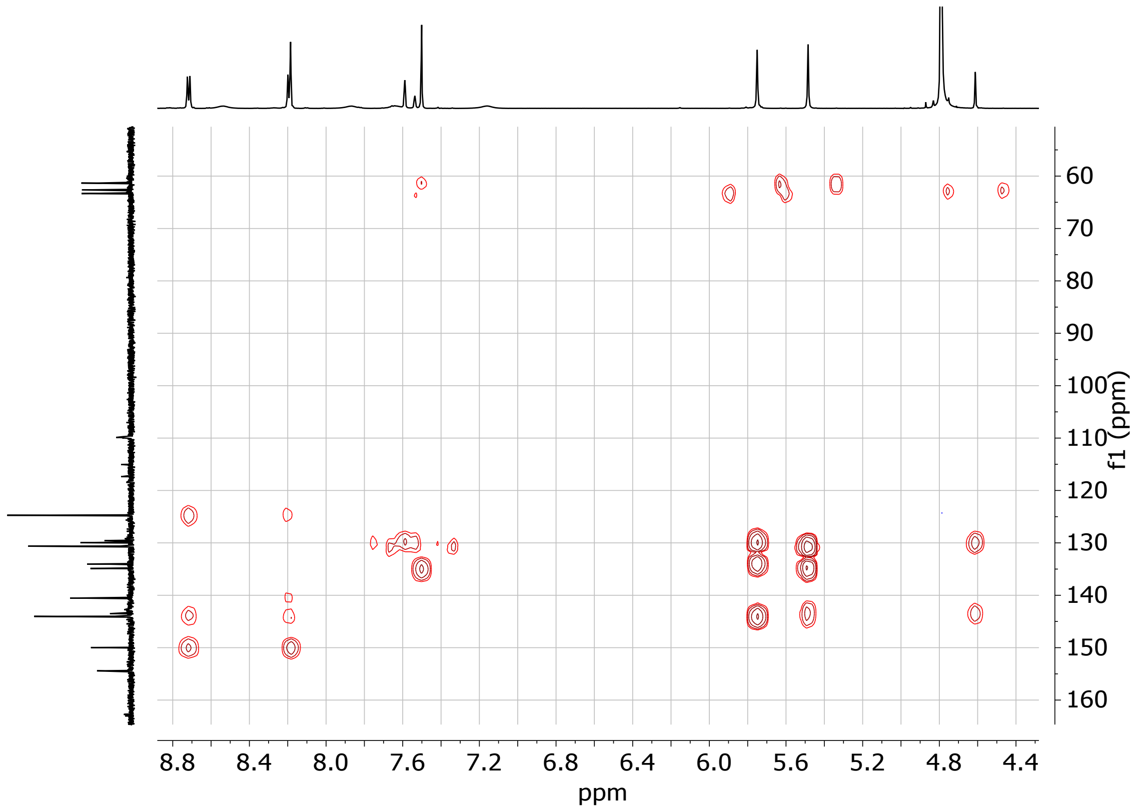

Figure S43: ^{1}H-$^{13}C\{^{1}H\}$ HMBC (500 and 126 MHz, D_2O) spectrum of A_b^{4+}.

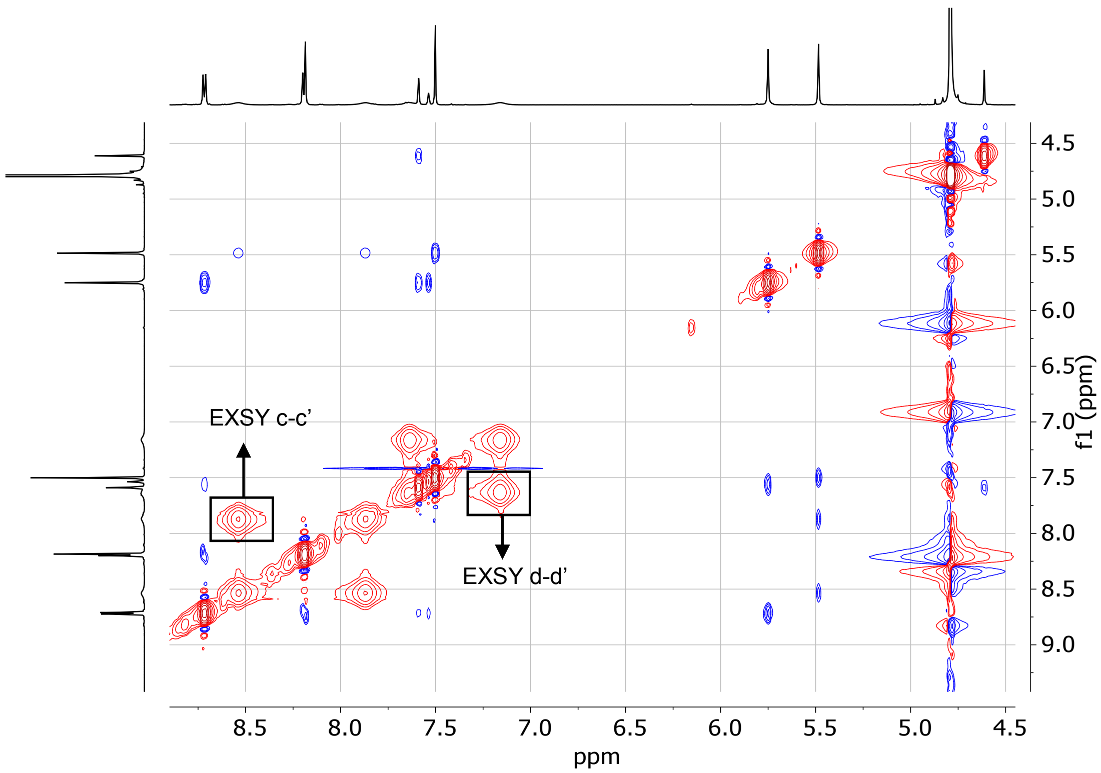

Figure S44: ^{1}H-^{1}H NOESY (500 MHz, D_2O) spectrum of A_b^{4+}.

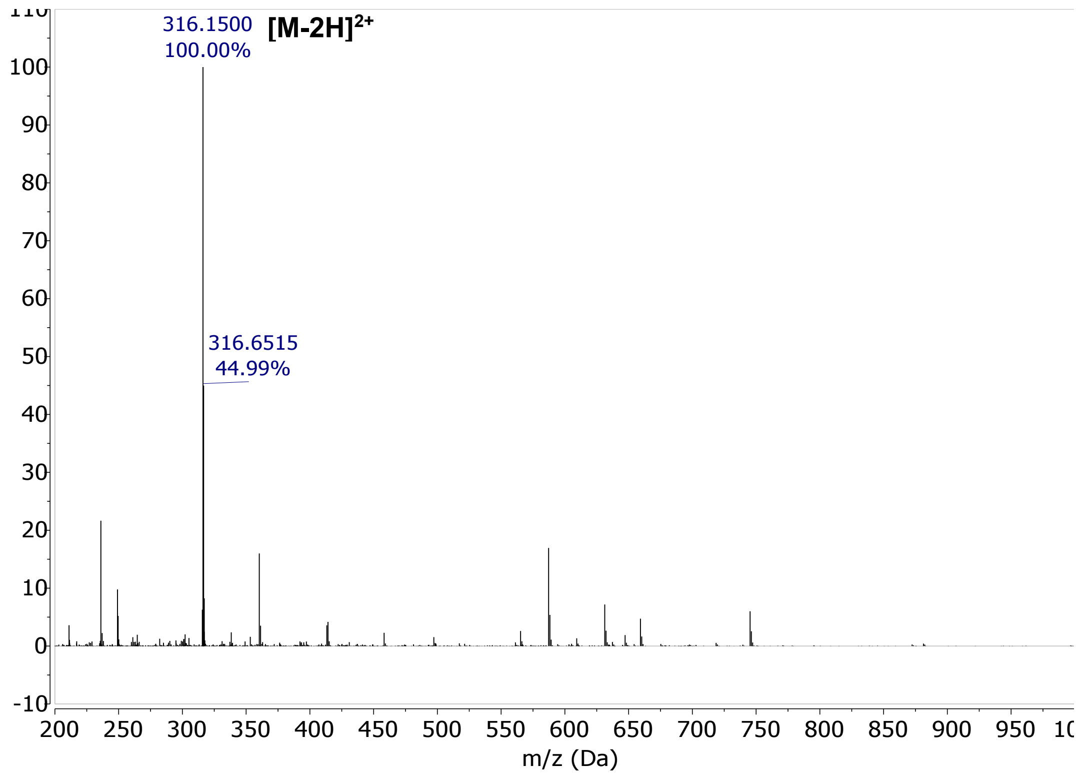

Figure S45: ESI-HRMS of A_b^{4+}.

2.5.3. Characterization of D^{12+}.

D^{12+} was obtained as a side product from the condensation reaction for the synthesis of $\mathbf{F_a}^{5+}$, but with modified stoichiometry: $\mathbf{1_a}^{2+}$ (97.0 mg, 0.188 mmol), $\mathbf{2_a}^{3+}$ (101.0 mg, 0.125 mmol); yielding a yellowish solid (12.8 mg, 19%).

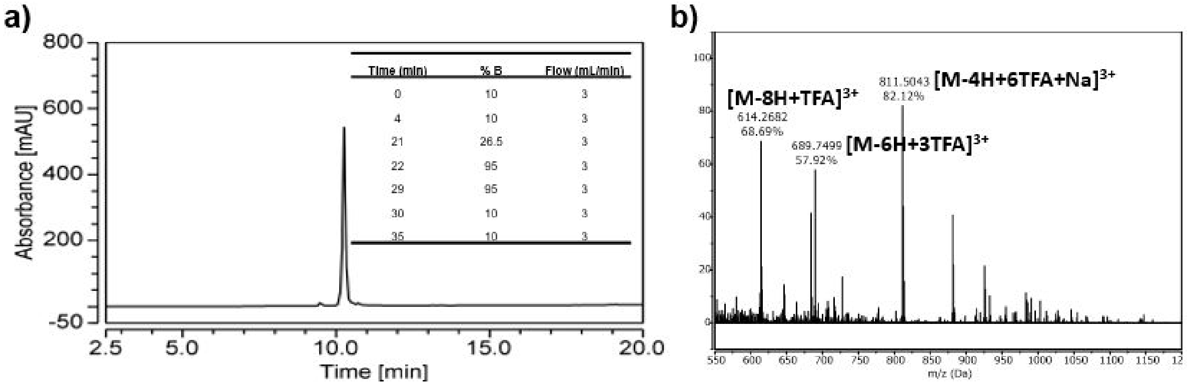

Figure S46: a) HPLC chromatogram (220 nm) of purified D^{12+} at t_R = 10.3 min (Inset: purification method). b) MS spectrum from the chromatographic peak at t_R = 10.3 min.

^{1}H NMR (D_2O, 500 MHz) δ = 8.82 (d, J = 6.5 Hz, 2H), 8.68 (d, J = 6.5 Hz, 4H), 8.46 (d, J = 6.7 Hz, 2H), 8.40 (s, 1H), 8.28 (d, J = 6.4 Hz, 3H), 8.23 (s, 1H), 8.19 (s, 2H), 8.16 (d, J = 6.5 Hz,

4H), 8.05 (d, J = 7.0 Hz, 2H), 7.85 (s, 1H), 7.68 (d, J = 4.9 Hz, 2H), 7.58 (s, 4H), 7.54 (s, 2H), 7.51 (s, 2H), 7.42 (s, 1H), 7.14 (d, J = 4.8 Hz, 3H), 5.77 (m, 6H), 5.49 (s, 2H), 5.44 (s, 4H). $^{13}C\{^1H\}$-NMR (D_2O, 125 MHz): δ = 154.5 (C), 154.5 (C), 149.8 (C), 149.8 (C), 144.5 (CH), 144.5 (CH), 143.8 (CH), 141.4 (CH), 140.9 (CH), 136.4 (C), 136.1 (C), 134.5 (C), 134.2 (C), 131.0 (CH), 130.2 (CH), 129.9 (CH), 129.8 (CH), 124.9 (CH), 124.7 (CH), 110.3 (CH), 109.7 (CH), 63.5 (CH_2), 63.4 (CH_2), 61.0 (CH_2), 60.9 (CH_2). HRMS (ESI): m/z calculated for $C_{108}H_{96}N_{24}^{6+}$ $[M\text{-}6H]^{6+}$ 288.3042, found 288.3041; m/z calculated for fragment $C_{58}H_{53}N_{12}^{\cdot 3+}$ $[M'\text{-}3H+e]^{\cdot 3+}$ 305.1448, found 305.1448; m/z calculated for fragment $C_{70}H_{62}N_{16}Na_4^{\cdot 3+}$ $[M'\text{-}5H+4Na+4e]^{\cdot 3+}$ 406.1639, found 406.1898.

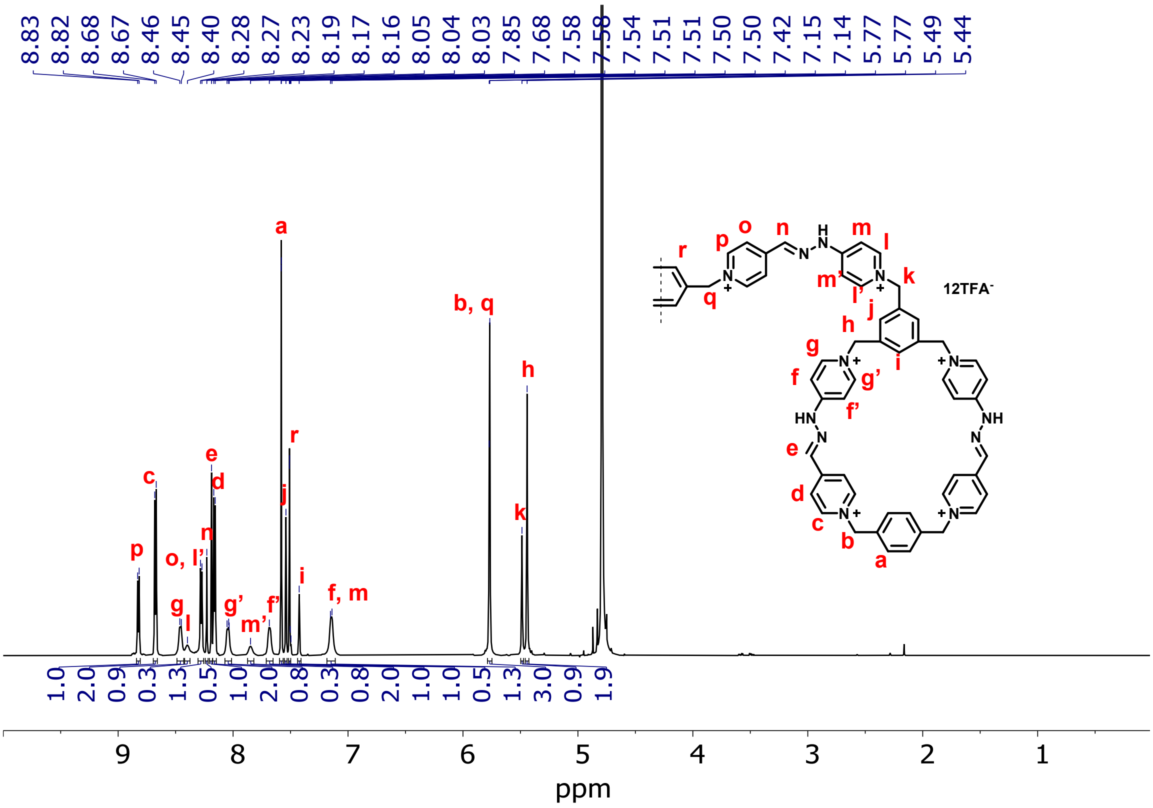

Figure S47: ^{1}H-NMR (500 MHz, D_2O) spectrum of D^{12+}.

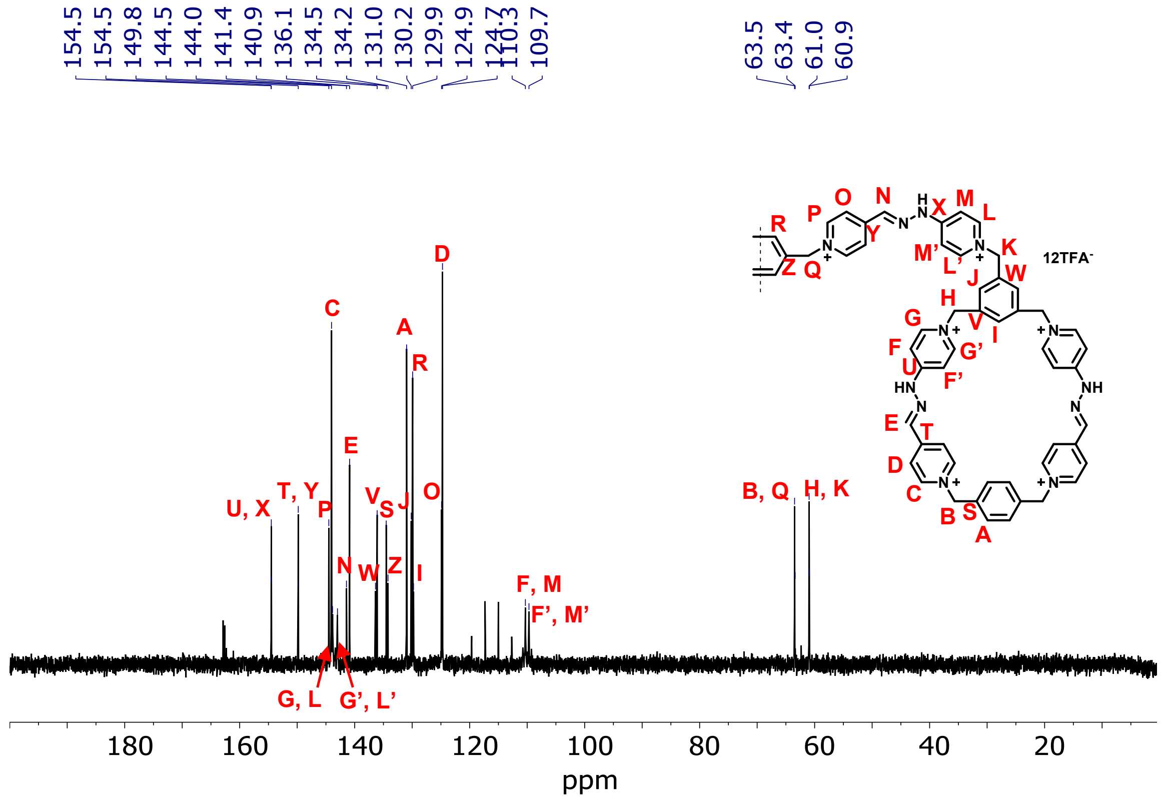

Figure S48: $^{13}C\{^1H\}$-NMR (126 MHz, D_2O) spectrum of D^{12+}.

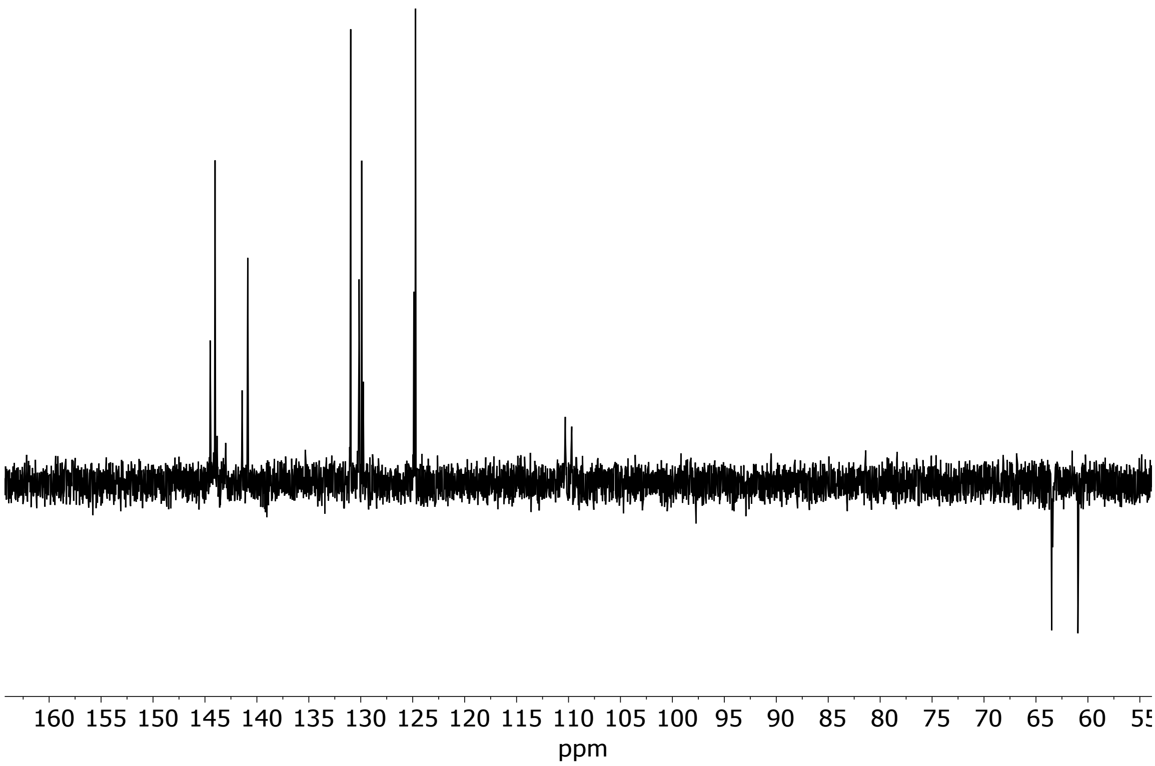

Figure S49: DEPT-135 (126 MHz, D_2O) spectrum of D^{12+}.

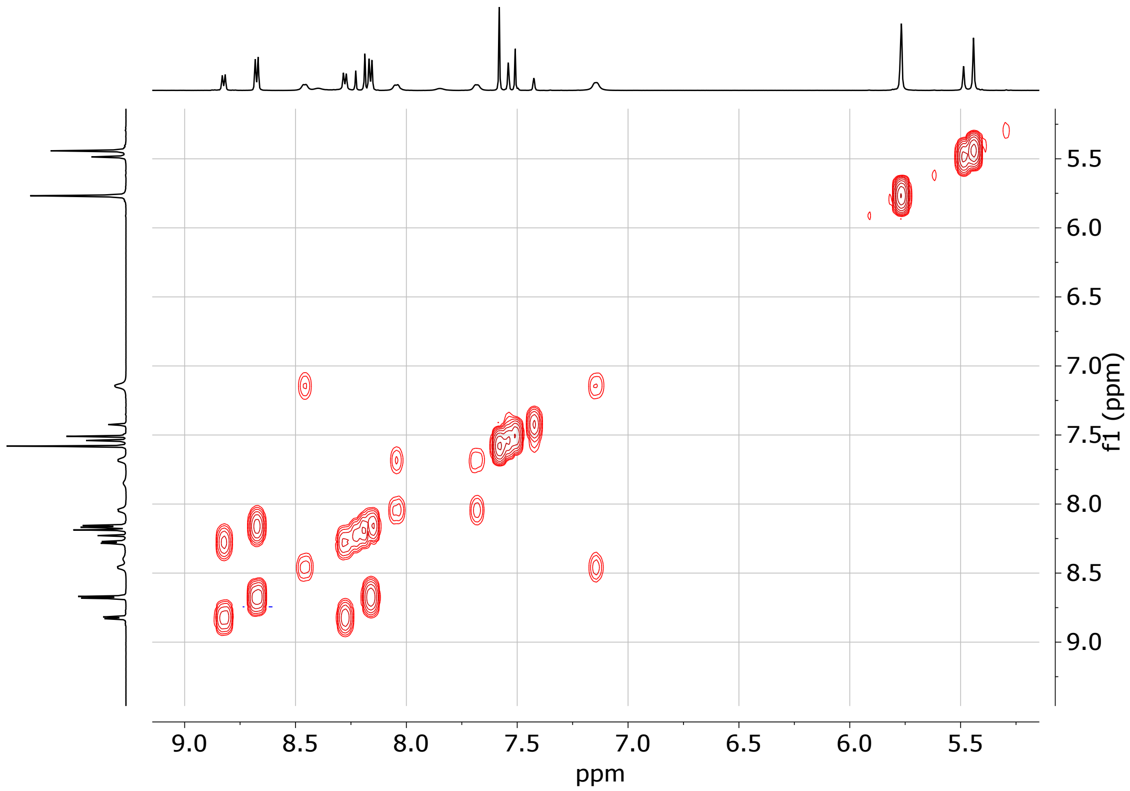

Figure S50: ^1H-^1H COSY (500 MHz, D_2O) spectrum of D^{12+}.

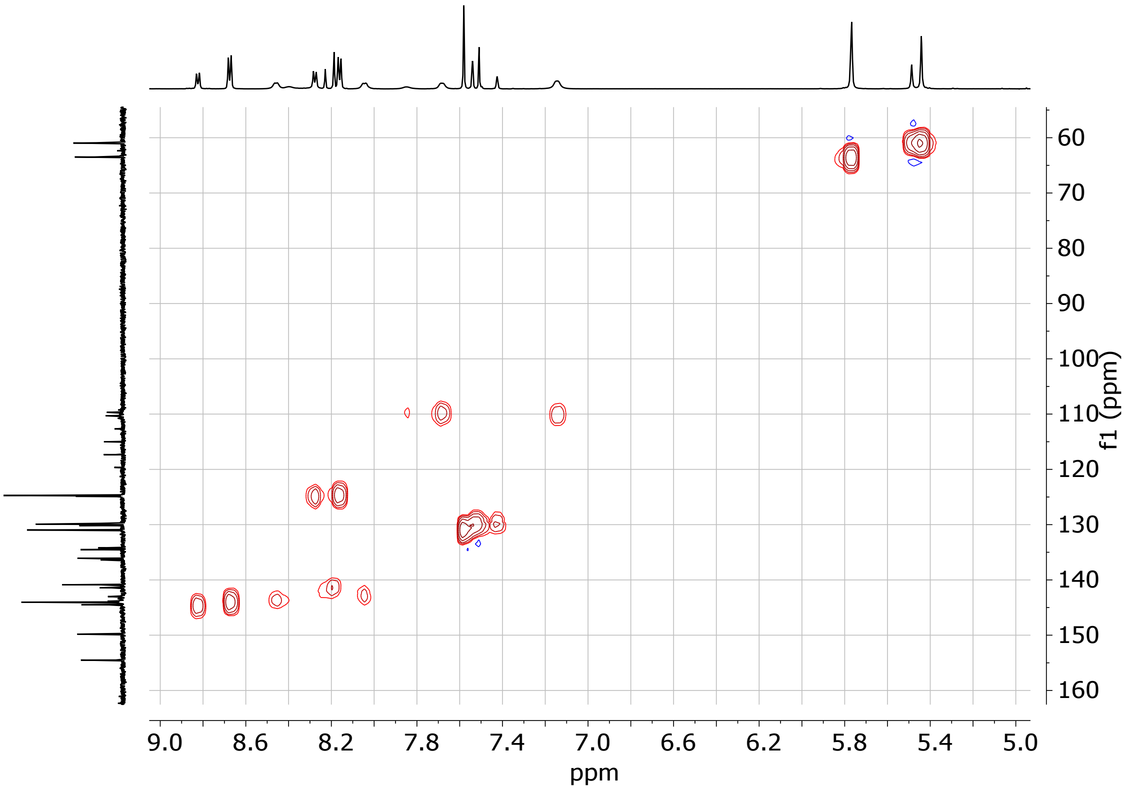

Figure S51: ^1H-$^{13}C\{^1H\}$ HSQC (500 and 126 MHz, D_2O) spectrum of D^{12+}.

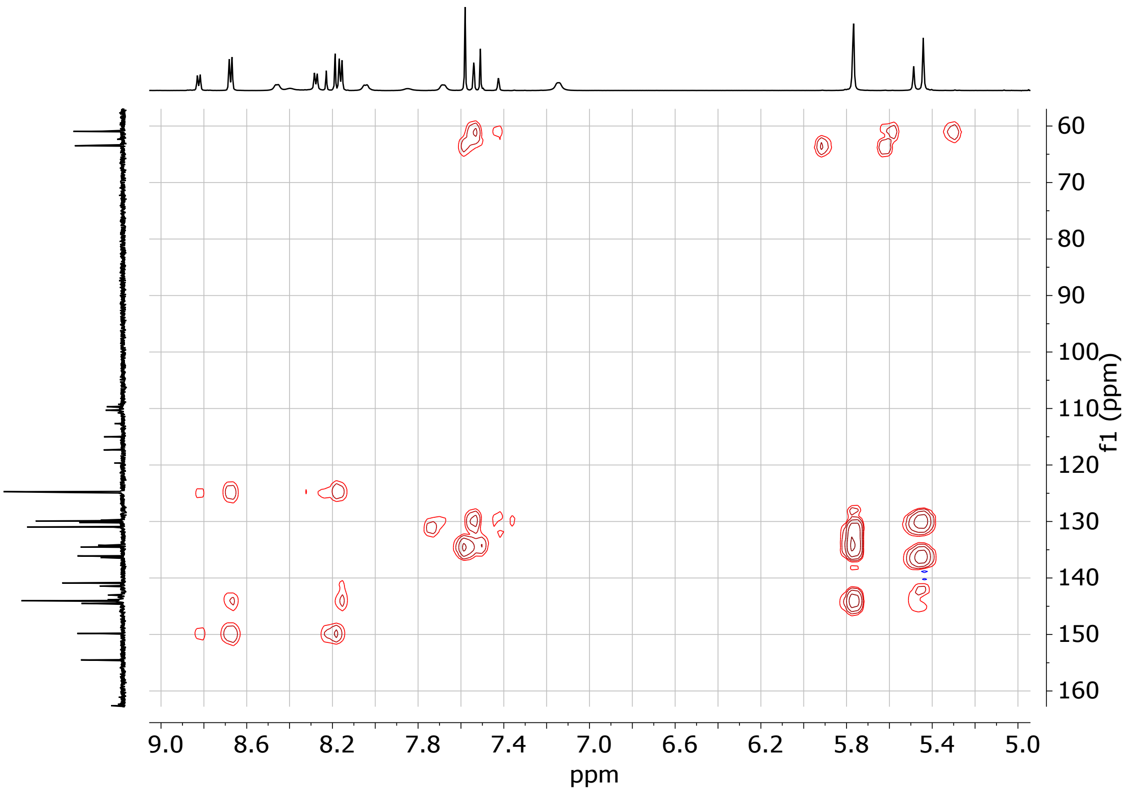

Figure S52: ^1H-$^{13}C\{^1H\}$ HMBC (500 and 126 MHz, D_2O) spectrum of D^{12+}.

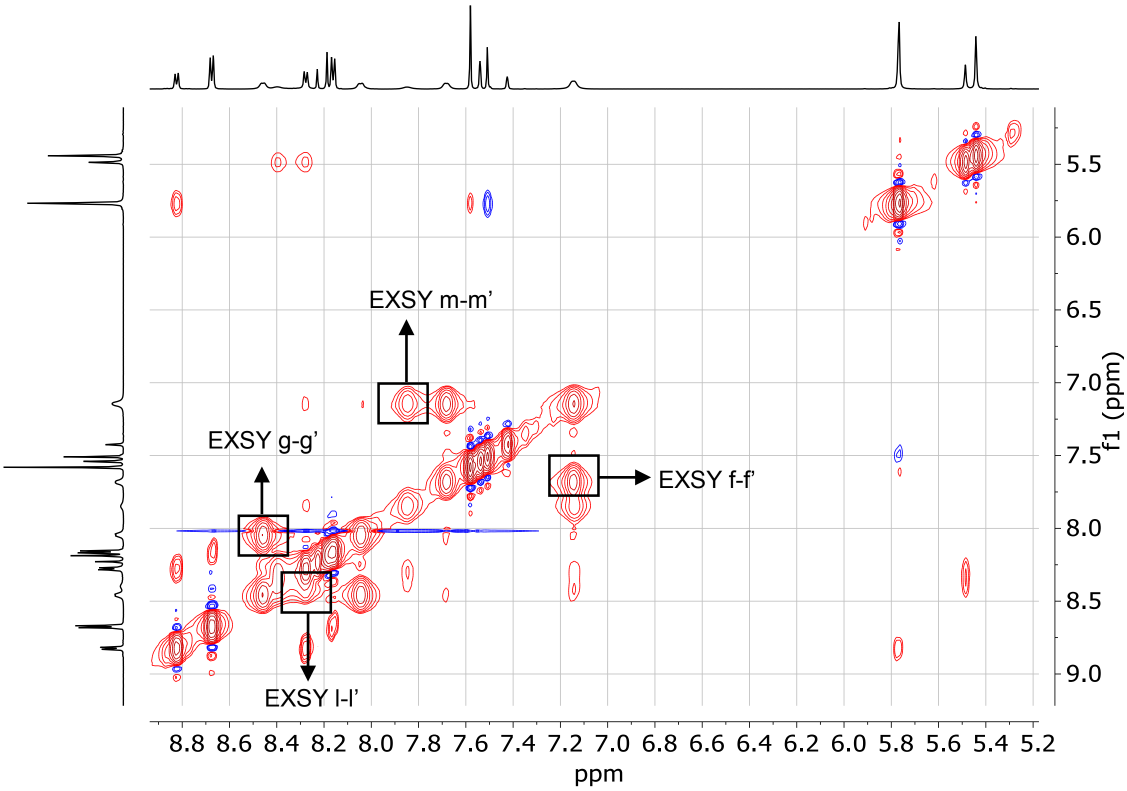

Figure S53: ^1H-^1H NOESY (500 MHz, D_2O) spectrum of D^{12+}.

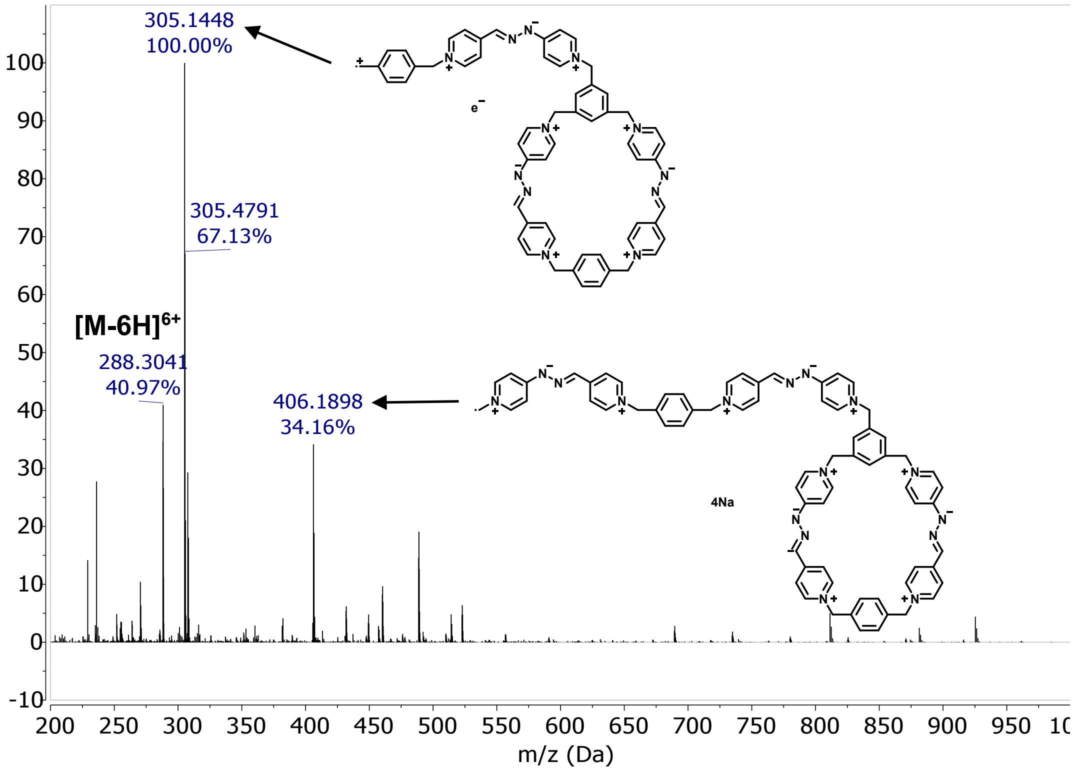

Figure S54: ESI-HRMS of D^{12+}.

2.6. General synthesis and NMR characterization of the inclusion complexes.

Equimolar 2.5 mM solutions of macrocycles $\mathbf{F_{a/b}}^{5+}$ and **Compound 4** were prepared in D_2O at room temperature and the corresponding NMR was recorded immediately after.

2.6.1. NMR characterization of $4{\subset}F_a^{5+}$.

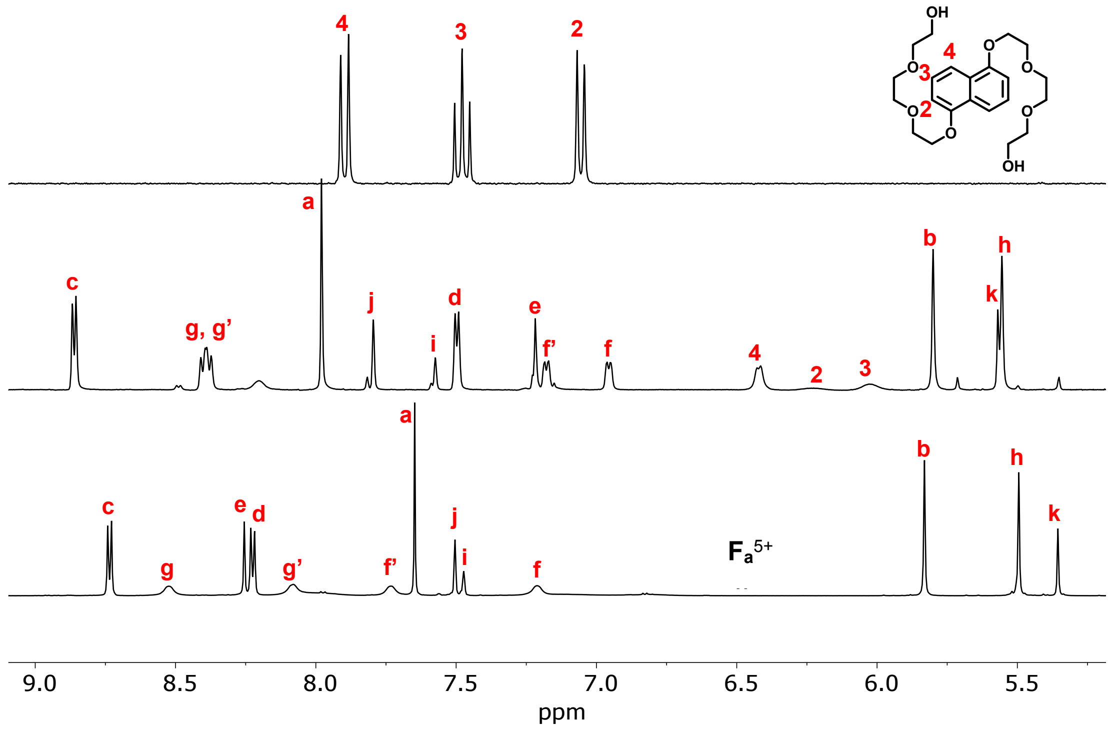

Figure S55: ^{1}**H-NMR spectra comparison between the guest 4 (top), the macrocyclic host F_a^{5+} (bottom) and the 1:1 inclusion complex $4{\subset}F_a^{5+}$.**

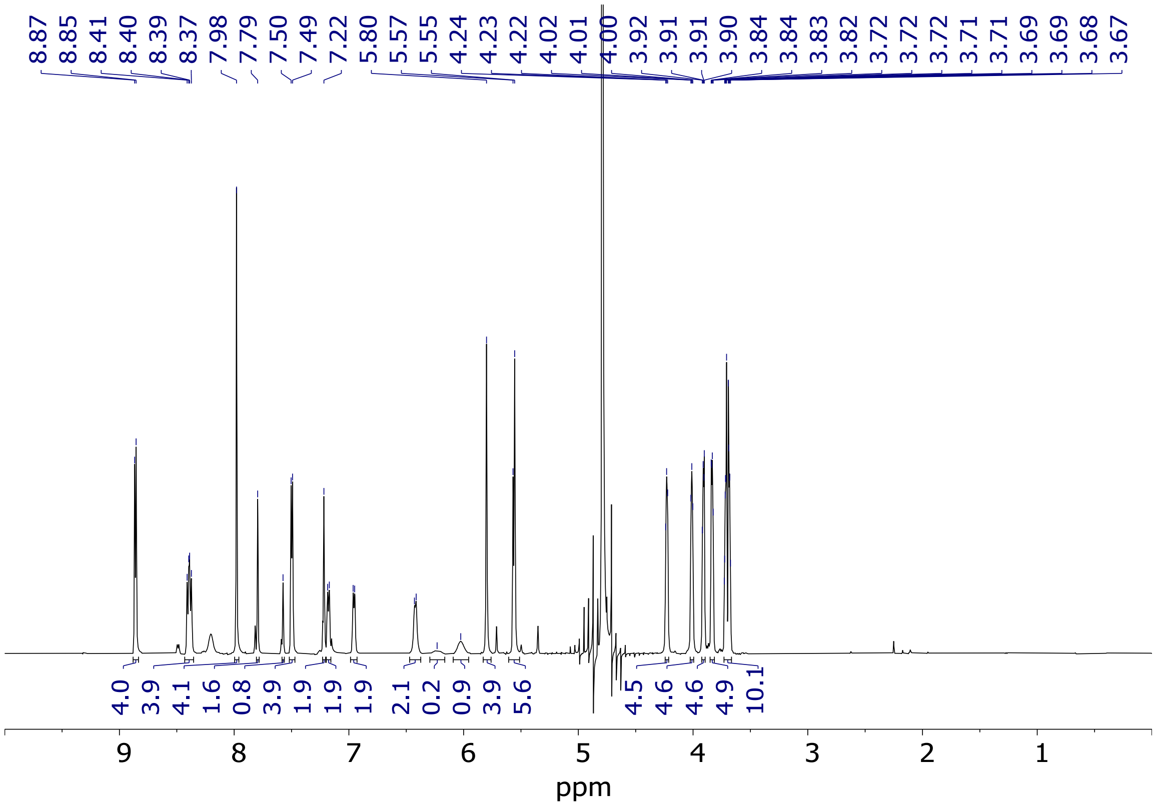

Figure S56: ^{1}H-NMR (500 MHz, D_2O) spectrum of $4\subset F_a^{5+}$.

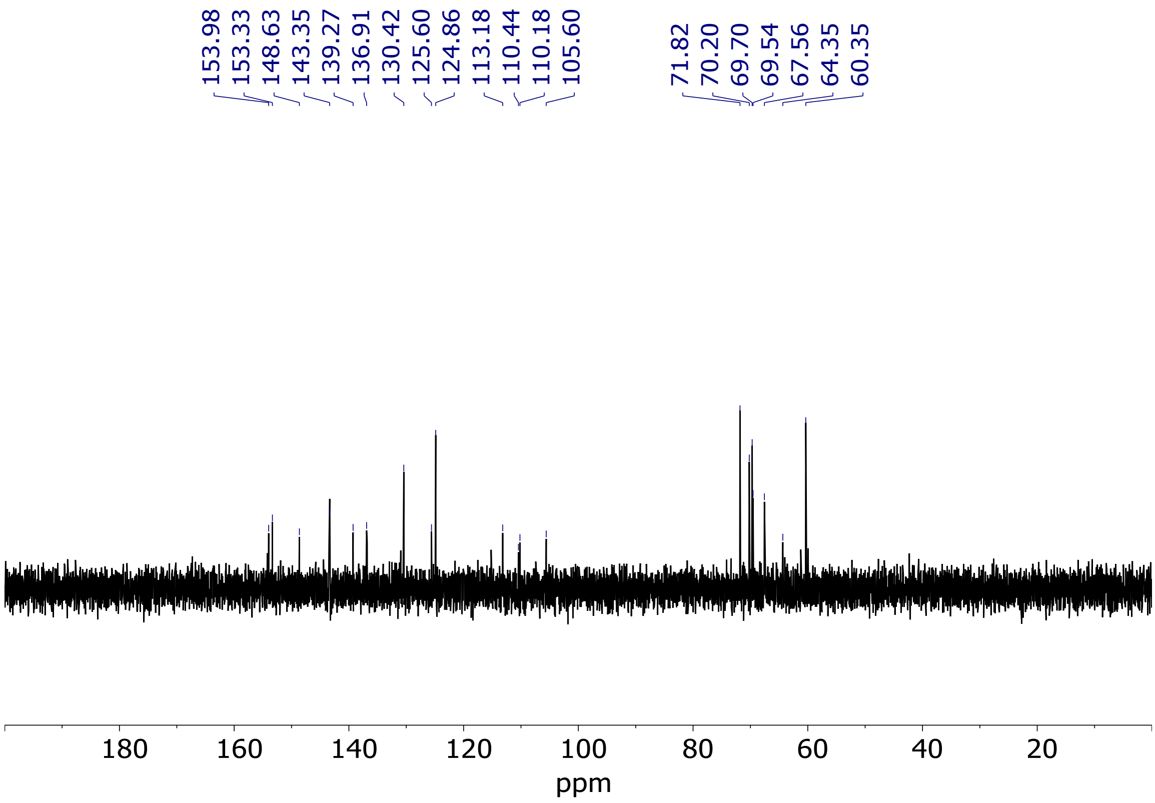

Figure S57: $^{13}C\{^{1}H\}$-NMR (126 MHz, D_2O) spectrum of $4\subset F_a^{5+}$.

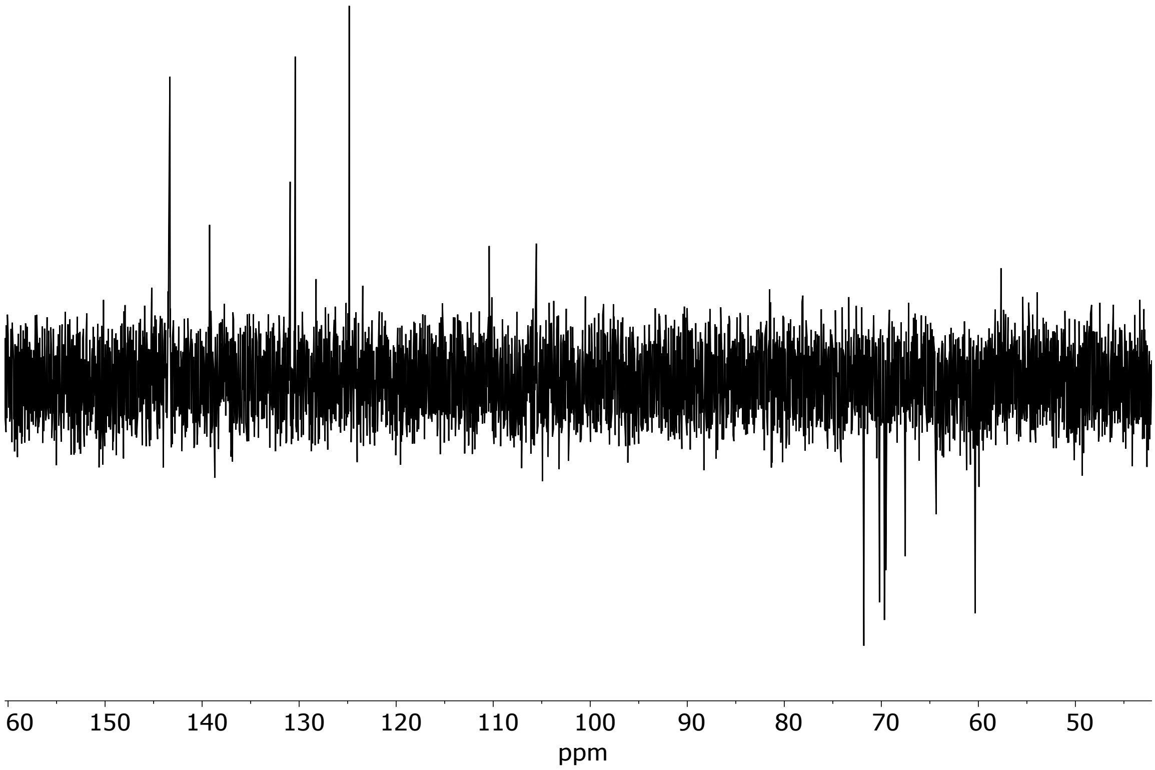

Figure S58: DEPT-135 (126 MHz, D_2O) spectrum of 4⊂F_a^{5+}.

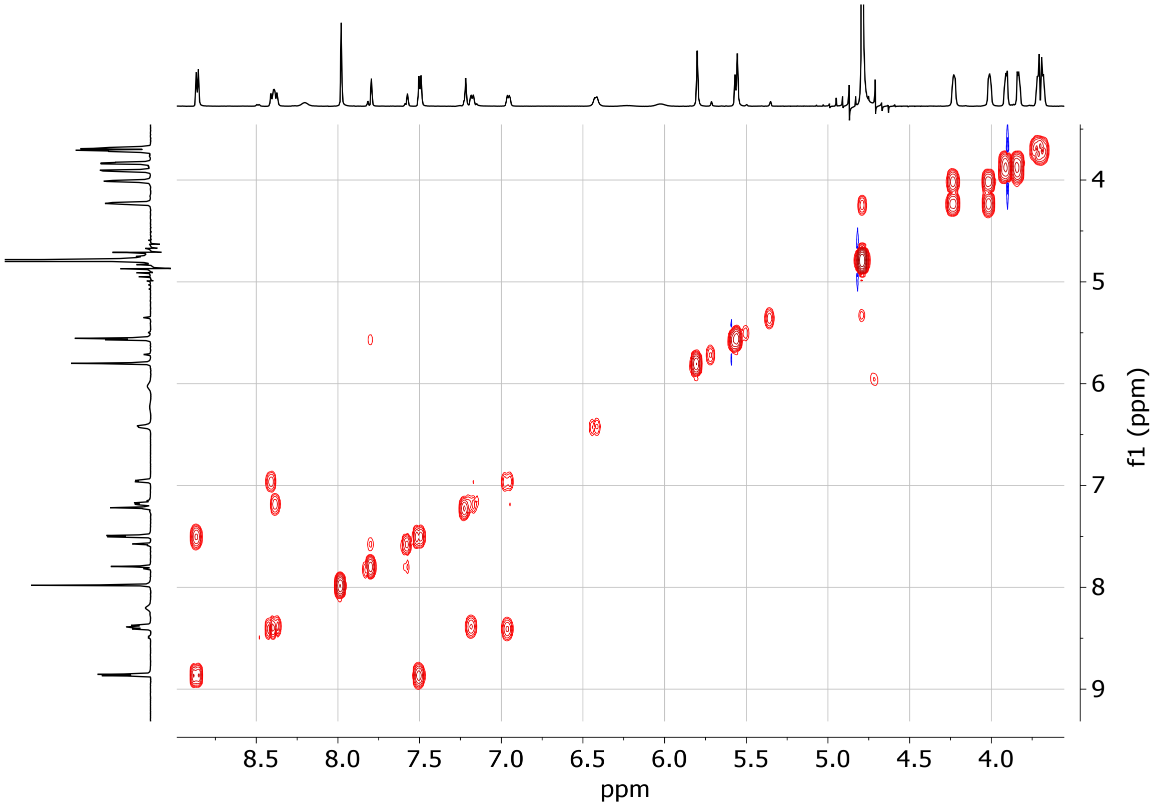

Figure S59: ^{1}H-^{1}H COSY (500 MHz, D_2O) spectrum of 4⊂F_a^{5+}.

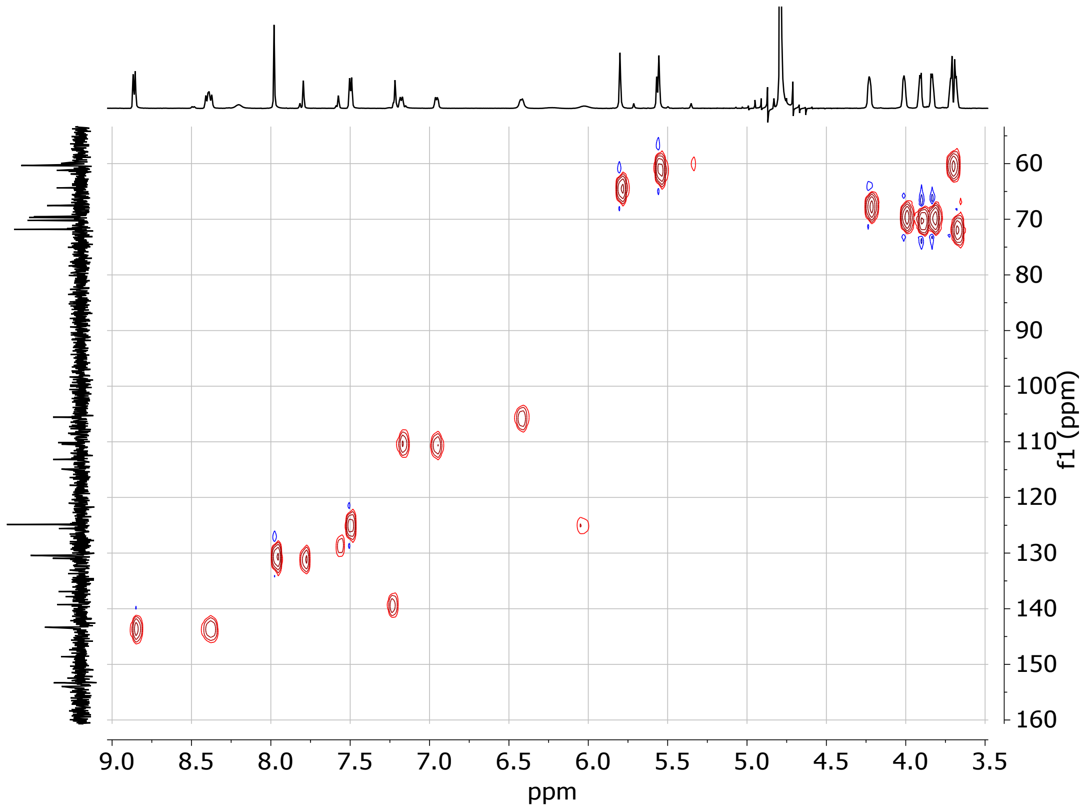

Figure S60: ^{1}H-$^{13}C\{^{1}H\}$ HSQC (500 and 126 MHz, D_2O) spectrum of $4\subset F_a^{5+}$.

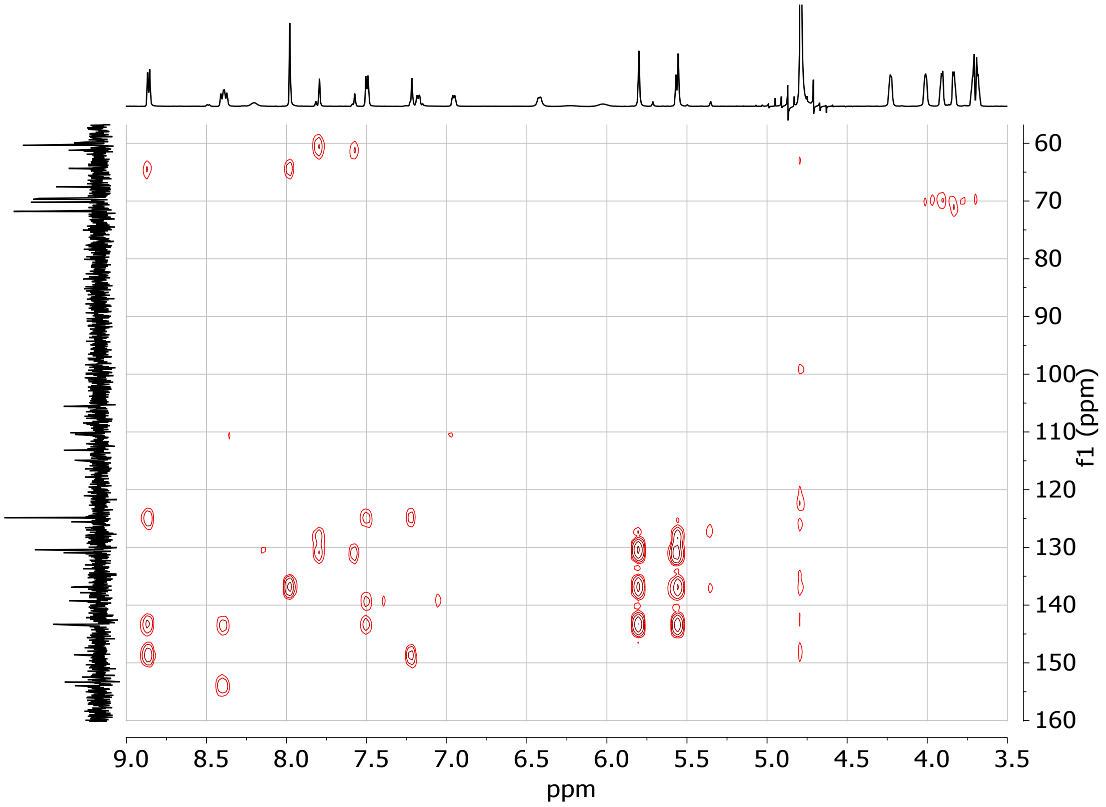

Figure S61: ^{1}H-$^{13}C\{^{1}H\}$ HMBC (500 and 126 MHz, D_2O) spectrum of $4\subset F_a^{5+}$.

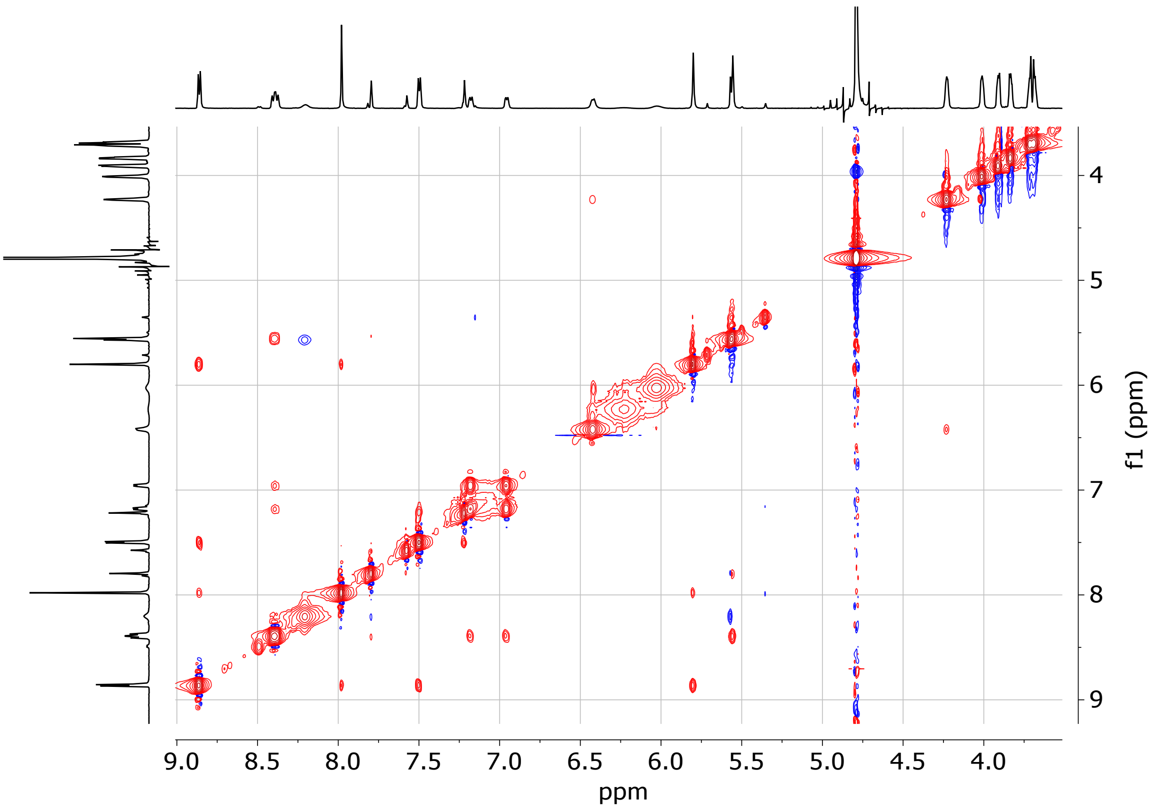

Figure S62: ^{1}H-^{1}H NOESY (500 MHz, D_2O) spectrum of 4⊂F_a^{5+}.

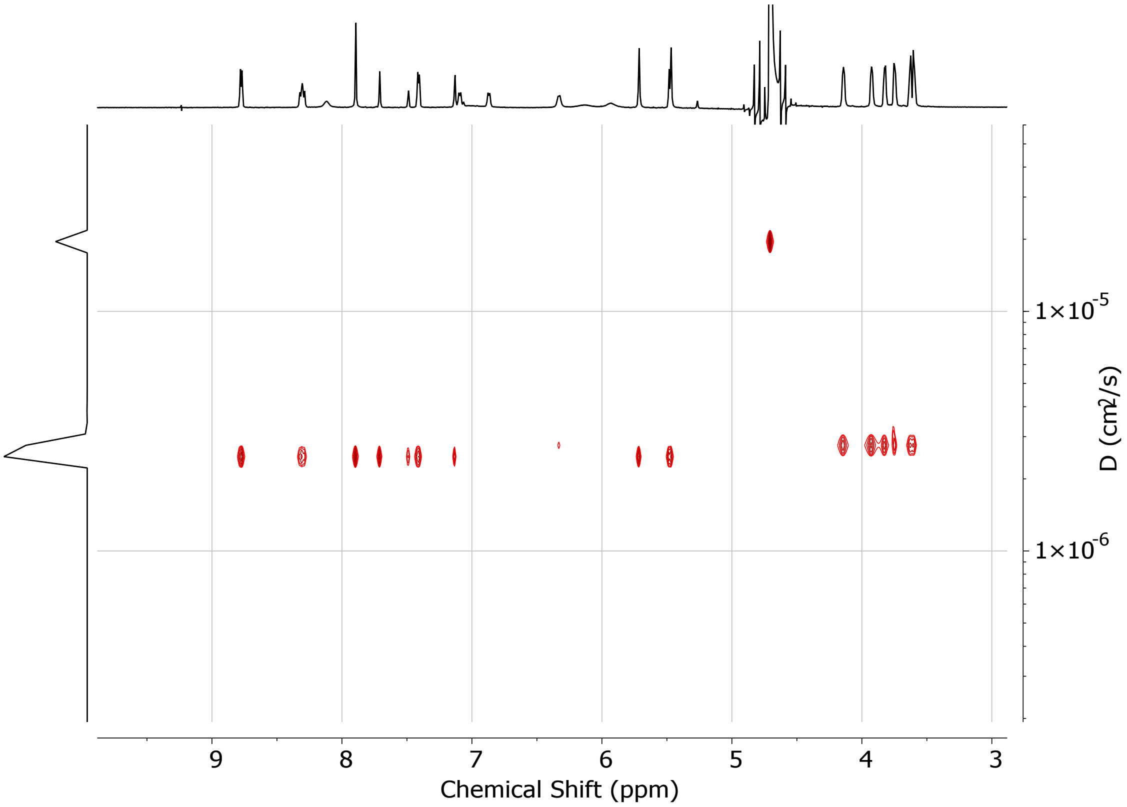

Figure S63: ^{1}H-DOSY spectrum of the 1:1 mixture of F_a^{5+} and 4.

2.6.2. NMR characterization of 4⊂F_b^{5+}.

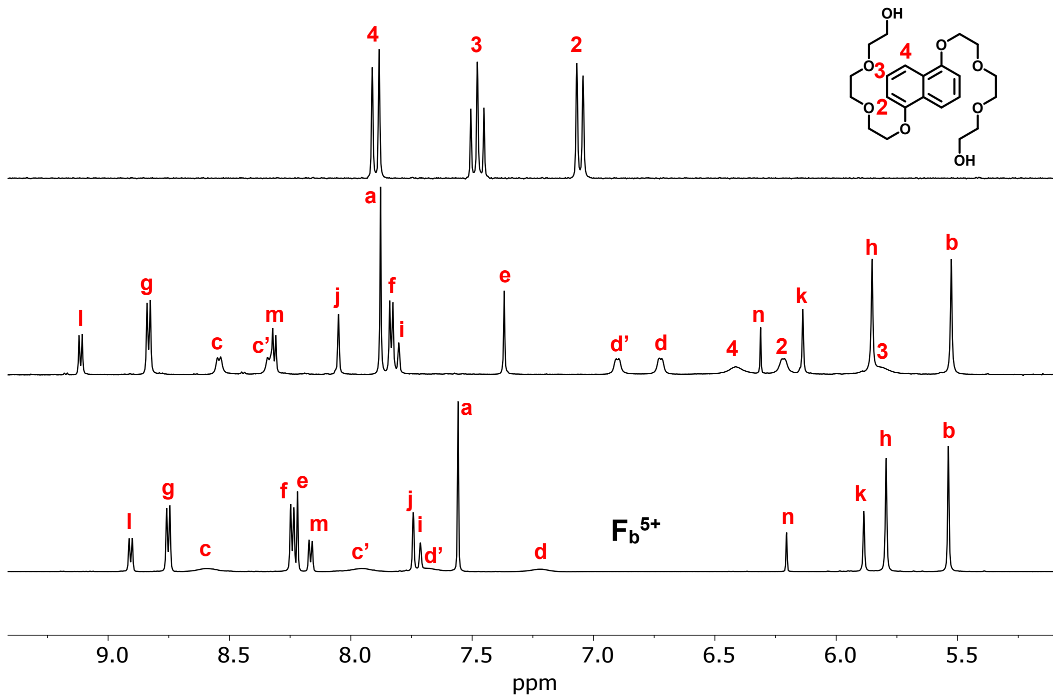

Figure S64: ^{1}H-NMR spectra comparison between the guest 4 (top), the macrocyclic host F_b^{5+} (bottom) and the 1:1 inclusion complex 4⊂F_b^{5+}.

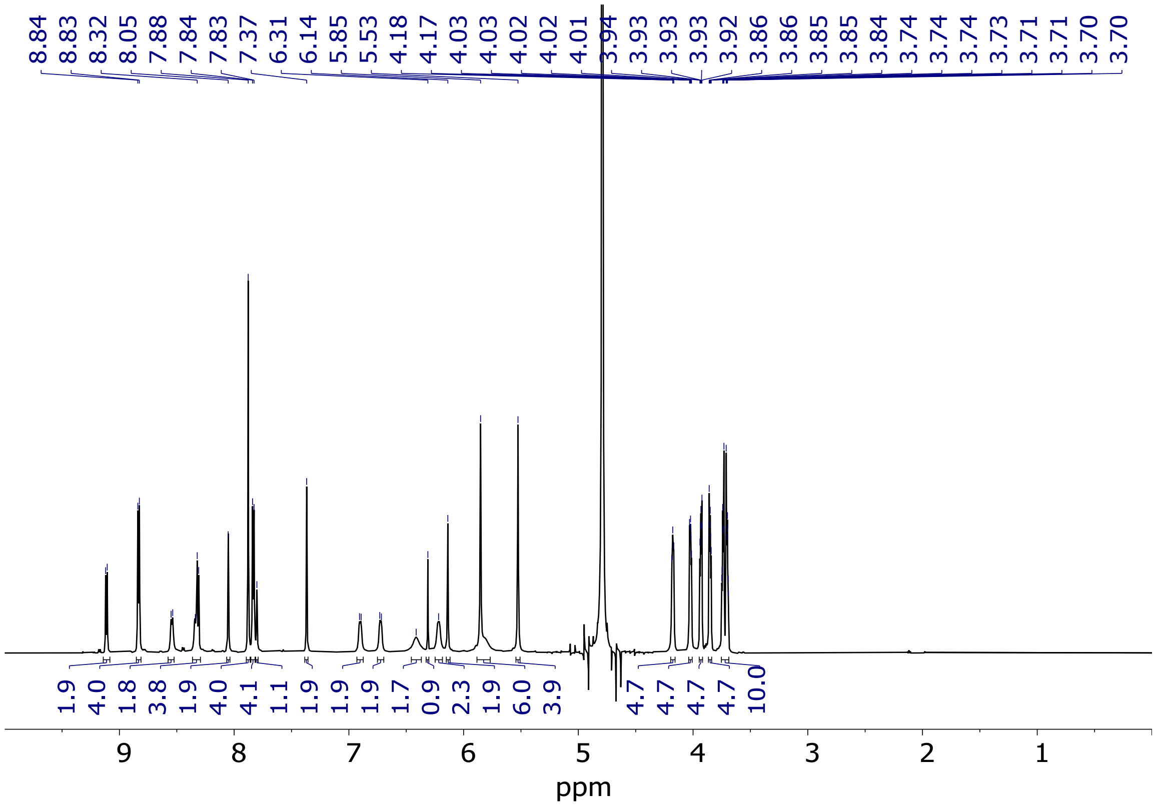

Figure S65: ^{1}H-NMR (500 MHz, D_2O) spectrum of 4⊂F_b^{5+}.

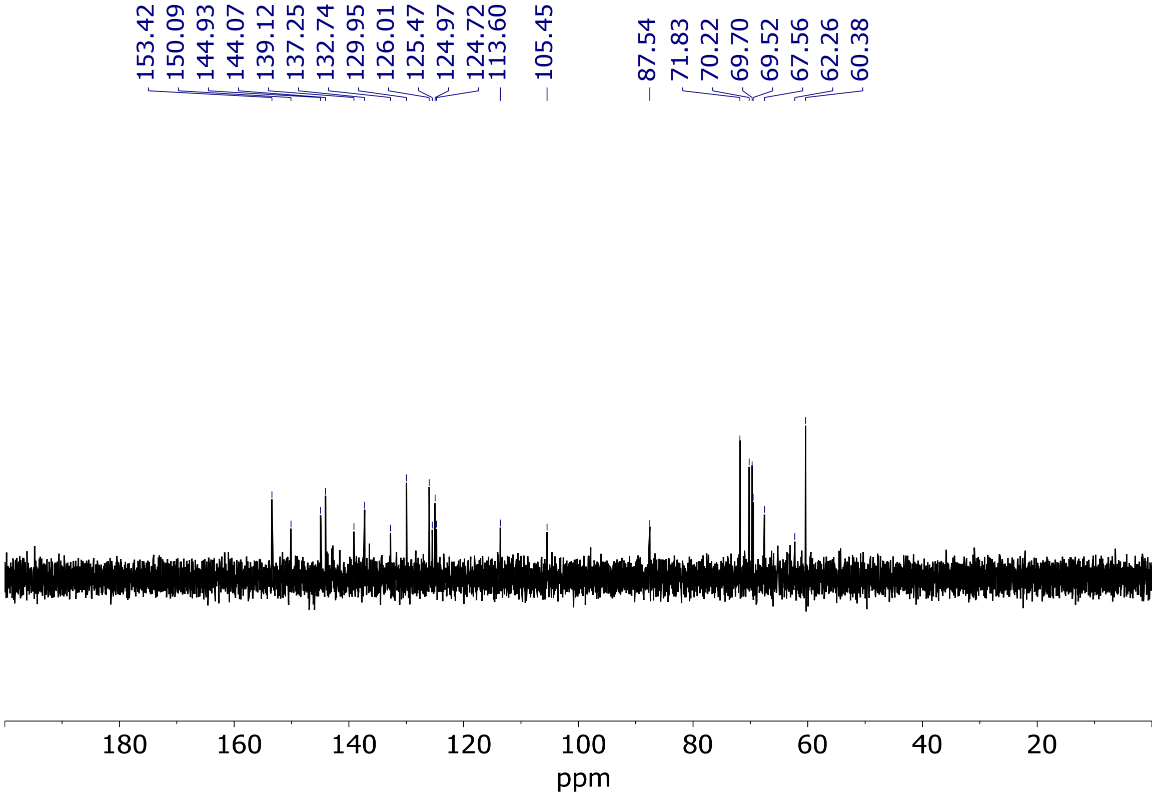

Figure S66: $^{13}C\{^{1}H\}$-NMR (126 MHz, D_2O) spectrum of 4⊂F_b^{5+}.

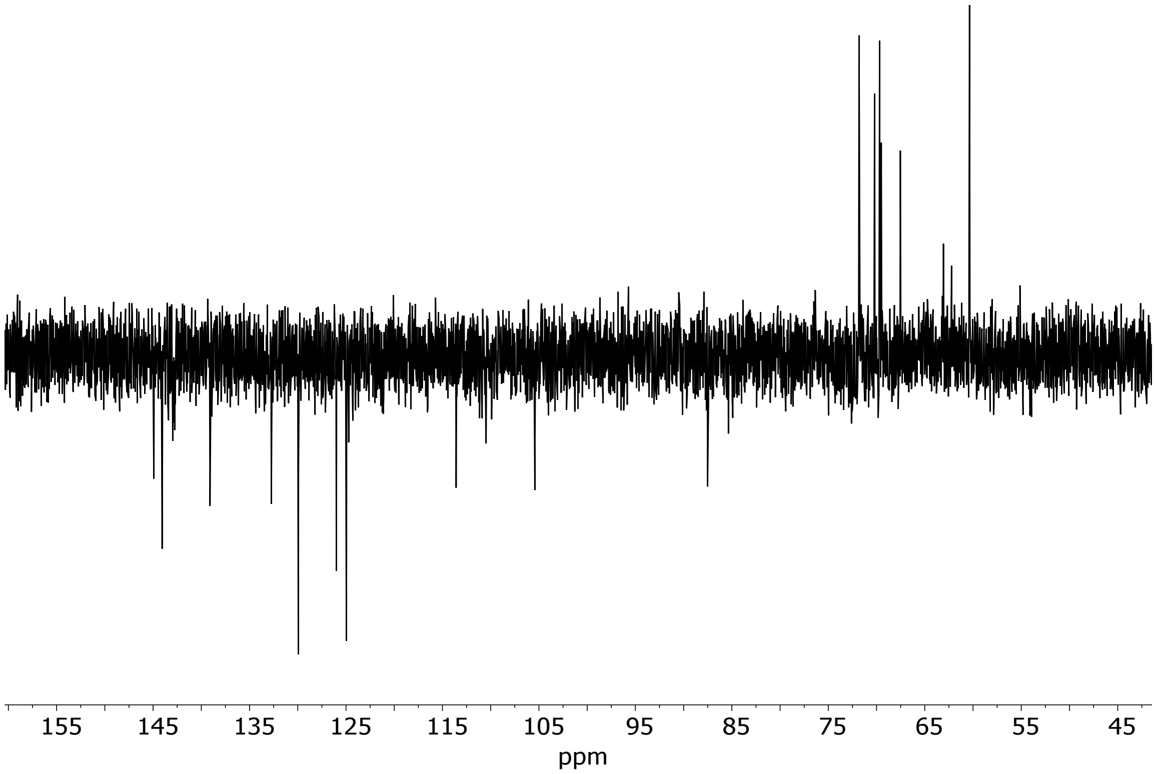

Figure S67: DEPT-135 (126 MHz, D_2O) spectrum of 4⊂F_b^{5+}.

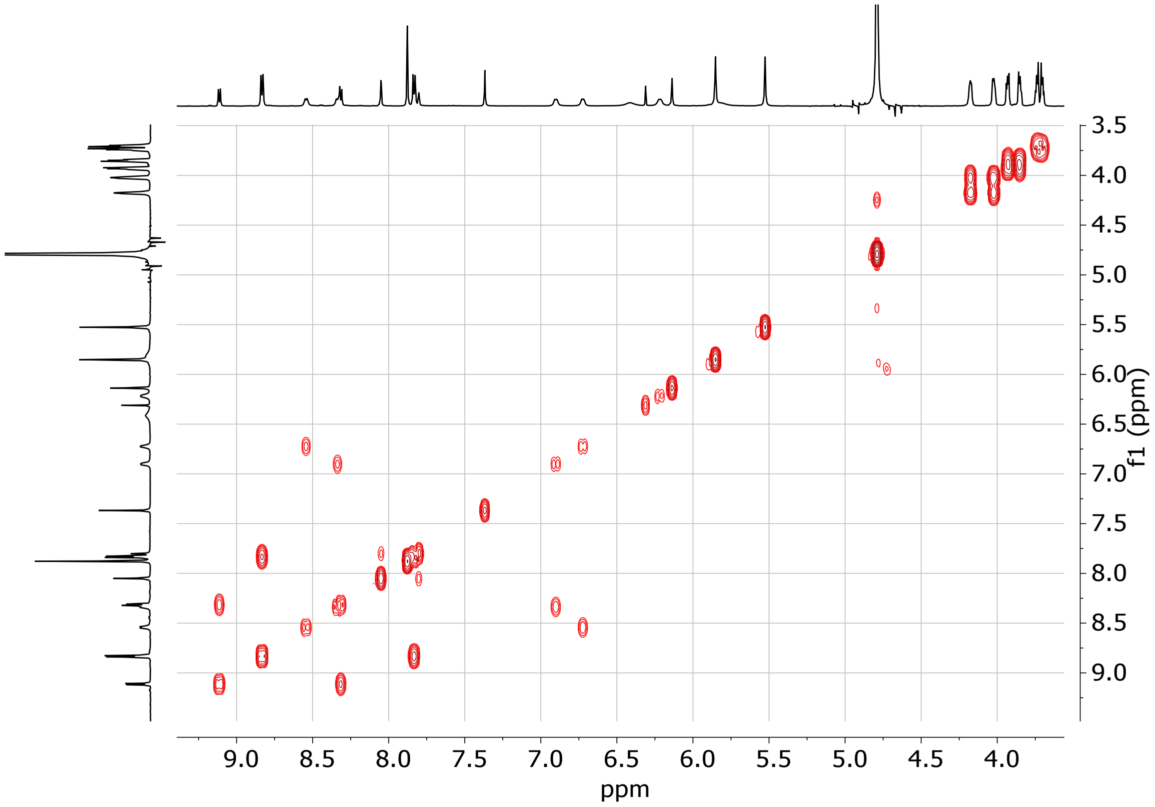

Figure S68: ^{1}H-^{1}H COSY (500 MHz, D_2O) spectrum of 4⊂F_b^{5+}.

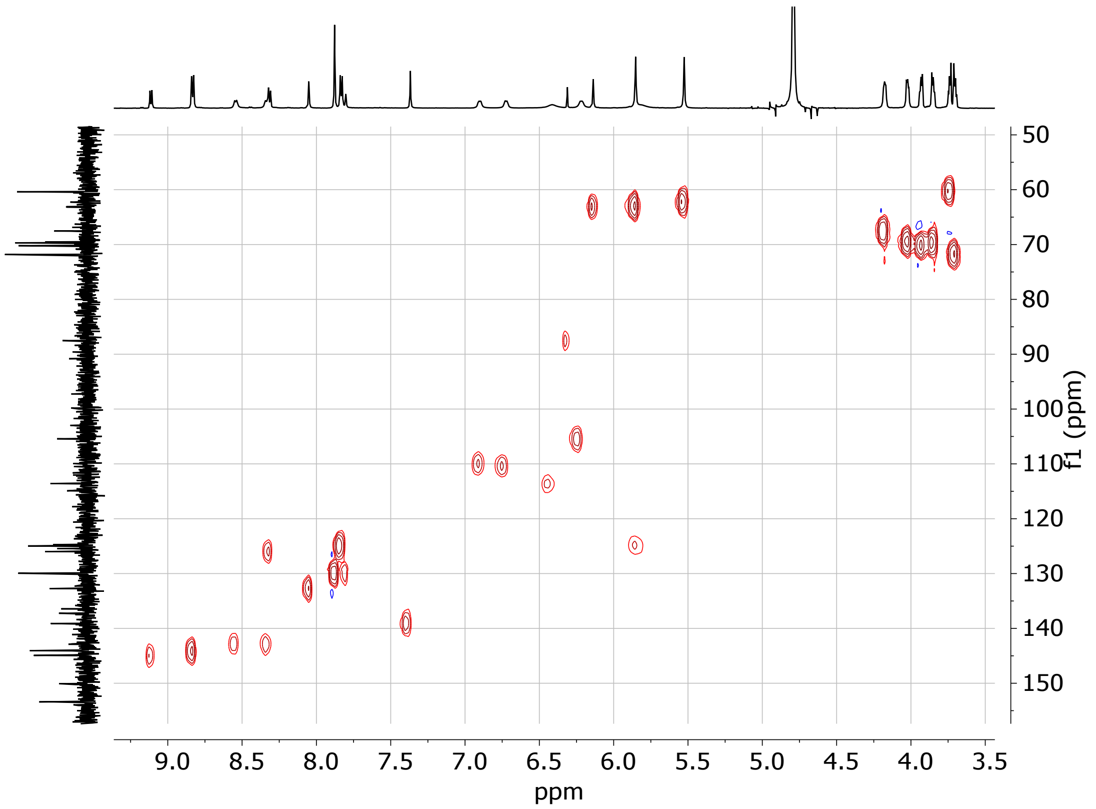

Figure S69: ¹H-¹³C{¹H} HSQC (500 and 126 MHz, D_2O) spectrum of 4⊂F_b^{5+}.

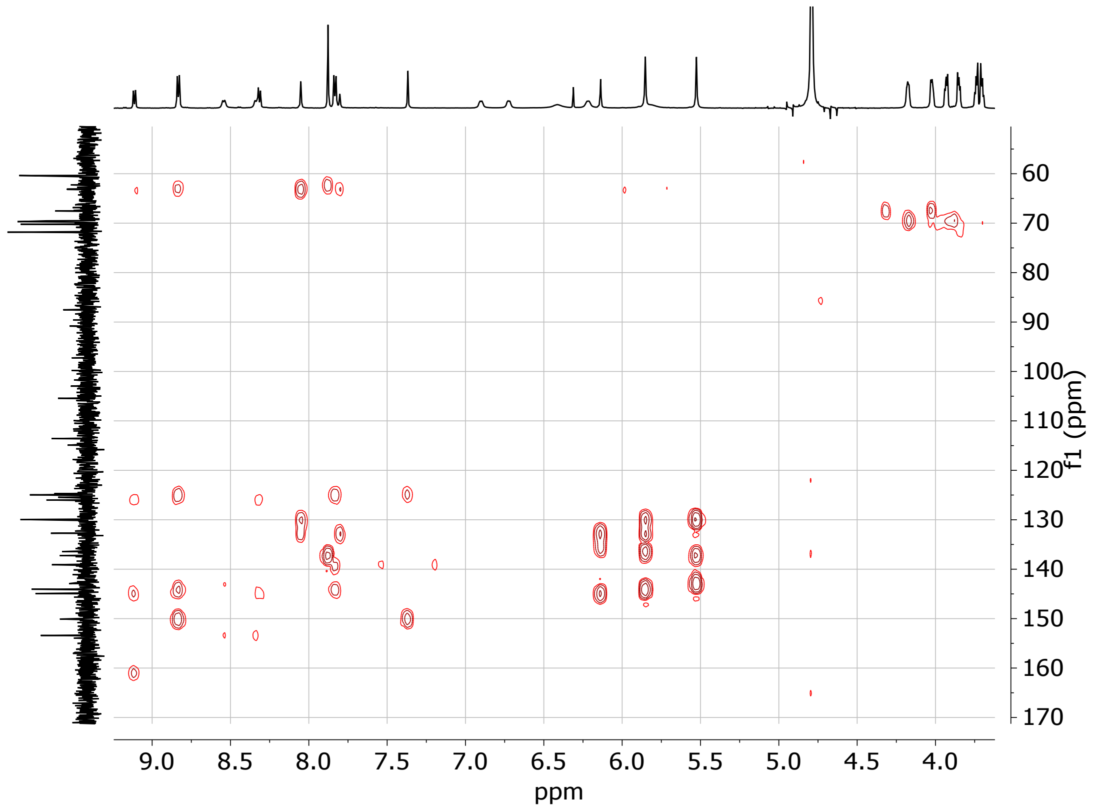

Figure S70: ¹H-¹³C{¹H} HMBC (500 and 126 MHz, D_2O) spectrum of 4⊂F_b^{5+}.

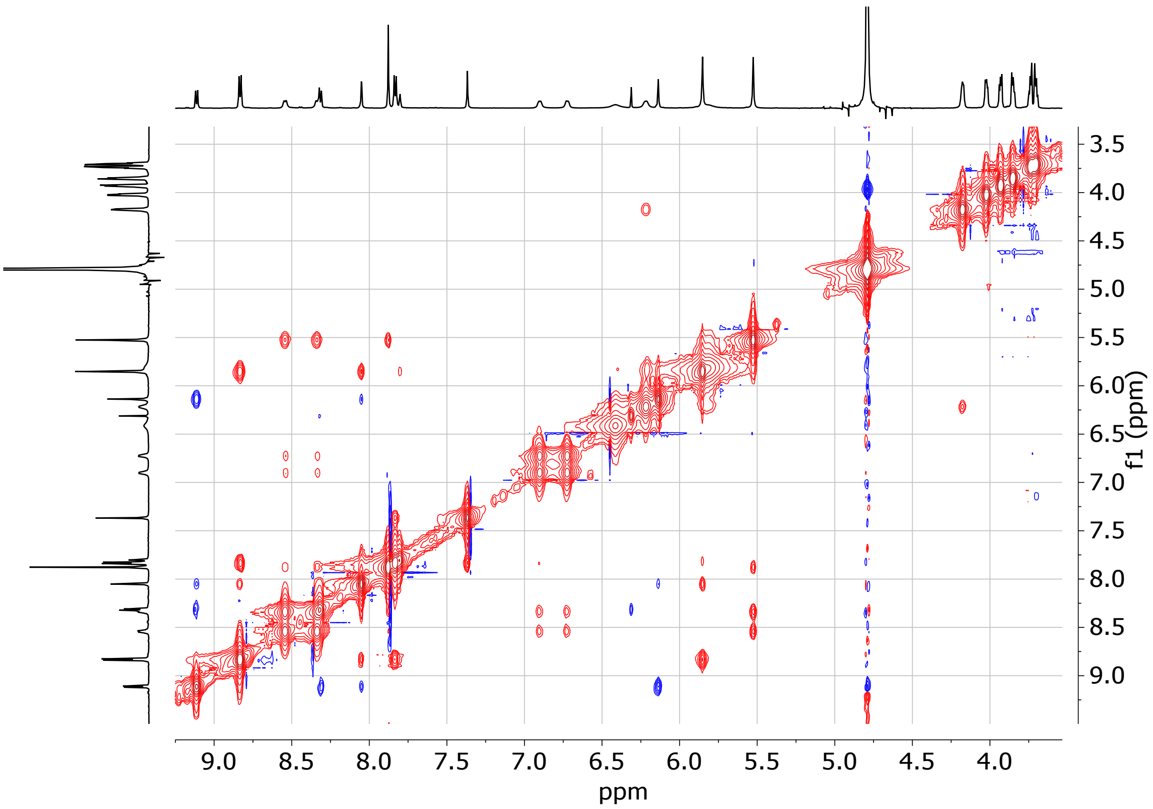

Figure S71: ^{1}H-^{1}H NOESY (500 MHz, D_2O) spectrum of $4 \subset F_b^{5+}$.

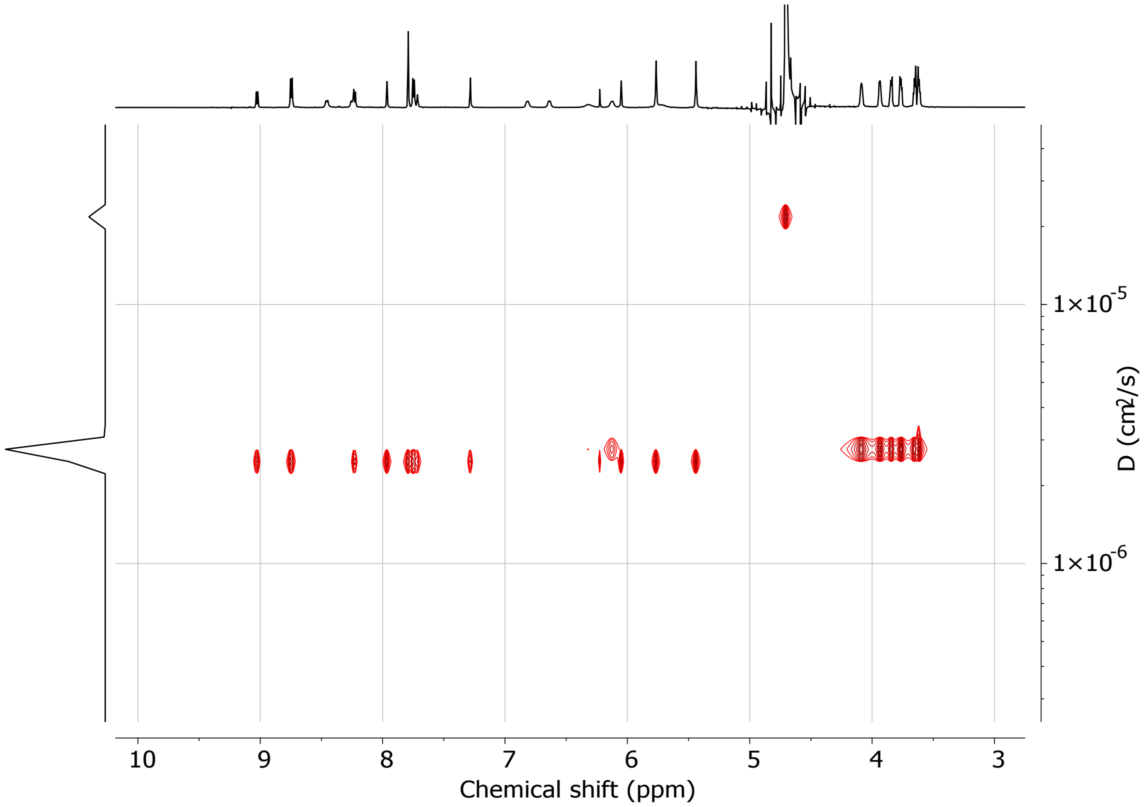

Figure S72: ^{1}H-DOSY spectrum of the 1:1 mixture of F_b^{5+} and 4.

2.7. ^{1}H NMR titration experiments: determination of K_a values.

2.7.1. $4\subset F_a^{5+}$ at pD = 5.

To carry out the titration, mixtures of $\mathbf{F_a}^{5+}$ and 4 of different proportions were prepared from appropriate stocks solutions in D_2O adjusted to pD = 5 with TFA-d.

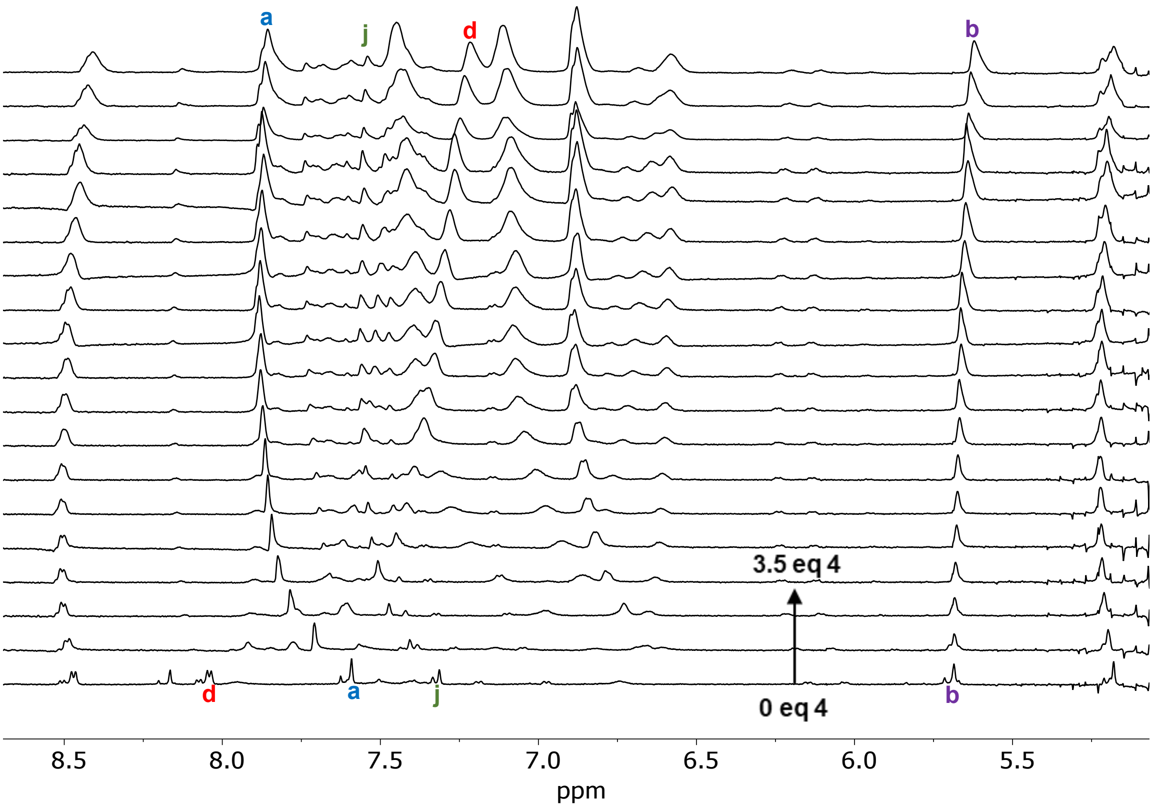

Figure S73: ^{1}H-NMR (500 MHz, D_2O) spectra of F_a^{5+} (1 mM) upon titration with 4 (10 mM) at pD = 5. The chemical shift of signals Hd, Ha, Hj and Hb were used for the fitting.[4]

The mechanism proposed for the fitting process equilibria, and introduced on the software *Dynafit* was the following:[5] $\mathbf{F_a}^{5+}$ + **4** ⟺ $\mathbf{4\subset F_a}^{5+}$.

[4] The spectra were presented in a way the chemical shift changes could be clearly observed. The coalescence obtained upon the addition of **4** made this necessary. $\mathbf{F_a}^{5+}$ concentration was kept constant throughout the whole titration experiment.
[5] P. Kuzmic, *Anal. Biochem.* 1996, **237**, 260-273.

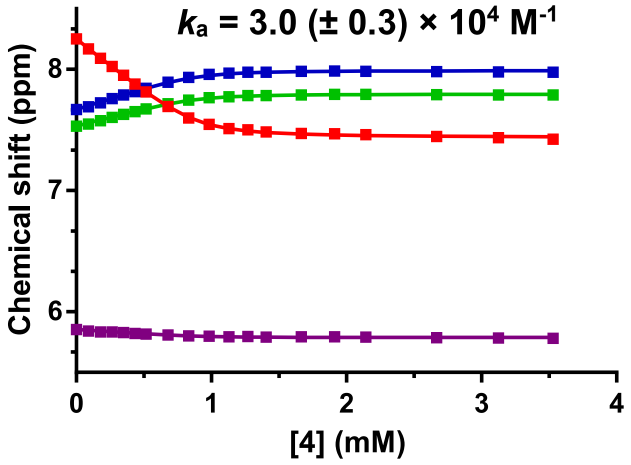

Figure S74: Fitting of experimental data (squares) for signals Hd, Ha, Hj and Hb.

2.7.2. $4 \subset F_a^{3+}$ at pD = 11.

To carry out the titration, mixtures of $\mathbf{F_a}^{5+}$ and **4** of different proportions were prepared from appropriate stocks solutions in D_2O adjusted to pD = 11 with NaOD.

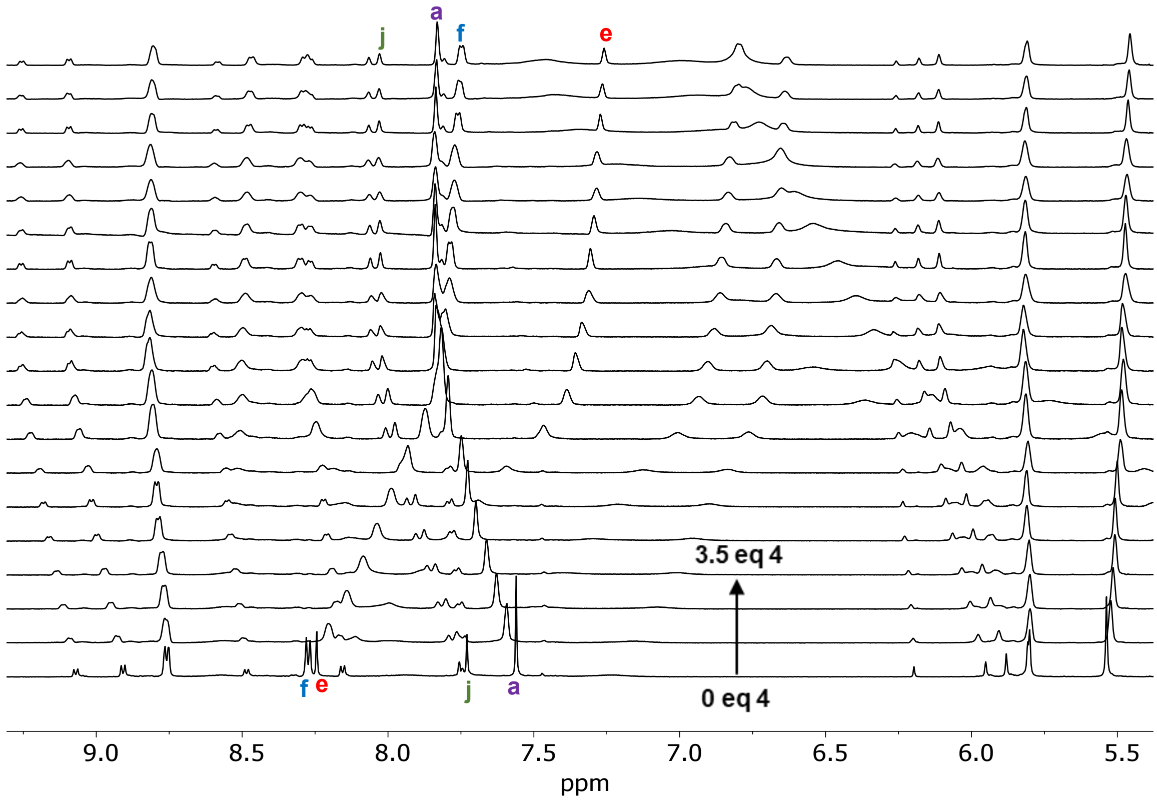

Figure S75: ^{1}H-NMR (500 MHz, D_2O) spectra of F_a^{3+} (1 mM) upon titration with 4 (10 mM) at pD = 11. The chemical shift of signals Hd, Ha, Hj and Hb were used for the fitting.

The mechanism proposed for the fitting process equilibria, and introduced on the software *Dynafit* was the following:[5] $\mathbf{F_a^{3+}}$ + **4** ⇐⇒ **4**⊂$\mathbf{F_a^{3+}}$.

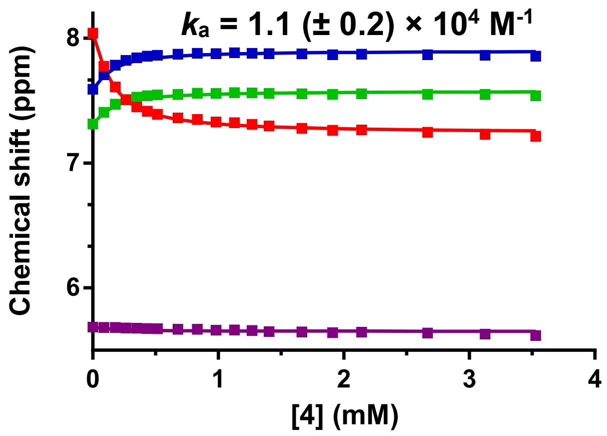

Figure S76: Fitting of experimental data (squares) for signals Hd, Ha, Hj and Hb.

2.7.3. 4⊂F_b^{5+} at pD = 5.

To carry out the titration, mixtures of $\mathbf{F_b^{5+}}$ and **4** of different proportions were prepared from appropriate stocks solutions in D_2O adjusted to pD = 5 with TFA-d.

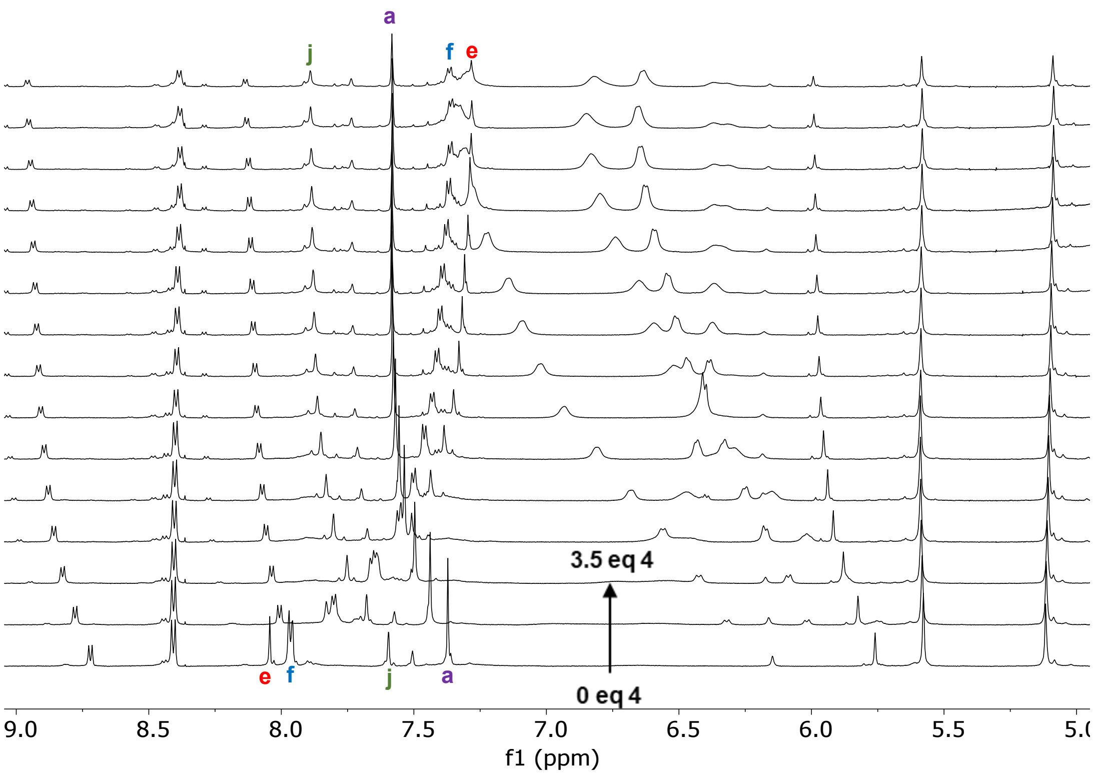

Figure S77: ^{1}H-NMR (500 MHz, D_2O) spectra of F_b^{5+} (1 mM) upon titration with 4 (10 mM) at pD = 11. The chemical shift of signals He, Hf, Hj and Ha were used for the fitting.

The mechanism proposed for the fitting process equilibria, and introduced on the software *Dynafit* was the following:[5] $\mathbf{F_b^{5+}}$ + **4** ⇐⇒ **4⊂$\mathbf{F_b^{5+}}$**.

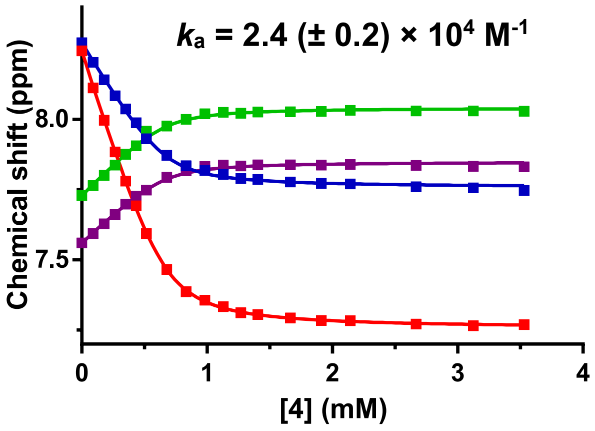

Figure S78: Fitting of experimental data (squares) for signals He, Hf, Hj and Ha.

2.7.4. 4⊂F_b^{3+} at pD = 11.

To carry out the titration, mixtures of $\mathbf{F_b^{5+}}$ and **4** of different proportions were prepared from appropriate stocks solutions in D_2O adjusted to pD = 11 with NaOD.

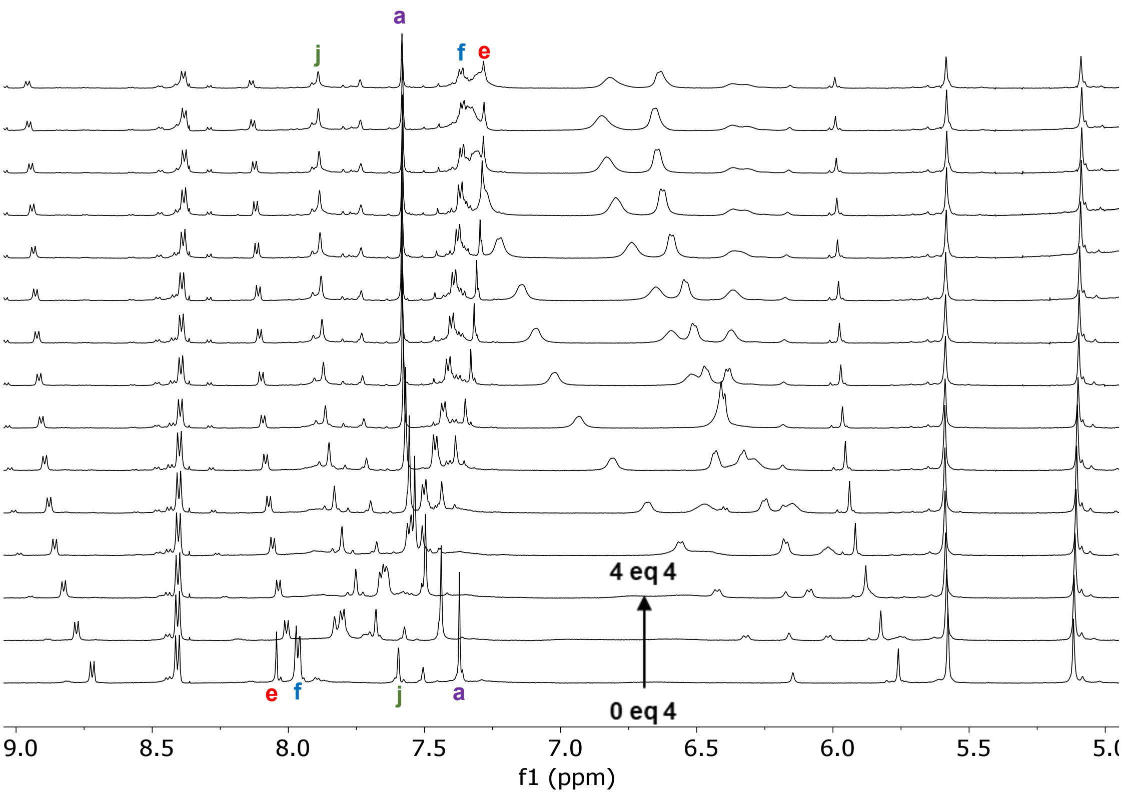

Figure S79: ^{1}H-NMR (500 MHz, D_2O) spectra of F_b^{3+} (1 mM) upon titration with 4 (10 mM) at pD = 11. The chemical shift of signals He, Hf, Hj and Ha were used for the fitting.

The mechanism proposed for the fitting process equilibria, and introduced on the software *Dynafit* was the following:[5] $\mathbf{F_b^{3+} + 4 \Longleftrightarrow 4{\subset}F_b^{3+}}$.

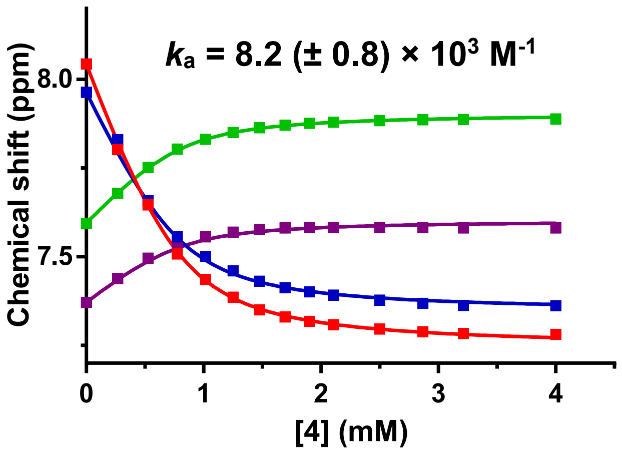

Figure S80: Fitting of experimental data (squares) for signals He, Hf, Hj and Ha.

2.8. Synthesis of 2-(2-(2-(2-(naphthalen-2-yloxy)ethoxy)ethoxy)ethoxy) acetaldehyde (Compound 3).

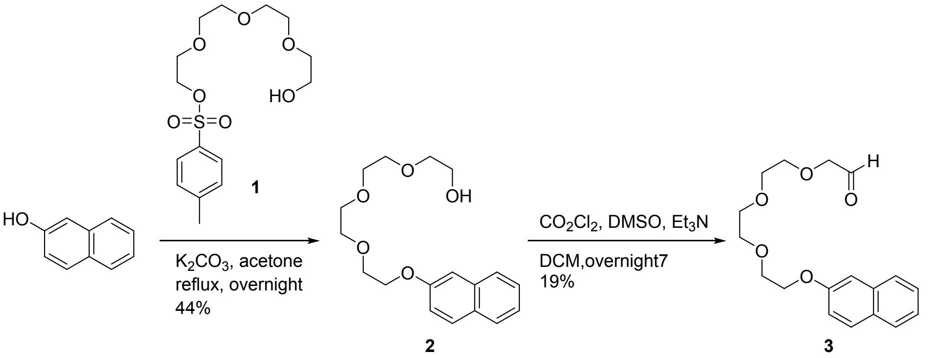

2-(2-(2-(2-(naphthalen-2-yloxy)ethoxy)ethoxy)ethoxy)ethan-1-ol (2): tosylate **1** (3.0012 g, 8.6 mmol) was mixed with acetone (50 mL). 2-naphtol (3.7389 g, 25.9 mmol) and K_2CO_3 (3.6201, 26.2 mmol) were added and the solution was stirred and heated to reflux overnight using a heat-on block. The reaction was followed by TLC and quenched by air cooling. The resulting oil was purified by chromatographic column (AcOEt), giving a viscous orange oil (1.2209 g, 44%).

^{1}H-NMR (D_2O, 400 MHz): δ = 7.92 (m, 3H), 7.58 (t, J = 7.5 Hz, 1H), 7.48 (t, J = 7.5 Hz, 1H), 7.43 (d, J = 2.6 Hz, 1H), 7.30 (dd, J_1 = 9.0 Hz, J_2 = 2.6 Hz, 1H), 4.39 (m, 2H), 4.00 (m, 2H), 3.82 (m, 2H), 3.75 (m, 2H), 3.68 (m, 6H), 3.59 (m, 2H). ^{13}C{^{1}H}-NMR (D_2O, 100 MHz): δ = 156.0 (C), 134.2 (C), 129.7 (CH), 128.9 (C), 127.7 (CH), 126.9 (CH), 126.8 (CH), 124.3 (CH), 118.6 (CH), 107.5 (CH), 71.7 (CH_2), 69.7 (CH_2), 69.7 (CH_2), 69.6 (CH_2), 69.4 (CH_2), 69.1 (CH_2), 67.2 (CH_2), 60.3 (CH_2). HRMS (ESI): *m/z* calculated for $C_{18}H_{25}O_5^+$ [*M*H]$^+$ 321.1697, found 321.1698; *m/z* calculated for $C_{18}H_{24}NaO_5^+$ [*M*+Na]$^+$ 343.1516, found 343.1518; *m/z* calculated for $C_{19}H_{25}O_6^+$ [*M*H+CHO]$^+$ 349.1646, found 349.1317; *m/z* calculated for $C_{19}H_{24}NaO_6^+$ [*M*+Na+CHO]$^+$ 371.1466, found 371.1136.

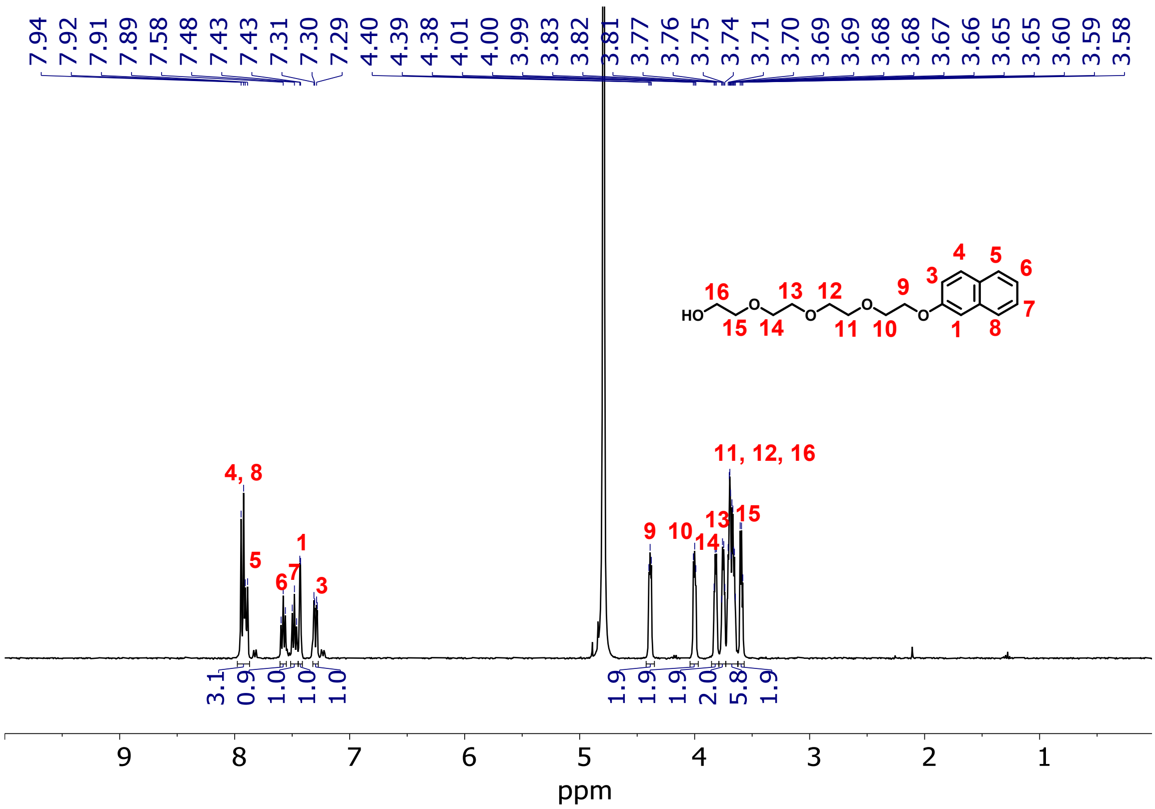

Figure S81. ^{1}H-NMR (400 MHz, D_2O) spectrum of Compound 2.

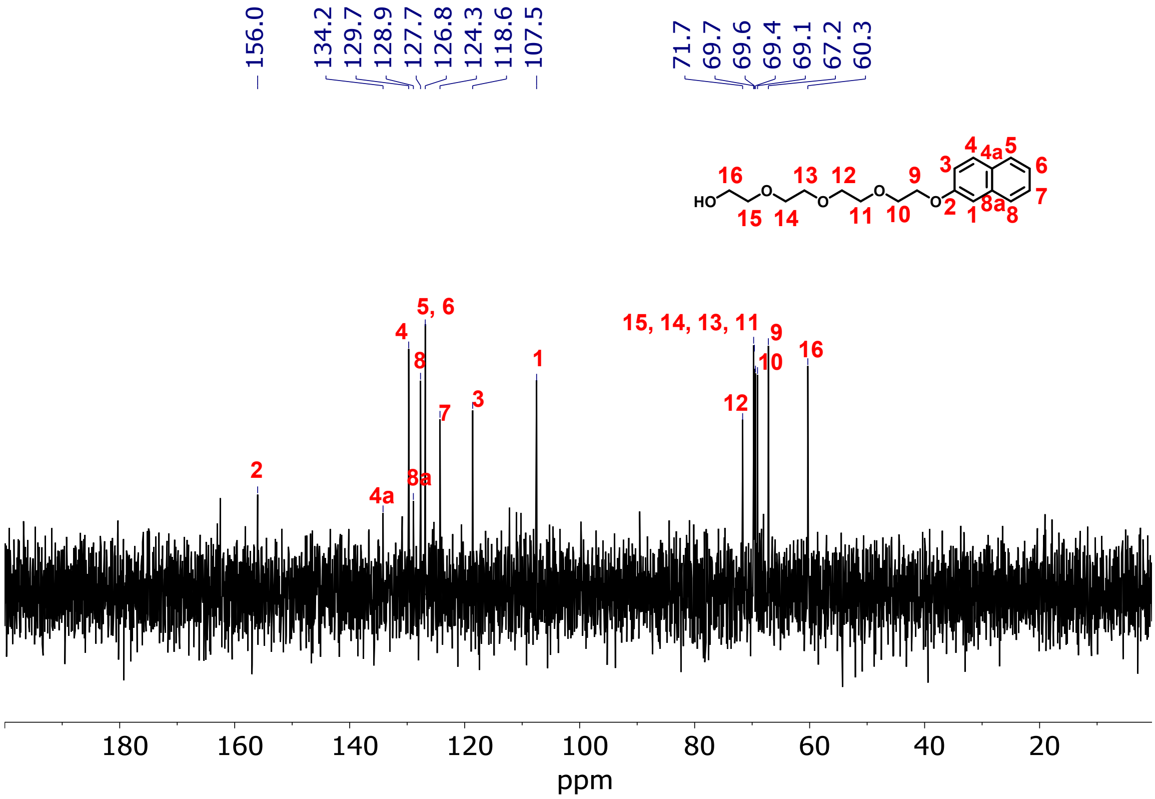

Figure S82. $^{13}C\{^{1}H\}$**-NMR (100 MHz,** D_2O**) spectrum of Compound 2.**

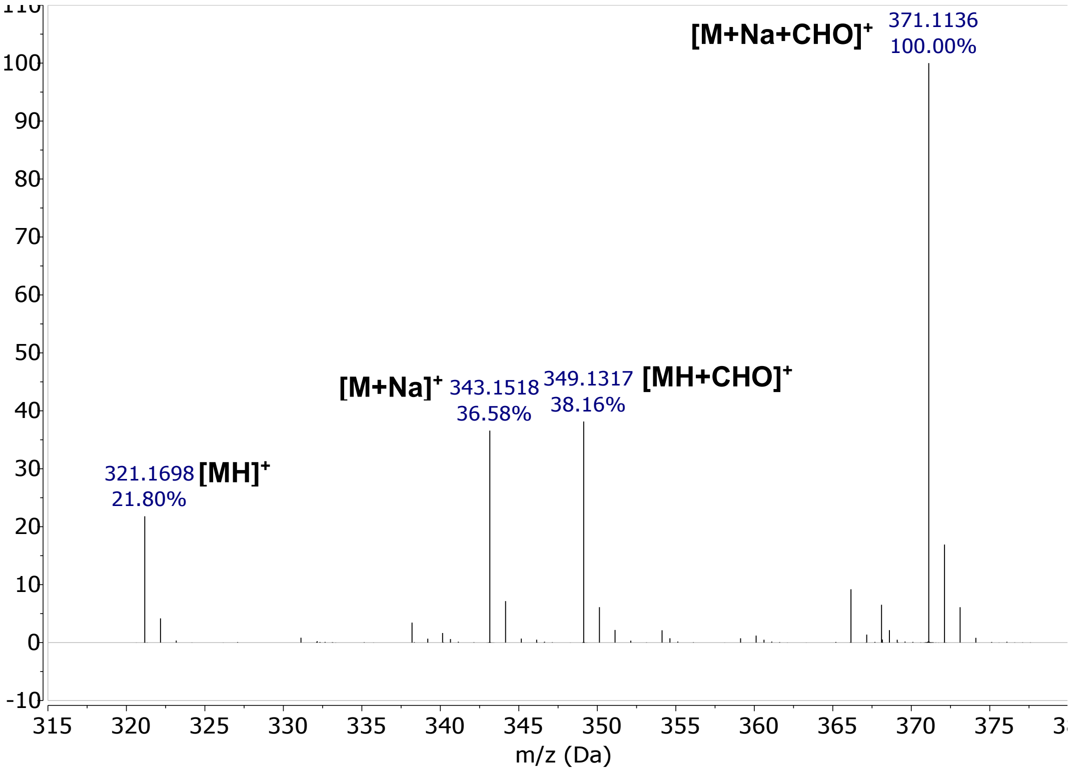

Figure S83: ESI-HRMS of Compound 2.

2-(2-(2-(2-(naphthalen-2-yloxy)ethoxy)ethoxy)ethoxy)acetaldehyde (3): Oxalyl chloride (0.7 mL, 8.2 mmol) was diluted in CH_2Cl_2 (anhydrous, 10 mL). DMSO (0.6 mL, 8.4 mmol) was diluted in DCM (anhydrous, 2 mL) and added dropwise under inert atmosphere at -33 ºC. After 5 min, 1-(2-naphtyl)tetraethyleneglycol **2** (1.2209 g, 3.8 mmol) was dissolved in DCM (anhydrous, 4 mL) and added dropwise. After 15 min, dry Et_3N (2.7 mL, 19.4 mmol) was added dropwise. The mixture was stirred at room temperature under inert atmosphere overnight. The reaction was followed by TLC and quenched by adding H_2O (25 mL).

The aqueous phase was extracted with DCM (2 x 15 mL). Organic phases were combined and washed with HCl 20% (10 mL) and $NaHCO_3$ 5% (10 mL). The resulting organic phase was dried with $MgSO_4$, filtered and concentrated to dryness, giving an orange oil.

The product was purified by chromatographic column (DCM:acetone 4:1), giving a viscous orange oil (231.0 mg, 19%).

^{1}H-NMR (D_2O, 500MHz): δ = 7.58 (d, J = 8.7 Hz, 2H), 7.82 (d, J =8.3 Hz, 1H), 7.49 (t, J = 7.6 Hz, 1H), 7.40 (t, J = 7.5 Hz, 1H), 7.35 (d, J = 2.6 Hz, 1H), 7.21 (dd, J_1 = 9.0 Hz, J_2 = 2.5 Hz, 1H), 5.06 (t, J = 5.0 Hz, 1H), 4.31 (m, 2H), 3.92 (m, 2H), 3.74 (m, 2H), 3.67 (m, 2H), 3.61 (m, 4H), 3.40 (d, J = 5.0 Hz, 2H). ^{13}C{^{1}H}-NMR (D_2O, 126 MHz): δ = 155.9 (C), 134.1 (C), 129.6 (CH), 128.8 (C), 127.6 (CH), 126.8 (CH), 128.7 (CH), 124.2 (CH), 118.5 (CH), 107.3 (CH), 88.0 (CH), 73.2 (CH_2), 69.9 (CH_2), 69.6 (CH_2), 69.5 (CH_2), 69.0 (CH_2), 67.0 (CH_2). HRMS (ESI): m/z calculated for $C_{18}H_{21}O_4^+$ [MH-H_2O]$^+$ 301.1434, found 301.1435.; m/z calculated for $C_{19}H_{26}NaO_6^+$ [M+MeOH+Na]$^+$ 373.1622, found 373.1622; m/z calculated for $C_{20}H_{26}NaO_7^+$ [M+MeOH+CHO+Na]$^+$ 301.1571, found 301.1241.

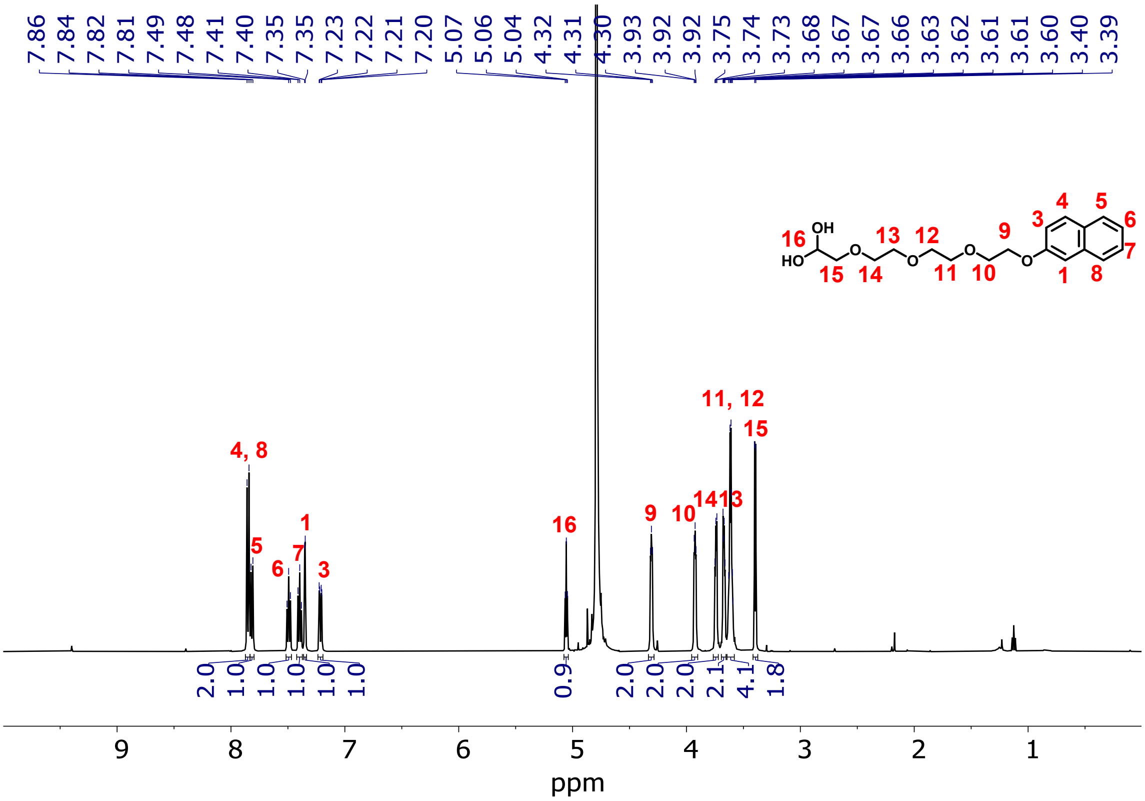

Figure S84: ^{1}H-NMR (500 MHz, D_2O) spectrum of Compound 3.

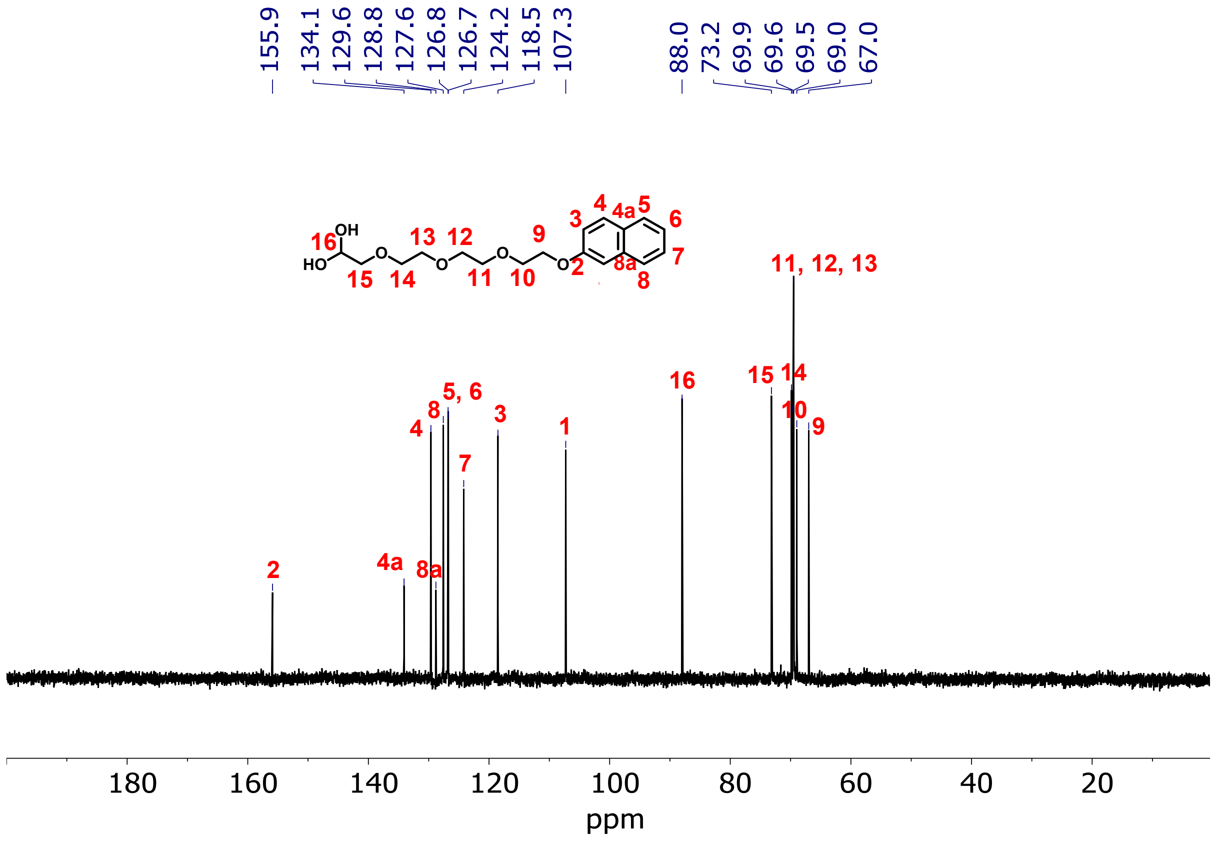

Figure S85: $^{13}C\{^{1}H\}$-NMR (126 MHz, D_2O) spectrum of Compound 3.

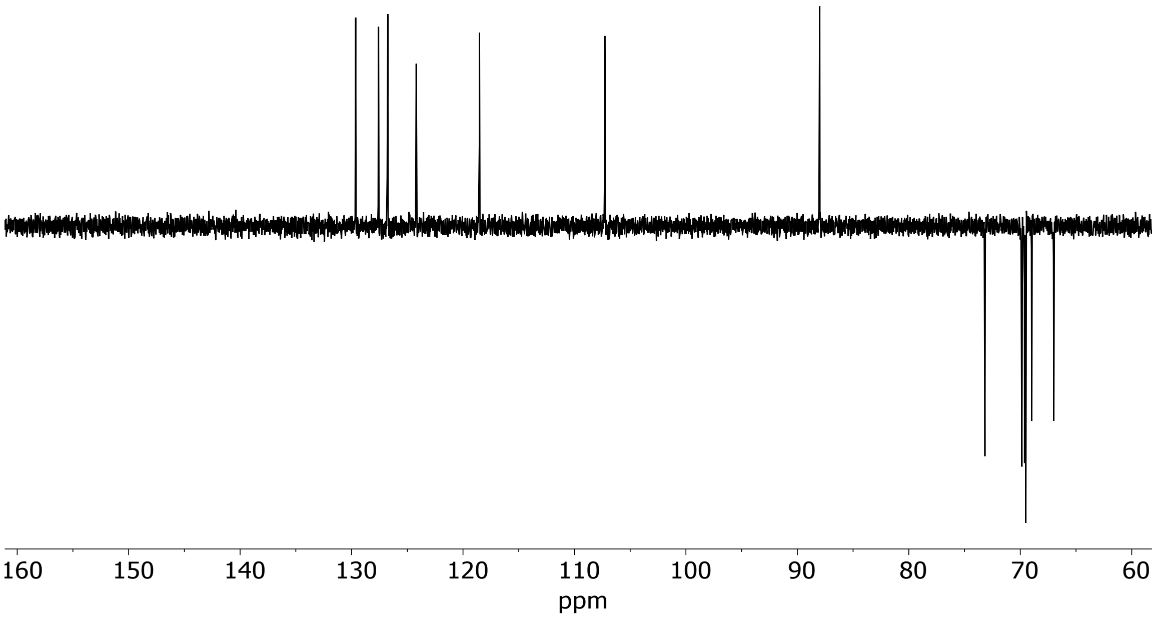

Figure S86: DEPT-135 (126 MHz, D_2O) spectrum of Compound 3.

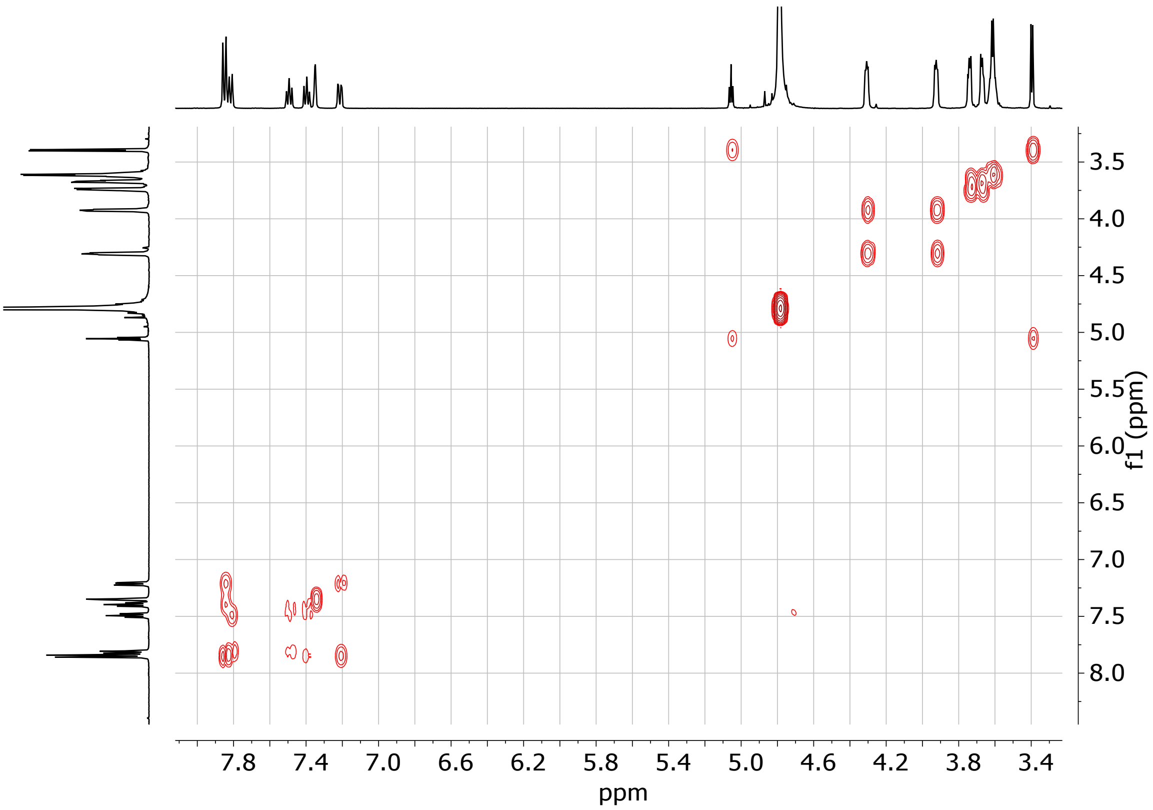

Figure S87: ^{1}H-^{1}H COSY (500 MHz, D_2O) spectrum of Compound 3.

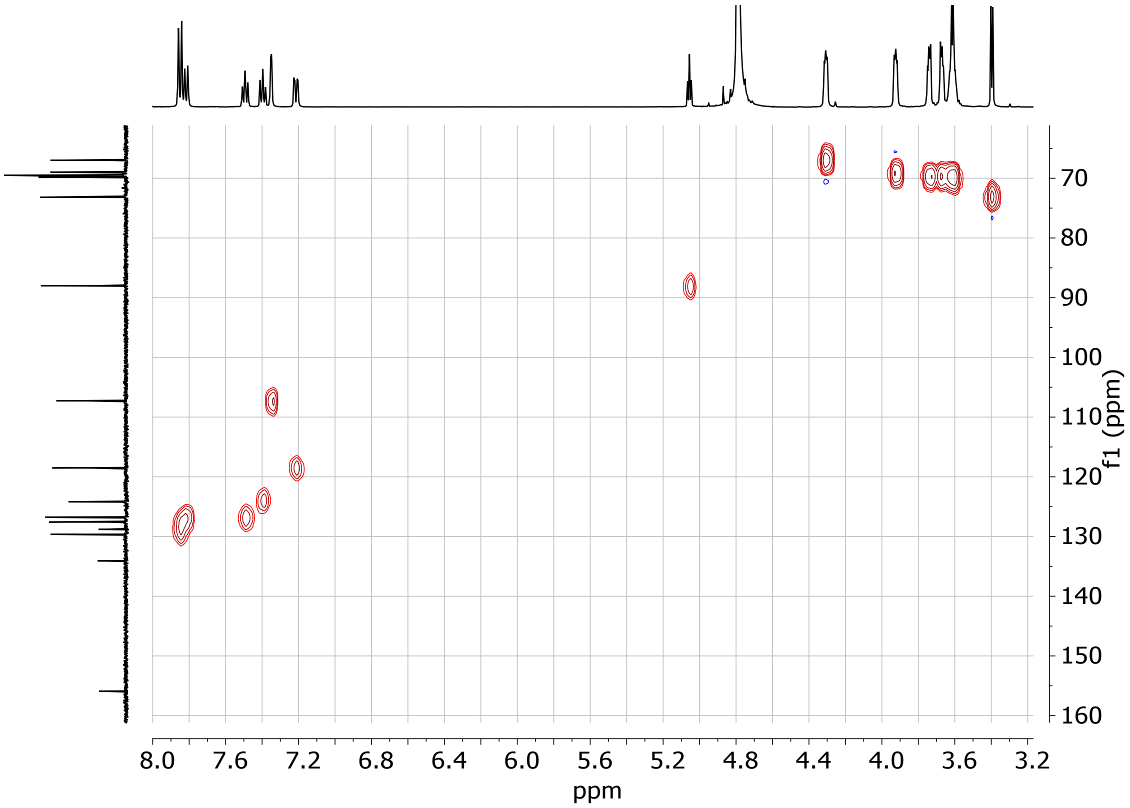

Figure S88: ^{1}H-$^{13}C\{^{1}H\}$ HSQC (500 and 126 MHz, D_2O) spectrum of Compound 3.

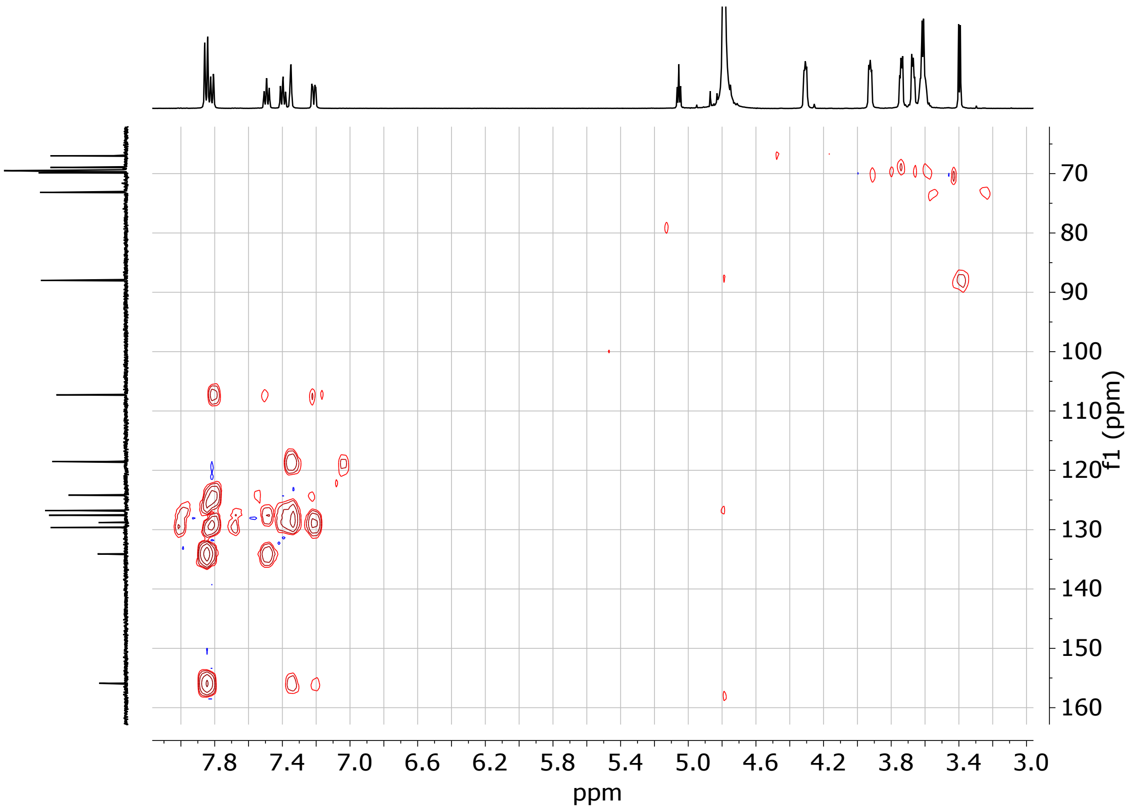

Figure S89: ^{1}H-^{13}C{^{1}H} HMBC (500 and 126 MHz, D_2O) spectrum of Compound 3.

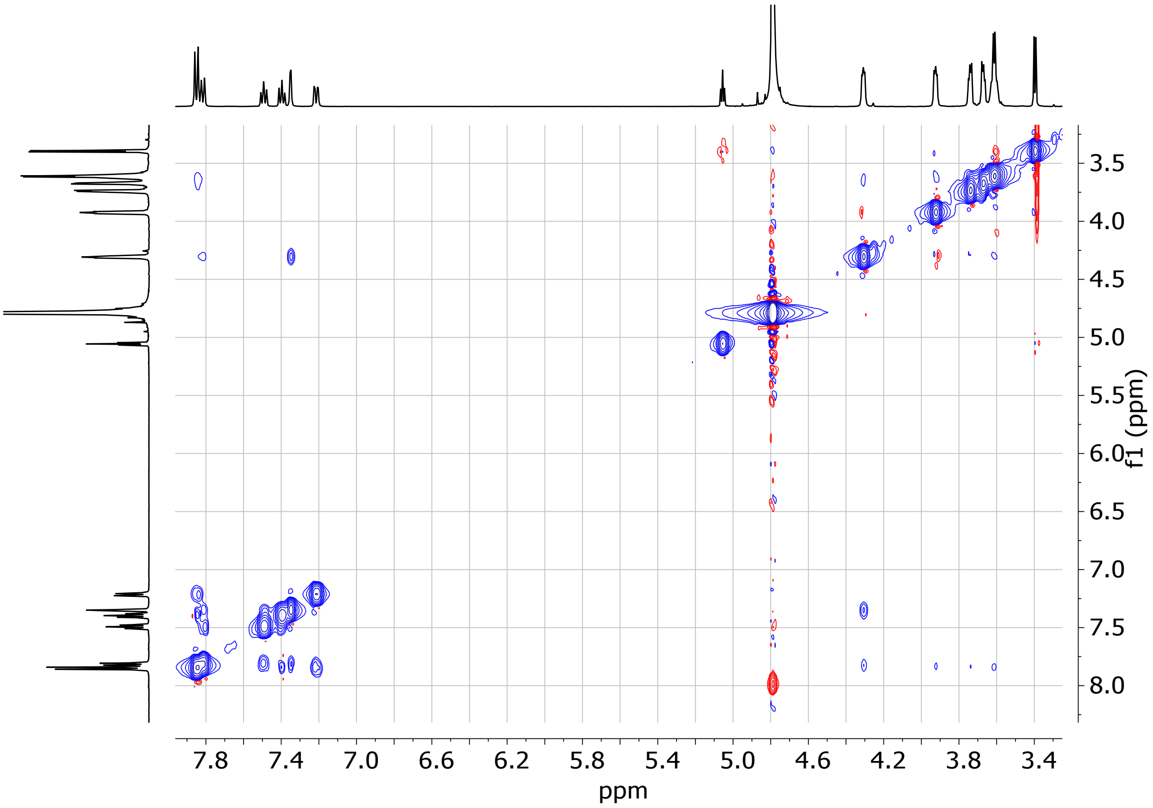

Figure S90: ^{1}H-^{1}H NOESY (500 MHz, D_2O) spectrum of Compound 3.

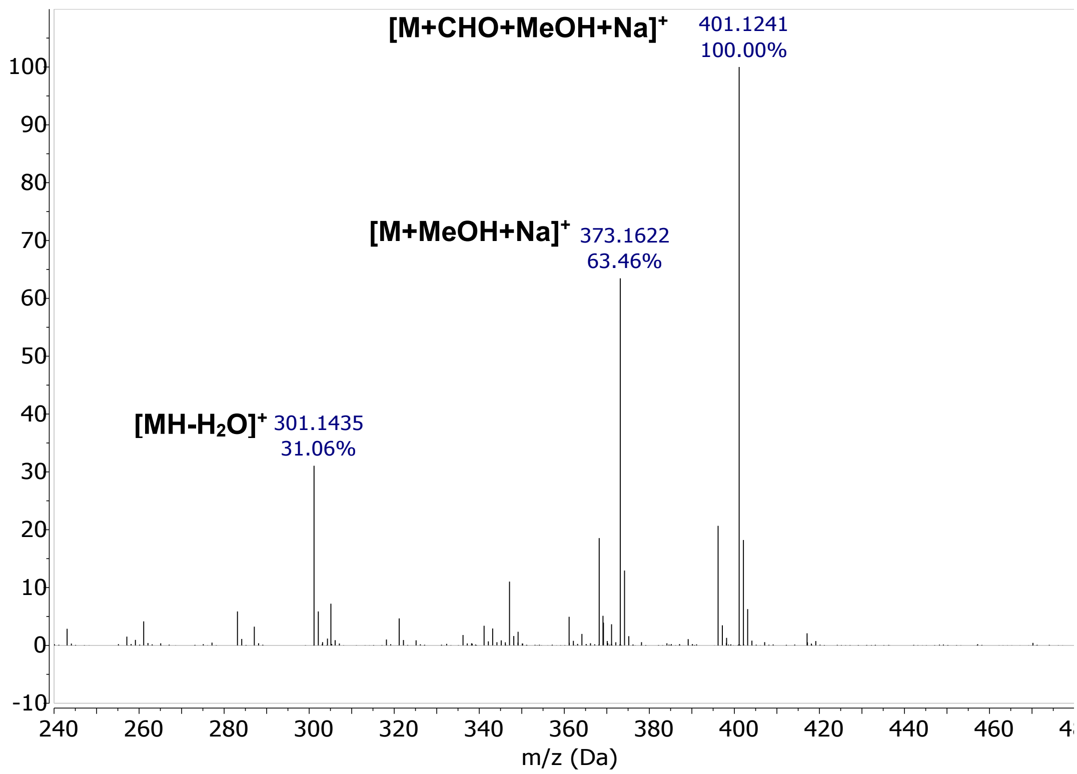

Figure S91: ESI-HRMS of Compound 3.

2.9. Synthesis of the pseudo[1]rotaxane S^{5+}.

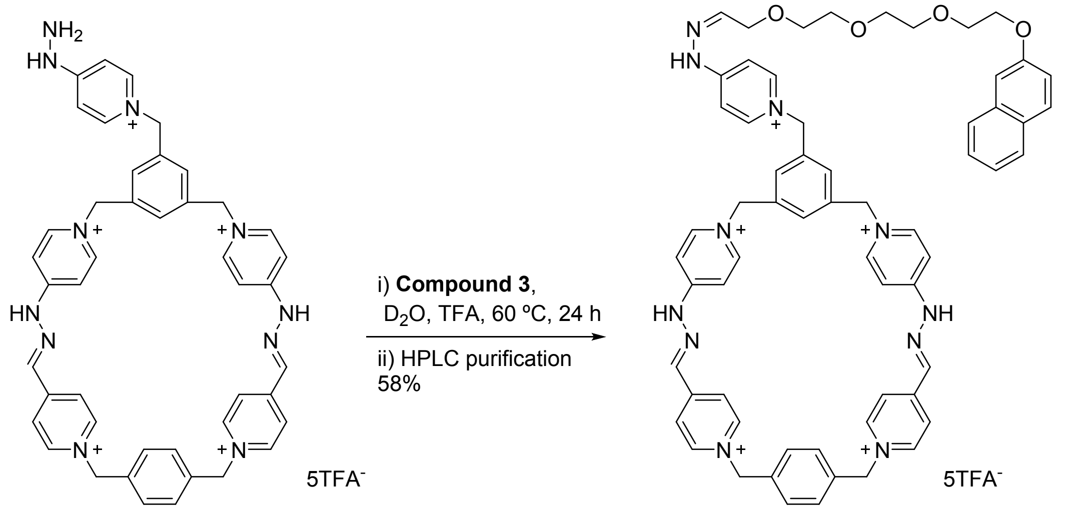

F_a^{5+} S^{5+}

A solution of $\mathbf{F_a}^{5+}$ (1.9 mg, 1.5 µmol), **Compound 3** (92 µL of a 24.5 mM/D_2O solution, 2.25 µmol) and TFA (10% molar) in 600 µL of D_2O was heated at 60 ºC for 24h using an oil bath.

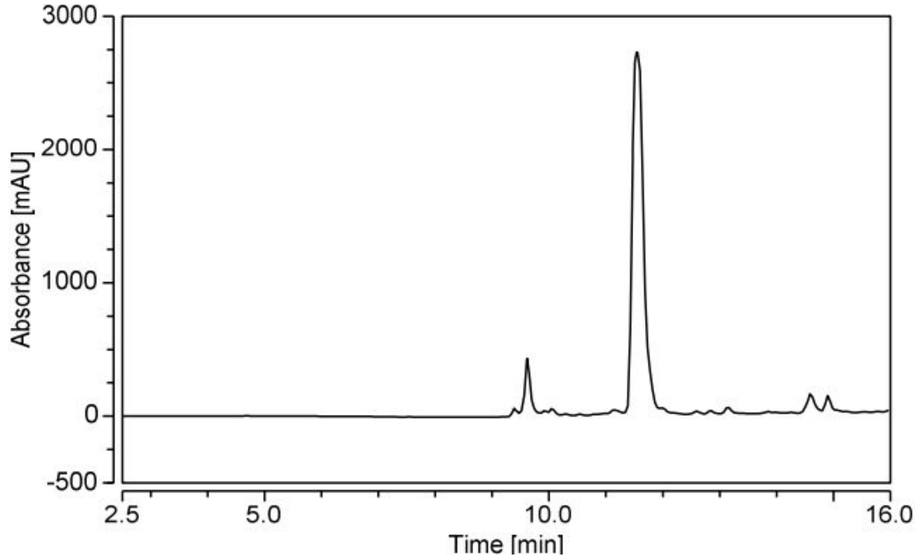

Figure S92: HPLC chromatogram of the raw reaction product showing a major peak at t_R = 10.7 min corresponding to S^{5+}.

The resulting solution was purified by reverse-phase HPLC (A: H_2O + 0.04% TFA, B: MeCN + 0.04% TFA), giving a yellowish solid (1.4 mg, 58%, $\mathbf{S^{5+}}$).

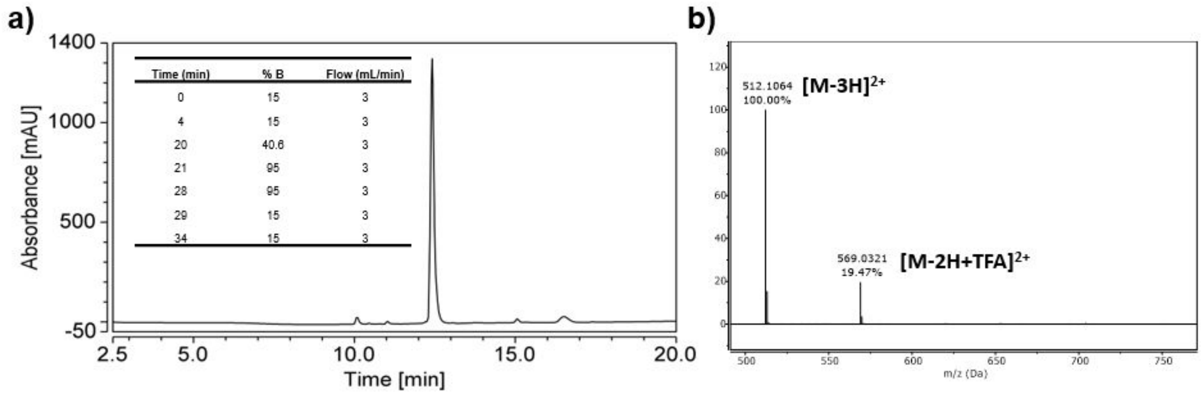

Figure S93: a) HPLC chromatogram (220 nm) of purified S^{5+} at t_R = 12.5 min (Inset: purification method). b) MS spectrum from the chromatographic peak at t_R = 12.5 min.

^{1}H-NMR (D_2O, 500MHz): δ = 8.84 (d, J = 6.4 Hz, 4H), 8.51 (s, 1H), 8.35 (m, 4H), 8.18 (s, 1H), 7.92 (s, 4H), 7.80 (s, 2H), 7.70 (s, 1H), 7.65 (s, 1H), 7.59 (m, 1H), 7.58 (d, J = 6.4 Hz, 4H), 7.25 (dd, J_1 = 7.4 Hz, J_2 = 2.7 Hz, 2H), 7.13 (s, 2H), 7.06 (s, 1H), 6.85 (dd, J_1 = 7.5 Hz, J_2 = 2.7 Hz, 2H), 6.69 (d, J = 8.2 Hz, 1H), 6.62 (d, J = 8.9 Hz, 1H), 6.35 (d, J = 2.5 Hz, 1H), 6.18 (d, J = 8.2 Hz, 1H), 5.99 (t, J = 7.5 Hz, 1H), 5.78 (s, 4H), 5.67 (t, J = 7.5 Hz, 1H), 5.60 (s, 2H), 5.53 (s, 4H), 5.06 (d, J = 8.9 Hz, 1H), 4.39 (d, J = 2.9 Hz, 2H), 3.81 (m, 6H), 3.74 (m, 4H), 3.50 (t, J = 5.2 Hz, 2H). ^{13}C{^{1}H}-NMR (D_2O, 126 MHz): δ = 154.9 (C), 154.7 (C), 149.0 (CH), 148.9 (C), 143.3 (CH), 143.3 (CH), 143.2 (CH), 139.3 (CH), 137.2 (C), 136.9 (C), 136.7 (C), 133.3 (C), 130.9 (CH), 130.4 (CH), 129.1 (CH), 128.4 (CH), 128.1 (C), 126.7 (CH), 125.5 (CH), 125.5 (CH), 124.8 (CH), 122.9 (CH), 117.5 (CH), 117.4 (CH), 115.1 (C), 110.4 (CH), 109.8 (CH), 108.5 (CH), 105.7 (CH), 70.0 (CH_2), 69.9 (CH_2), 69.8 (CH_2), 68.6 (CH_2), 68.5 (CH_2), 65.7 (CH_2), 64.3 (CH_2), 61.2 (CH_2), 60.5 (CH_2). HRMS (ESI): *m/z* calculated for $C_{62}H_{62}N_{11}O_4^{3+}$ [*M*-2H]$^{3+}$ 341.4990, found 341.4989.

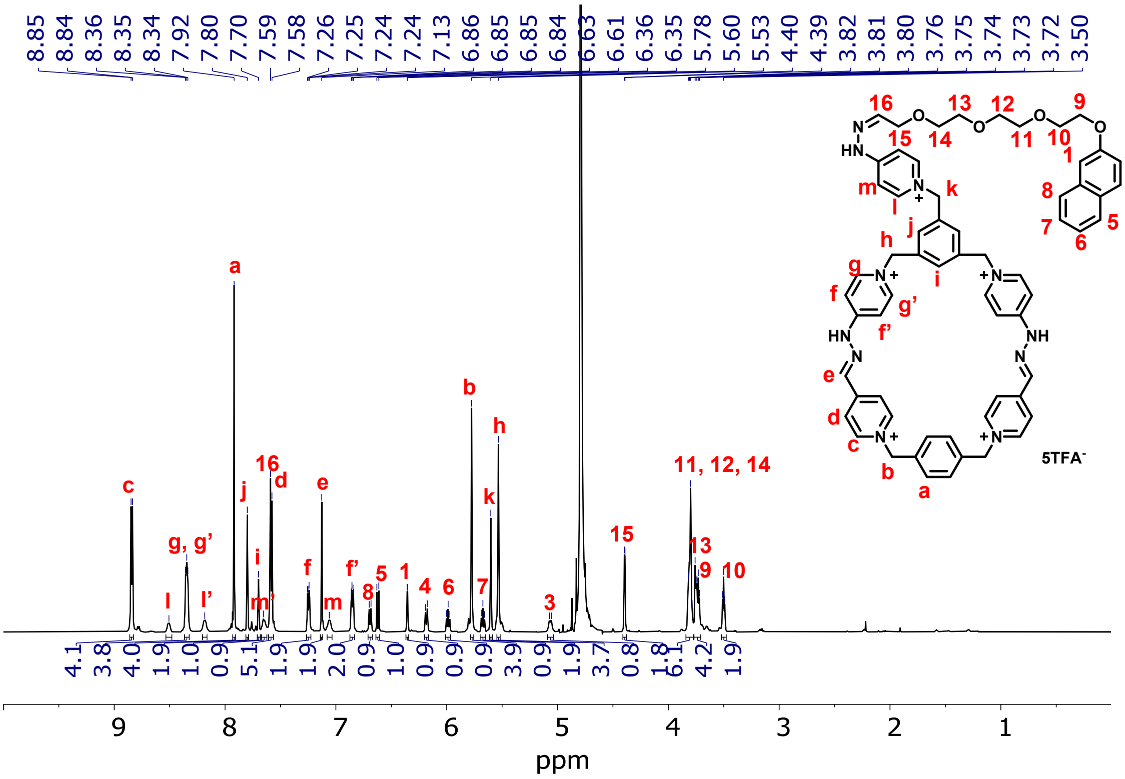

Figure S94: ^{1}H-NMR (500 MHz, D_2O) spectrum of S^{5+}.

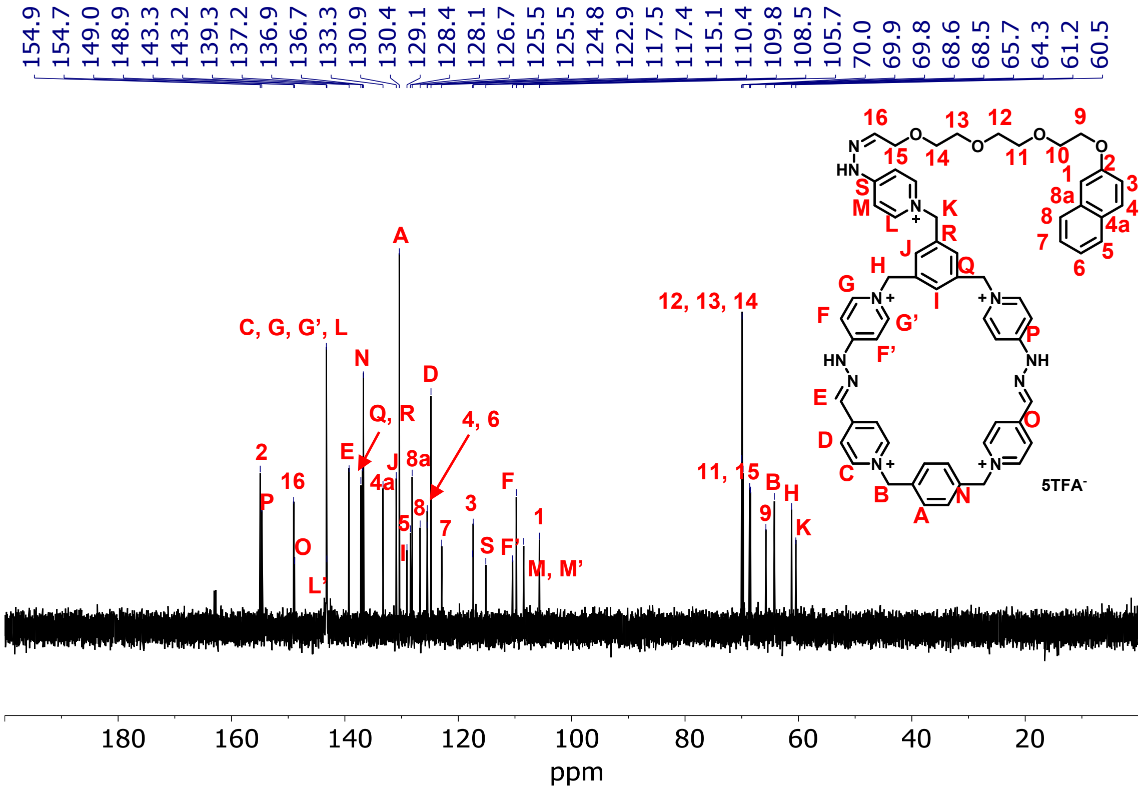

Figure S95: $^{13}C\{^{1}H\}$-NMR (126 MHz, D_2O) spectrum of S^{5+}.

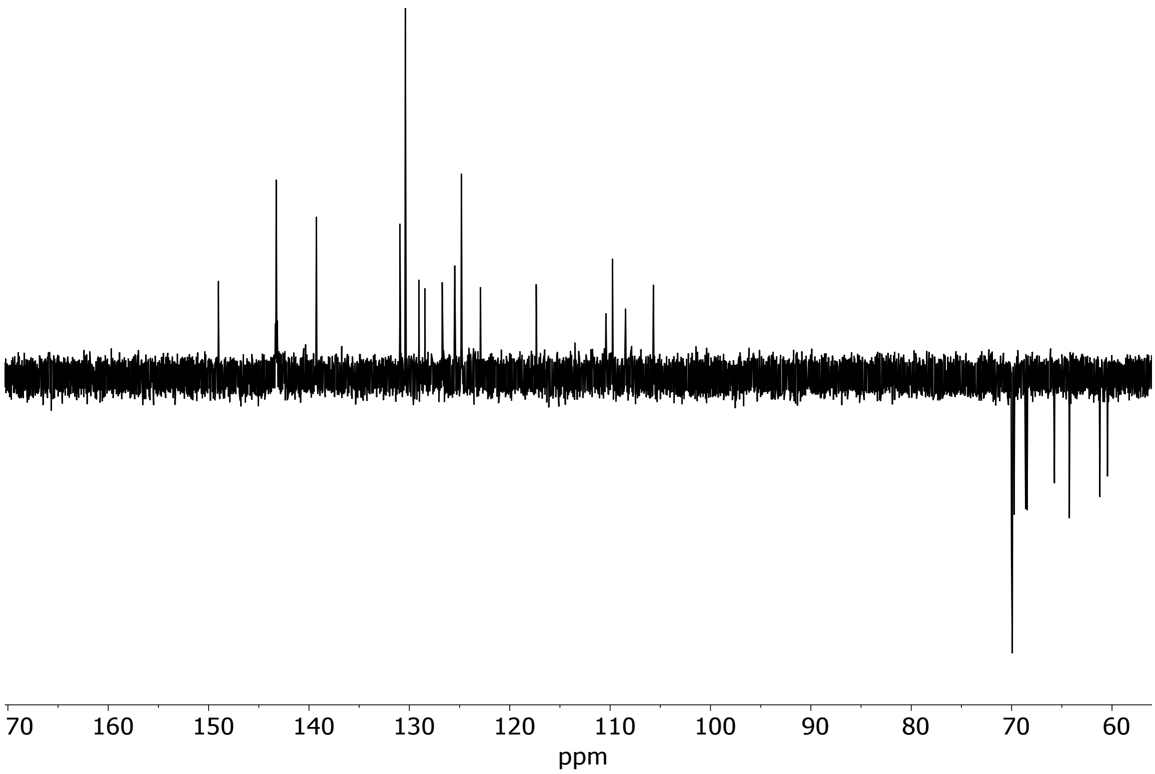

Figure S96: DEPT-135 (126 MHz, D_2O) spectrum of S^{5+}.

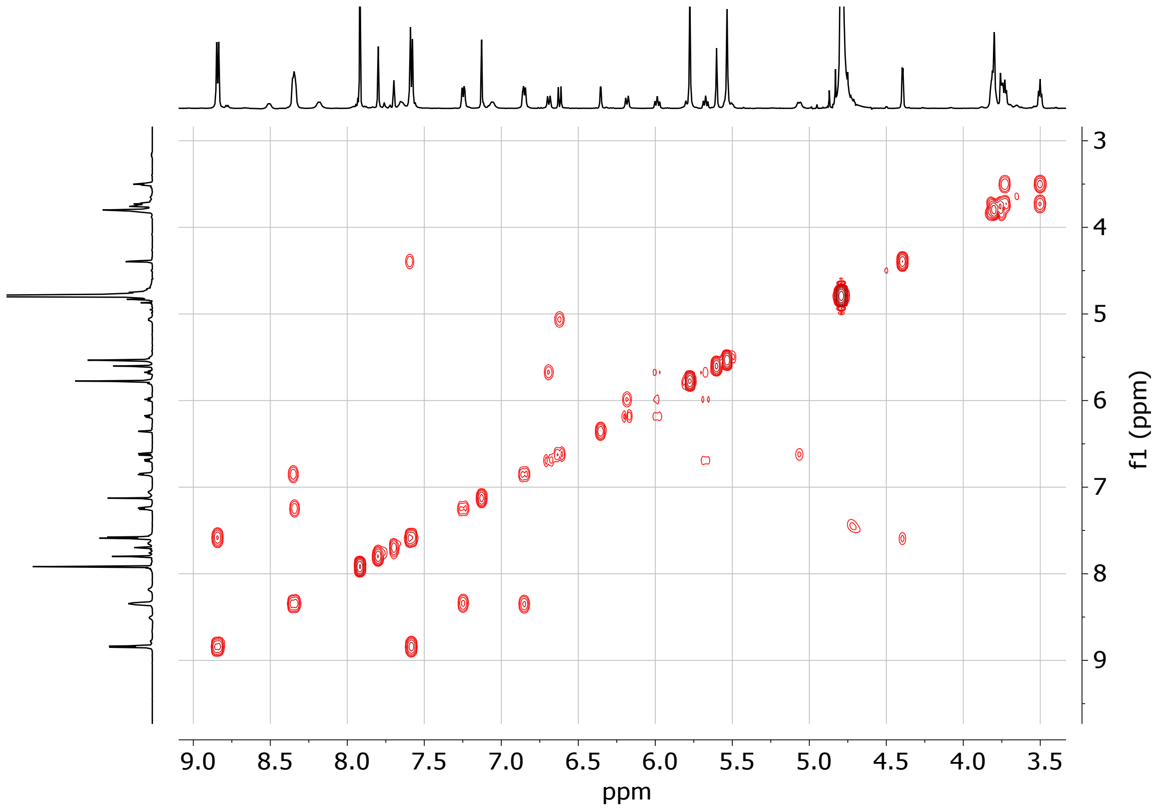

Figure S97: ^{1}H-^{1}H COSY (500 MHz, D_2O) spectrum of S^{5+}.

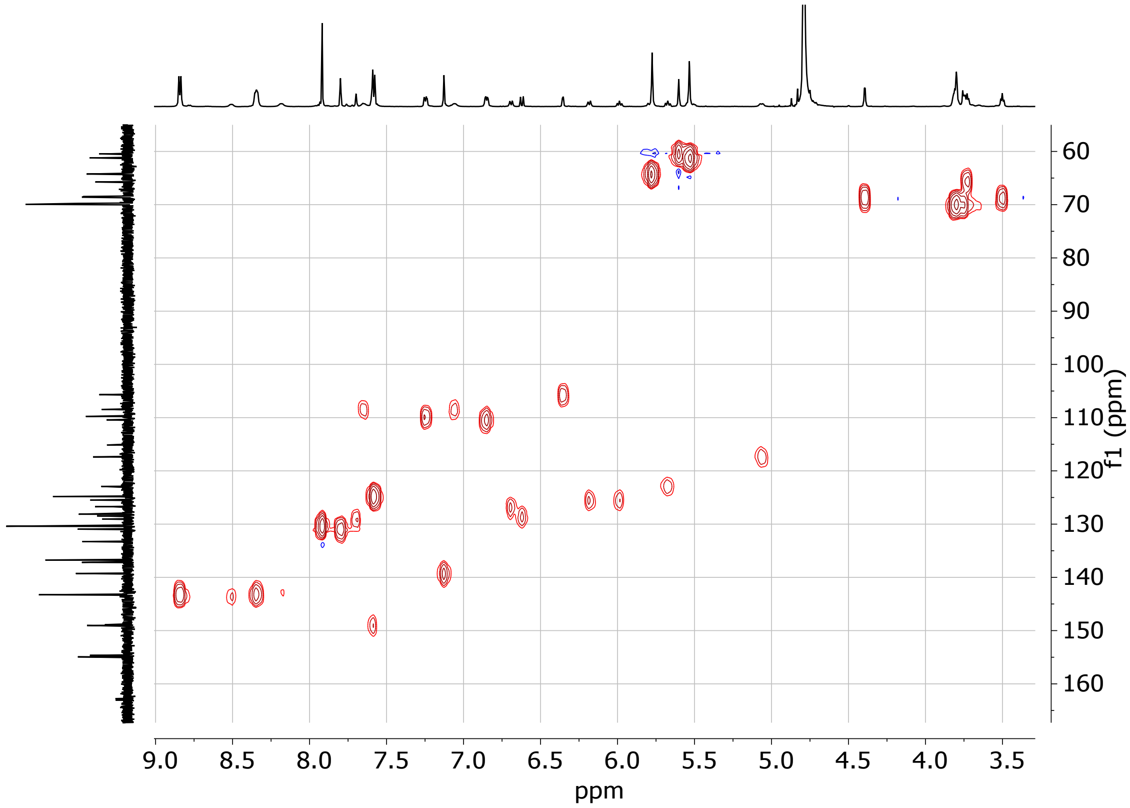

Figure S98: ^{1}H-$^{13}C\{^{1}H\}$ HSQC (500 and 126 MHz, D_2O) spectrum of S^{5+}.

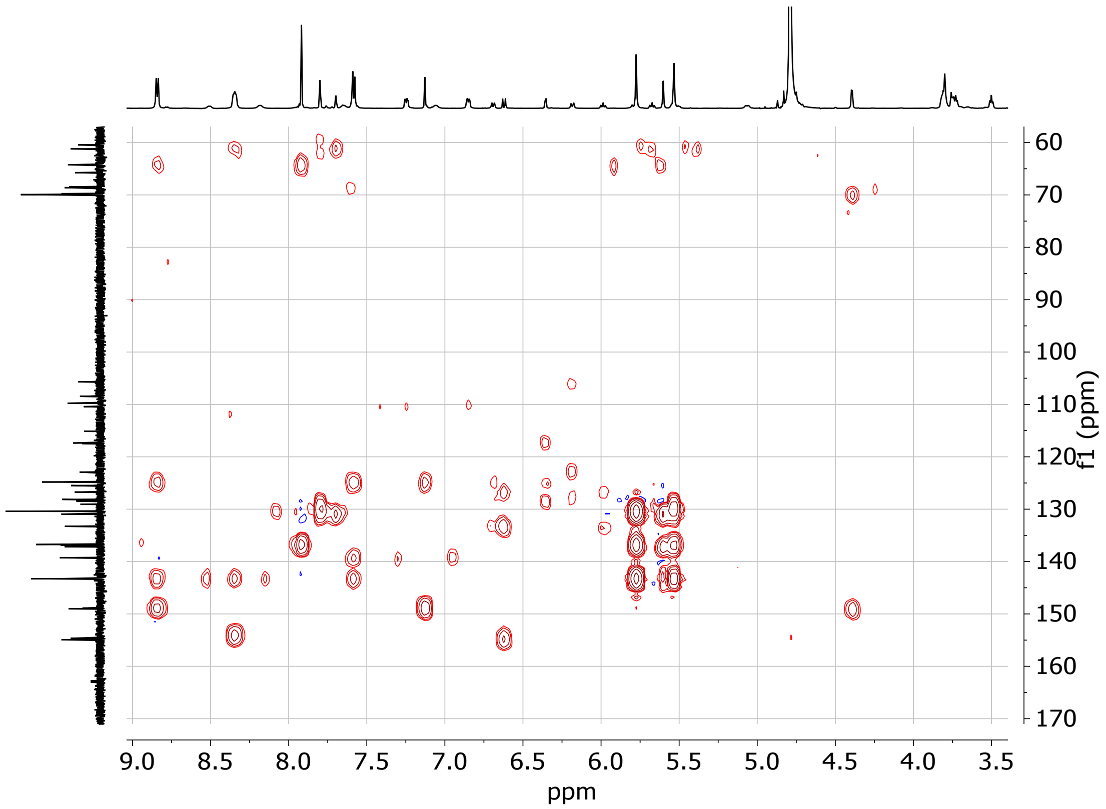

Figure S99: ^{1}H-$^{13}C\{^{1}H\}$ HMBC (500 and 126 MHz, D_2O) spectrum of S^{5+}.

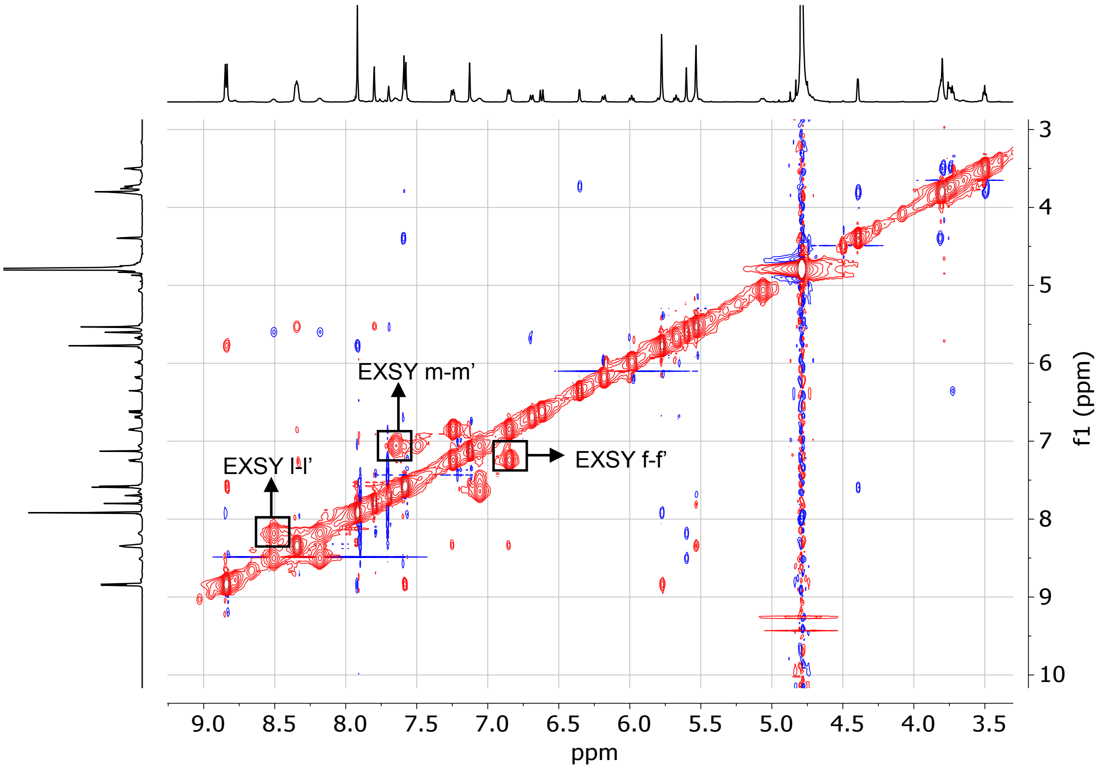

Figure S100: ^{1}H-^{1}H NOESY (500 MHz, D_2O) spectrum of S^{5+}.

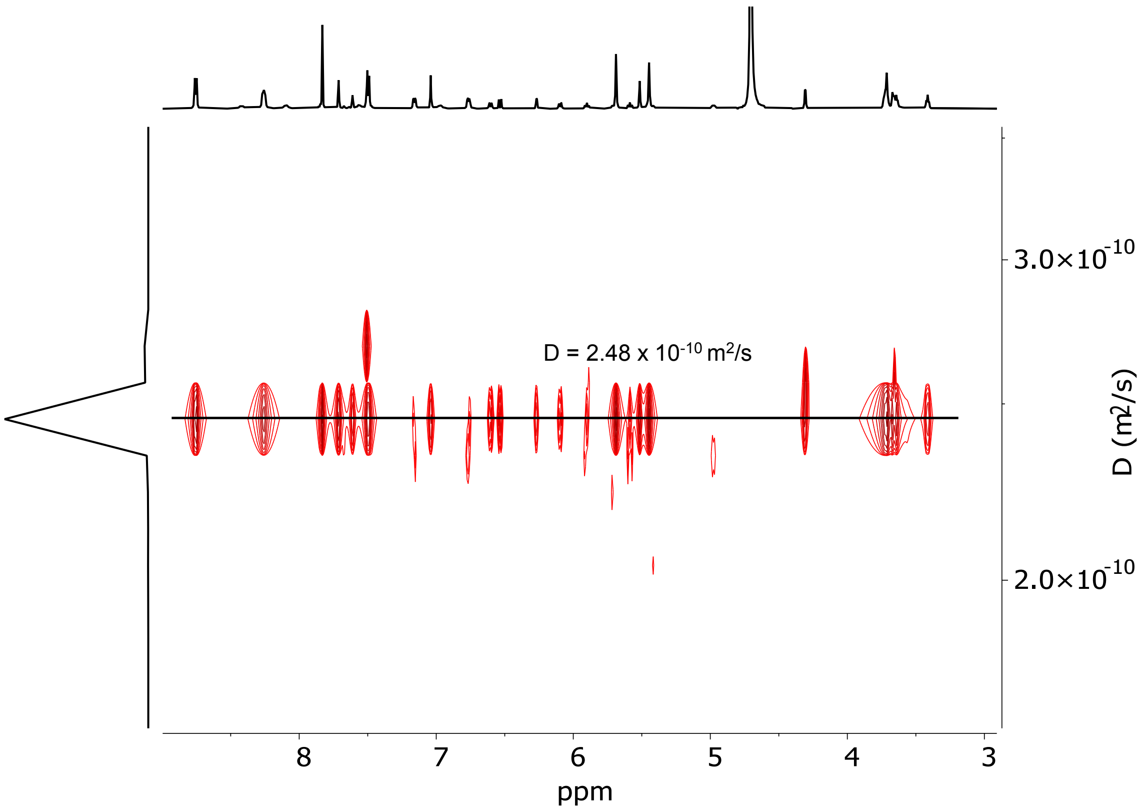

Figure S101: DOSY (500 MHz, D_2O) spectrum of S^{5+} at 2.5 mM.

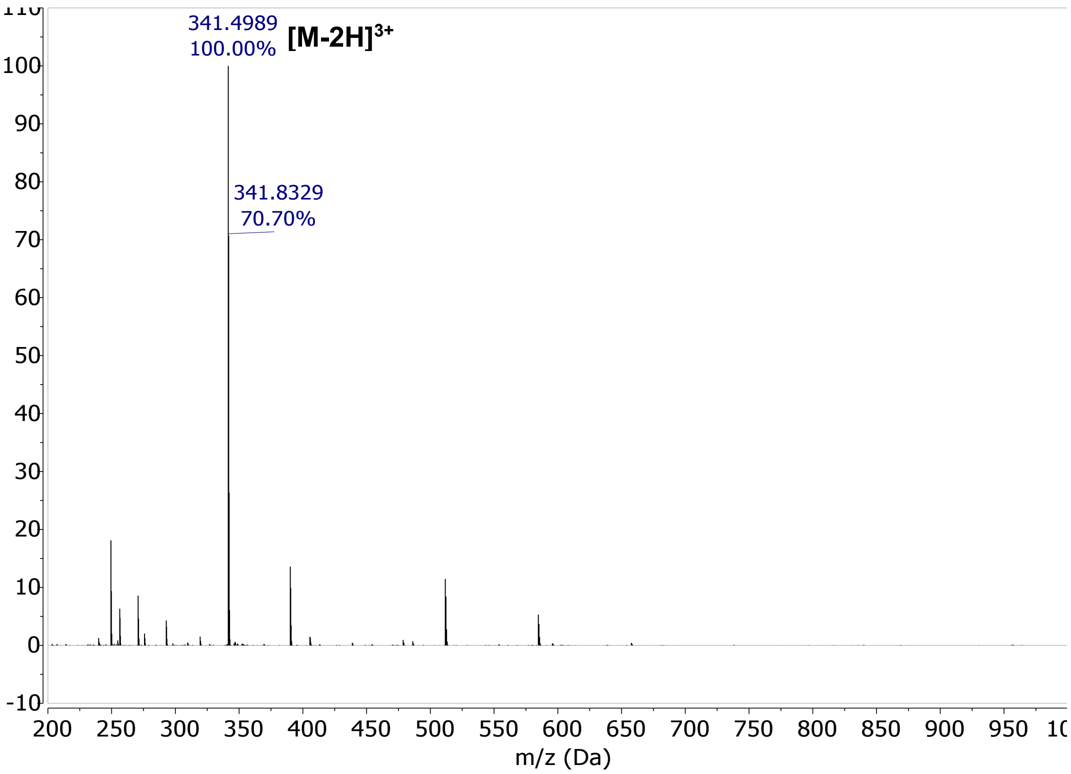

Figure S102: ESI-HRMS of S^{5+}.

2.10. Aggregation studies of S^{5+}.

2.10.1. UV-Vis at low concentrations.

The self-assembly of **S^{5+}** at low concentrations was assessed by means of UV-Vis spectroscopy. The linear correlation of the absorbance intensity with the concentration indicates the lack of self-assembly at the studied concentrations (0.3 – 25 μM).

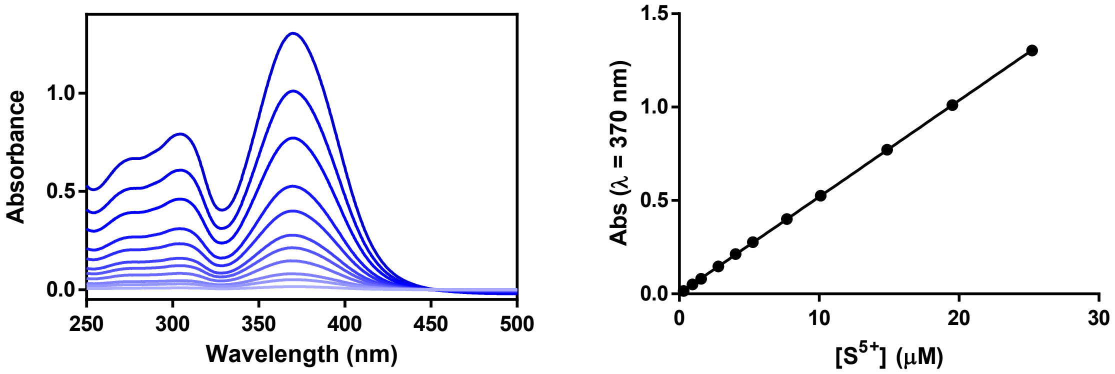

Figure S103: Absorption spectra of S^{5+} at different concentrations (from 0.3 to 25 μM) (left) and linear correlation of the absorbance with the concentration of S^{5+}.

2.10.2. ^{1}H-NMR and DOSY experiments at high concentration.

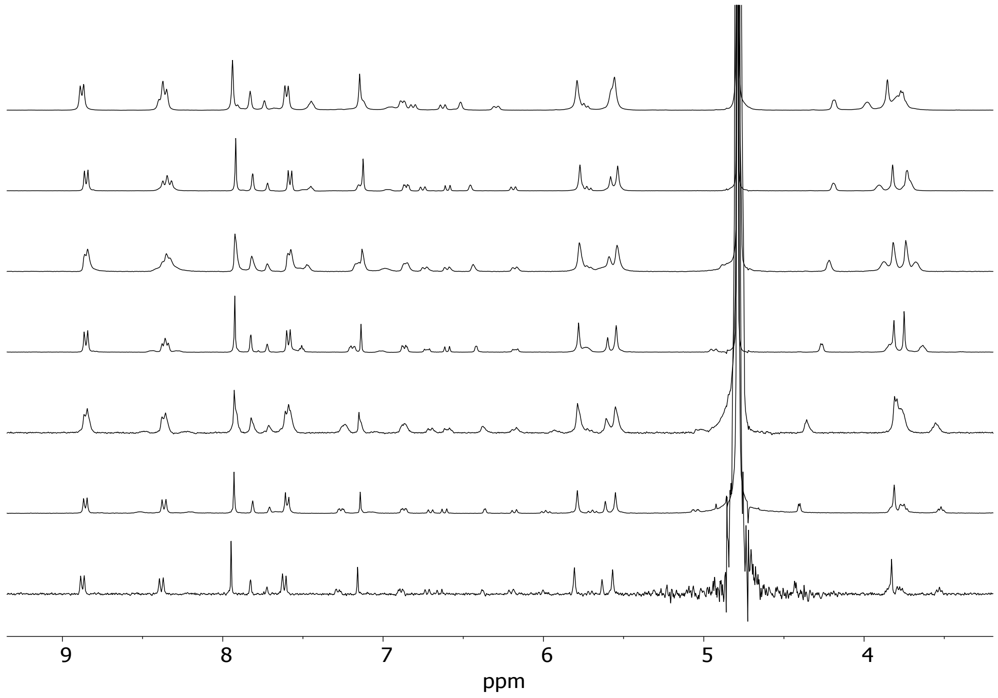

Figure S104: ^{1}H-NMR (300 MHz, D_2O) of S^{5+} at increasing concentrations: from 1 mM (bottom) to 70 mM (top).

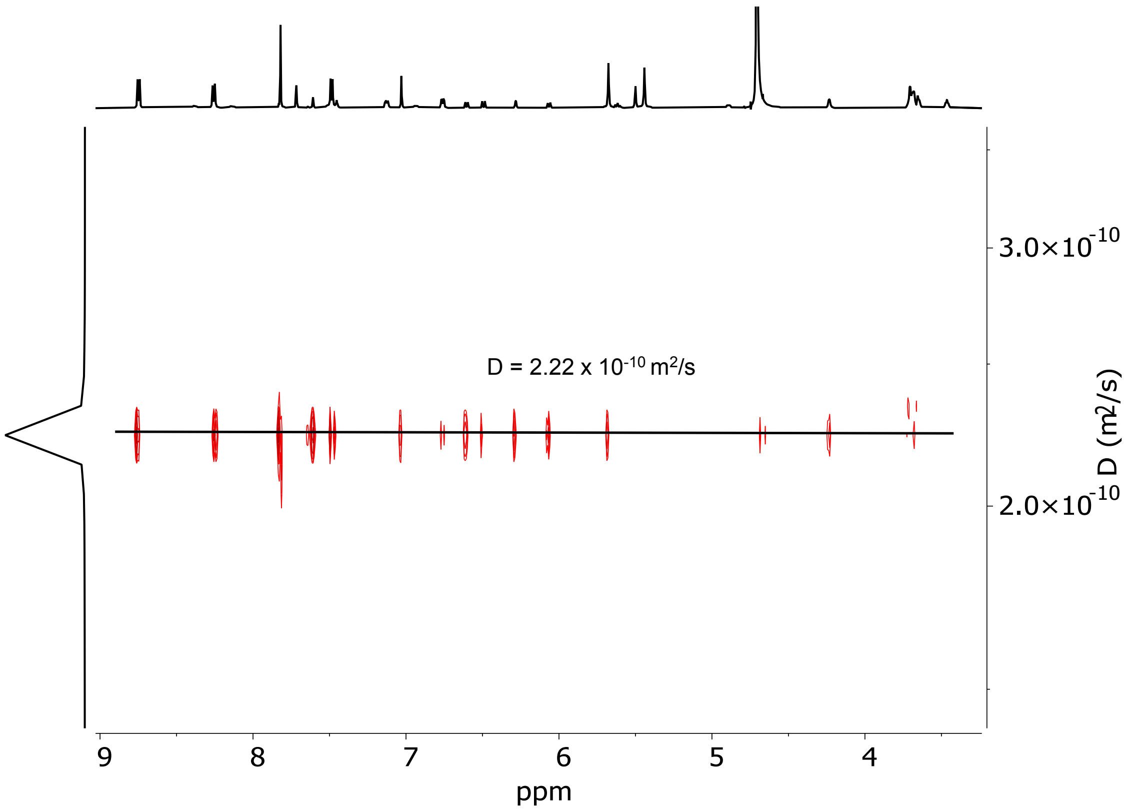

Figure S105: DOSY (500 MHz, D_2O) spectrum of S^{5*} at 10 mM.

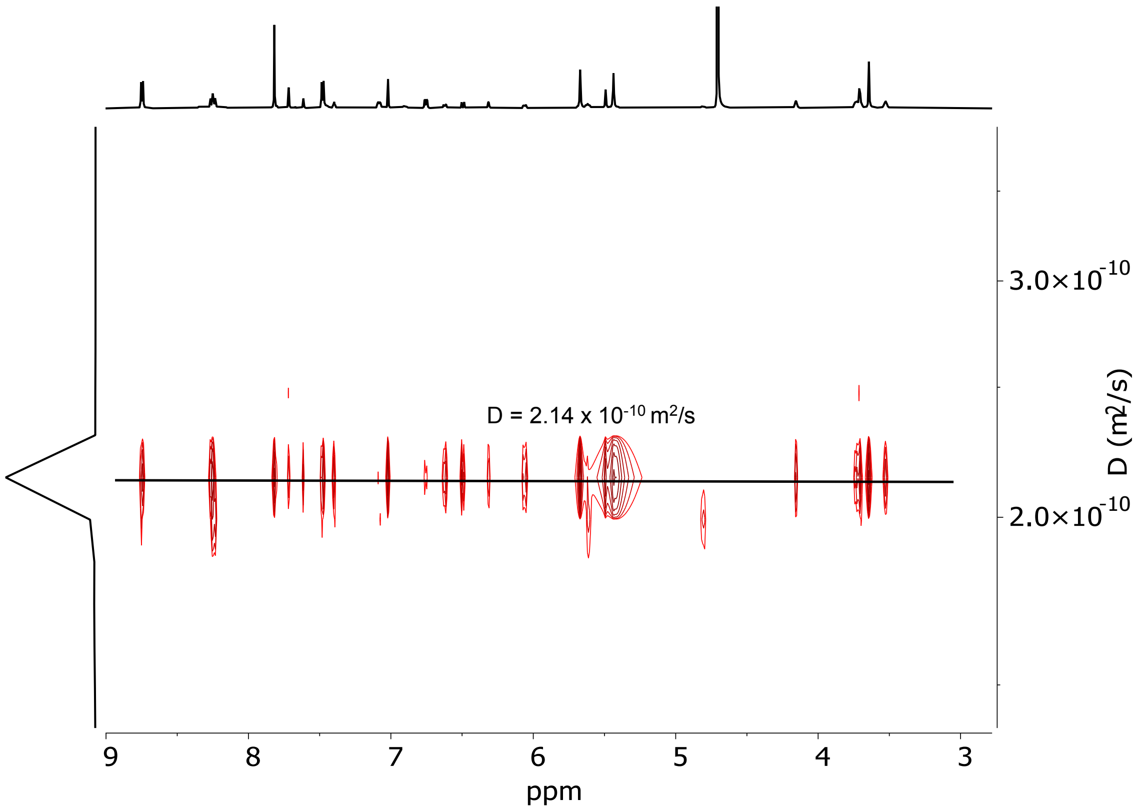

Figure S106: DOSY (500 MHz, D_2O) spectrum of S^{5*} at 20 mM.

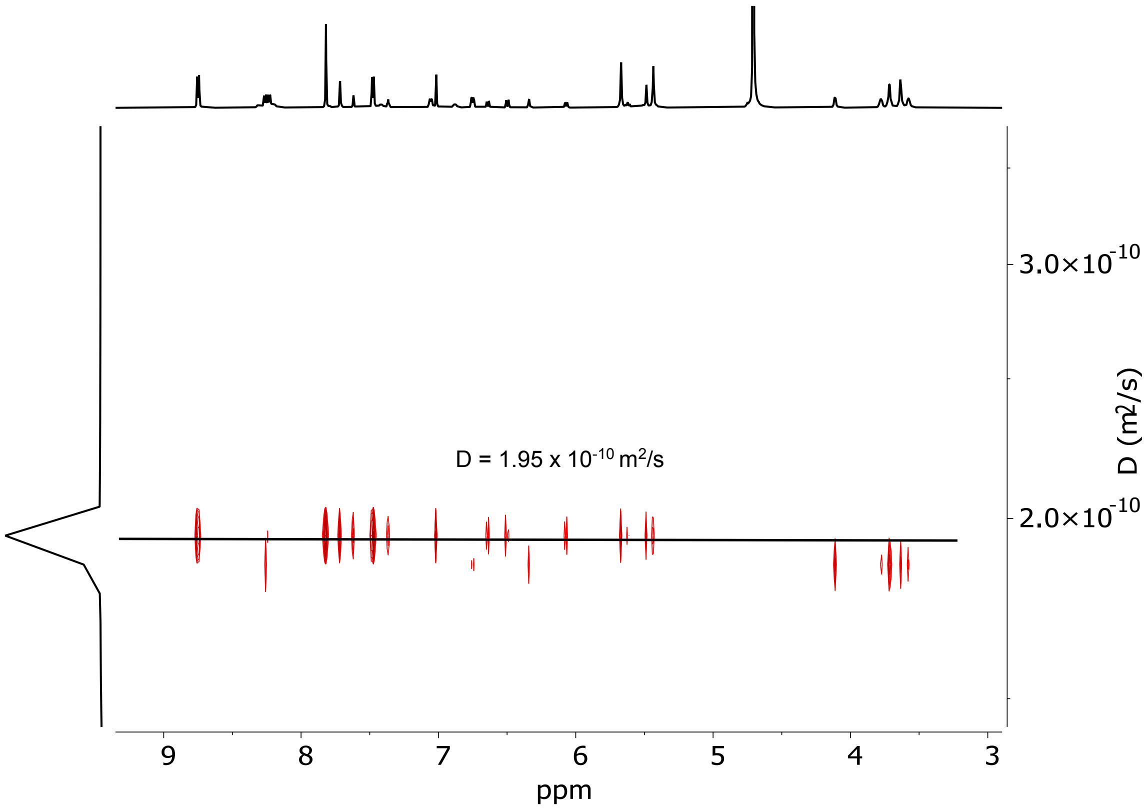

Figure S107: DOSY (500 MHz, D_2O) spectrum of S^{5*} at 30 mM.

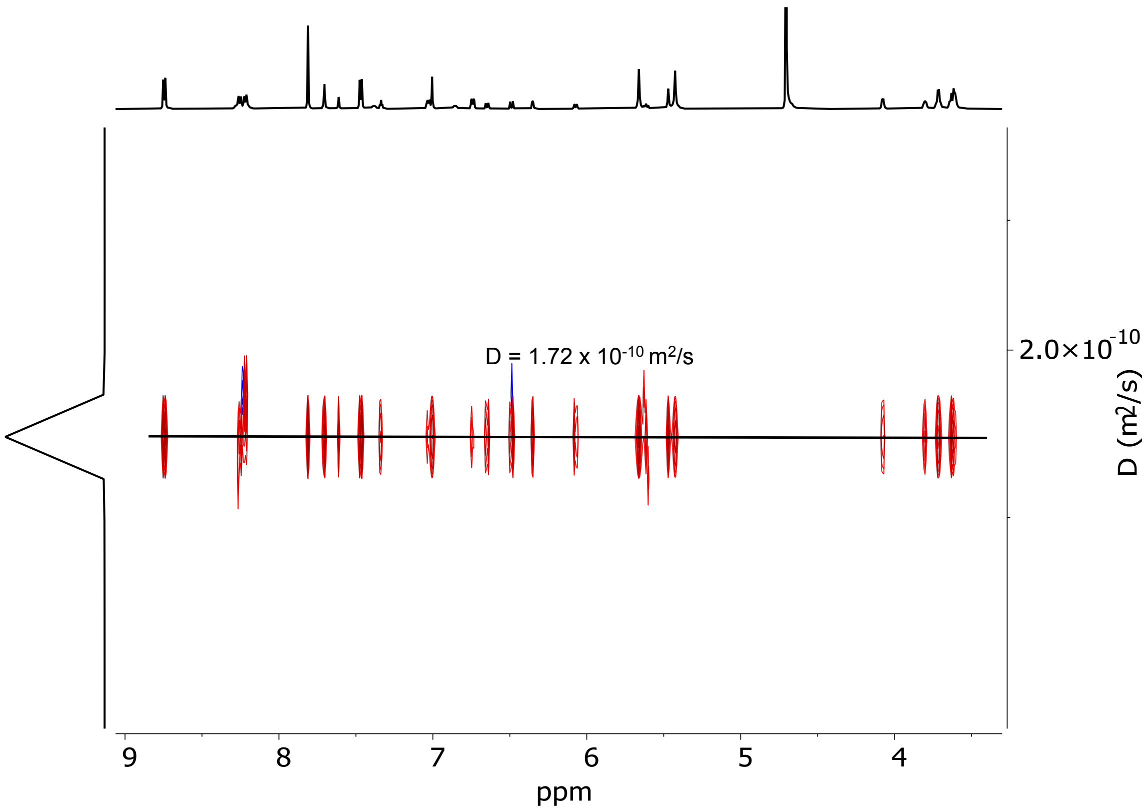

Figure S108: DOSY (500 MHz, D_2O) spectrum of S^{5*} at 40 mM.

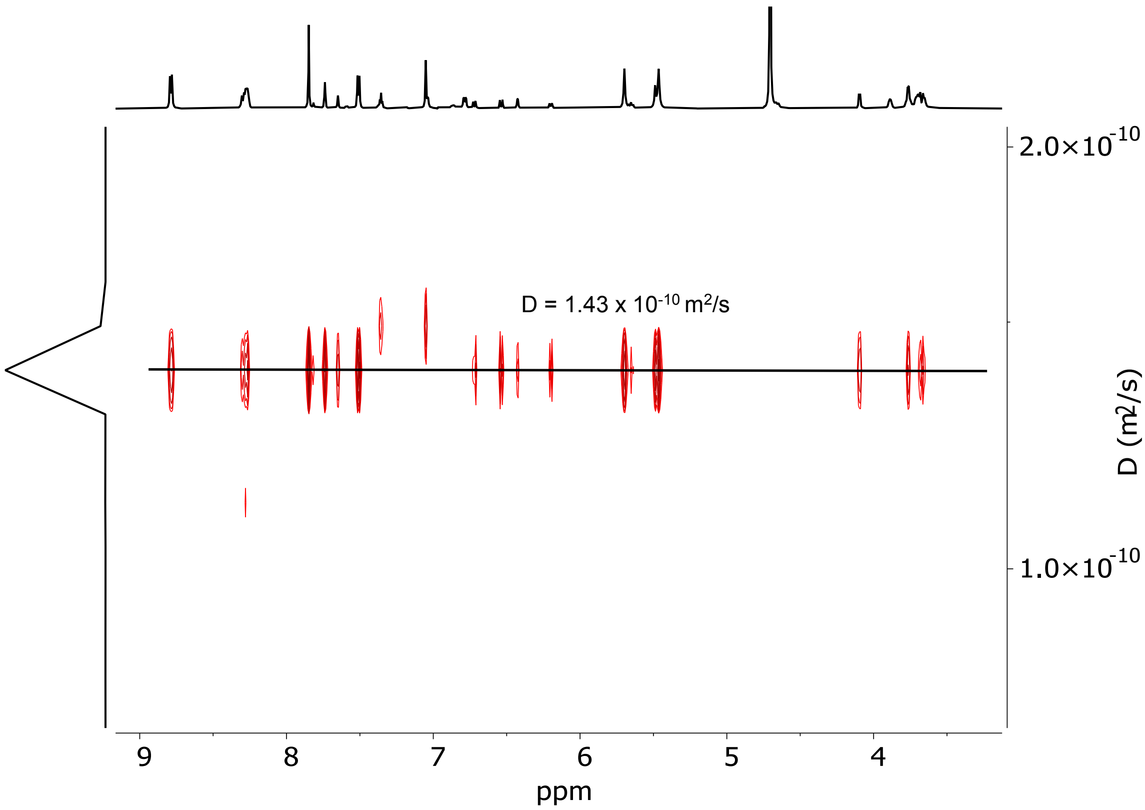

Figure S109: DOSY (500 MHz, D_2O) spectrum of S^{5*} at 70 mM.

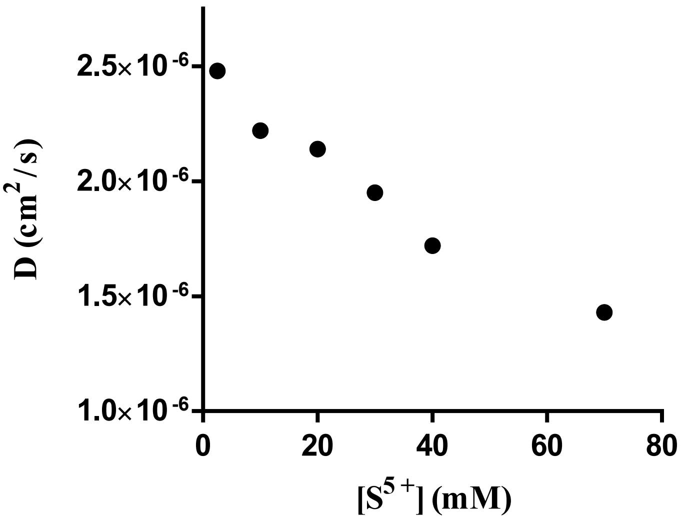

Figure S110: Evolution of the diffusion coefficient of S^{5+} upon increasing the concentration.

2.11. Determination of diffusion coefficient from theoretical model.

The diffusion coefficient was calculated from the modified Stokes-Einstein equation according to a prolate ellipsoid model.[6] The distances *a* and *b* for $\mathbf{S^{5+}}$ were obtained from the minimized structure as shown in **Figure S110**, considering the Van der Waals radius of carbon (1.7 Å).[7] The viscosity value μ used was D_2O (1.232×10^{-3} Pas at 298 K).[6]

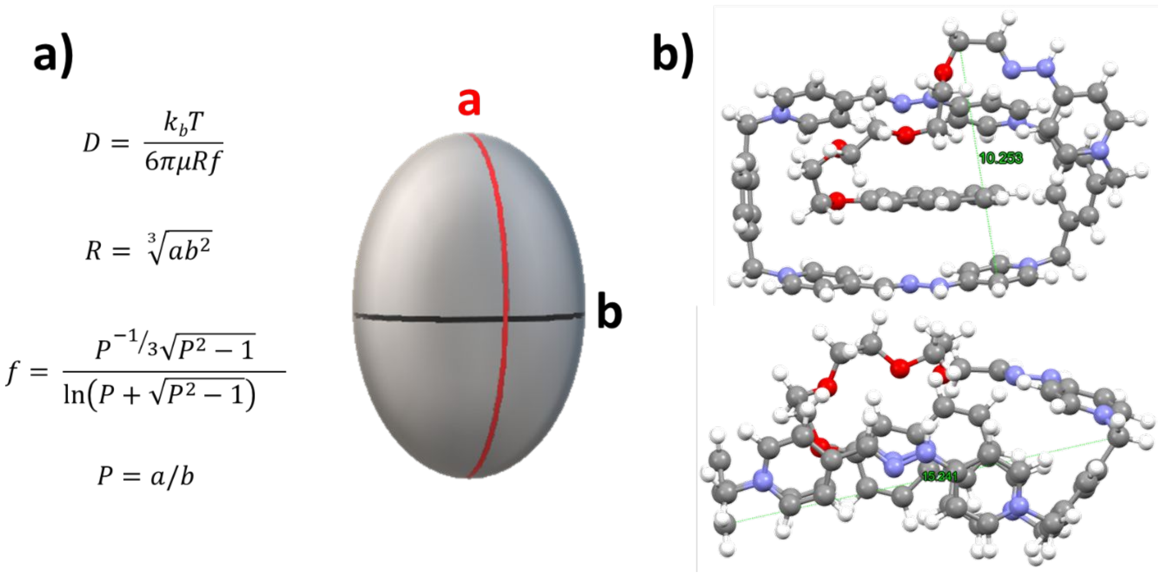

Figure S111: a) Stokes-Einstein equation according to a prolate ellipsoid model (k_b, Boltzmann constant; *T,* temperature; *μ*, viscosity; *R*, hydrodynamic radius; *f*, correction factor). b) Minimized structure of S^{5+} showing measured distances *a* (lower structure) and *b* (upper structure).

**a** × 10[10] (Å)[a]	**b** × 10[10] (Å)[a]	$\mathbf{R_{(cal)}}$ × 10[10] (Å)	$\mathbf{R_{(exp)}}$ × 10[10] (Å)[b,c]
9.32	6.83	7.57	7.25
**f**	**P**	$\mathbf{D_{(cal)}}$ × 10[10] (m^2s^{-1})	$\mathbf{D_{(exp)}}$ × 10[10] (m^2s^{-1})[b]
1.0087	1.3653	2.32	2.45

[a] Data obtained from the minimized structure of $\mathbf{S^{5+}}$ and added Van der Waals radius of carbon. [b] Experimental data obtained from DOSY NMR experiment of $\mathbf{S^{5+}}$ at 2.5 mM. [c] Value of the hydrodynamic radius of $\mathbf{S^{5+}}$ at 2.5 mM from $D_{(exp)}$ using the unmodified Stokes-Einstein equation.

Table S3: Data obtained from the minimized S^{5+} structure and the modified Stokes-Einstein equation and experimental data of S^{5+} at 2.5 mM.

[6] a) N. J. Wheate, P. G. A. Kumar, A. M. Torres, J. R. Aldrich-Wright and William S. Price, *J. Phys. Chem. B*, 2008, **112**, 2311-2314. b) V. Blanco, M. D. Garcia, C. Platas-Iglesias, C. Peinador and J. M. Quintela, *Chem. Commun.*, 2010,**46**, 6672-6674.
[7] A. Bondi, *J. Phys. Chem.*, 1964, **68**, 441-451.

2.12. One-pot synthesis of S^{5+}.

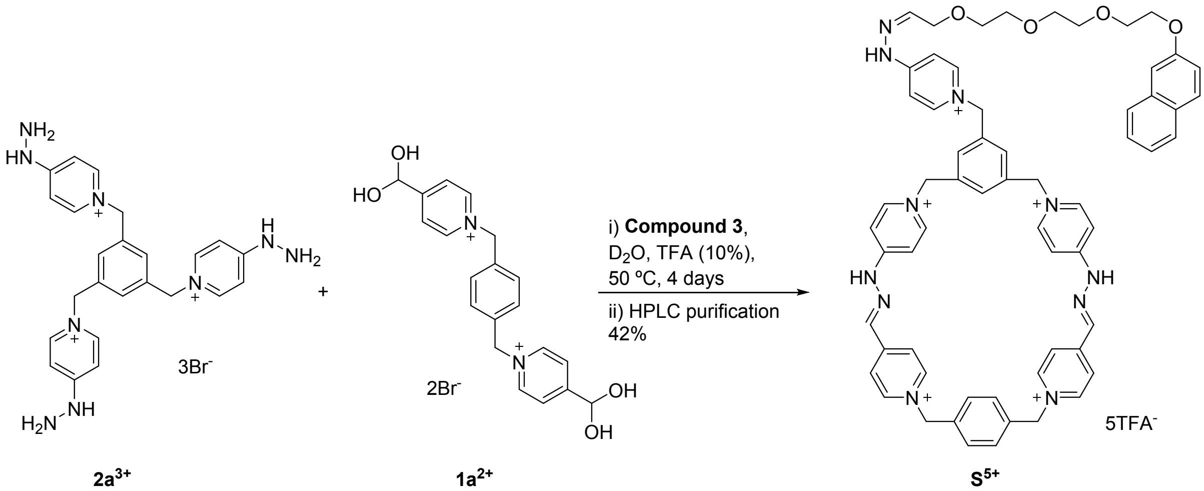

A solution of $\mathbf{1_a}^{2+}$ (1.5 µmol), $\mathbf{2_a}^{3+}$ (1.5 µmol), **Compound 3** (0.7 mg, 2.25 µmol) and TFA (10% molar) in 600 µL of D_2O was stirred and heated at 50 ºC for 3 days using an oil bath.

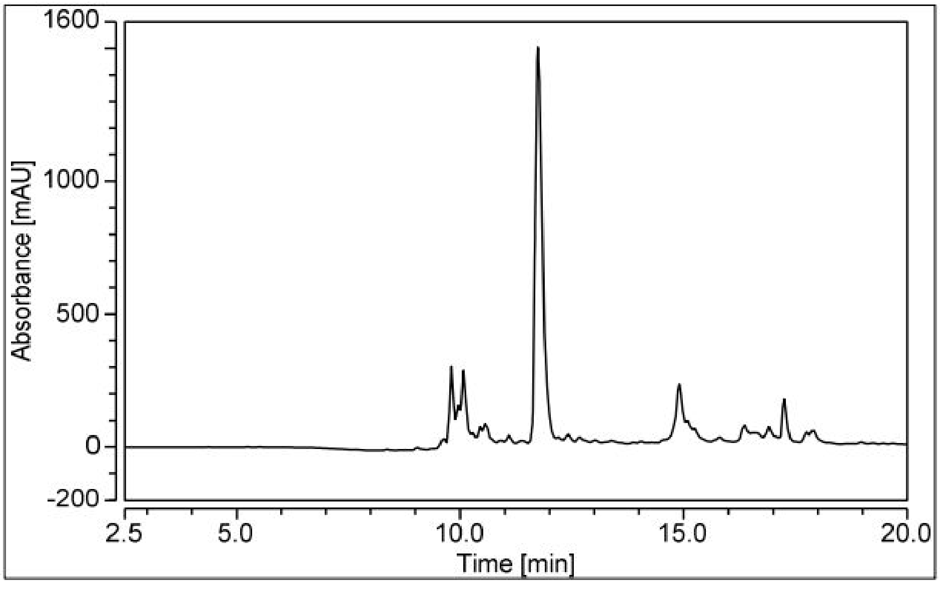

Figure S112: HPLC chromatogram (280 nm) of the raw reaction product showing a major peak at t_R = 11.7 min corresponding to S^{5+}.

The resulting solution was purified by reverse-phase HPLC (A: H_2O + 0.04% TFA, B: MeCN + 0.04% TFA), giving a yellowish solid (1.0 mg, 42%, $\mathbf{S}^{5+}$).

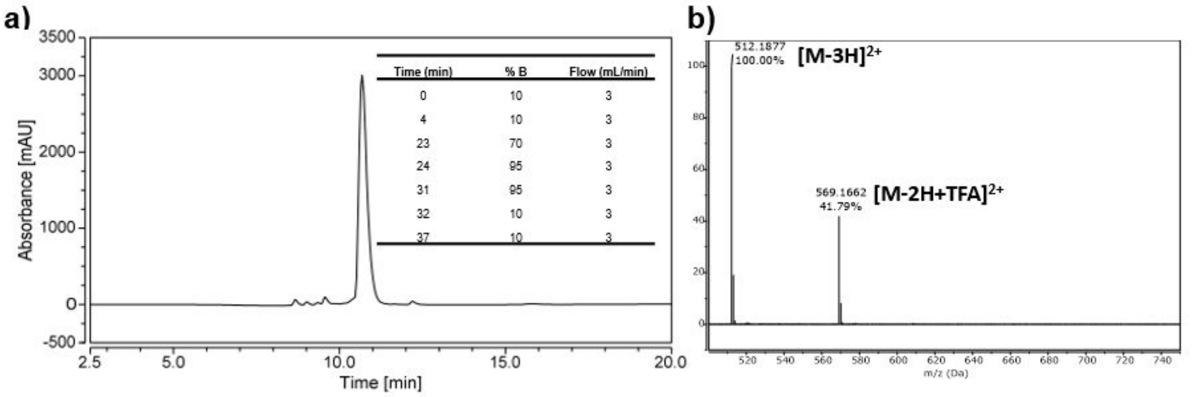

Figure S113: a) HPLC chromatogram (280 nm) of purified S^{5+} at t_R = 12.7 min (Inset: purification method). b) MS spectrum from the chromatographic peak at t_R = 10.7 min.

2.13. Hydrazone metathesis of S^{5+}.

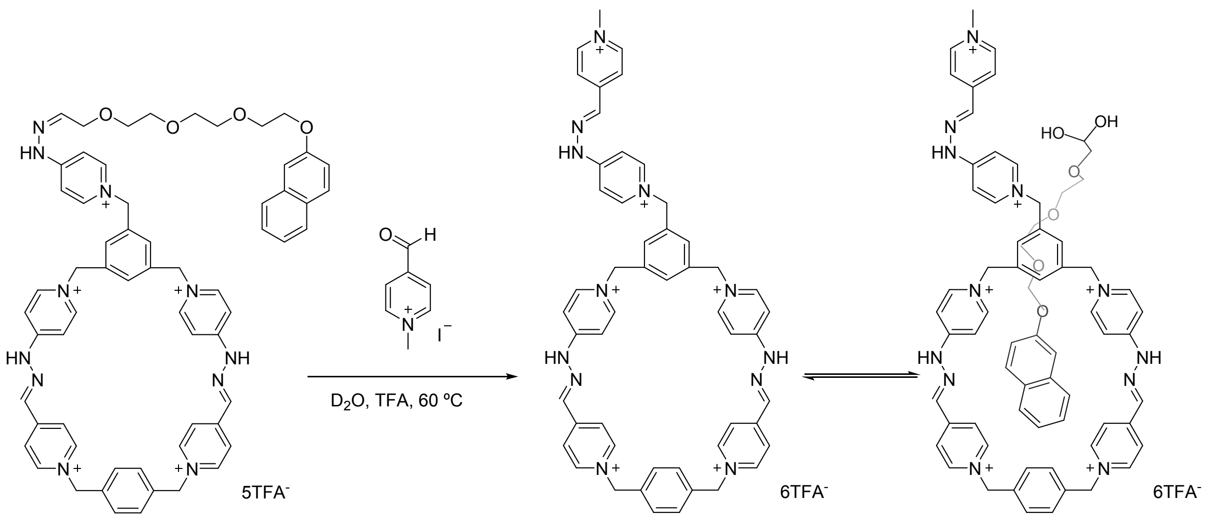

S^{5+} F_c^{6+} $3\subset F_c^{6+}$

A solution of $\mathbf{S^{5+}}$ (8 µmol), 4-formyl-*N*-methylpyridinium iodide (**Compound 6**) (48 µmol, 6 eq) and TFA (10% molar) in 200 µL of D_2O (40 mM) was heated at 50 ºC for 3 days using an oil bath. ^{1}H-NMR shows the formation of the new major species $\mathbf{F_c^{6+}}$, which was also confirmed by HPLC-MS. DOSY spectrum shows the formation of the complex $\mathbf{3\subset F_c^{6+}}$, as it shows one single diffusion for both compounds, and an extra diffusion for the excess 4-formyl-*N*-methylpyridinium iodide.

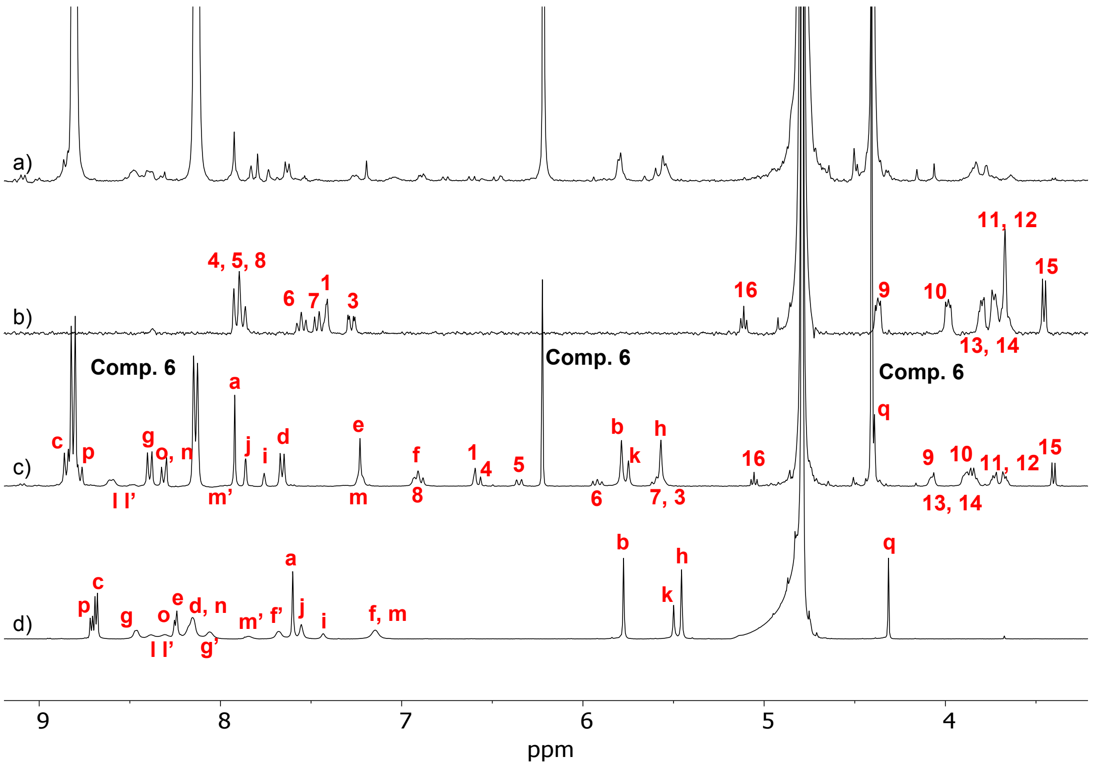

Figure S114: ^{1}H-NMR (300 MHz, D_2O) comparison spectra of the hydrazone metathesis from S^{5+} to F_c^{6+}, a) t = 0, b) linker 3, c) t = 3 days and d) F_c^{6+}.

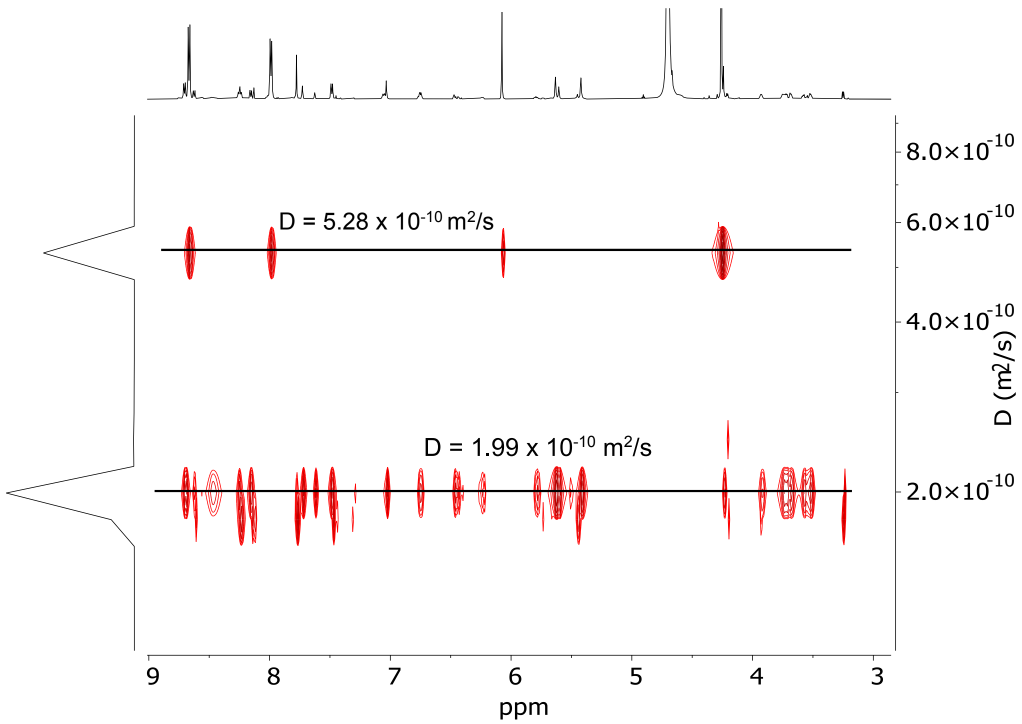

Figure S115: DOSY (500 MHz, D_2O) spectrum of the reaction products at 40 mM.

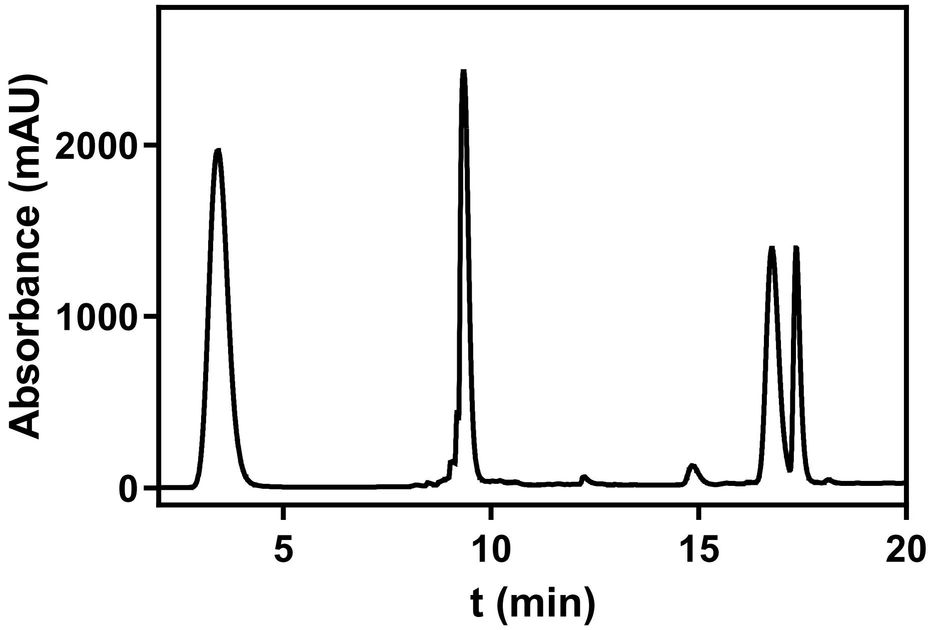

Figure S116: HPLC chromatogram (220 nm) of the raw reaction product showing a major peak at t_R = 9.3 min corresponding to F_c^{6+}. Excess Compound 6 chromatographic peak is shown at t_R = 3.4 min.

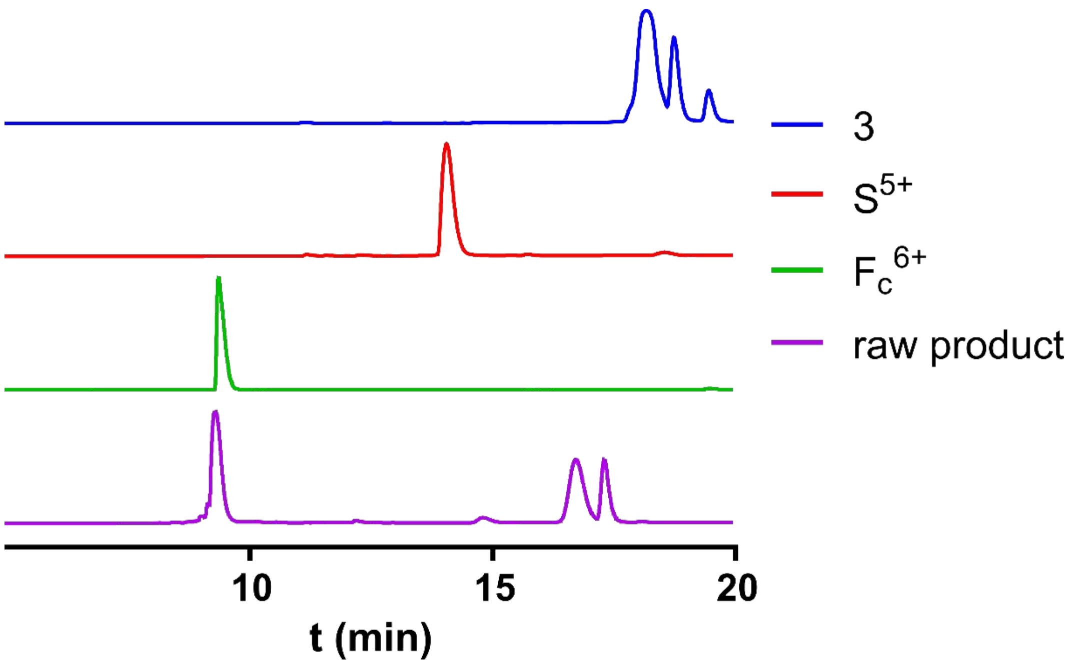

Figure S117: HPLC chromatogram (220 nm) comparison for the hydrazone metathesis reaction.

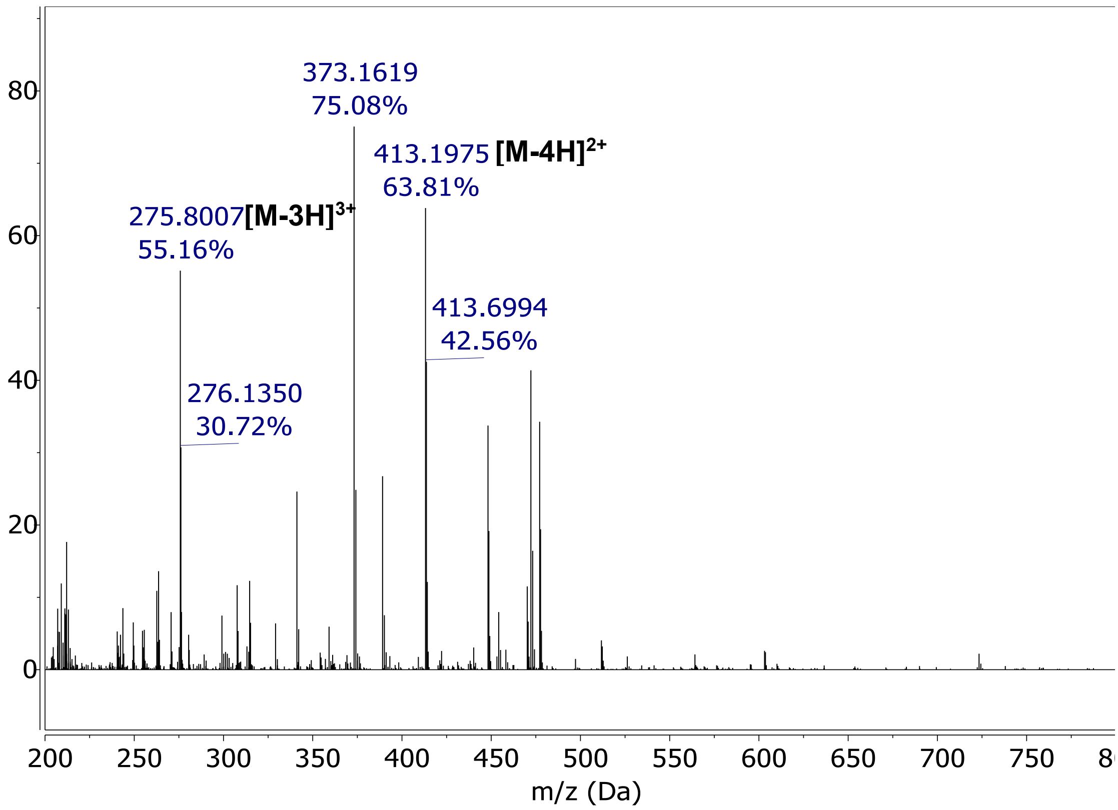

Figure S118: ESI-HRMS of the raw product F_c^{6+} (40 mM).

2.14. Characterization of F_c^{6+}.

A fraction of the hydrazone metathesis product from the previous section (50 µL, 2 µmol) was purified by reverse-phase HPLC (A: H_2O + 0.1% TFA, B: MeCN + 0.1% TFA), giving a yellowish solid (1.2 mg, 40%, $\mathbf{F_c}^{6+}$).

^{1}H-NMR (D_2O, 500 MHz): δ = 8.71 (d, J = 6.4 Hz, 2H), 8.69 (d, J = 6.5 Hz, 4H), 8.46 (s, 2H), 8.38 (s, 2H), 8.31 (s, 2H), 8.25 (d, J = 6.8 Hz, 3H), 8.16 (m, 5H), 8.06 (s, 2H), 7.84 (s, 1H), 7.68 (s, 2H), 7.60 (s, 4H), 7.55 (s, 2H), 7.43 (s, 1H), 7.15 (s, 2H), 5.78 (s, 4H), 5.50 (s, 2H), 5.46 (s, 4H), 4.31 (s, 3H). ^{13}C{^{1}H}-NMR (126 MHz, D_2O): δ = 154.6 (C), 154.5 (C), 149.8 (C), 149.0 (C), 145.2 (CH), 144.0 (CH), 143.9 (CH), 143.0 (CH), 141.7 (CH), 140.9 (CH), 136.1 (C), 134.6 (C), 131.0 (CH), 130.2 (CH), 129.7 (CH), 124.7 (CH), 124.5 (CH), 110.6 (CH), 110.3 (CH), 109.7 (CH), 109.2 (CH), 63.5 (CH_2), 61.0 (CH_2), 60.9 (CH_2), 47.6 (CH_3). HRMS (ESI): *m/z* calculated for $C_{51}H_{47}N_{12}^{3+}$ [*M*-3H]$^{3+}$ 475.8010, found 275.8009; *m/z* calculated for $C_{51}H_{46}N_{12}^{2+}$ [*M*-4H]$^{2+}$ 413.1979, found 413.1977; *m/z* calculated for $C_{53}H_{47}F_3N_{12}O_2^{2+}$ [*M*-3H+TFA]$^{2+}$ 470.1943, found 470.1942.

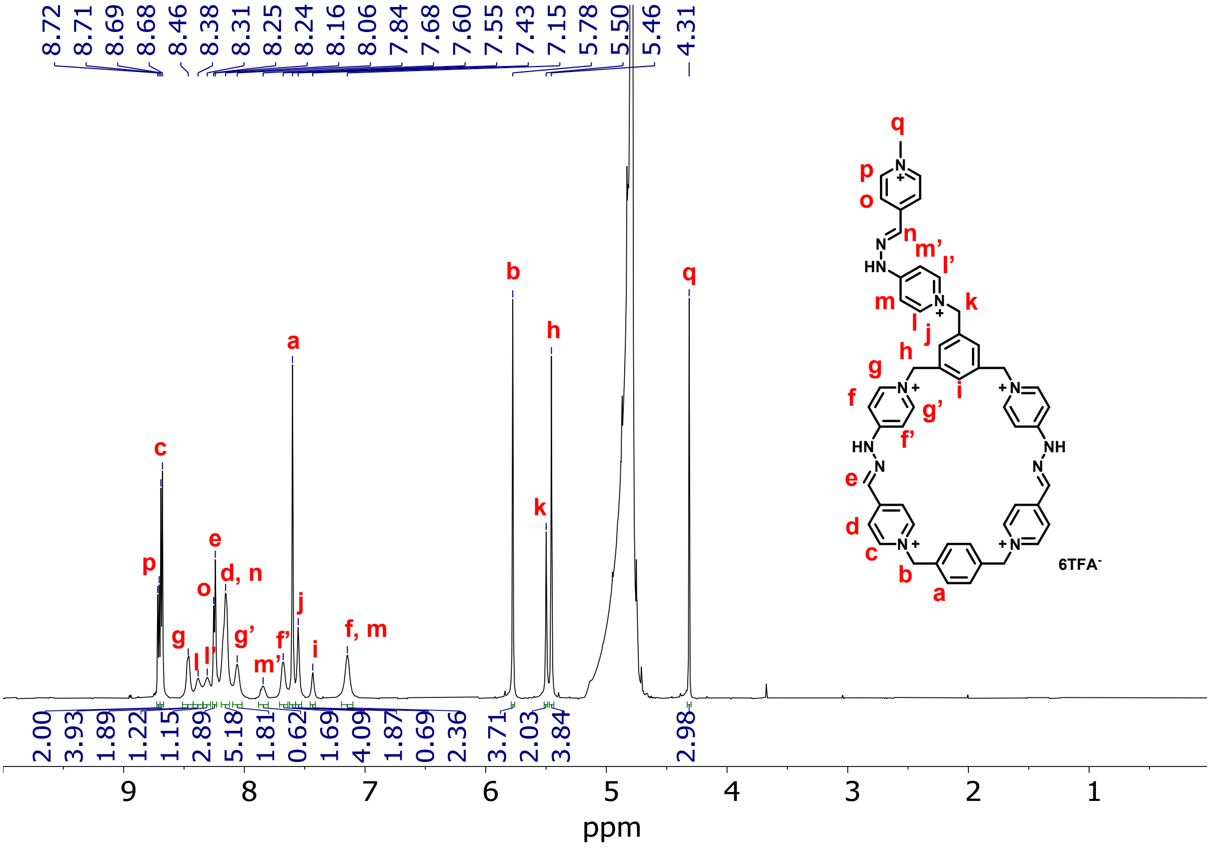

Figure S119: ^{1}H-NMR (500 MHz, D_2O) spectrum of F_c^{6+}.

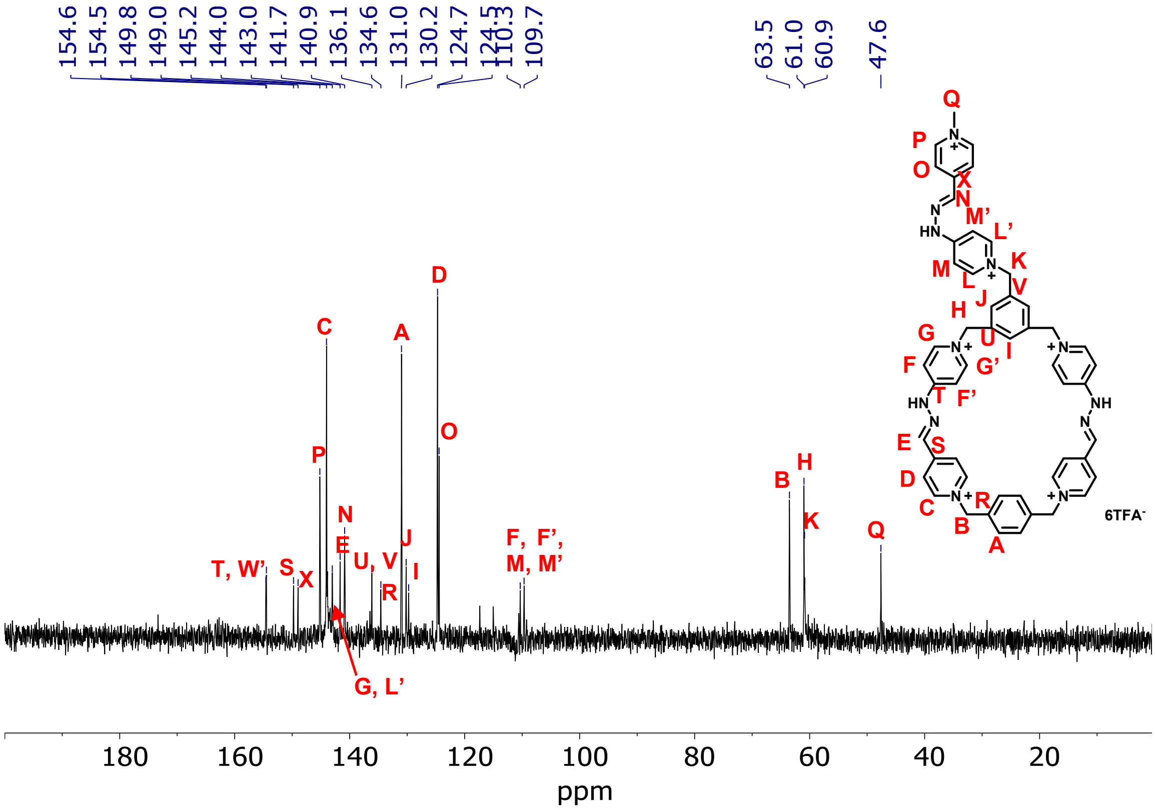

Figure S120: $^{13}C\{^{1}H\}$-NMR (126 MHz, D_2O) spectrum of F_c^{6+}.

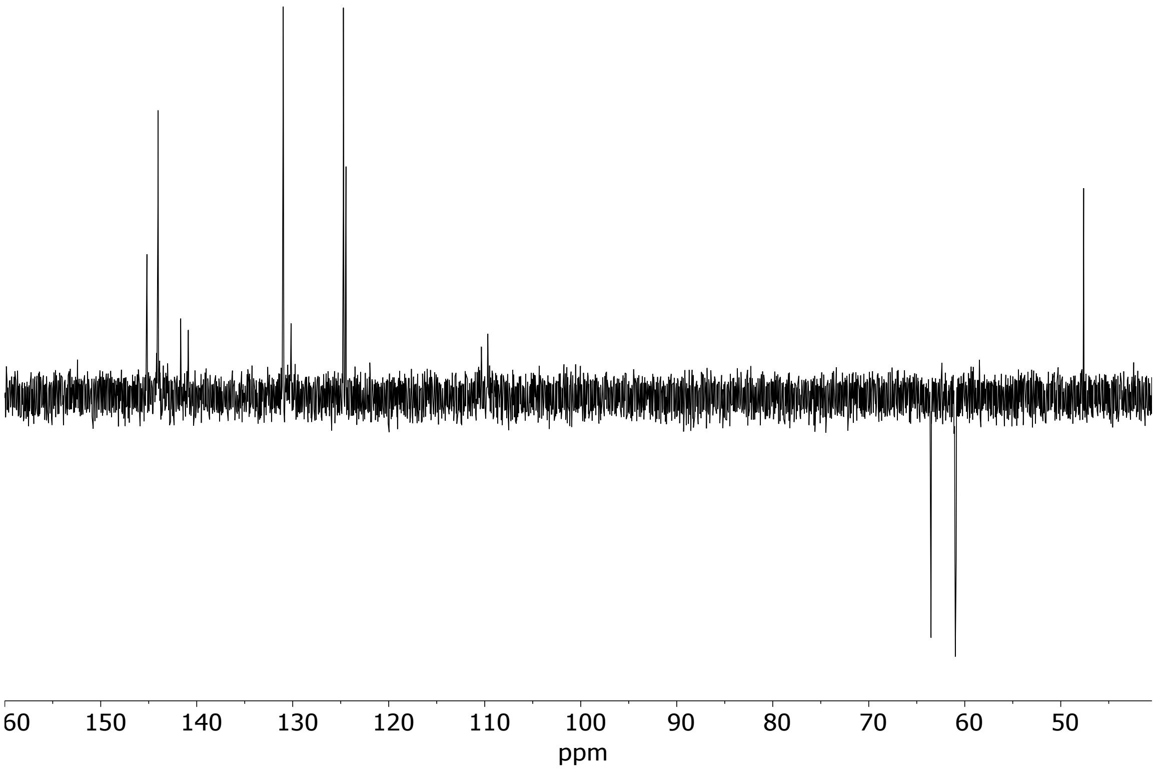

Figure S121: DEPT-135 (126 MHz, D_2O) spectrum of F_c^{6+}.

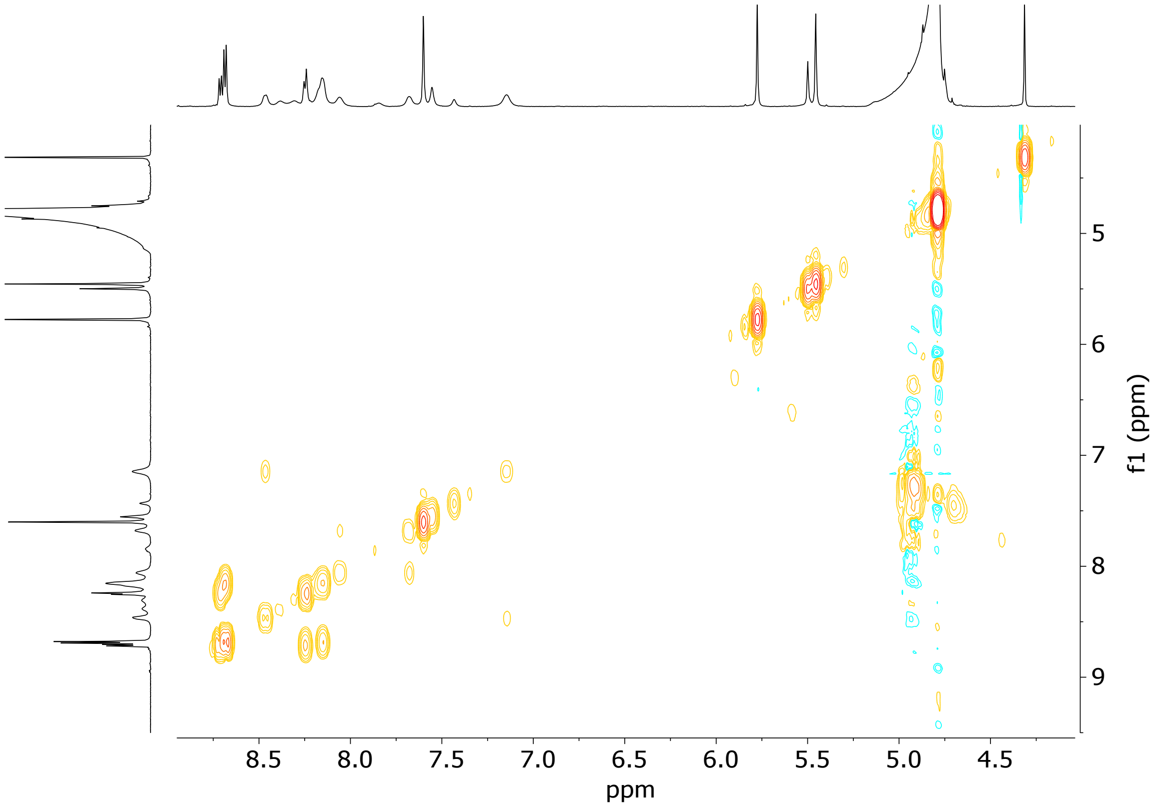

Figure S122: ^1H-^1H COSY (500 MHz, D_2O) spectrum of F_c^{6+}.

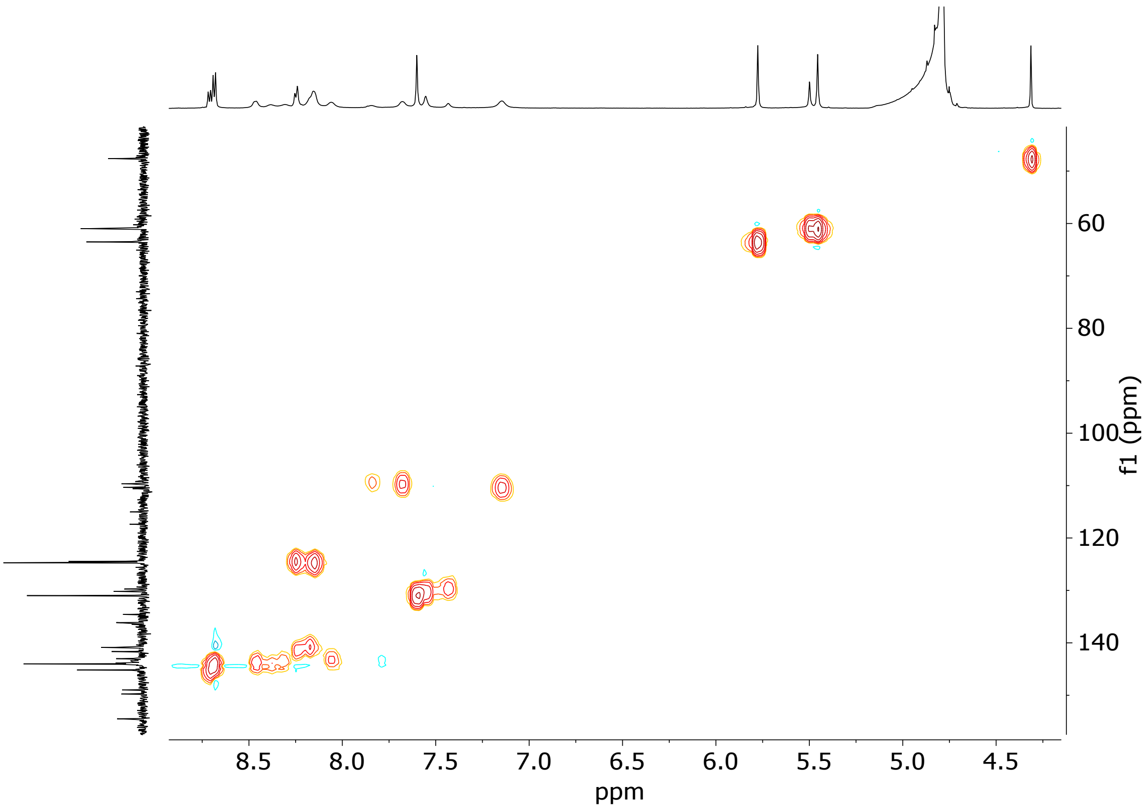

Figure S123: ^{1}H-$^{13}C\{^{1}H\}$ HSQC (500 and 126 MHz, D_2O) spectrum of F_c^{6+}.

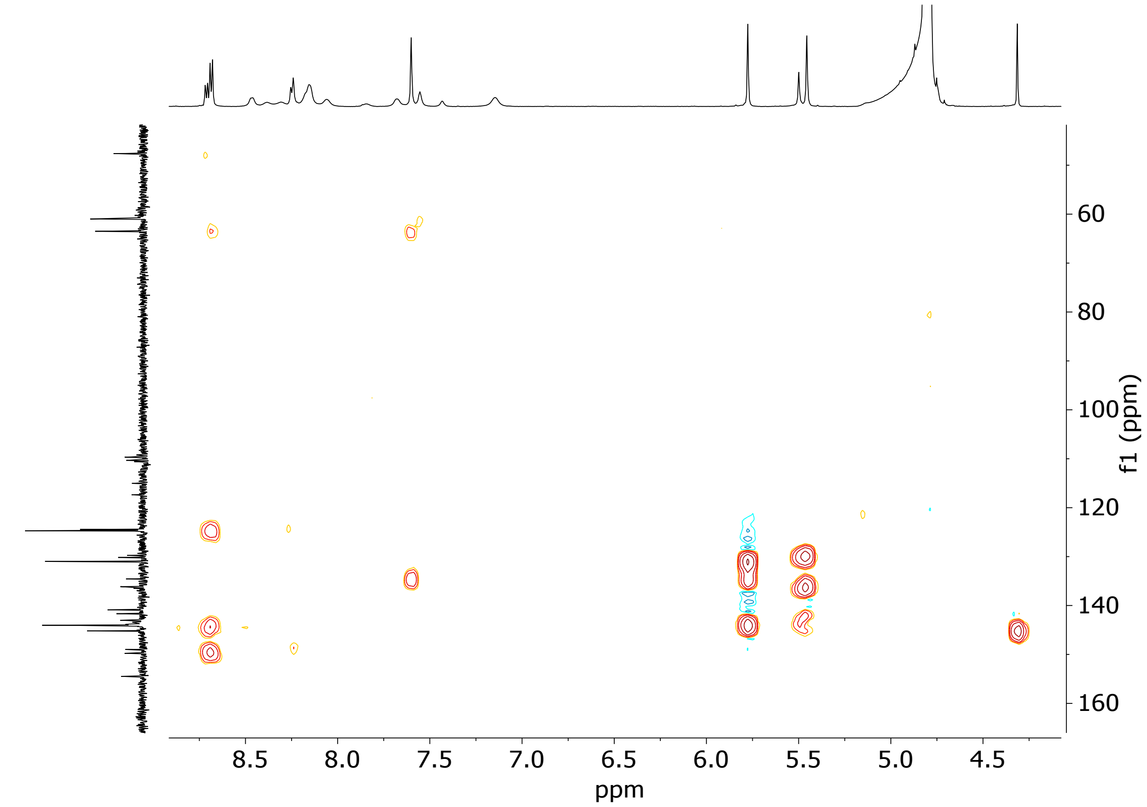

Figure S124: ^{1}H-$^{13}C\{^{1}H\}$ HMBC (500 and 126 MHz, D_2O) spectrum of F_c^{6+}.

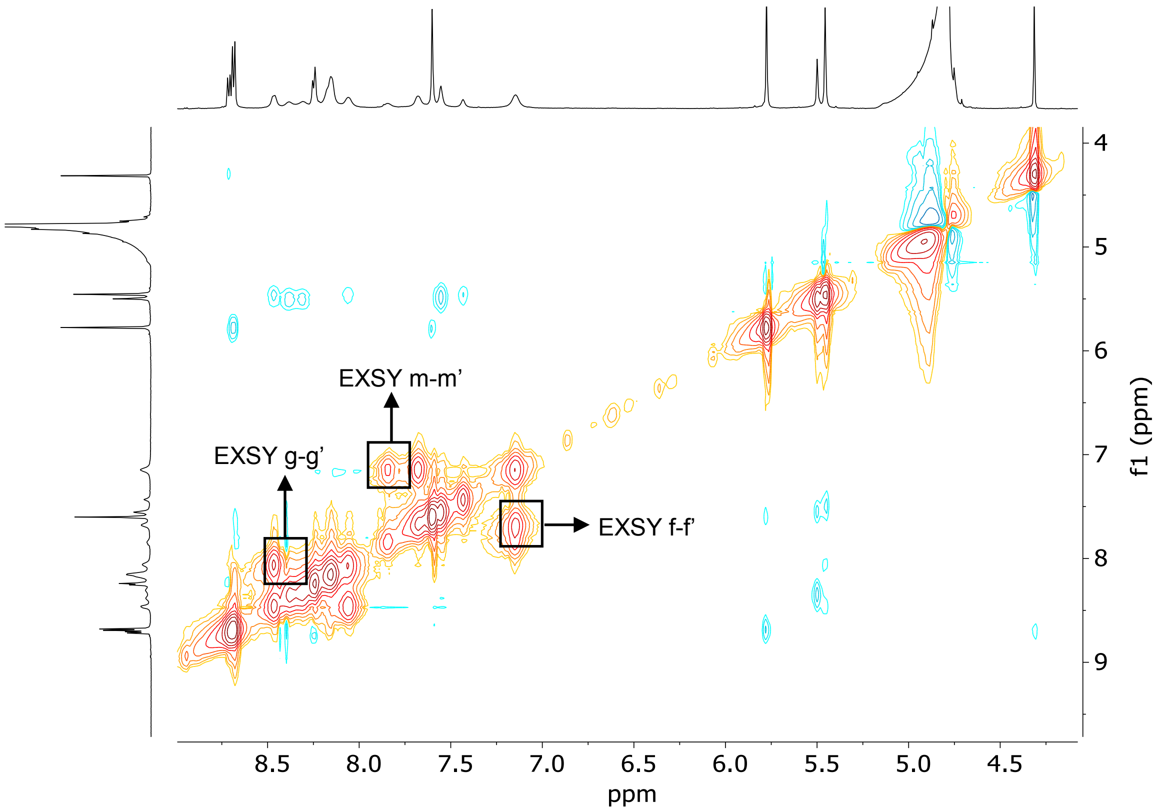

Figure S125: ^1H-^1H NOESY (500 MHz, D_2O) spectrum of F_c^{6+}.

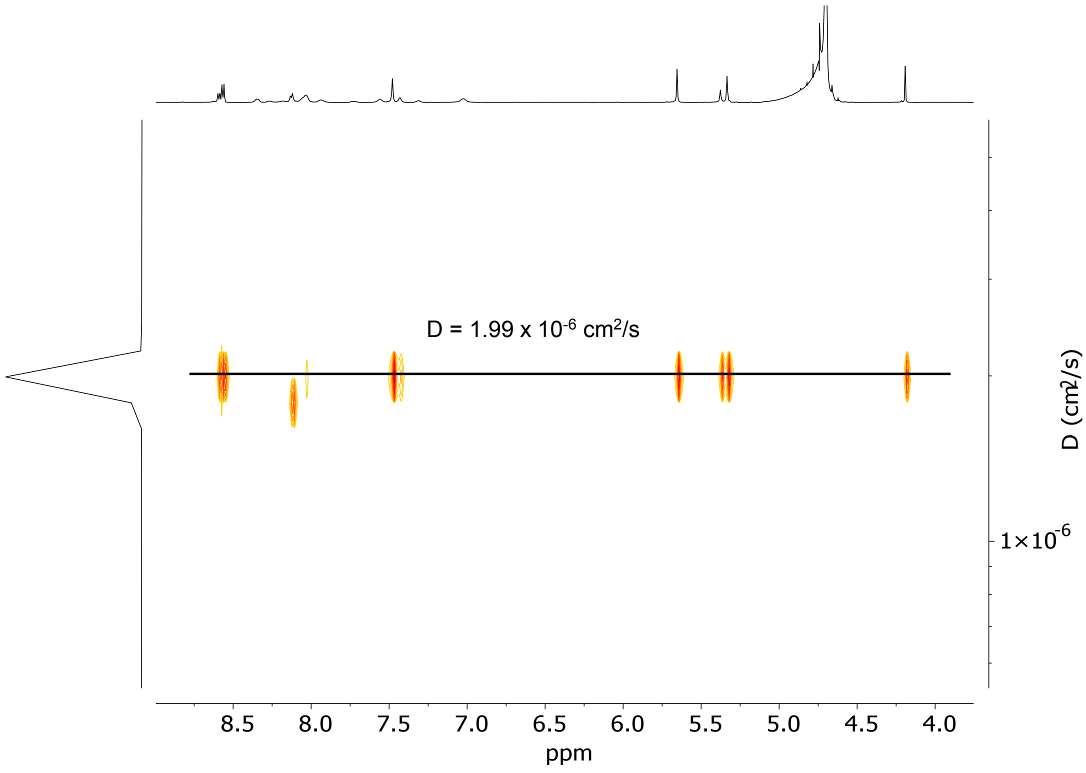

Figure S126: DOSY spectrum of F_c^{6+}.

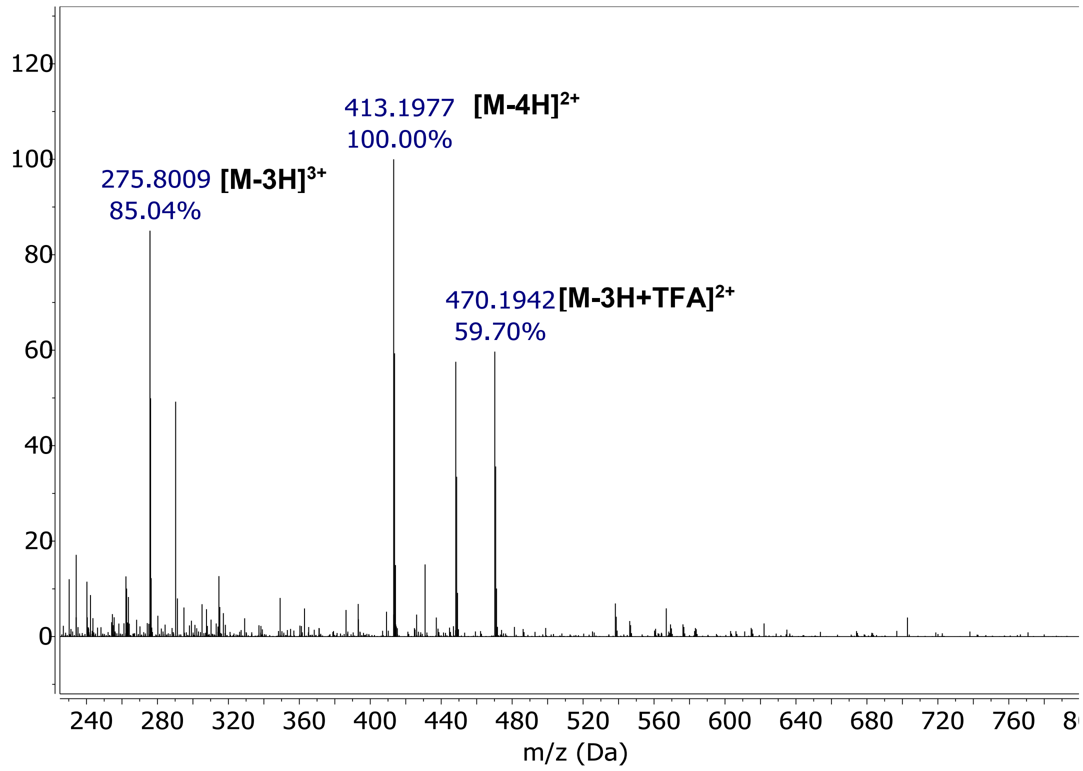

Figure S127: ESI-HRMS of F_c^{6+}.

2.15. Computational details.

All quantum mechanical calculations reported in this work were performed using the free-available program packages ORCA 5.0.3 (DFT), [8] and xTB 6.4.1 (semiempirical).[9]

2.15.1. Computation of Gibb's free energy for F_a^{5+} + 5 ⇋ 5⊂F_a^{5+}.

Initial geometries were generated by hand using the AVOGADRO software,[10] and further optimized by using the GFN2-xTB semiempirical electronic structure method,[11] and the analytical linearized Poisson-Boltzmann (ALPB)[12] solvation model (water). Conformer ensembles were obtained at the same level of theory, performing three independent conformational searches for each of the different species using the utility/driver for the xTB software CREST (conformer-rotamer sampling tool),[13] employing standard settings for $\mathbf{F_a}^{5+}$ and **5**, and the non-covalent interactions mode (NCI), in the case of **5⊂$\mathbf{F_a}^{5+}$**. The obtained ensembles for each species were further refined using Grimme's CRENSO workflow[14] with the following settings: **part0**: exclude conformers > 4 kcal/mol at B97-D3/def2-SV(P)//GFN2-XTB→ **part1**: exclude conformers > 3.5 kcal/mol at r²scan3c+ SMD(water) + $G^{\circ}_{gas,\ mRRHO}$ (GFN2[ALPB(water)-SPH]→ **part2**: geometry optimization at r²scan3c/SMD(water) and final ΔG°_{aq} at E^{DFT}_{gas} (r2scan-3c) δ_{solv} (SMD(water) +$G^{\circ}_{gas,\ mRRHO}$(GFN2[ALPB(water)-SPH]. Consequently, free energies for the $\mathbf{F_a}^{5+}$ + **5** ⇋ **5⊂$\mathbf{F_a}^{5+}$** association processes were calculated following the supramolecular approach:

$$\Delta G^{\circ}_{aq} = G^{\circ}_{aq}\text{G}{\subset}\text{H} - G^{\circ}_{aq}\text{G} - G^{\circ}_{aq}\text{H} \quad \text{(eq. 1)}$$

where for each species X the free energy in aqueous solution was computed as:

$$G^{\circ}_{aq}(X) = \left[E^{DFT}_{gas}(\text{X}) + \delta_{\text{solv}}(X)\right] + G^{\circ}_{gas,\ mRRHO}(X) \quad \text{(eq. 2)}$$

Consequently, free energies for each refined conformer ensemble were obtained out of the CRENSO workflow as the electronic energy + δ_{solv} for each ensemble at the r²scan-3c[15] using

[8] F. Neese, *Comput Mol Sci.*, 2022, **12**, e1606.
[9] C. Bannwarth, E. Caldeweyher, S. Ehlert, A. Hansen, P. Pracht, J. Seibert, S. Spicher and S. Grimme, *Comput. Mol. Sci.*, 2021, **11**, e1493.
[10] M. D. Hanwell, D. E. Curtis, D. C. Lonie, T. Vandermeersch, E. Zurek and G. R. Hutchison, *J. Cheminform.*, 2012, **4**, 17.
[11] C. Bannwarth, S. Ehlert and S. Grimme, *J. Chem. Theory Comput.*, 2019, **15**, 1652-1671.
[12] S. Ehlert, M. Stahn, S. Spicher and S. Grimme, *J. Chem. Theory Comput.*, 2021, **17**, 4250-4261.
[13] P. Pracht, F. Bohle and S. Grimme, *Phys. Chem. Chem. Phys.*, 2020, **22**, 7169-7192.
[14] S. Grimme, F. Bohle, A. Hansen, P. Pracht, S. Spicher and M. Stahn, *J. Phys. Chem. A*, 2021, **125**, 4039–4054.
[15] S. Grimme, A. Hansen, S. Ehlert and J.-M. Mewes, *J. Chem. Phys.*, 2021, **154**, 064103.

Thrular's solvation model SMD[16] to account for solvation effects in water. Energy to free energy thermoestatical contributions $G^{\circ}_{gas,\ mRRHO}(X)$ were calculated by a modified rigid rotor harmonic oscillator model, through single point hessian calculations at the GFN2-XTB level of theory on the r²scan-3c minimized structures.[17] The obtained results are compiled in **Table S4**.

**Species**	**G_i (ha)=E_{DFT}+G^t_{mRHOO}+δG_{solv}**	***Conformers in ensemble***	ΔG (kcal/mol)	K_i (M^{-1})
**F_a^{5+}**	-2307.877709	20		
**5**	-501.131330	2		
**$5\subset F_a^{5+}$**	-2809.021942	5	**-8.1**	**8.7E+05**

Table S4: Targeted quantities $\left[E^{DFT}_{gas}(\mathbf{X}) + \delta_{\text{solv}}(X)\right]$ and $G^{\circ}_{gas,\ mrrho}(X)$ for each conformer ensemble used for the estimation of the free energies of binding in water for the inclusion complex $5\subset F_a^{5+}$.

Representative structures of the lowest-lying conformer out of each ensemble were optimized using the composite method r²scan-3c/SMD(water), and confirmed as local minima by frequency analysis.

2.15.2. Local minimum for pseudo[1]rotaxane S^{5+}.

In order to obtain a representative low-lying structure for the pseudorotaxane **S**$^{5+}$, a starting geometry was generated by hand using the AVOGADRO software, and three independent conformational searches were performed using the software CREST with standard settings. The lowest-lying conformer obtained after the three runs, was then optimized using the r²scan-3c composite method and SMD(water) solvation model. The geometry was confirmed as a local minimum by frequency analysis.

2.15.3. Cartesian coordinates for representative structures.

5: local minimum on the potential energy surface at the r²scan-3c/SMD(water) level of theory.

[16] A. V. Marenich, C. J. Cramer and D. G. Truhlar, *J. Phys. Chem. B*, 2009, **113**, 6378-6396.
[17] S. Spicher and S. Grimme, *J. Chem. Theory Comput.*, 2021, **17**, 1701-1714.

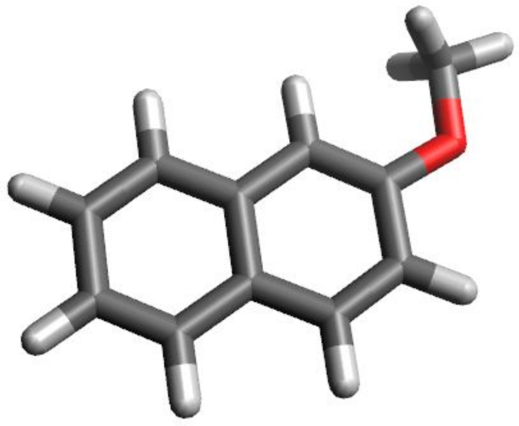

22

C	1.73000	-0.26300	0.00100
O	3.09300	-0.14200	0.00000
C	3.63400	1.18600	-0.00300
C	1.26200	-1.60300	0.00100
C	-0.08100	-1.86300	0.00000
C	-1.03000	-0.80700	0.00000
C	-0.55100	0.54000	0.00100
C	0.84500	0.79400	0.00100
C	-2.42400	-1.04900	-0.00100
C	-3.31800	-0.00200	-0.00200
C	-2.84800	1.32900	-0.00000
C	-1.49800	1.59600	0.00100
H	1.99200	-2.40700	0.00100
H	-0.43700	-2.89000	-0.00000
H	1.18400	1.82400	0.00200
H	-2.77600	-2.07800	-0.00300
H	-4.38700	-0.19600	-0.00300
H	-3.56200	2.14900	-0.00000
H	-1.13800	2.62200	0.00200
H	3.32500	1.73600	-0.90000
H	3.32900	1.73800	0.89400
H	4.71900	1.06400	-0.00500

F_a^{5+}: local minimum on the potential energy surface at the r2scan-3c/SMD(water) level of theory.

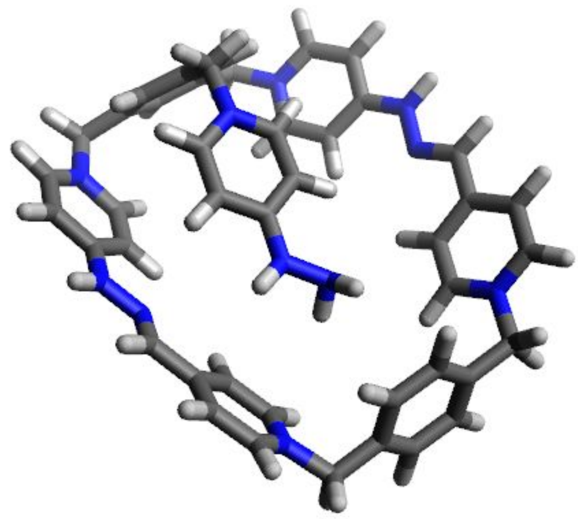

99

C	3.73974	2.91270	1.73379
N	4.87546	3.31009	1.10710
C	4.83820	4.22581	0.11703
C	3.64233	4.78354	-0.27233
C	2.45049	4.39035	0.34967
C	2.52538	3.43795	1.38300
C	1.19651	4.94379	-0.11469
N	0.09014	4.48690	0.36944
N	-1.04334	5.00352	-0.11147
C	-2.24902	4.47200	0.23396
C	-3.41329	4.97705	-0.36935
C	-4.62088	4.39842	-0.08677
N	-4.71481	3.36929	0.78847
C	-3.60325	2.89348	1.40627
C	-2.36816	3.42104	1.16394
C	6.15495	2.65539	1.48281
C	-5.95738	2.56014	0.87457
C	6.15081	1.19671	1.10176
C	6.46108	0.23217	2.05708
C	6.47809	-1.11586	1.71127
C	6.18088	-1.51166	0.40895
C	5.87112	-0.54188	-0.55024
C	5.85414	0.80197	-0.20645
C	-5.87697	-2.46958	1.11290
C	6.17716	-2.96639	0.04360
N	-4.68984	-3.34538	0.95404
N	4.79365	-3.51551	-0.14452
C	-4.75795	-4.47629	0.21186

C	-3.62459	-5.14324	-0.16709	C	-1.67358	-0.80495	-1.75427
C	-2.36795	-4.62152	0.18372	N	1.35324	1.02394	-0.86293
C	-2.31603	-3.46939	0.99304	N	2.17659	0.05557	-0.26710
C	-3.48396	-2.85888	1.34698	H	3.85570	2.17121	2.51747
C	3.74531	-3.00017	0.53889	H	5.78382	4.48896	-0.34390
C	2.49112	-3.53880	0.41278	H	3.63894	5.51562	-1.07277
C	2.28306	-4.63904	-0.43362	H	1.63675	3.10366	1.90522
C	3.39102	-5.15695	-1.11924	H	1.22745	5.71943	-0.88815
C	4.62768	-4.58027	-0.96062	H	-1.01708	5.73789	-0.82199
N	-1.24220	-5.23004	-0.28356	H	-3.36563	5.79551	-1.07991
N	-0.04395	-4.68224	-0.06737	H	-5.54217	4.72378	-0.55664
C	0.97744	-5.23520	-0.62977	H	-3.75219	2.06118	2.08590
C	-5.01273	-1.23111	-0.90180	H	-1.50486	3.01103	1.67111
C	-5.65784	-1.19247	0.33408	H	6.28514	2.77519	2.56175
C	-5.99239	0.03749	0.89817	H	6.95558	3.19811	0.97461
C	-5.70585	1.22174	0.21978	H	-6.75111	3.12299	0.37659
C	-5.06016	1.17180	-1.01405	H	-6.22180	2.43880	1.92826
C	-4.69174	-0.05142	-1.56527	H	6.68669	0.53434	3.07618
C	-3.79541	-0.08865	-2.77576	H	6.71446	-1.86535	2.46190
N	-2.39292	0.17885	-2.35540	H	5.64496	-0.84294	-1.57027
C	-1.88881	1.44053	-2.41781	H	5.61475	1.54788	-0.96095
C	-0.65050	1.73456	-1.93129	H	-6.74865	-3.02540	0.75623
C	0.13169	0.72116	-1.32191	H	-6.01471	-2.25508	2.17643
C	-0.42975	-0.57644	-1.23961	H	6.65653	-3.56599	0.82359

H	6.70193	-3.13552	-0.89993	H	-6.44026	0.07573	1.88841
H	-5.74743	-4.80992	-0.07910	H	-4.78577	2.08981	-1.53051
H	-3.71126	-6.04061	-0.77027	H	-3.81635	-1.06893	-3.25806
H	-1.37519	-3.04486	1.31722	H	-4.06630	0.67499	-3.50800
H	-3.50159	-1.94985	1.93893	H	-2.51621	2.19000	-2.88776
H	3.94898	-2.15494	1.18567	H	-0.26900	2.74618	-2.02529
H	1.67641	-3.09865	0.97478	H	0.11742	-1.39296	-0.78593
H	3.28843	-6.00584	-1.78660	H	-2.13657	-1.78638	-1.71870
H	5.51215	-4.94202	-1.47320	H	1.70170	1.96840	-0.99925
H	-1.33800	-6.05412	-0.88116	H	2.50457	0.41950	0.62649
H	0.89701	-6.12171	-1.26746	H	3.00066	-0.08117	-0.85161
H	-4.70416	-2.17880	-1.33772				

5⊂F$_a$$^{5+}$: local minimum on the potential energy surface at the r2scan-3c/SMD(water) level of theory.

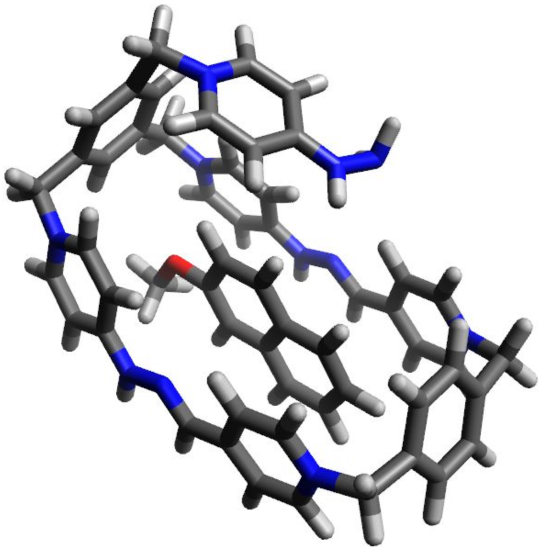

121

C	1.17600	1.09800	-1.07100
O	2.47400	1.40600	-1.35600

C	2.72900	2.12400	-2.57300	C	5.66700	2.88600	1.45000
C	-3.68000	-3.41100	-0.53600	C	-6.42500	1.76200	2.48800
N	-4.91100	-3.18500	-1.05500	N	4.29800	3.38300	1.14200
C	-5.05200	-2.64800	-2.28700	N	-5.22500	2.47600	1.95400
C	-3.94800	-2.33300	-3.04200	C	3.25900	2.95000	1.89800
C	-2.66100	-2.53700	-2.52600	C	1.96900	3.28400	1.59900
C	-2.54900	-3.10200	-1.24500	C	1.71700	4.08400	0.46800
C	-1.52100	-2.12400	-3.31300	C	2.80900	4.56500	-0.27300
N	-0.33100	-2.19500	-2.81500	C	4.07700	4.18400	0.07200
N	0.66700	-1.76900	-3.59700	C	-4.03000	2.32100	2.57400
C	1.93800	-1.65700	-3.12400	C	-2.88400	2.84200	2.03300
C	2.92800	-1.12000	-3.96600	C	-2.94100	3.54200	0.81700
C	4.19200	-0.92400	-3.48300	C	-4.19300	3.69100	0.20400
N	4.51700	-1.25800	-2.21100	C	-5.31200	3.14000	0.78100
C	3.58500	-1.81600	-1.40000	N	0.45300	4.38600	0.05900
C	2.30200	-2.03500	-1.81900	N	-0.60500	3.88100	0.69800
C	-6.11600	-3.36100	-0.18800	C	-1.76500	4.07900	0.16600
C	5.91600	-1.02500	-1.72300	C	5.74100	0.51400	2.27400
C	-6.34000	-2.09400	0.59700	C	5.77100	1.40300	1.19900
C	-7.17800	-1.09400	0.10400	C	5.83700	0.91100	-0.10500
C	-7.24900	0.13900	0.74500	C	5.92800	-0.46300	-0.33000
C	-6.48500	0.38100	1.88700	C	5.95600	-1.33700	0.75500
C	-5.69400	-0.64200	2.41500	C	5.81700	-0.85900	2.05500
C	-5.62100	-1.87100	1.77300	C	5.56900	-1.83800	3.17600

N	4.19800	-2.38900	2.99600	H	4.97900	-0.48900	-4.08900
C	3.13000	-1.60700	3.31800	H	3.89700	-2.07400	-0.39500
C	1.86700	-1.93300	2.92900	H	1.58900	-2.46800	-1.12800
C	1.64900	-3.08900	2.13700	H	-5.92100	-4.21500	0.46400
C	2.77100	-3.89200	1.82700	H	-6.95800	-3.58900	-0.84400
C	4.01100	-3.51100	2.25800	H	6.37900	-0.33800	-2.43400
N	0.40900	-3.38900	1.72200	H	6.44200	-1.98400	-1.76100
N	0.08200	-4.47400	0.89400	H	-7.75600	-1.26900	-0.80000
C	0.98400	0.43100	0.16600	H	-7.88200	0.92500	0.34200
C	-0.27700	0.07900	0.56700	H	-5.11800	-0.47100	3.32100
C	-1.40800	0.36100	-0.24100	H	-4.98900	-2.65600	2.18000
C	-1.20600	1.03400	-1.48600	H	6.36200	3.44600	0.82100
C	0.10500	1.39700	-1.88500	H	5.87500	3.11800	2.49800
C	-2.72000	0.00700	0.14900	H	-6.32100	1.72800	3.57500
C	-3.79500	0.30400	-0.65500	H	-7.30300	2.35700	2.22700
C	-3.59900	0.96800	-1.88500	H	3.50500	2.32200	2.74700
C	-2.33500	1.32400	-2.29300	H	1.16600	2.90900	2.21900
H	-3.64500	-3.84300	0.45800	H	2.66200	5.20400	-1.13700
H	-6.06600	-2.48300	-2.63300	H	4.94800	4.50200	-0.49000
H	-4.09200	-1.90300	-4.02800	H	-4.03600	1.77300	3.50900
H	-1.58300	-3.29400	-0.79300	H	-1.94500	2.69500	2.55400
H	-1.71300	-1.73300	-4.31900	H	-4.29400	4.22300	-0.73600
H	0.46900	-1.43000	-4.54100	H	-6.29800	3.21200	0.33500
H	2.69500	-0.82500	-4.98400	H	0.33400	4.91800	-0.80700

H	-1.89700	4.63100	-0.77100	H	-0.42700	-0.42100	1.52000
H	5.64100	0.89500	3.28800	H	0.23300	1.91000	-2.83200
H	5.81300	1.59900	-0.94800	H	-2.86600	-0.50600	1.09800
H	6.04100	-2.40700	0.57400	H	-4.79900	0.03000	-0.34700
H	6.26800	-2.67800	3.15400	H	-4.45700	1.19800	-2.51100
H	5.61600	-1.35500	4.15400	H	-2.18300	1.83600	-3.24100
H	3.34500	-0.71400	3.89600	H	0.62300	-4.41200	0.03200
H	1.03900	-1.29500	3.21800	H	0.34500	-5.34200	1.35900
H	2.67900	-4.80300	1.24900	H	3.81000	2.27100	-2.61200
H	4.89900	-4.09100	2.03000	H	2.40300	1.54600	-3.44500
H	-0.35200	-2.76100	1.94700	H	2.22200	3.09600	-2.56800
H	1.85400	0.21700	0.78100				

S^{5+}: local minimum on the potential energy surface at the r2scan-3c/SMD(water) level of theory.

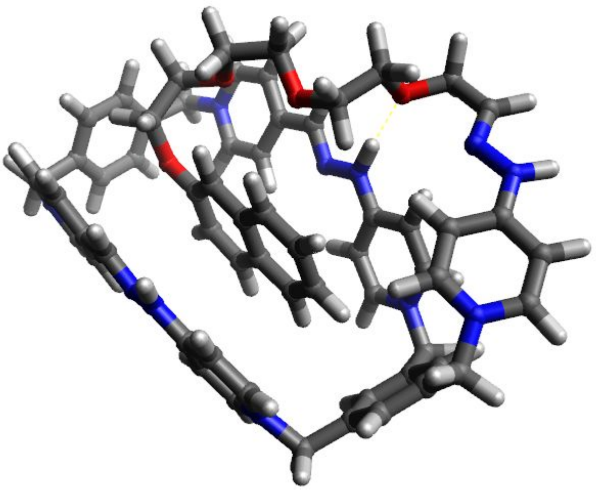

141				O	-3.65500	-0.51500	-0.40700
				C	-4.28600	-1.55300	0.37500
C	-2.29600	-0.51600	-0.58000	C	-4.50300	-1.11700	1.80800

O	-3.31100	-0.99700	2.58900	C	-6.80200	3.14800	1.20100
C	-2.89100	-2.23600	3.17000	C	5.15500	2.74800	-0.77500
C	-1.68700	-1.99500	4.04400	C	-7.07100	2.23700	0.03300
O	-0.58200	-1.54900	3.24400	C	-7.00000	2.72600	-1.27100
C	0.69300	-1.97600	3.73300	C	-7.11700	1.85900	-2.35300
C	1.08700	-1.39400	5.06700	C	-7.31500	0.49600	-2.13900
O	1.24200	0.03100	4.93800	C	-7.43500	0.01300	-0.83300
C	2.01500	0.60000	5.98200	C	-7.30900	0.87600	0.24600
C	3.49400	0.43200	5.82300	C	5.00000	-0.70100	-4.45700
C	-4.37800	3.12900	0.86900	C	-7.32300	-0.46200	-3.30100
N	-5.41800	2.90400	1.70900	N	3.66100	-1.34000	-4.31300
C	-5.21500	2.35900	2.92800	N	-6.00600	-1.15900	-3.37900
C	-3.94500	2.02400	3.34200	C	2.53500	-0.63400	-4.57400
C	-2.85400	2.22300	2.48600	C	1.29300	-1.15900	-4.34000
C	-3.10000	2.80300	1.22900	C	1.19100	-2.44700	-3.78700
C	-1.52800	1.80600	2.88900	C	2.37100	-3.17400	-3.54400
N	-0.54500	2.06600	2.09300	C	3.58000	-2.59500	-3.80400
N	0.67500	1.65800	2.44100	C	-5.86900	-2.40000	-2.86500
C	1.74000	1.96900	1.64700	C	-4.63400	-3.00200	-2.81700
C	3.01400	1.50700	2.01900	C	-3.50400	-2.32600	-3.29600
C	4.09300	1.80400	1.23800	C	-3.68200	-1.04000	-3.83600
N	3.96200	2.53100	0.09900	C	-4.93200	-0.48100	-3.85500
C	2.74100	2.98200	-0.27600	N	-0.00100	-3.01900	-3.46200
C	1.62300	2.73900	0.47600	N	-1.15400	-2.36600	-3.62100

C	-2.21300	-2.96600	-3.18600	C	0.89300	-2.26900	0.21200
C	6.32600	-1.06800	-2.35400	H	-3.74100	-2.49800	0.29200
C	5.52100	-0.22500	-3.12300	H	-5.27100	-1.69500	-0.08200
C	5.14300	1.02200	-2.62400	H	-5.20600	-1.81900	2.28300
C	5.61500	1.44700	-1.38200	H	-4.96700	-0.12400	1.80300
C	6.46800	0.62300	-0.65400	H	-3.69800	-2.64700	3.79600
C	6.79300	-0.65300	-1.11000	H	-2.65000	-2.97600	2.39200
C	7.57300	-1.59500	-0.22000	H	-1.92100	-1.25000	4.81800
N	7.07600	-1.47200	1.17200	H	-1.44100	-2.94400	4.54200
C	7.82000	-0.85500	2.12600	H	1.42100	-1.67000	2.97200
C	7.27300	-0.48100	3.32100	H	0.71500	-3.07400	3.81700
C	5.89600	-0.68400	3.55500	H	2.03600	-1.85800	5.36500
C	5.15100	-1.37500	2.57100	H	0.34100	-1.60900	5.84700
C	5.76200	-1.73700	1.40700	H	1.70900	0.20700	6.96600
N	5.33500	-0.20900	4.68700	H	1.80100	1.67800	5.99700
N	3.97500	-0.18000	4.81000	H	4.12200	0.89500	6.59500
C	-1.81200	0.56100	-1.36600	H	-4.61700	3.56900	-0.09200
C	-0.46900	0.69100	-1.61100	H	-6.09200	2.20100	3.54400
C	0.46500	-0.23600	-1.08300	H	-3.81200	1.58300	4.32300
C	-0.03300	-1.33300	-0.31600	H	-2.29400	2.98600	0.52900
C	-1.42400	-1.45900	-0.08100	H	-1.40400	1.28900	3.84600
C	1.86100	-0.10700	-1.27900	H	0.83200	1.09500	3.29300
C	2.73700	-1.01400	-0.73200	H	3.15200	0.91900	2.92100
C	2.24400	-2.10800	0.01400	H	5.09200	1.48400	1.50700

H	2.69600	3.54800	-1.20000
H	0.66900	3.12400	0.14200
H	-6.86500	4.19900	0.90700
H	-7.48700	2.96600	2.03200
H	5.93800	3.18600	-0.15000
H	4.86500	3.47100	-1.54000
H	-6.83300	3.78700	-1.44100
H	-7.04100	2.24100	-3.36700
H	-7.61400	-1.04600	-0.66300
H	-7.38300	0.49400	1.26200
H	5.66900	-1.44600	-4.89500
H	4.88700	0.12900	-5.15700
H	-7.47300	0.06100	-4.24800
H	-8.08700	-1.23300	-3.19000
H	2.67200	0.36400	-4.97500
H	0.41700	-0.56000	-4.55400
H	2.33500	-4.17800	-3.13500
H	4.51700	-3.10900	-3.62200
H	-6.77100	-2.87800	-2.50000
H	-4.55100	-3.99700	-2.39300
H	-2.84600	-0.47300	-4.22700
H	-5.12500	0.51200	-4.24500
H	0.00900	-3.94100	-3.02000
H	-2.17400	-3.95600	-2.71900
H	6.57800	-2.05900	-2.72300
H	4.47900	1.66100	-3.20100
H	6.86100	0.97300	0.29800
H	7.44400	-2.63000	-0.55000
H	8.64200	-1.36600	-0.21100
H	8.85700	-0.66000	1.88000
H	7.89500	0.01900	4.05600
H	4.10100	-1.59700	2.70900
H	5.22900	-2.24200	0.60800
H	5.91700	0.31100	5.34500
H	-2.52800	1.27600	-1.76300
H	-0.10400	1.52300	-2.20900
H	-1.76100	-2.29800	0.51300
H	2.23000	0.72600	-1.87400
H	3.80700	-0.90000	-0.88300
H	2.94000	-2.83000	0.43400
H	0.51600	-3.10900	0.79000
